# Behçet's: A Disease or a Syndrome? Answer from an Expression Profiling Study

**DOI:** 10.1371/journal.pone.0149052

**Published:** 2016-02-18

**Authors:** Ali Kemal Oğuz, Seda Taşır Yılmaz, Çağdaş Şahap Oygür, Tuba Çandar, Irmak Sayın, Sibel Serin Kılıçoğlu, İhsan Ergün, Aşkın Ateş, Hilal Özdağ, Nejat Akar

**Affiliations:** 1 Department of Internal Medicine, Ufuk University School of Medicine, Ankara, Turkey; 2 Biotechnology Institute, Ankara University, Ankara, Turkey; 3 Department of Internal Medicine, Başkent University School of Medicine, Ankara, Turkey; 4 Department of Biochemistry, Ufuk University School of Medicine, Ankara, Turkey; 5 Department of Histology and Embryology, Ufuk University School of Medicine, Ankara, Turkey; 6 Division of Nephrology, Department of Internal Medicine, Ufuk University School of Medicine, Ankara, Turkey; 7 Division of Rheumatology, Department of Internal Medicine, Ankara University School of Medicine, Ankara, Turkey; 8 Department of Pediatrics, TOBB University of Economics and Technology Hospital, Ankara, Turkey; Queen's University Belfast, UNITED KINGDOM

## Abstract

Behçet’s disease (BD) is a chronic, relapsing, multisystemic inflammatory disorder with unanswered questions regarding its etiology/pathogenesis and classification. Distinct manifestation based subsets, pronounced geographical variations in expression, and discrepant immunological abnormalities raised the question whether Behçet’s is “a disease or a syndrome”. To answer the preceding question we aimed to display and compare the molecular mechanisms underlying distinct subsets of BD. For this purpose, the expression data of the gene expression profiling and association study on BD by Xavier et al (2013) was retrieved from GEO database and reanalysed by gene expression data analysis/visualization and bioinformatics enrichment tools. There were 15 BD patients (B) and 14 controls (C). Three subsets of BD patients were generated: MB (isolated mucocutaneous manifestations, n = 7), OB (ocular involvement, n = 4), and VB (large vein thrombosis, n = 4). Class comparison analyses yielded the following numbers of differentially expressed genes (DEGs); B *vs* C: 4, MB *vs* C: 5, OB *vs* C: 151, VB *vs* C: 274, MB *vs* OB: 215, MB *vs* VB: 760, OB *vs* VB: 984. Venn diagram analysis showed that there were no common DEGs in the intersection “MB *vs* C” ∩ “OB *vs* C” ∩ “VB *vs* C”. Cluster analyses successfully clustered distinct expressions of BD. During gene ontology term enrichment analyses, categories with relevance to IL-8 production (MB *vs* C) and immune response to microorganisms (OB *vs* C) were differentially enriched. Distinct subsets of BD display distinct expression profiles and different disease associated pathways. Based on these clear discrepancies, the designation as “Behçet’s syndrome” (BS) should be encouraged and future research should take into consideration the immunogenetic heterogeneity of BS subsets. Four gene groups, namely, negative regulators of inflammation (*CD69*, *CLEC12A*, *CLEC12B*, *TNFAIP3*), neutrophil granule proteins (*LTF*, *OLFM4*, *AZU1*, *MMP8*, *DEFA4*, *CAMP*), antigen processing and presentation proteins (*CTSS*, *ERAP1*), and regulators of immune response (*LGALS2*, *BCL10*, *ITCH*, *CEACAM8*, *CD36*, *IL8*, *CCL4*, *EREG*, *NFKBIZ*, *CCR2*, *CD180*, *KLRC4*, *NFAT5*) appear to be instrumental in BS immunopathogenesis.

## Introduction

Behçet’s disease (BD) is a multisystemic inflammatory disorder with a strong and complex genetic background [1]. Now, nearly 80 years after its initial description in 1937, many important questions regarding BD still remain unanswered, not only in relation to its etiology/pathogenesis but also to its classification [2]. Besides its significant morbidity profile, BD is reported to be a cause of increased mortality among the young male patients [3].

The hallmark of BD is its recurrent mucocutaneous lesions. Nevertheless, patients with BD also display a diverse spectrum of clinical manifestations including ocular, vascular, gastrointestinal, musculoskeletal, and central nervous systems [4]. The presence of well-defined clusters/subsets of BD patients with distinctly associated manifestations of the disease, marked regional variations in the expression of BD around the globe, and the proposal stating that distinct immunological abnormalities are underlying distinct classification groups of BD raised the question whether Behçet’s is “a disease or a syndrome” [4–6]. At present, despite the massive amount of available data, this question is still not answered conclusively.

The introduction of microarray technology and its implementation for whole-genome expression analysis allowed scientists to study the differentially expressed genes (DEGs) in health and disease states at a genome-wide level [7]. With such a huge amount of high-throughput expression data in hand, bioinformatic and pathway analysis tools help researchers to delineate the pathways responsible for the development of diseases. Furthermore, the accumulation of research data in public repositories (i.e., databases open to public access) creates an opportunity for meta-analysis and data mining and thus analyzing data from different perspectives and condensing it into useful information.

With the purpose of answering the question of whether BD is a disease or a syndrome, we aimed to clarify and compare the molecular mechanisms underlying different expressions of BD. In this context, we used the expression data of the key gene expression profiling and association study on BD by Xavier et al [8]. The gene expression data provided by Xavier et al was retrieved from Gene Expression Omnibus (GEO) and reanalysed by implementation of gene expression data analysis/visualization and bioinformatics enrichment tools [9]. We obtained evidence of apparent expression profile discrepancies among BD patients with distinct expressions of the disorder. Furthermore, our findings supported the potential role of four gene groups (i.e., negative regulators of inflammation, neutrophil granule proteins, antigen processing and presentation proteins, and regulators of immune response) in BD immunopathogenesis.

## Materials and Methods

### The gene expression profiling study by Xavier *et al*

Fifteen patients with BD all diagnosed according to the revised International Criteria and 14 healthy control subjects were enrolled in the study by Xavier et al [8, 10]. Total RNA was isolated from peripheral blood mononuclear cells and GeneChip^®^ Human Genome U133 Plus 2.0 (Affymetrix, Santa Clara, CA, USA) microarrays were used for hybridization [8]. According to the specifications in its product description, the GeneChip^®^ Human Genome U133 Plus 2.0 array is a comprehensive whole human genome expression array which covers >47,000 transcripts for expression profiling. The study by Xavier et al was conducted and reported in accordance with the Minimum Information About Microarray Experiment (MIAME) guidelines and both the raw and the processed microarray data were deposited on GEO database with the Series ID GSE17114 [8, 9, 11, 12].

### Retrieval of the microarray data

The raw microarray data of the study by Xavier et al was retrieved from GEO by using the GEO accession GSE17114 on August 25^th^ 2015 [8, 9, 12]. The relevant file was a TAR file with the name “GSE17114_RAW” including 29 individually compressed CEL files (GSM428037.CEL.gz—GSM428065.CEL.gz).

### Definition of subsets of patients with Behçet’s disease

By using the principal clinical characteristics (major clinical symptoms) of BD patients briefly summarized in the article by Xavier et al, we subgrouped the BD patients according to their disease manifestations [8]. Three subsets were generated, namely, BD patients with mucocutaneous involvement (MB), BD patients with ocular involvement (OB), and BD patients with vascular involvement (VB). BD patients with isolated mucocutaneous manifestations (i.e., oral aphtosis, genital aphtosis, skin aphtosis, pseudofolliculitis, erythema nodosum, positive Pathergy test) were grouped as MB; BD patients with any kind of ocular involvement (i.e., anterior uveitis, posterior uveitis, retinal vasculitis) were grouped as OB, and BD patients demonstrating large vein thrombosis were grouped as VB. The group inclusive of all of the 15 BD patients was named as B, while the control group was given the name C.

### Pre-processing of the microarray data

Before obtaining the transcriptomic-level measurements and continuing with the downstream analysis, pre-processing of the microarray data was performed using BRB-ArrayTools v4.4.1 Stable Release developed by Dr. Richard Simon and BRB-ArrayTools Development Team [13]. The gene expression data present as raw CEL files was collated by the data import function of BRB-ArrayTools. The Robust Multiarray Average (RMA) algorithm, including background correction, log base 2 transformation, and quantile normalization was used for normalization of the microarray data [14]. Following normalization, the replicate spots within each individual array were averaged. Finally, gene filters were implemented which excluded genes if less than 20% of the genes’ expression values had at least a 1.5 fold change in either direction from the genes’ median expression values or if the genes’ missing expression values exceeded 50%.

### Verification of the manifestation based subgrouping of Behçet’s disease patients

For the verification of our manifestation based subgrouping of BD patients, an initial cluster analysis was performed. BRB-ArrayTools’ built-in clustering tools, Cluster 3.0 and TreeView softwares developed by Michael Eisen and the Stanford group were used for clustering [13, 15]. A hierarchical clustering algorithm using Euclidean distance metric and average linkage was implemented and both the patients and the genes were clustered. The gene sets used for clustering were constituted from the DEGs identified during the class comparisons MB *vs* OB, MB *vs* VB, OB *vs* VB (two-sample t-test, p≤0.001, fold change ≥3 for MB *vs* VB and ≥4 for MB *vs* OB and OB *vs* VB), and MB *vs* OB *vs* VB (ANOVA, p≤0.001).

### Analysis of the gene expression data

Class comparison analysis among BD and C groups were performed using BRB-ArrayTools [13]. For class comparison analysis of two classes, two-sample t-test with random variance model was implemented. DEGs were selected using a p-value ≤0.05 and a fold change (FC) ≥2. In only two cases, namely, for the class comparisons B *vs* C and MB *vs* C, an FC of ≥1.5 was also used. The Venn diagram representation of class comparisons was drawn with Venny 2.0.2 by Juan Carlos Oliveros [16].

The tools used (i.e., BRB-ArrayTools’ built-in clustering tools, Cluster 3.0 and TreeView softwares) and the methodology implemented (i.e., hierarchical clustering using Euclidean distance metric and average linkage) for cluster analysis of the gene expression data were exactly the same as described above [13, 15]. Similarly, patients and genes were clustered together and again, the gene sets used for clustering were constituted from the DEGs identified during the class comparisons MB *vs* OB, MB *vs* VB, OB *vs* VB (two-sample t-test, p≤0.001 and FC≥4 for all), and MB *vs* OB *vs* VB (ANOVA, p≤0.001).

Gene Ontology (GO) term enrichment analysis of the DEGs were performed with Web-Based Gene Set Analysis Toolkit (WebGestalt) and the enrichment analysis specifically focused on the sub-root of biological process (BP) [17, 18]. For the enrichment analysis of GO terms, the DEGs retrieved by the class comparisons MB *vs* C (p≤0.05 and FC≥1.5), OB *vs* C and VB *vs* C (p≤0.05 and FC≥2 for both) were implemented and the setup included the hypergeometric test, the Benjamini-Hochberg procedure for multiple test adjustment, and 2 as the minimum number of genes for a category.

**Fig 1:**
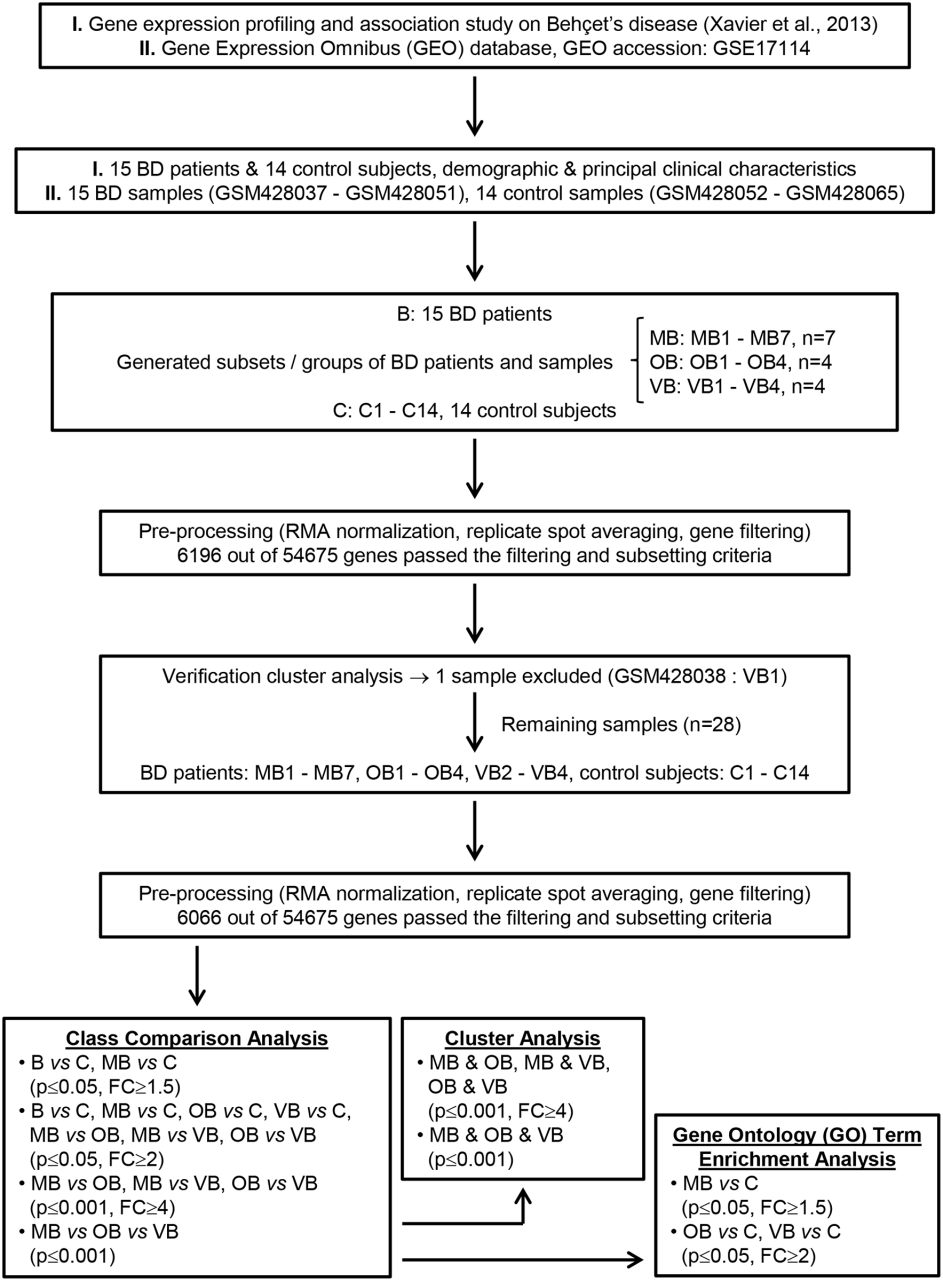
Flow diagram of the study. B, the group including all of the Behçet’s disease patients; BD, Behçet’s disease; C, control group; FC, fold change; MB, Behçet’s disease patients with isolated mucocutaneous manifestations; OB, Behçet’s disease patients with any kind of ocular involvement; RMA, robust multiarray average; VB, Behçet’s disease patients with large vein thrombosis.

The flow diagram of the study is shown in Fig 1.

### Matching of the loci of the differentially expressed genes with the loci identified in the genome-wide association and the genome-wide linkage studies of Behçet’s disease

In order to document the matches between the loci identified in the genome-wide association (GWA) and the genome-wide linkage (GWL) studies of BD and the loci of the DEGs defined in the present study, the linkage study by Karasneh et al, and the two association studies by Meguro et al and Kirino et al were employed [19–21]. A total of 25 non-HLA loci were included and the DEGs identified during the class comparisons MB *vs* C (p≤0.05 and FC≥1.5), OB *vs* C and VB *vs* C (p≤0.05 and FC≥2 for both) were utilized.

### Statistical analysis

Demographic data analysis of the study population was carried out using the SPSS 17 software (SPSS Statistics for Windows, Version 17.0, Chicago, SPSS Inc.). Ages were expressed as mean±SD and gender as ratios (M/F). For comparing the means of two independent groups the Mann-Whitney *U* test was implemented, whereas comparison of the ratios of two independent groups was performed by the chi-square (χ2) test. p≤0.05 was considered to be statistically significant.

## Results

### Demographic and clinical characteristics

Demographic and basic clinical characteristics of the study population is presented in Table 1. The ID, GEO sample, group, gender, and age columns were reproduced from Xavier et al and GEO database (GSE17114) [8, 12]. BD patients and control subjects were similar with respect to their ages (mean±SD, B: 37.1±11.0, C: 36.7±13.5, p = 0.939) and gender (M/F ratio, B: 7/8, C: 7/7, p = 0.858). There were 7 (MB1-MB7), 4 (OB1-OB4), and 4 (VB1-VB4) patients in the MB, OB, and VB subsets respectively, whereas 14 (C1-C14) subjects in group C.

**Table 1:** Demographic and basic clinical characteristics of the study population.^a^^,^ ^b^

ID	GEO Sample	Group	Gender	Age^c^	M	O	V	BD Subset	Remarks^d^
1	GSM428037	Patient	F	29	+	-	-	Mucocutaneous	MB1
2	GSM428038	Patient	F	40	+	-	+	Vascular	VB1, Excl.^e^
3	GSM428039	Patient	F	36	+	-	-	Mucocutaneous	MB2
4	GSM428040	Patient	F	29	+	-	-	Mucocutaneous	MB3
5	GSM428041	Patient	F	55	+	-	-	Mucocutaneous	MB4
6	GSM428042	Patient	F	30	+	-	-	Mucocutaneous	MB5
7	GSM428043	Patient	F	44	+	+	-	Ocular	OB1
8	GSM428044	Patient	F	46	+	-	+	Vascular	VB2
9	GSM428045	Patient	M	20	+	-	-	Mucocutaneous	MB6
10	GSM428046	Patient	M	57	+	+	-	Ocular	OB2
11	GSM428047	Patient	M	50	+	-	+	Vascular	VB3
12	GSM428048	Patient	M	30	+	-	+	Vascular	VB4
13	GSM428049	Patient	M	33	+	+	-	Ocular	OB3
14	GSM428050	Patient	M	29	+	-	-	Mucocutaneous	MB7
15	GSM428051	Patient	M	28	+	+	-	Ocular	OB4
16	GSM428052	Control	F	27	-	-	-	-	C1
17	GSM428053	Control	F	32	-	-	-	-	C2
18	GSM428054	Control	F	62	-	-	-	-	C3
19	GSM428055	Control	F	51	-	-	-	-	C4
20	GSM428056	Control	F	26	-	-	-	-	C5
21	GSM428057	Control	F	26	-	-	-	-	C6
22	GSM428058	Control	F	46	-	-	-	-	C7
23	GSM428059	Control	M	35	-	-	-	-	C8
24	GSM428060	Control	M	42	-	-	-	-	C9
25	GSM428061	Control	M	31	-	-	-	-	C10
26	GSM428062	Control	M	28	-	-	-	-	C11
27	GSM428063	Control	M	61	-	-	-	-	C12
28	GSM428064	Control	M	26	-	-	-	-	C13
29	GSM428065	Control	M	21	-	-	-	-	C14

BD, Behçet’s disease; C, control group; GEO, Gene Expression Omnibus; M, mucocutaneous manifestations; MB, Behçet’s disease patients with isolated mucocutaneous manifestations; O, ocular manifestations; OB, Behçet’s disease patients with any kind of ocular involvement; V, large vein thrombosis; VB, Behçet’s disease patients with large vein thrombosis.

^a^ The ID, GEO sample, group, gender, and age columns are reproduced from Xavier et al and GEO database (GSE17114) [8, 12].

^b^ Clinical manifestations (M, O, and V) are adapted from Xavier et al and BD subsets are assigned according to these clinical manifestations [8].

^c^ Age-at-evaluation.

^d^ Sample/experiment names are given in the “Remarks” column.

^e^ Excluded based on initial verification cluster analysis results.

### Verification cluster analysis

**Fig 2:**
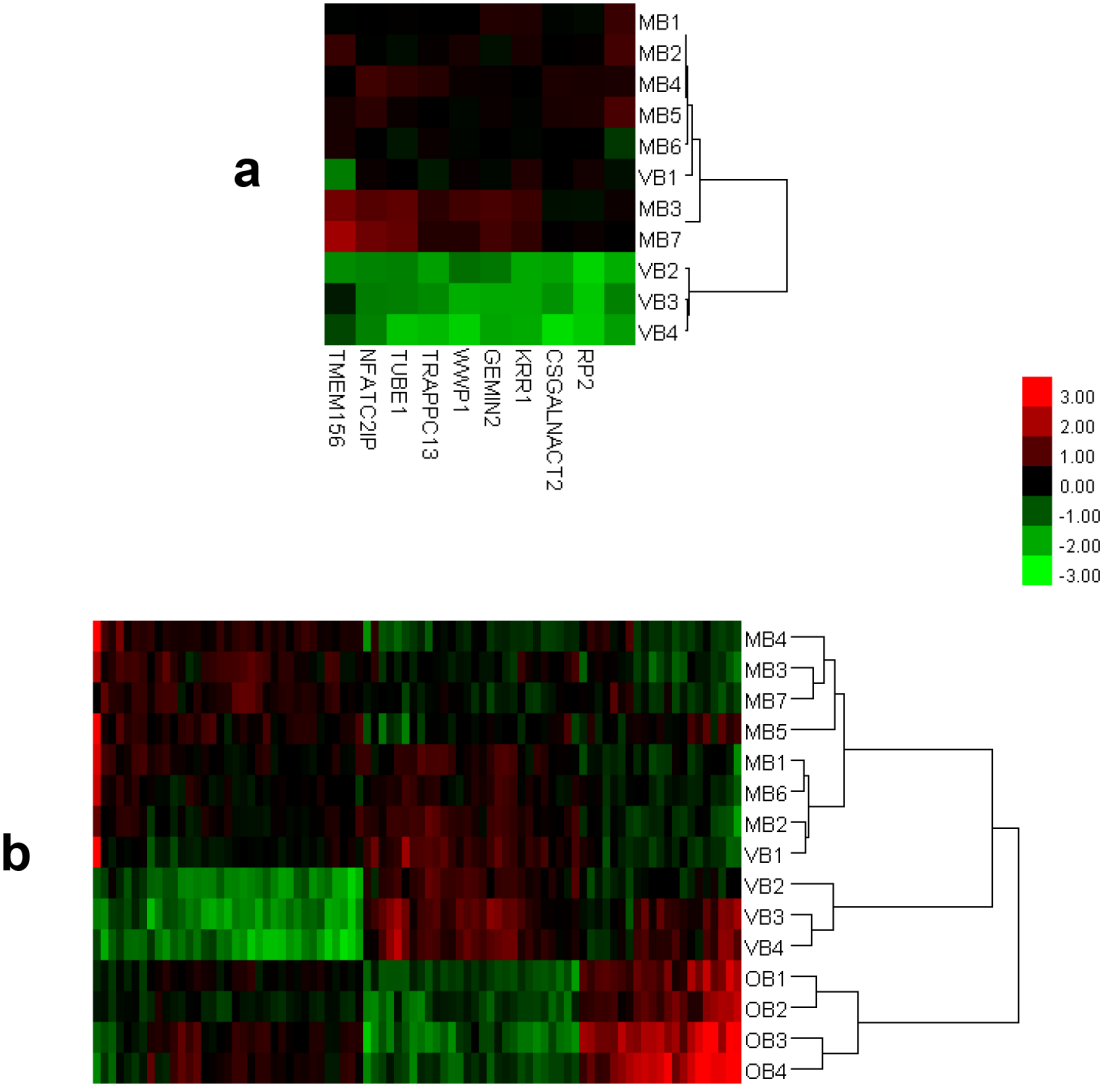
Dendrogram and heatmap representations of the results of the initial cluster analysis “MB & VB” (a) and “MB & OB & VB” (b). For both cases, hierarchical clustering using Euclidean metric and average linkage was employed and both patients and genes were clustered. For sake of simplicity, only the dendrograms for clustering of the patients are shown in the heatmaps. Take note of the position of VB1 (study ID 2, GSM428038) in the MB branch of the dendrograms. Based on these clustering results, VB1 was excluded prior to further analysis. Also, as can be seen in the figure, the cluster analysis successfully clustered distinct expressions of Behçet’s disease and with the exception of VB1, the expression profiling based clustering results were in accordance with the manifestation based clustering of Behçet’s disease patients. The gene sets used for clustering were constituted from the DEGs identified during the corresponding class comparisons (i.e., MB *vs* VB and MB *vs* OB *vs* VB).

During initial clustering, the sample with the experiment name VB1 (study ID 2, GEO accession number GSM428038) appeared to belong to a different BD subset (i.e., MB) instead of its originally assigned BD subset (i.e., VB). The dendrogram and heatmap representations of the clustering experiments “MB & VB” and “MB & OB & VB” are depicted in Fig 2. Subsequently, this sample was excluded prior to further analysis and thus the number of samples in group B and VB dropped to 14 and 3 respectively. Except the case with VB1, the expression profiling based clustering results exactly matched the manifestation (clinical) based clustering of BD patients.

The experiment descriptor file (EDF) of the study is given in the S1 File, while the gene lists used for initial clustering are presented in the S2 File.

### Class comparison analysis

The number of the DEGs found during class comparison analysis between BD and C groups are summarized in Table 2. The class comparison B *vs* C yielded a DEG number of 4 and when an FC of ≥1.5 was implemented, the number of the DEGs increased to 20. Similarly, the class comparison MB *vs* C also documented a very low number of DEGs (i.e., 5) which increased to 71 again with an FC of ≥1.5. Interestingly, class comparison analysis between the two other BD subsets and C (i.e., OB *vs* C and VB *vs* C) and also class comparison analysis of BD subsets among themselves yielded significantly higher numbers of DEGs (Table 2). The number of the DEGs for class comparisons OB *vs* C, VB *vs* C, MB *vs* OB, MB *vs* VB, and OB *vs* VB were 151, 274, 215, 760, and 984 respectively. The gene lists of the DEGs are presented in the S3 File.

**Table 2:** Summary of key results of the class comparison analysis.

Compared Classes	Number of Differentially Expressed Genes					
	p≤0.05, FC≥1.5			p≤0.05, FC≥2.0		
	Total	Increased^a^	Decreased^b^	Total	Increased^a^	Decreased^b^
**B *vs* C**	20	9	11	4	2	2
**MB *vs* C**	71	48	23	5	1	4
**OB *vs* C**	-	-	-	151	47	104
**VB *vs* C**	-	-	-	274	53	221
**MB *vs* OB**	-	-	-	215	128	87
**MB *vs* VB**	-	-	-	760	626	134
**OB *vs* VB**	-	-	-	984	481	503

B, the group including all of the Behçet’s disease patients; FC, fold change.

^a^ Increased expression in the first class (e.g., B) with respect to the second class (e.g., C).

^b^ Decreased expression in the first class (e.g., B) with respect to the second class (e.g., C).

**Fig 3:**
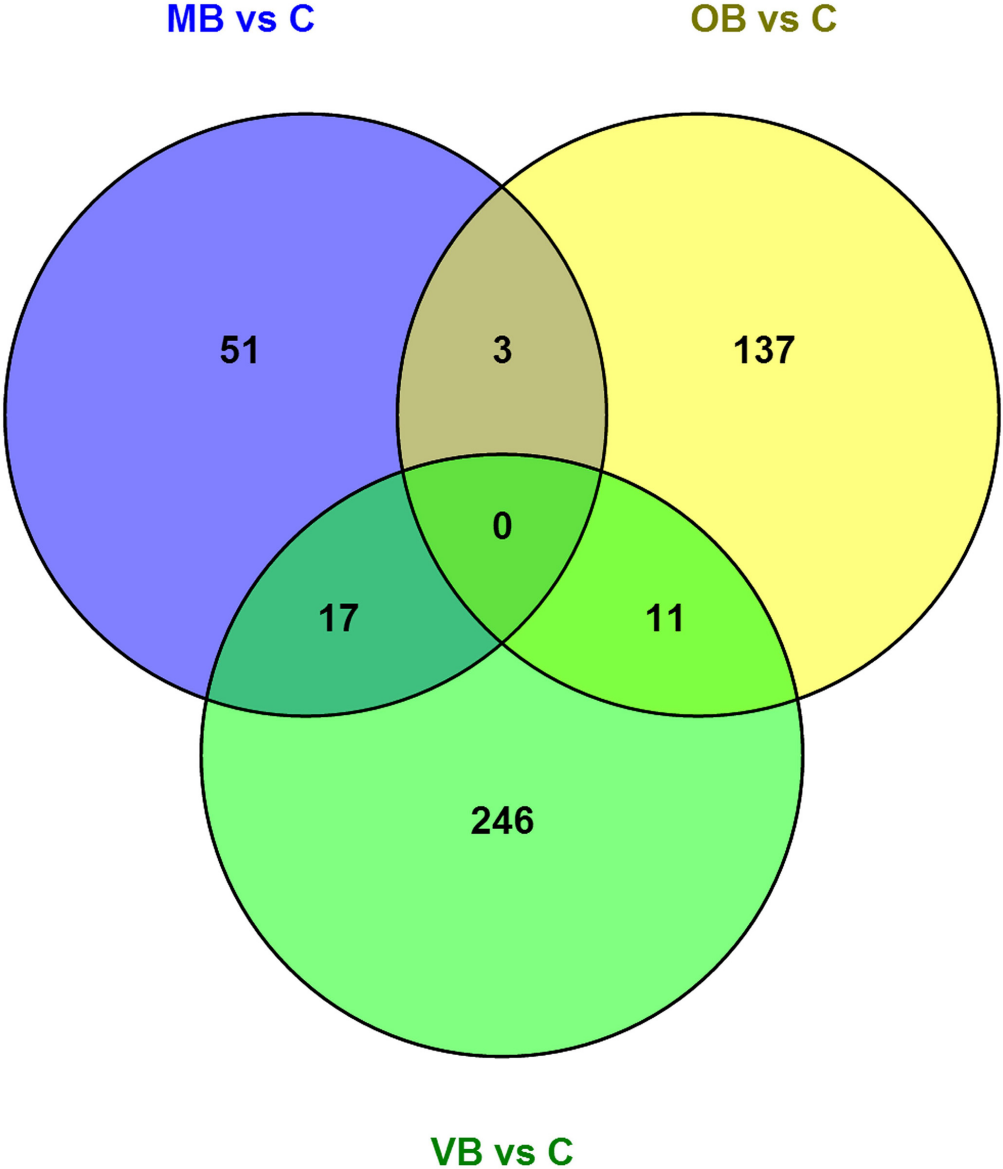
Venn diagram representation of the class comparisons MB *vs* C, OB *vs* C, and VB *vs* C. The number of the common DEGs present in the intersections are shown on the figure. Note that, the intersection “MB *vs* C” ∩ “OB *vs* C” ∩ “VB *vs* C” has no common DEGs. The gene lists of the intersections are as follows: “MB *vs* C” ∩ “OB *vs* C”: *LTF*, *OLFM4*, *CEACAM8*; “MB *vs* C” ∩ “VB *vs* C”: *HBG1*, *TMEM66*, *LGALS2*, *SEC24D*, *BCL10*, *EIF1AX*, *MAP3K4*, *KRR1*, *RP2*, *ABO*, *ATF1*, *TAX1BP1*, *CD69*, *TLR4*; “OB *vs* C” ∩ “VB *vs* C”: *PRKCQ*, *TNFAIP3*, *DDX17*, *SLC6A8*, *RGS1*, *NR4A2*, *G0S2*, *OSM*. Missing counts in the gene lists occur because of recurring gene symbols and/or probe sets without assigned symbols.

The Venn diagram representation of the class comparisons MB *vs* C, OB *vs* C, and VB *vs* C is shown in Fig 3. As can be seen in Fig 3, the number of the common DEGs in the intersection of MB *vs* C and OB *vs* C and VB *vs* C is zero.

The top 20 DEGs with respect to their FC values are listed in Table 3 with their gene symbols, probe set IDs, FC and p values. Worthy of note, while the absolute maximum and minimum FC values of the top 20 DEGs of the class comparison MB *vs* C were between 2.49 and 1.52, they were between 7.37 and 2.83 for OB *vs* C, and 6.31 and 2.66 for VB *vs* C. Another particularly important finding was the appearance of *Epiregulin* (*EREG*) in the top 20 DEGs list since it was a key finding of Xavier et al and was also among the “top genes differentially expressed between BD cases and controls” in their study (Table 3) [8].

**Table 3:** The top 20 most differentially expressed genes in the class comparison analysis.^a^

Increased^b^					Decreased^c^				
Gene Symbol		Probe Set ID	FC	p	Gene Symbol		Probe Set ID	FC	P
**MB *vs* C**^d^									
**1**	*PMAIP1*	204286_s_at	1.95	0.01768	**1**	*LTF*	202018_s_at	-2.49	0.01236
**2**	*RP2*	205191_at	1.93	0.03448	**2**	*CEACAM8*	206676_at	-2.16	0.02634
**3**	*CTSS*	202901_x_at	1.89	0.04829	**3**	*OLFM4*	212768_s_at	-1.67	0.01988
**4**	*LGALS2*	208450_at	1.85	0.01606	**4**	*KIAA0907*	230028_at	-1.65	0.01763
**5**	*BCL10*	1557257_at	1.78	0.02045	**5**	*YPEL3*	232077_s_at	-1.63	0.01782
**6**	*SEC24D*	202375_at	1.77	0.01648	**6**	*STK24*	215188_at	-1.62	0.01275
**7**	*NUCKS1*	222027_at	1.77	0.02384	**7**	*CD36*	209555_s_at	-1.55	0.00281
**8**	*CD69*	209795_at	1.70	0.04193	**8**	*AZU1*	214575_s_at	-1.55	0.02322
**9**	*ITCH*	235057_at	1.69	0.01707	**9**	*VASH1*	1556423_at	-1.53	0.00848
**10**	*MYLIP*	228097_at	1.68	0.00678	**10**	*ATG16L2*	229389_at	-1.52	0.02743
**OB *vs* C**^e^									
**1**	*MMP8*	231688_at	6.09	0.00004	**1**	*CTCF*	214349_at	-7.37	0.00037
**2**	*LTF*	202018_s_at	5.23	0.00066	**2**	*IL8*	211506_s_at	-6.16	0.02745
**3**	*OLFM4*	212768_s_at	4.86	0.00013	**3**	*TMEM107*	224496_s_at	-4.50	0.00113
**4**	*CEACAM8*	206676_at	4.84	0.00156	**4**	*RGS1*	202988_s_at	-3.90	0.01328
**5**	*CA1*	205950_s_at	3.53	0.01229	**5**	*G0S2*	213524_s_at	-3.19	0.01272
**6**	*CRISP3*	207802_at	3.41	0.00140	**6**	*CCL4*	204103_at	-3.06	0.01276
**7**	*AHSP*	219672_at	2.99	0.02106	**7**	*RHOH*	236293_at	-2.97	0.00020
**8**	*DEFA4*	207269_at	2.98	0.01174	**8**	*SRSF3*	232392_at	-2.91	0.00058
**9**	*LCN2*	212531_at	2.96	0.00019	**9**	*TNFAIP3*	202643_s_at	-2.84	0.00319
**10**	*CHI3L1*	209395_at	2.88	0.00006	**10**	*CDC42SE2*	229026_at	-2.83	0.00800
**VB *vs* C**^e^									
**1**	*HBM*	240336_at	3.35	0.01314	**1**	*EREG*	205767_at	-6.31	0.03990
**2**	*AMFR*	202203_s_at	3.29	0.02310	**2**	*NR4A2*	216248_s_at	-5.87	0.00605
**3**	*HBD*	206834_at	3.15	0.02727	**3**	*RGS1*	202988_s_at	-5.31	0.00868
**4**	*SLC25A37*	228527_s_at	2.97	0.00279	**4**	*CD69*	209795_at	-4.04	0.00076
**5**	*ALAS2*	211560_s_at	2.84	0.04500	**5**	*G0S2*	213524_s_at	-3.81	0.01691
**6**	*SNCA*	204467_s_at	2.79	0.00489	**6**	*S100B*	209686_at	-3.66	0.00573
**7**	*SRSF6*	206108_s_at	2.72	0.02661	**7**	*MAFF*	36711_at	-3.57	0.00709
**8**	*PDZK1IP1*	219630_at	2.67	0.03309	**8**	*SERPINB2*	204614_at	-3.51	0.01877
**9**	*EPB42*	210746_s_at	2.67	0.04710	**9**	*EID1*	211698_at	-3.35	0.00688
**10**	*KRT1*	205900_at	2.66	0.00349	**10**	*GALNACT2*	218871_x_at	-3.24	0.00252

^a^ Only the results of the comparisons MB *vs* C, OB *vs* C, and VB *vs* C are presented.

^b^ Increased expression in the first class (e.g., MB) with respect to the second class (e.g., C).

^c^ Decreased expression in the first class (e.g., MB) with respect to the second class (e.g., C).

^d^ For the class comparison MB *vs* C, p and FC were ≤0.05 and ≥1.5 respectively.

^e^ For the class comparisons OB *vs* C and VB *vs* C, p and FC were ≤0.05 and ≥2.0 respectively.

### Cluster analysis

**Fig 4:**
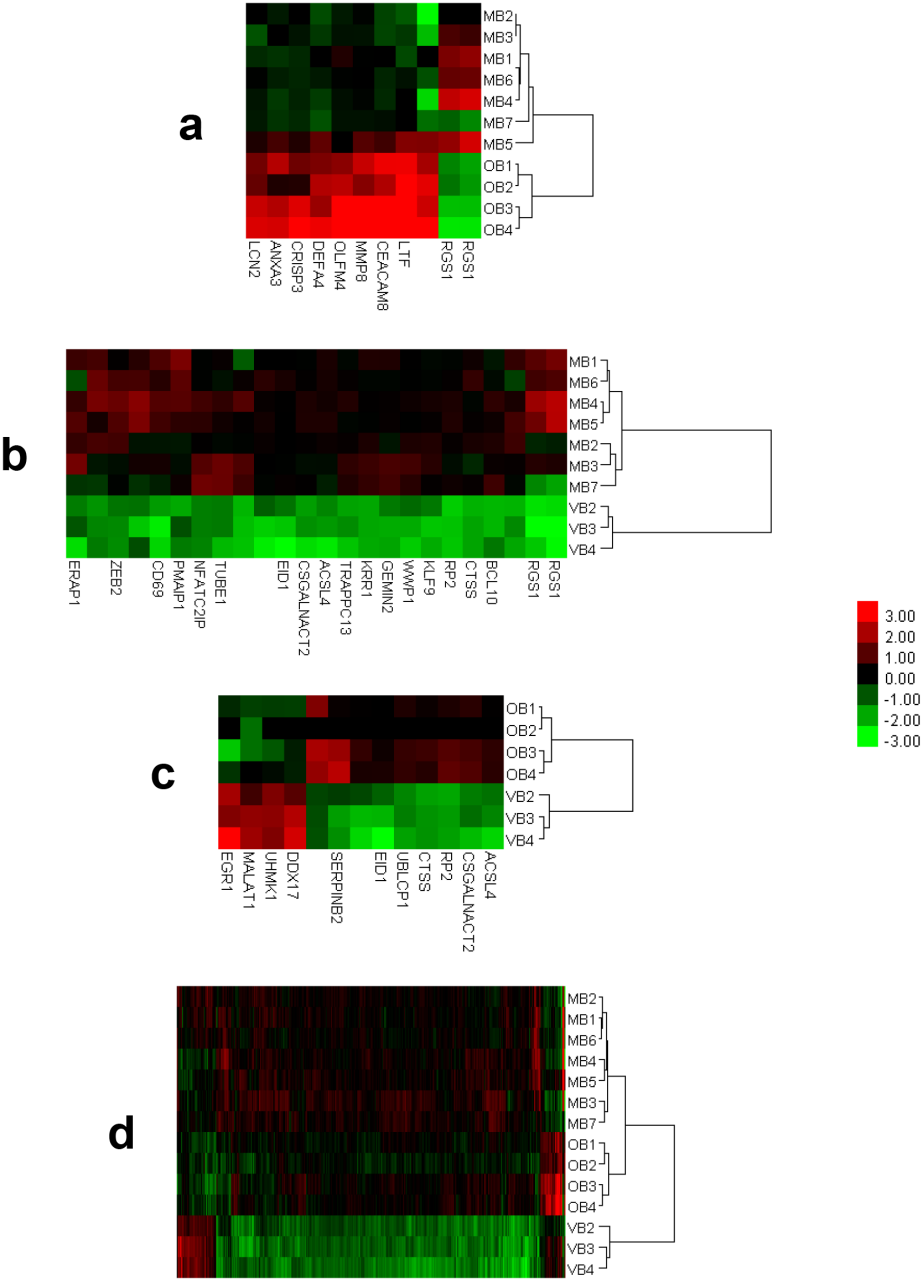
Dendrogram and heatmap representations of the results of the cluster analysis “MB & OB” (a), “MB & VB” (b), “OB & VB” (c), and “MB & OB & VB” (d). For every case, hierarchical clustering using Euclidean metric and average linkage was employed and both patients and genes were clustered. For ease of demonstration, only the dendrograms for clustering of the patients are shown in the heatmaps. As the figure shows, the algorithm and the gene sets implemented successfully clustered distinct expressions of Behçet’s disease. The gene sets used for clustering were constituted from the DEGs identified during the corresponding class comparisons (i.e., MB *vs* OB, MB *vs* VB, OB *vs* VB, and MB *vs* OB *vs* VB).

The results of the cluster analysis are displayed in Fig 4. The number of the DEGs present in each of the gene sets employed during clustering of MB & OB, MB & VB, OB & VB, and MB & OB & VB groups were 11, 24, 13, and 373 respectively. As Fig 4 shows, the clustering algorithm and the gene sets employed for clustering successfully clustered distinct expressions of BD, namely, MB, OB, and VB. The gene lists of the DEGs used for clustering are presented in the S4 File.

### Gene Ontology term enrichment analysis

Summary of the key findings of the GO term enrichment analysis is presented in Table 4. For each class comparison (i.e., MB *vs* C, OB *vs* C, and VB *vs* C), the top 10 GO categories with respect to their enrichment scores are listed in the table. The comprehensive results of the GO term enrichment analysis are given in the S5–S7 Files. Remarkably, several GO categories with relevance to interleukin-8 (IL-8) production (for MB *vs* C) and immune response to microorganisms (for OB *vs* C) were enriched. Additionally, biological processes of cytokine production regulation, leukocyte activation, and immune response gained substantial prominence.

**Table 4:** Summary of key findings of the Gene Ontology term enrichment analysis.^a^^,^ ^b^^,^ ^c^

GO Category	GO ID	G	O	E	R	Raw p	Adj p
**MB *vs* C**^d^							
Positive regulation of myeloid leukocyte cytokine production	0061081	8	3	0.02	143.25	9.14e-07	0.0003
Regulation of interleukin-8 biosynthetic process	0045414	12	3	0.03	95.50	3.57e-06	0.0003
Interleukin-8 biosynthetic process	0042228	13	3	0.03	88.15	4.63e-06	0.0003
Regulation of cytokine production involved in immune response	0002718	39	4	0.10	39.18	3.05e-06	0.0003
Regulation of interleukin-8 production	0032677	40	4	0.10	38.20	3.38e-06	0.0003
*Innate immune response (GO*:*0045087); Defense response (GO*:*0006952); Regulation of cytokine production (GO*:*0001817); Immune response (GO*:*0006955); Immune system process (GO*:*0002376)*.							
**OB *vs* C**^e^							
Response to bacterium	0009617	319	10	2.11	4.74	4.77e-05	0.0047
Leukocyte activation	0045321	537	15	3.55	4.22	2.18e-06	0.0005
Response to other organism	0051707	547	15	3.62	4.14	2.73e-06	0.0005
Response to biotic stimulus	0009607	574	15	3.80	3.95	4.92e-06	0.0008
Immune response	0006955	1006	25	6.66	3.76	4.49e-09	2.23e-06
*Immune system process (GO*:*0002376); Defense response (GO*:*0006952); Positive regulation of metabolic process (GO*:*0009893); Positive regulation of cellular process (GO*:*0048522); Positive regulation of macromolecule metabolic process (GO*:*0010604)*.							
**VB *vs* C**^e^							
Protein modification process	0036211	2409	57	29.60	1.93	2.39e-07	0.0001
Cellular protein modification process	0006464	2409	57	29.60	1.93	2.39e-07	0.0001
Macromolecule modification	0043412	2501	57	30.74	1.85	8.57e-07	0.0002
Cellular protein metabolic process	0044267	3150	69	38.71	1.78	1.31e-07	0.0001
Protein metabolic process	0019538	3730	75	45.84	1.64	9.45e-07	0.0002
*Single-organism metabolic process (GO*:*0044710); Cellular metabolic process (GO*:*0044237); Organic substance metabolic process (GO*:*0071704); Metabolic process (GO*:*0008152); Macromolecule metabolic process (GO*:*0043170)*.							

Adj, adjusted (by the multiple test adjustment); E, the expected number of genes in the category; G, the number of reference genes in the category; GO, gene ontology; O, the number of genes in the gene set and also in the category; R, ratio of enrichment.

^a^ For each class comparison, the top 10 GO categories with respect to their enrichment scores are presented.

^b^ For the 6^th^ to 10^th^ GO categories, only the GO category names and the GO IDs are listed.

^c^ The GO term enrichment analysis specifically focused on the sub-root of biological process.

^d^ For the class comparison MB *vs* C, p and FC were ≤0.05 and ≥1.5 respectively.

^e^ For the class comparisons OB *vs* C and VB *vs* C, p and FC were ≤0.05 and ≥2.0 respectively.

### Matching of the differentially expressed genes’ loci with the loci identified in the genome-wide association and the genome-wide linkage studies of Behçet’s disease

The matches between the loci identified in the GWA and the GWL studies of BD and the loci of the DEGs documented in the present study are listed in Table 5. A total of 25 non-HLA loci are included and although 5 of the GWAS/GWLS loci (i.e., 1p31.3, 2q32.2-q32.3, 10q24, 16q12, 22q11.22) had no corresponding DEGs, the remaining 20 loci hosted a total of 51 matching DEGs (range 1–6). Interestingly and importantly, congruous with the findings of Kirino et al, *ERAP1* (5q15), *KLRC4* (12p13.2-p12.3), and *CCR2* (3p21) were among the matching DEGs (Table 5) [21].

**Table 5:** Matches between the loci identified in the genome-wide association and the genome-wide linkage studies of Behçet’s disease and the loci of the differentially expressed genes documented in the present study.^a^

GWAS/GWLS Loci	Differentially Expressed Genes with Overlapping Loci	Remarks
**1p31.3**^b^	-	-
**1p36**^c^	*SRSF10*, *EIF4G3*	VB *vs* C
**1q31-q32**^b^	*NUCKS1*, *CHI3L1*, *RGS1*, *G0S2*, *ELK4*, *ZNF281*	MB *vs* C, OB *vs* C, VB *vs* C
**2q32.2-q32.3**^b^	-	-
**3p12**^d^	*NFKBIZ*	OB *vs* C
**3p21**^b^	*LTF*, *PBRM1*, *CAMP*, *CCR2*	MB *vs* C, OB *vs* C, VB *vs* C
**4p15**^c^	*DCAF16*	VB *vs* C
**5q12**^c^	*TRAPPC13*, *SREK1IP1*, *CD180*, *CENPK*	VB *vs* C
**5q15**^b^	*ELL2*, *TTC37*, *ERAP1*	MB *vs* C, VB *vs* C
**5q23**^c^	*HBEGF*	VB *vs* C
**6q16**^c^	*MANEA*, *UFL1*	MB *vs* C, VB *vs* C
**6q25-26**^c^	*TULP4*, *SYTL3*	Significant locus^c^^,^ ^d^, OB *vs* C
**6q25.1**^d^	*PPIL4*	VB *vs* C
**7p21**^c^	*ARL4A*, *UMAD1*	MB *vs* C, VB *vs* C
**10q24**^c^	-	-
**12p12-13**^c^	*CD69*, *CCND2*, *PTPRO*, *SLC2A3*	Significant locus^c^^,^ ^d^, MB *vs* C, OB *vs* C, VB *vs* C
**12p12.1**^d^	*BCAT1*, *ETNK1*	OB *vs* C, VB *vs* C
**12p13.2-p12.3**^b^	*CLEC12A*, *CLEC12B*, *KLRC4*	MB *vs* C, OB *vs* C, VB *vs* C
**12q13**^c^	*RPAP3*, *ATF1*, *PCBP2*, *SLC16A7*, *KRT1*	MB *vs* C, OB *vs* C, VB *vs* C
**16q12**^c^	-	-
**16q21-23**^c^	*CTCF*, *NFAT5*, *AMFR*	OB *vs* C, VB *vs* C
**17p13**^c^	*TMEM107*, *PER1*	OB *vs* C, VB *vs* C
**20q12-13**^c^	*SLMO2*, *SRSF6*	VB *vs* C
**22q11.22**^d^	-	-
**Xq26-28**^c^	*SLC6A8*	OB *vs* C, VB *vs* C

GWAS, genome-wide association study; GWLS, genome-wide linkage study.

^a^ Only the non-HLA loci are listed.

^b^ Kirino et al, 2013 [21].

^c^ Karasneh et al, 2005 [19].

^d^ Meguro et al, 2009 [20].

## Discussion

For BD, the appropriateness of the use of “Behçet’s syndrome” instead of “Behçet’s disease” has been previously suggested [22, 23]. In support of this recommendation, it was proposed by Lehner et al that distinct immunological abnormalities were underlying distinct classification groups of BD [6]. To date, the epidemiological basis of and the genetic linkage in BD has been pretty well studied. Nevertheless, in the era of “multi-omics”, omics data, particularly genome-wide transcription data is scarce in BD. In the present study, by borrowing the expression profiling data of Xavier et al and implementing a “data mining” approach, it was demonstrated that: (1) BD patients demonstrate distinct expression profiles in distinct disease subsets; (2) Different disease associated pathways seem to be functional in different disease expressions of BD; and (3) Four functionally related gene groups, namely, negative regulators of inflammation, neutrophil granule proteins, antigen processing and presentation proteins, and regulators of immune response are differentially expressed in BD patients with respect to healthy controls [8].

The immunological aberrations underlying the clinical manifestations of BD is comprehensively studied and reviewed elsewhere [24, 25]. As previously stated, BD is a chronic relapsing multisystemic inflammatory disorder with a strong genetic background. HLA-B51 allele, a MHC class I gene, is shown to be a causal risk determinant for BD. Infectious agents including some common bacteria (e.g., streptococci) and viruses (e.g., HSV) seem to have a role in triggering the immune responses in BD patients [24, 25]. While neutrophil leukocyte hyperactivity is a well-documented and central theme in BD, γδ T lymphocytes, which have functions in both innate and adaptive immune responses show distinct expansion patterns during periods of increased BD activity [24, 25]. With regard to adaptive immune responses, increased levels of IL-12 and a consequent Th1 response is characteristic of BD and human heat shock proteins seem to be targets for adaptive immune responses as a result of their homologies with certain streptococcal and/or mycobacterial peptides [24, 25]. Although not implicated in disease pathogenesis, various autoantibodies (e.g., antibodies against α-enolase, α-tropomyosin, kinectin) are also detected in certain subsets of BD patients [24]. Finally, endothelial cell injury is another important heading in the immunopathogenesis of BD which probably is responsible for the well-known prothrombotic state of this disorder [24].

Before going deep into the discussion of the findings, it is necessary to touch on the aberrant behaviour of the sample VB1. This 40 year old female patient with a BD diagnosis of 5 years duration was reported to demonstrate “oral and genital aphtosis, pseudofolliculitis, erythema nodosum, and large vein thrombosis” as her “major clinical symptoms” and therefore was subgrouped as VB initially [8]. Unexpectedly, during initial verification cluster analysis, VB1 consistently clustered with MB (Fig 2). As a potential explanation for this finding, we propose the possible association of a hereditary and/or acquired hypercoagulable state as the primal cause of vascular thrombosis in this mucocutaneous BD patient. It is a well-documented fact that various hypercoagulable states associated with increased risk of thrombosis contribute to the intrinsic prothrombotic state of BD [26]. Therefore, in the case of VB1 a search for thrombophilia seems relevant and may prove worthwhile.

The results of the class comparison analysis revealed strong evidences of an immunogenetic heterogeneity in different disease expressions of BD. First of all, pooling and collectively comparing the BD patients with controls (i.e., B *vs* C) seemed to have a pronounced attenuating effect on the number of DEGs (Table 2). Conversely, the class comparisons of BD subsets both with C and among themselves yielded substantially increased number of DEGs (Table 2). When taken together, these two findings point to a reciprocal gene expression pattern in different subsets of BD patients which was exactly the case for some of the DEGs (e.g., *CD69*, *LTF*, *CEACAM8*, *OLFM4*) as documented in Tables 3 and 6. This pattern of opposite immunological findings is a well-known concept in BD (e.g., conflicting reports of increased, normal or decreased neutrophil functions) [27].

**Table 6:** Potentially significant differentially expressed genes with immune/inflammatory functions.^a^

Symbol	Function^b^	BD Subset(s)^c^	Remarks^d^
**Negative regulators of inflammation**			
***CD69***	(-) regulation of inflammation, leukocyte activation marker	VB (↓), MB (↑)	Locus: 12p12-13, CLEC
***CLEC12A***	(-) regulator of granulocyte and monocyte function	MB (↑)	Locus: 12p13.2, CLEC
***CLEC12B***	Inhibitory receptor of myeloid cells	OB (↑)	Locus: 12p13.2, CLEC
***TNFAIP3***	Potent inhibitor of NF-κB signaling pathway	VB (↓), OB (↓)	Loss-of-function mutations resemble BD
**Neutrophil granule proteins**			
***AZU1***	Antimicrobial, chemotactic, inflammatory	MB (↓)	
***CAMP***	Antimicrobial, chemotaxis, inflammatory	OB (↑)	Locus: 3p21.3
***DEFA4***	Antimicrobial, corticostatic	OB (↑)	
***LTF***	Antimicrobial, anti-inflammatory	OB (↑), MB (↓)	Locus: 3p21.31
***MMP8***	Inflammatory, collagen degrading	OB (↑)	
***OLFM4***	(-) regulator of neutrophil bactericidal activity	OB (↑), MB (↓)	
**Antigen processing and presentation**			
***CTSS***	Antigenic protein degradation, elastase	MB (↑)	
***ERAP1***	Antigenic protein degradation	VB (↓)	Locus: 5q15
**Regulators of immune response**			
***BCL10***	Activation of NF-κB signaling pathway, B and T cell receptors signaling pathways	VB (↓), MB (↑)	
***CCL4***	Chemokine, inflammatory	OB (↓)	
***CCR2***	Chemokine receptor, inflammatory	VB (↑)	Locus: 3p21.31
***CD36***	Receptor for cell adhesion and oxLDL, inflammatory	MB (↓)	
***CD180***	Pathogen receptor, TLR, inflammatory	VB (↑)	Locus: 5q12
***CEACAM8***	Receptor for cell adhesion	OB (↑), MB (↓)	
***CXCL8***	Chemokine, inflammatory (neutrophilic)	OB (↓)	
***EREG***	Inflammation, wound healing	VB (↓)	Among the top DEGs in the study of Xavier et al^e^
***ITCH***	Regulation of immune response	MB (↑)	Works with *TNFAIP3*, mutations cause autoimmunity
***KLRC4***	NK cell MHC recognition receptor	VB (↓)	Locus: 12p13.2-p12.3, CLEC
***LGALS2***	Lymphotoxin binding lectin	VB (↑), MB (↑)	Lectin
***NFAT5***	Activated T cell transcription factor, inflammatory	OB (↓)	Locus: 16q22.1
***NFKBIZ***	Regulation of immune response	OB (↓)	Locus: 3p12-q12

CLEC, C-type lectin; DEGs, differentially expressed genes.

^a^ During preparation of the table, the top 20 most differentially expressed genes (Table 3) and the differentially expressed genes featuring genomic loci matching with the loci identified in the genome-wide association and the genome-wide linkage studies of Behçet’s disease (Table 5) were reviewed.

^b^ Not a comprehensive list of functions is presented.

^c^ Behçet’s disease subsets with differential expression of the mentioned gene and the direction of change (in brackets) are listed.

^d^ Important genomic loci, prominent gene groups, and significant disease associations are given in the “Remarks” column.

^e^ Xavier et al, 2013 [8].

When taken together with the relatively limited number of DEGs found in the class comparison MB *vs* C, the modest FC values observed may implicate that, among BD subsets, MB has the least difference in gene expression patterns compared to controls (Tables 2 and 3). Consistently, BD patients with only the mucocutaneous manifestations of the disease are widely recognized as having the mildest presentation of the disease.

Another evidence of immunogenetic heterogeneity came from the Venn diagram analysis of the class comparisons. As shown in Fig 3, the number of the common DEGs in the binary intersections of the class comparisons were markedly limited (i.e., 3, 17, and 11), while the same number in the intersection “MB *vs* C” ∩ “OB *vs* C” ∩ “VB *vs* C” was zero. This was a striking finding demonstrating that not even a single DEG was shared among the class comparisons of BD subsets with C; again indicating an important degree of pathogenetic heterogeneity among BD subsets.

An additional evidence was provided by the results of the cluster analysis. Using a gene set of 373 DEGs (ANOVA, p≤0.001), the clustering experiment effectively clustered BD patients into three clusters which exactly matched the manifestation based clusters of BD patients (Fig 4). The chosen level of significance, the number of the DEGs employed, and the success of clustering offered supporting evidence for an immunogenetic heterogeneity in distinct disease expressions of BD.

The results of the GO term enrichment analysis were also supportive. It appeared that GO categories with relevance to IL-8 production (MB *vs* C) and immune response to microorganisms (OB *vs* C) were prominently and differentially enriched (Table 4). IL-8, which is also known as “neutrophil chemotactic factor”, plays a central role in neutrophil functions by inducing both chemotaxis and phagocytosis [28]. The mucocutaneous lesions (e.g., Pathergy reaction, pustular folliculitis) which are the hallmarks of BD, characteristically demonstrate significant neutrophilic infiltrates [29–31]. Additionally, IL-8 has previously shown to be increased in BD patients [32, 33]. Thus, enrichment of the GO terms relevant to IL-8 production was consistent with the literature.

Infectious agents, especially streptococci have long been pointed to as etiologic/triggering factors in BD [2, 34–37]. With regard to an infectious etiology, an indirect mechanism involving heat shock proteins (HSP) and a cross-reactivity/molecular mimicry etiology have been postulated among many others [38]. An important potential link between ocular involvement in BD and the streptococci may be the streptococcus-related *bes-1* gene derived peptides, which are shown to demonstrate a high level of homology with human retinal protein Brn-3b and HSP60 [39, 40]. As such, it was noteworthy to find the immune response to microorganisms related GO categories enriched for the class comparison OB *vs* C.

Although many different definitions exist, “disease” can be defined as “a definite pathological process having a characteristic set of symptoms and signs” while “syndrome” as “the aggregate of symptoms and signs associated with any morbid process” [41, 42]. While currently no molecular level differentation of the terms “disease” and “syndrome” is possible, as authors we strongly recommend the preference of “Behçet’s syndrome” (BS) instead of “Behçet’s disease”, based on the findings of the present study.

As is known, the distinguishing features of BS are its recurrent mucocutaneous lesions. Additionally, whether the International Study Group (ISG) or the International Team for the Revision of the International Criteria for Behçet's Disease (ITR-ICBD) criteria are used, the diagnosis / classification of BS requires the presence of certain characteristic mucocutaneous lesions (4 out of 5 and 4 out of 6 criteria are mucocutaneous in origin in the ISG and the ITR-ICBD criteria sets respectively) [10, 43]. Furthermore, while oral aphthous ulcer is a “must” in the ISG criteria, genital aphthous ulcers have more diagnostic value than other criteria in the ITR-ICBD set [10, 43]. As summarized in Table 1, all BS patients in the current study were also sharing mucocutaneous manifestations irrespective of their BS subsets (i.e., MB, OB or VB). We believe that this resemblance of BS patients is of importance, not only from a diagnostic / classification perspective but also from an etiopathogenetic point of view. When the Venn diagram representation of the class comparisons MB *vs* C, OB *vs* C, and VB *vs* C is taken into consideration (Fig 3), the close resemblance of BS patients with respect to their mucocutaneous manifestations is inexplicable with no common DEGs in the intersection “MB *vs* C” ∩ “OB *vs* C” ∩ “VB *vs* C”. Therefore, it is crucial to elucidate the molecular pathogenetic mechanisms responsible for the common mucocutaneous manifestations observed in different BS subsets displaying disparate sets of DEGs and distinct disease pathways.

When the FC values and the genomic loci of the DEGs were specifically taken into consideration, negative regulators of inflammation (*CD69*, *CLEC12A*, *CLEC12B*, *TNFAIP3*), neutrophil granule proteins (*LTF*, *OLFM4*, *AZU1*, *MMP8*, *DEFA4*, *CAMP*), antigen processing and presentation proteins (*CTSS*, *ERAP1*), and regulators of immune response (*LGALS2*, *BCL10*, *ITCH*, *CEACAM8*, *CD36*, *IL8*, *CCL4*, *EREG*, *NFKBIZ*, *CCR2*, *CD180*, *KLRC4*, *NFAT5*) were found to be differentially expressed in BS patients with respect to controls (Tables 3, 5 and 6). If the fundamental pathogenetic mechanism of BS is defined as a pro-inflammatory, innate-immune system derived response sustained by acquired immune system responses against environmental and/or self-antigens, it is motivating to note the congruences between the definition and the above-listed gene groups [34].

**Fig 5:**
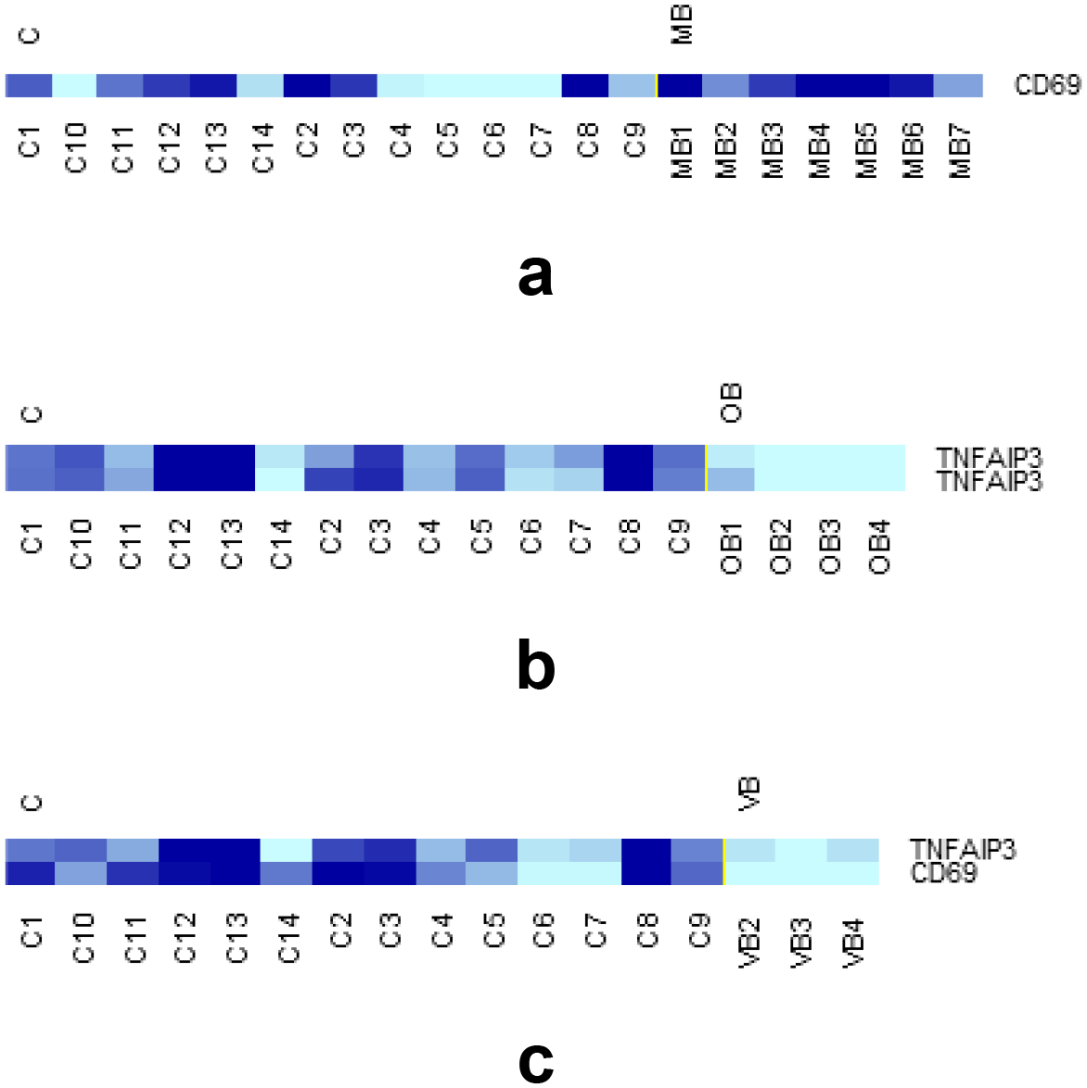
Clustered heatmap representations of *TNFAIP3* and *CD69* expressions in class comparisons MB *vs* C (a), OB *vs* C (b), and VB *vs* C (c). *CD69*, cluster of differentiation 69; *TNFAIP3*, tumor necrosis factor, α-induced protein 3.

Albeit deserving a comprehensive and rigorous discussion which is beyond the limits and the main theme of this manuscript, the negative regulators of inflammation merit special consideration. *CD69*, *CLEC12A*, *CLEC12B*, and *TNFAIP3* are among the well-documented inhibitors of inflammation/immune response [44–48]. Recently, Zhou et al reported a novel autoinflammatory disorder (haploinsufficiency of A20 [HA20]) occurring as a result of loss-of-function mutations in the *TNFAIP3* gene [48]. It is remarkable to note that, phenotypically, HA20 closely resembles BS with the occurrence of recurrent oral ulcers, dermal abscesses, positive pathergy response, and retinal vasculitis [48]. In the present study, we documented that, at the transcriptomic level *TNFAIP3* was downregulated in the OB and VB subsets of BS patients (Table 7 and Fig 5). *CD69*, yet another gene responsible for the regulation of inflammation was also shown to be significantly underexpressed in the VB subset (Table 7 and Fig 5). *CLEC12A* followed a similar trend with underexpression in the VB subset when compared to both the MB and OB subsets. Recently, this finding was communicated as a preliminary result to support a hypothesis about the role *CLEC12A* plays in BS [49]. Taken together, these findings indicate that, in patients with severe forms of BS, negative regulators of inflammation are underexpressed compared to controls and/or patients with milder presentations of the syndrome. Such a downregulation of inhibitors of inflammation may well be responsible for the pro-inflammatory milieu which is characteristic of BS [34]. Conversely stating, in patients with BS, increased expression of negative regulators of inflammation may serve a protective role against severe forms of the syndrome.

**Table 7:** Expression patterns of TNFAIP3 and CD69 in BS subsets and controls.

Gene Symbol	Probe Set ID	Geom mean of intensities in control group	Geom mean of intensities in BS subsets	Fold Change^a^	Parametric p-value
**Class comparison MB *vs* C**^b^					
Differential expression of *TNFAIP3* was not observed					
*CD69*	209795_at	1279.38	2169.5	1.70	0.0419338
**Class comparison OB *vs* C**^c^					
*TNFAIP3*	202643_s_at	1219.4	428.69	0.35	0.0031889
*TNFAIP3*	202644_s_at	2742.78	1164.59	0.42	0.0052164
Differential expression of *CD69* was not observed					
**Class comparison VB *vs* C**^c^					
*TNFAIP3*	202644_s_at	2742.78	1259.57	0.46	0.0209217
*CD69*	209795_at	1279.38	316.75	0.25	0.0007591

BS, Behçet’s syndrome; *CD69*, cluster of differentiation 69; *TNFAIP3*, tumor necrosis factor, α-induced protein 3.

^a^ According to the expression in BS subset with respect to control group.

^b^ For the class comparison MB *vs* C, p and FC were ≤0.05 and ≥1.5 respectively.

^c^ For the class comparisons OB *vs* C and VB *vs* C, p and FC were ≤0.05 and ≥2.0 respectively.

This study may be a good example for data mining. By borrowing the gene expression profiling data of Xavier et al, keeping a different perspective, and implementing a novel strategy, our group was able to document significant molecular level discrepancies among BS patient subsets [8, 12]. In their original paper Xavier et al combined gene expression profiling with association studies to elucidate BS’s genetic background [8]. Finally, they were able to document that *EREG*-*AREG* and *NRG1* (members of the epidermal growth factor family), seemed to modulate BS susceptibility through both by direct effects and by gene-gene interactions [8]. Their study strongly emphasized the value of combining “omics” strategies (integration of “omics” data) to reveal the genetic background of complex diseases. Nevertheless, in their study Xavier et al collectively analysed BS patients and did not implement any manifestation based clinical grouping [8]. We believe that their approach has at least two important justifications; the first one being to keep a time honored approach implemented in BS research. With the exception of a limited number of studies which mainly investigate ocular BS in isolation, the current literature harbors research which analyze BS patients in a collective manner regardless of their clinical picture. The second one is due to the “omics” integration design of their study. The genome-wide association studies and the validation patient dataset used by Xavier et al belonged to collective / inclusive sets of BS patients [8]. As such, for their integration study, Xavier et al used the gene expression profiling data of a collective set of BS patients [8].

As is the case with any scientific research, the present study also is not without limitations. The well-known fact about the marked regional variations in the expression of BS necessitates the interpretation of our findings in the context that they belong to a Portuguese population [5, 8]. Because of ethical considerations, continuing therapeutic regimens of BS patients had not been interrupted with potential implications in their expression profiles [8]. Also, in addition to a limited number of patients in each BS subset, BS patients with gastrointestinal, musculoskeletal, or central nervous system involvement were not represented [8]. Therefore, new expression profiling studies enrolling large numbers of treatment-naive BS patients with a wide spectrum of manifestations are clearly needed. The authors also think that in BS, eQTL (expression quantitative trait loci) analysis which simultaneously explore genome-wide expression and genetic variation data will prove worthwhile [50, 51]. The need for validation of the findings in an independent BS cohort may be another issue regarding limitations. Regrettably, this validation was not possible due to lack of an independent BS cohort’s peripheral blood mononuclear cells expression profiling data [9, 52]. Furthermore, the marked regional variations observed in the expression of BS complicate the matter and necessitate that, such a validation data should belong to a Portuguese population.

## Conclusions

BS patients display distinct expression profiles and different disease associated pathways in distinct subsets of the disorder. IL-8 production and immune response to microorganisms categories are differentially enriched among BS patient subsets. Future research, especially the studies focusing on a molecular level should take into account the immunogenetic heterogeneity of BS subsets. Based on these discrepancies, the designation as “Behçet’s syndrome” should be encouraged.

Negative regulators of inflammation, neutrophil granule proteins, antigen processing and presentation proteins, and regulators of immune response appear to be instrumental in BS immunopathogenesis. Some of these genes/gene products may prove to be specific, effective, and low toxicity therapeutic targets in BS.

## Supporting Information

S1 File Experiment descriptor file. (XLSX)

S2 File Gene lists used for *initial verification* clustering. (XLSX)

S3 File Gene lists of the differentially expressed genes identified during class comparison analysis. (XLSX)

S4 File Gene lists used for clustering. (XLSX)

S5 File GO term enrichment analysis results for MB vs C. (HTML)

S6 File GO term enrichment analysis results for OB vs C. (HTML)

S7 File GO term enrichment analysis results for VB vs C. (HTML)
