# Supplementary material for: Behçet's: A Disease or a Syndrome? Answer from an Expression Profiling Study
Source: PLoS One. 2016 Feb 18;11(2):e0149052. doi: 10.1371/journal.pone.0149052 (PMC4758705; doi:10.1371/journal.pone.0149052)
Supplement: S5 File — (HTML) [file pone.0149052.s005.html]

Anchored HTML File of EIDs


|  |  |
| --- | --- |
|  | WEB-based GEne SeT AnaLysis Toolkit |
|  |  |
|  | ***Translating gene lists into biological insights...*** |
|  |  |

---

  

| **Database:biological process      &nbspName:innate immune response      &nbspID:GO:0045087** | | | | | | |
| --- | --- | --- | --- | --- | --- | --- |
| C=496; O=9; E=1.30; R=6.93; rawP=3.79e-06; adjP=0.0003 | | | | | | |
| Index | UserID | Value | Gene Symbol | Gene Name | EntrezGene | Ensembl |
| 1 | 1558233\_s\_at | NA | ATF1 | activating transcription factor 1 | 466 | ENSG00000123268 |
| 2 | 202901\_x\_at | NA | CTSS | cathepsin S | 1520 | ENSG00000163131 |
| 3 | 202270\_at | NA | GBP1 | guanylate binding protein 1, interferon-inducible | 2633 | ENSG00000117228 |
| 4 | 235057\_at | NA | ITCH | itchy E3 ubiquitin protein ligase | 83737 | ENSG00000078747 |
| 5 | 213786\_at | NA | TAX1BP1 | Tax1 (human T-cell leukemia virus type I) binding protein 1 | 8887 | ENSG00000106052 |
| 6 | 213506\_at | NA | F2RL1 | coagulation factor II (thrombin) receptor-like 1 | 2150 | ENSG00000164251 |
| 7 | 232068\_s\_at | NA | TLR4 | toll-like receptor 4 | 7099 | ENSG00000136869 |
| 8 | 215118\_s\_at | NA | IGHG1 | immunoglobulin heavy constant gamma 1 (G1m marker) | 3500 | NULL |
| 9 | 1557257\_at | NA | BCL10 | B-cell CLL/lymphoma 10 | 8915 | ENSG00000142867 |

  
  

| **Database:biological process      &nbspName:defense response      &nbspID:GO:0006952** | | | | | | |
| --- | --- | --- | --- | --- | --- | --- |
| C=1026; O=13; E=2.69; R=4.84; rawP=9.41e-07; adjP=0.0003 | | | | | | |
| Index | UserID | Value | Gene Symbol | Gene Name | EntrezGene | Ensembl |
| 1 | 1558233\_s\_at | NA | ATF1 | activating transcription factor 1 | 466 | ENSG00000123268 |
| 2 | 202901\_x\_at | NA | CTSS | cathepsin S | 1520 | ENSG00000163131 |
| 3 | 221841\_s\_at | NA | KLF4 | Kruppel-like factor 4 (gut) | 9314 | ENSG00000136826 |
| 4 | 202270\_at | NA | GBP1 | guanylate binding protein 1, interferon-inducible | 2633 | ENSG00000117228 |
| 5 | 235057\_at | NA | ITCH | itchy E3 ubiquitin protein ligase | 83737 | ENSG00000078747 |
| 6 | 213786\_at | NA | TAX1BP1 | Tax1 (human T-cell leukemia virus type I) binding protein 1 | 8887 | ENSG00000106052 |
| 7 | 214575\_s\_at | NA | AZU1 | azurocidin 1 | 566 | ENSG00000172232 |
| 8 | 213506\_at | NA | F2RL1 | coagulation factor II (thrombin) receptor-like 1 | 2150 | ENSG00000164251 |
| 9 | 209555\_s\_at | NA | CD36 | CD36 molecule (thrombospondin receptor) | 948 | ENSG00000135218 |
| 10 | 232068\_s\_at | NA | TLR4 | toll-like receptor 4 | 7099 | ENSG00000136869 |
| 11 | 202018\_s\_at | NA | LTF | lactotransferrin | 4057 | ENSG00000012223 |
| 12 | 215118\_s\_at | NA | IGHG1 | immunoglobulin heavy constant gamma 1 (G1m marker) | 3500 | NULL |
| 13 | 1557257\_at | NA | BCL10 | B-cell CLL/lymphoma 10 | 8915 | ENSG00000142867 |

  
  

| **Database:biological process      &nbspName:regulation of cytokine production involved in immune response      &nbspID:GO:0002718** | | | | | | |
| --- | --- | --- | --- | --- | --- | --- |
| C=39; O=4; E=0.10; R=39.18; rawP=3.05e-06; adjP=0.0003 | | | | | | |
| Index | UserID | Value | Gene Symbol | Gene Name | EntrezGene | Ensembl |
| 1 | 213506\_at | NA | F2RL1 | coagulation factor II (thrombin) receptor-like 1 | 2150 | ENSG00000164251 |
| 2 | 209555\_s\_at | NA | CD36 | CD36 molecule (thrombospondin receptor) | 948 | ENSG00000135218 |
| 3 | 232068\_s\_at | NA | TLR4 | toll-like receptor 4 | 7099 | ENSG00000136869 |
| 4 | 1557257\_at | NA | BCL10 | B-cell CLL/lymphoma 10 | 8915 | ENSG00000142867 |

  
  

| **Database:biological process      &nbspName:regulation of cytokine production      &nbspID:GO:0001817** | | | | | | |
| --- | --- | --- | --- | --- | --- | --- |
| C=364; O=8; E=0.95; R=8.40; rawP=3.53e-06; adjP=0.0003 | | | | | | |
| Index | UserID | Value | Gene Symbol | Gene Name | EntrezGene | Ensembl |
| 1 | 221841\_s\_at | NA | KLF4 | Kruppel-like factor 4 (gut) | 9314 | ENSG00000136826 |
| 2 | 235057\_at | NA | ITCH | itchy E3 ubiquitin protein ligase | 83737 | ENSG00000078747 |
| 3 | 213786\_at | NA | TAX1BP1 | Tax1 (human T-cell leukemia virus type I) binding protein 1 | 8887 | ENSG00000106052 |
| 4 | 214575\_s\_at | NA | AZU1 | azurocidin 1 | 566 | ENSG00000172232 |
| 5 | 232068\_s\_at | NA | TLR4 | toll-like receptor 4 | 7099 | ENSG00000136869 |
| 6 | 209555\_s\_at | NA | CD36 | CD36 molecule (thrombospondin receptor) | 948 | ENSG00000135218 |
| 7 | 213506\_at | NA | F2RL1 | coagulation factor II (thrombin) receptor-like 1 | 2150 | ENSG00000164251 |
| 8 | 1557257\_at | NA | BCL10 | B-cell CLL/lymphoma 10 | 8915 | ENSG00000142867 |

  
  

| **Database:biological process      &nbspName:immune response      &nbspID:GO:0006955** | | | | | | |
| --- | --- | --- | --- | --- | --- | --- |
| C=1006; O=12; E=2.63; R=4.56; rawP=5.31e-06; adjP=0.0003 | | | | | | |
| Index | UserID | Value | Gene Symbol | Gene Name | EntrezGene | Ensembl |
| 1 | 1558233\_s\_at | NA | ATF1 | activating transcription factor 1 | 466 | ENSG00000123268 |
| 2 | 202901\_x\_at | NA | CTSS | cathepsin S | 1520 | ENSG00000163131 |
| 3 | 202270\_at | NA | GBP1 | guanylate binding protein 1, interferon-inducible | 2633 | ENSG00000117228 |
| 4 | 235057\_at | NA | ITCH | itchy E3 ubiquitin protein ligase | 83737 | ENSG00000078747 |
| 5 | 213786\_at | NA | TAX1BP1 | Tax1 (human T-cell leukemia virus type I) binding protein 1 | 8887 | ENSG00000106052 |
| 6 | 206676\_at | NA | CEACAM8 | carcinoembryonic antigen-related cell adhesion molecule 8 | 1088 | ENSG00000124469 |
| 7 | 213506\_at | NA | F2RL1 | coagulation factor II (thrombin) receptor-like 1 | 2150 | ENSG00000164251 |
| 8 | 209555\_s\_at | NA | CD36 | CD36 molecule (thrombospondin receptor) | 948 | ENSG00000135218 |
| 9 | 232068\_s\_at | NA | TLR4 | toll-like receptor 4 | 7099 | ENSG00000136869 |
| 10 | 202018\_s\_at | NA | LTF | lactotransferrin | 4057 | ENSG00000012223 |
| 11 | 215118\_s\_at | NA | IGHG1 | immunoglobulin heavy constant gamma 1 (G1m marker) | 3500 | NULL |
| 12 | 1557257\_at | NA | BCL10 | B-cell CLL/lymphoma 10 | 8915 | ENSG00000142867 |

  
  

| **Database:biological process      &nbspName:regulation of interleukin-8 biosynthetic process      &nbspID:GO:0045414** | | | | | | |
| --- | --- | --- | --- | --- | --- | --- |
| C=12; O=3; E=0.03; R=95.50; rawP=3.57e-06; adjP=0.0003 | | | | | | |
| Index | UserID | Value | Gene Symbol | Gene Name | EntrezGene | Ensembl |
| 1 | 232068\_s\_at | NA | TLR4 | toll-like receptor 4 | 7099 | ENSG00000136869 |
| 2 | 221841\_s\_at | NA | KLF4 | Kruppel-like factor 4 (gut) | 9314 | ENSG00000136826 |
| 3 | 1557257\_at | NA | BCL10 | B-cell CLL/lymphoma 10 | 8915 | ENSG00000142867 |

  
  

| **Database:biological process      &nbspName:positive regulation of myeloid leukocyte cytokine production involved in immune response      &nbspID:GO:0061081** | | | | | | |
| --- | --- | --- | --- | --- | --- | --- |
| C=8; O=3; E=0.02; R=143.25; rawP=9.14e-07; adjP=0.0003 | | | | | | |
| Index | UserID | Value | Gene Symbol | Gene Name | EntrezGene | Ensembl |
| 1 | 209555\_s\_at | NA | CD36 | CD36 molecule (thrombospondin receptor) | 948 | ENSG00000135218 |
| 2 | 232068\_s\_at | NA | TLR4 | toll-like receptor 4 | 7099 | ENSG00000136869 |
| 3 | 1557257\_at | NA | BCL10 | B-cell CLL/lymphoma 10 | 8915 | ENSG00000142867 |

  
  

| **Database:biological process      &nbspName:regulation of interleukin-8 production      &nbspID:GO:0032677** | | | | | | |
| --- | --- | --- | --- | --- | --- | --- |
| C=40; O=4; E=0.10; R=38.20; rawP=3.38e-06; adjP=0.0003 | | | | | | |
| Index | UserID | Value | Gene Symbol | Gene Name | EntrezGene | Ensembl |
| 1 | 213506\_at | NA | F2RL1 | coagulation factor II (thrombin) receptor-like 1 | 2150 | ENSG00000164251 |
| 2 | 232068\_s\_at | NA | TLR4 | toll-like receptor 4 | 7099 | ENSG00000136869 |
| 3 | 221841\_s\_at | NA | KLF4 | Kruppel-like factor 4 (gut) | 9314 | ENSG00000136826 |
| 4 | 1557257\_at | NA | BCL10 | B-cell CLL/lymphoma 10 | 8915 | ENSG00000142867 |

  
  

| **Database:biological process      &nbspName:immune system process      &nbspID:GO:0002376** | | | | | | |
| --- | --- | --- | --- | --- | --- | --- |
| C=1708; O=16; E=4.47; R=3.58; rawP=1.89e-06; adjP=0.0003 | | | | | | |
| Index | UserID | Value | Gene Symbol | Gene Name | EntrezGene | Ensembl |
| 1 | 202375\_at | NA | SEC24D | SEC24 family, member D (S. cerevisiae) | 9871 | ENSG00000150961 |
| 2 | 235057\_at | NA | ITCH | itchy E3 ubiquitin protein ligase | 83737 | ENSG00000078747 |
| 3 | 213786\_at | NA | TAX1BP1 | Tax1 (human T-cell leukemia virus type I) binding protein 1 | 8887 | ENSG00000106052 |
| 4 | 206676\_at | NA | CEACAM8 | carcinoembryonic antigen-related cell adhesion molecule 8 | 1088 | ENSG00000124469 |
| 5 | 214575\_s\_at | NA | AZU1 | azurocidin 1 | 566 | ENSG00000172232 |
| 6 | 209555\_s\_at | NA | CD36 | CD36 molecule (thrombospondin receptor) | 948 | ENSG00000135218 |
| 7 | 202018\_s\_at | NA | LTF | lactotransferrin | 4057 | ENSG00000012223 |
| 8 | 215118\_s\_at | NA | IGHG1 | immunoglobulin heavy constant gamma 1 (G1m marker) | 3500 | NULL |
| 9 | 1557257\_at | NA | BCL10 | B-cell CLL/lymphoma 10 | 8915 | ENSG00000142867 |
| 10 | 1558233\_s\_at | NA | ATF1 | activating transcription factor 1 | 466 | ENSG00000123268 |
| 11 | 202901\_x\_at | NA | CTSS | cathepsin S | 1520 | ENSG00000163131 |
| 12 | 204286\_s\_at | NA | PMAIP1 | phorbol-12-myristate-13-acetate-induced protein 1 | 5366 | ENSG00000141682 |
| 13 | 202270\_at | NA | GBP1 | guanylate binding protein 1, interferon-inducible | 2633 | ENSG00000117228 |
| 14 | 221841\_s\_at | NA | KLF4 | Kruppel-like factor 4 (gut) | 9314 | ENSG00000136826 |
| 15 | 232068\_s\_at | NA | TLR4 | toll-like receptor 4 | 7099 | ENSG00000136869 |
| 16 | 213506\_at | NA | F2RL1 | coagulation factor II (thrombin) receptor-like 1 | 2150 | ENSG00000164251 |

  
  

| **Database:biological process      &nbspName:interleukin-8 biosynthetic process      &nbspID:GO:0042228** | | | | | | |
| --- | --- | --- | --- | --- | --- | --- |
| C=13; O=3; E=0.03; R=88.15; rawP=4.63e-06; adjP=0.0003 | | | | | | |
| Index | UserID | Value | Gene Symbol | Gene Name | EntrezGene | Ensembl |
| 1 | 232068\_s\_at | NA | TLR4 | toll-like receptor 4 | 7099 | ENSG00000136869 |
| 2 | 221841\_s\_at | NA | KLF4 | Kruppel-like factor 4 (gut) | 9314 | ENSG00000136826 |
| 3 | 1557257\_at | NA | BCL10 | B-cell CLL/lymphoma 10 | 8915 | ENSG00000142867 |

  
  

| **Database:molecular function      &nbspName:pattern recognition receptor activity      &nbspID:GO:0008329** | | | | | | |
| --- | --- | --- | --- | --- | --- | --- |
| C=15; O=2; E=0.04; R=56.55; rawP=0.0006; adjP=0.0462 | | | | | | |
| Index | UserID | Value | Gene Symbol | Gene Name | EntrezGene | Ensembl |
| 1 | 209555\_s\_at | NA | CD36 | CD36 molecule (thrombospondin receptor) | 948 | ENSG00000135218 |
| 2 | 232068\_s\_at | NA | TLR4 | toll-like receptor 4 | 7099 | ENSG00000136869 |

  
  

| **Database:molecular function      &nbspName:kinase regulator activity      &nbspID:GO:0019207** | | | | | | |
| --- | --- | --- | --- | --- | --- | --- |
| C=127; O=3; E=0.30; R=10.02; rawP=0.0033; adjP=0.1270 | | | | | | |
| Index | UserID | Value | Gene Symbol | Gene Name | EntrezGene | Ensembl |
| 1 | 221841\_s\_at | NA | KLF4 | Kruppel-like factor 4 (gut) | 9314 | ENSG00000136826 |
| 2 | 1557257\_at | NA | BCL10 | B-cell CLL/lymphoma 10 | 8915 | ENSG00000142867 |
| 3 | 202241\_at | NA | TRIB1 | tribbles homolog 1 (Drosophila) | 10221 | ENSG00000173334 |

  
  

| **Database:molecular function      &nbspName:carbohydrate derivative binding      &nbspID:GO:0097367** | | | | | | |
| --- | --- | --- | --- | --- | --- | --- |
| C=187; O=3; E=0.44; R=6.80; rawP=0.0095; adjP=0.2438 | | | | | | |
| Index | UserID | Value | Gene Symbol | Gene Name | EntrezGene | Ensembl |
| 1 | 208450\_at | NA | LGALS2 | lectin, galactoside-binding, soluble, 2 | 3957 | ENSG00000100079 |
| 2 | 214575\_s\_at | NA | AZU1 | azurocidin 1 | 566 | ENSG00000172232 |
| 3 | 202018\_s\_at | NA | LTF | lactotransferrin | 4057 | ENSG00000012223 |

  
  

| **Database:molecular function      &nbspName:serine hydrolase activity      &nbspID:GO:0017171** | | | | | | |
| --- | --- | --- | --- | --- | --- | --- |
| C=159; O=2; E=0.37; R=5.33; rawP=0.0538; adjP=0.2959 | | | | | | |
| Index | UserID | Value | Gene Symbol | Gene Name | EntrezGene | Ensembl |
| 1 | 214575\_s\_at | NA | AZU1 | azurocidin 1 | 566 | ENSG00000172232 |
| 2 | 202018\_s\_at | NA | LTF | lactotransferrin | 4057 | ENSG00000012223 |

  
  

| **Database:molecular function      &nbspName:heparin binding      &nbspID:GO:0008201** | | | | | | |
| --- | --- | --- | --- | --- | --- | --- |
| C=129; O=2; E=0.30; R=6.58; rawP=0.0370; adjP=0.2959 | | | | | | |
| Index | UserID | Value | Gene Symbol | Gene Name | EntrezGene | Ensembl |
| 1 | 214575\_s\_at | NA | AZU1 | azurocidin 1 | 566 | ENSG00000172232 |
| 2 | 202018\_s\_at | NA | LTF | lactotransferrin | 4057 | ENSG00000012223 |

  
  

| **Database:molecular function      &nbspName:carbohydrate binding      &nbspID:GO:0030246** | | | | | | |
| --- | --- | --- | --- | --- | --- | --- |
| C=239; O=3; E=0.56; R=5.32; rawP=0.0184; adjP=0.2959 | | | | | | |
| Index | UserID | Value | Gene Symbol | Gene Name | EntrezGene | Ensembl |
| 1 | 208450\_at | NA | LGALS2 | lectin, galactoside-binding, soluble, 2 | 3957 | ENSG00000100079 |
| 2 | 209795\_at | NA | CD69 | CD69 molecule | 969 | ENSG00000110848 |
| 3 | 1569401\_at | NA | CLEC12A | C-type lectin domain family 12, member A | 160364 | ENSG00000172322 |

  
  

| **Database:molecular function      &nbspName:serine-type endopeptidase activity      &nbspID:GO:0004252** | | | | | | |
| --- | --- | --- | --- | --- | --- | --- |
| C=136; O=2; E=0.32; R=6.24; rawP=0.0407; adjP=0.2959 | | | | | | |
| Index | UserID | Value | Gene Symbol | Gene Name | EntrezGene | Ensembl |
| 1 | 214575\_s\_at | NA | AZU1 | azurocidin 1 | 566 | ENSG00000172232 |
| 2 | 202018\_s\_at | NA | LTF | lactotransferrin | 4057 | ENSG00000012223 |

  
  

| **Database:molecular function      &nbspName:endopeptidase activity      &nbspID:GO:0004175** | | | | | | |
| --- | --- | --- | --- | --- | --- | --- |
| C=347; O=3; E=0.82; R=3.67; rawP=0.0477; adjP=0.2959 | | | | | | |
| Index | UserID | Value | Gene Symbol | Gene Name | EntrezGene | Ensembl |
| 1 | 202901\_x\_at | NA | CTSS | cathepsin S | 1520 | ENSG00000163131 |
| 2 | 214575\_s\_at | NA | AZU1 | azurocidin 1 | 566 | ENSG00000172232 |
| 3 | 202018\_s\_at | NA | LTF | lactotransferrin | 4057 | ENSG00000012223 |

  
  

| **Database:molecular function      &nbspName:ubiquitin protein ligase binding      &nbspID:GO:0031625** | | | | | | |
| --- | --- | --- | --- | --- | --- | --- |
| C=147; O=2; E=0.35; R=5.77; rawP=0.0468; adjP=0.2959 | | | | | | |
| Index | UserID | Value | Gene Symbol | Gene Name | EntrezGene | Ensembl |
| 1 | 1557257\_at | NA | BCL10 | B-cell CLL/lymphoma 10 | 8915 | ENSG00000142867 |
| 2 | 202241\_at | NA | TRIB1 | tribbles homolog 1 (Drosophila) | 10221 | ENSG00000173334 |

  
  

| **Database:molecular function      &nbspName:guanyl ribonucleotide binding      &nbspID:GO:0032561** | | | | | | |
| --- | --- | --- | --- | --- | --- | --- |
| C=361; O=3; E=0.85; R=3.52; rawP=0.0525; adjP=0.2959 | | | | | | |
| Index | UserID | Value | Gene Symbol | Gene Name | EntrezGene | Ensembl |
| 1 | 205191\_at | NA | RP2 | retinitis pigmentosa 2 (X-linked recessive) | 6102 | ENSG00000102218 |
| 2 | 205020\_s\_at | NA | ARL4A | ADP-ribosylation factor-like 4A | 10124 | ENSG00000122644 |
| 3 | 202270\_at | NA | GBP1 | guanylate binding protein 1, interferon-inducible | 2633 | ENSG00000117228 |

  
  

| **Database:cellular component      &nbspName:lipopolysaccharide receptor complex      &nbspID:GO:0046696** | | | | | | |
| --- | --- | --- | --- | --- | --- | --- |
| C=5; O=2; E=0.01; R=150.94; rawP=6.82e-05; adjP=0.0050 | | | | | | |
| Index | UserID | Value | Gene Symbol | Gene Name | EntrezGene | Ensembl |
| 1 | 232068\_s\_at | NA | TLR4 | toll-like receptor 4 | 7099 | ENSG00000136869 |
| 2 | 1557257\_at | NA | BCL10 | B-cell CLL/lymphoma 10 | 8915 | ENSG00000142867 |

  
  

| **Database:cellular component      &nbspName:phagocytic vesicle      &nbspID:GO:0045335** | | | | | | |
| --- | --- | --- | --- | --- | --- | --- |
| C=49; O=2; E=0.13; R=15.40; rawP=0.0074; adjP=0.2628 | | | | | | |
| Index | UserID | Value | Gene Symbol | Gene Name | EntrezGene | Ensembl |
| 1 | 209555\_s\_at | NA | CD36 | CD36 molecule (thrombospondin receptor) | 948 | ENSG00000135218 |
| 2 | 202018\_s\_at | NA | LTF | lactotransferrin | 4057 | ENSG00000012223 |

  
  

| **Database:cellular component      &nbspName:external side of plasma membrane      &nbspID:GO:0009897** | | | | | | |
| --- | --- | --- | --- | --- | --- | --- |
| C=193; O=3; E=0.51; R=5.87; rawP=0.0144; adjP=0.2628 | | | | | | |
| Index | UserID | Value | Gene Symbol | Gene Name | EntrezGene | Ensembl |
| 1 | 209555\_s\_at | NA | CD36 | CD36 molecule (thrombospondin receptor) | 948 | ENSG00000135218 |
| 2 | 232068\_s\_at | NA | TLR4 | toll-like receptor 4 | 7099 | ENSG00000136869 |
| 3 | 209795\_at | NA | CD69 | CD69 molecule | 969 | ENSG00000110848 |

  
  

| **Database:cellular component      &nbspName:membrane raft      &nbspID:GO:0045121** | | | | | | |
| --- | --- | --- | --- | --- | --- | --- |
| C=193; O=3; E=0.51; R=5.87; rawP=0.0144; adjP=0.2628 | | | | | | |
| Index | UserID | Value | Gene Symbol | Gene Name | EntrezGene | Ensembl |
| 1 | 209555\_s\_at | NA | CD36 | CD36 molecule (thrombospondin receptor) | 948 | ENSG00000135218 |
| 2 | 232068\_s\_at | NA | TLR4 | toll-like receptor 4 | 7099 | ENSG00000136869 |
| 3 | 1557257\_at | NA | BCL10 | B-cell CLL/lymphoma 10 | 8915 | ENSG00000142867 |

  
  

| **Database:cellular component      &nbspName:cytoplasmic vesicle      &nbspID:GO:0031410** | | | | | | |
| --- | --- | --- | --- | --- | --- | --- |
| C=900; O=5; E=2.39; R=2.10; rawP=0.0877; adjP=0.2814 | | | | | | |
| Index | UserID | Value | Gene Symbol | Gene Name | EntrezGene | Ensembl |
| 1 | 214575\_s\_at | NA | AZU1 | azurocidin 1 | 566 | ENSG00000172232 |
| 2 | 209555\_s\_at | NA | CD36 | CD36 molecule (thrombospondin receptor) | 948 | ENSG00000135218 |
| 3 | 202375\_at | NA | SEC24D | SEC24 family, member D (S. cerevisiae) | 9871 | ENSG00000150961 |
| 4 | 235057\_at | NA | ITCH | itchy E3 ubiquitin protein ligase | 83737 | ENSG00000078747 |
| 5 | 202018\_s\_at | NA | LTF | lactotransferrin | 4057 | ENSG00000012223 |

  
  

| **Database:cellular component      &nbspName:lytic vacuole      &nbspID:GO:0000323** | | | | | | |
| --- | --- | --- | --- | --- | --- | --- |
| C=334; O=3; E=0.89; R=3.39; rawP=0.0582; adjP=0.2814 | | | | | | |
| Index | UserID | Value | Gene Symbol | Gene Name | EntrezGene | Ensembl |
| 1 | 202901\_x\_at | NA | CTSS | cathepsin S | 1520 | ENSG00000163131 |
| 2 | 214575\_s\_at | NA | AZU1 | azurocidin 1 | 566 | ENSG00000172232 |
| 3 | 1557257\_at | NA | BCL10 | B-cell CLL/lymphoma 10 | 8915 | ENSG00000142867 |

  
  

| **Database:cellular component      &nbspName:secretory granule      &nbspID:GO:0030141** | | | | | | |
| --- | --- | --- | --- | --- | --- | --- |
| C=243; O=3; E=0.64; R=4.66; rawP=0.0263; adjP=0.2814 | | | | | | |
| Index | UserID | Value | Gene Symbol | Gene Name | EntrezGene | Ensembl |
| 1 | 214575\_s\_at | NA | AZU1 | azurocidin 1 | 566 | ENSG00000172232 |
| 2 | 209555\_s\_at | NA | CD36 | CD36 molecule (thrombospondin receptor) | 948 | ENSG00000135218 |
| 3 | 202018\_s\_at | NA | LTF | lactotransferrin | 4057 | ENSG00000012223 |

  
  

| **Database:cellular component      &nbspName:extracellular region      &nbspID:GO:0005576** | | | | | | |
| --- | --- | --- | --- | --- | --- | --- |
| C=1914; O=9; E=5.07; R=1.77; rawP=0.0596; adjP=0.2814 | | | | | | |
| Index | UserID | Value | Gene Symbol | Gene Name | EntrezGene | Ensembl |
| 1 | 212768\_s\_at | NA | OLFM4 | olfactomedin 4 | 10562 | ENSG00000102837 |
| 2 | 216929\_x\_at | NA | ABO | ABO blood group (transferase A, alpha 1-3-N-acetylgalactosaminyltransferase; transferase B, alpha 1-3-galactosyltransferase) | 28 | NULL |
| 3 | 202901\_x\_at | NA | CTSS | cathepsin S | 1520 | ENSG00000163131 |
| 4 | 202270\_at | NA | GBP1 | guanylate binding protein 1, interferon-inducible | 2633 | ENSG00000117228 |
| 5 | 206676\_at | NA | CEACAM8 | carcinoembryonic antigen-related cell adhesion molecule 8 | 1088 | ENSG00000124469 |
| 6 | 214575\_s\_at | NA | AZU1 | azurocidin 1 | 566 | ENSG00000172232 |
| 7 | 1556423\_at | NA | VASH1 | vasohibin 1 | 22846 | ENSG00000071246 |
| 8 | 202018\_s\_at | NA | LTF | lactotransferrin | 4057 | ENSG00000012223 |
| 9 | 215118\_s\_at | NA | IGHG1 | immunoglobulin heavy constant gamma 1 (G1m marker) | 3500 | NULL |

  
  

| **Database:cellular component      &nbspName:Golgi apparatus      &nbspID:GO:0005794** | | | | | | |
| --- | --- | --- | --- | --- | --- | --- |
| C=1133; O=6; E=3.00; R=2.00; rawP=0.0762; adjP=0.2814 | | | | | | |
| Index | UserID | Value | Gene Symbol | Gene Name | EntrezGene | Ensembl |
| 1 | 216929\_x\_at | NA | ABO | ABO blood group (transferase A, alpha 1-3-N-acetylgalactosaminyltransferase; transferase B, alpha 1-3-galactosyltransferase) | 28 | NULL |
| 2 | 219003\_s\_at | NA | MANEA | mannosidase, endo-alpha | 79694 | ENSG00000172469 |
| 3 | 213506\_at | NA | F2RL1 | coagulation factor II (thrombin) receptor-like 1 | 2150 | ENSG00000164251 |
| 4 | 209555\_s\_at | NA | CD36 | CD36 molecule (thrombospondin receptor) | 948 | ENSG00000135218 |
| 5 | 202375\_at | NA | SEC24D | SEC24 family, member D (S. cerevisiae) | 9871 | ENSG00000150961 |
| 6 | 202270\_at | NA | GBP1 | guanylate binding protein 1, interferon-inducible | 2633 | ENSG00000117228 |

  
  

| **Database:cellular component      &nbspName:organelle lumen      &nbspID:GO:0043233** | | | | | | |
| --- | --- | --- | --- | --- | --- | --- |
| C=3256; O=13; E=8.63; R=1.51; rawP=0.0734; adjP=0.2814 | | | | | | |
| Index | UserID | Value | Gene Symbol | Gene Name | EntrezGene | Ensembl |
| 1 | 1558233\_s\_at | NA | ATF1 | activating transcription factor 1 | 466 | ENSG00000123268 |
| 2 | 202901\_x\_at | NA | CTSS | cathepsin S | 1520 | ENSG00000163131 |
| 3 | 226099\_at | NA | ELL2 | elongation factor, RNA polymerase II, 2 | 22936 | ENSG00000118985 |
| 4 | 205020\_s\_at | NA | ARL4A | ADP-ribosylation factor-like 4A | 10124 | ENSG00000122644 |
| 5 | 221841\_s\_at | NA | KLF4 | Kruppel-like factor 4 (gut) | 9314 | ENSG00000136826 |
| 6 | 216983\_s\_at | NA | ZNF224 | zinc finger protein 224 | 7767 | ENSG00000186019 |
| 7 | 223400\_s\_at | NA | PBRM1 | polybromo 1 | 55193 | ENSG00000163939 |
| 8 | 222402\_at | NA | POMP | proteasome maturation protein | 51371 | ENSG00000132963 |
| 9 | 221596\_s\_at | NA | RBM48 | RNA binding motif protein 48 | 84060 | ENSG00000127993 |
| 10 | 202018\_s\_at | NA | LTF | lactotransferrin | 4057 | ENSG00000012223 |
| 11 | 215188\_at | NA | STK24 | serine/threonine kinase 24 | 8428 | ENSG00000102572 |
| 12 | 203203\_s\_at | NA | KRR1 | KRR1, small subunit (SSU) processome component, homolog (yeast) | 11103 | ENSG00000111615 |
| 13 | 232077\_s\_at | NA | YPEL3 | yippee-like 3 (Drosophila) | 83719 | ENSG00000090238 |

  
  
  
  


---

WebGestalt is currently developed and maintained by Jing Wang and Bing Zhang at the  Zhang Lab. Other people who have made significant contribution to the project include Dexter Duncan, Stefan Kirov, Zhiao Shi, and Jay Snoddy.  
  
**Funding credits:** NIH/NIAAA (U01 AA016662, U01 AA013512); NIH/NIDA (P01 DA015027); NIH/NIMH (P50 MH078028, P50 MH096972); NIH/NCI (U24 CA159988); NIH/NIGMS (R01 GM088822).
