# Supplementary material for: Behçet's: A Disease or a Syndrome? Answer from an Expression Profiling Study
Source: PLoS One. 2016 Feb 18;11(2):e0149052. doi: 10.1371/journal.pone.0149052 (PMC4758705; doi:10.1371/journal.pone.0149052)
Supplement: S6 File — (HTML) [file pone.0149052.s006.html]

Anchored HTML File of EIDs


|  |  |
| --- | --- |
|  | WEB-based GEne SeT AnaLysis Toolkit |
|  |  |
|  | ***Translating gene lists into biological insights...*** |
|  |  |

---

  

| **Database:biological process      &nbspName:immune system process      &nbspID:GO:0002376** | | | | | | |
| --- | --- | --- | --- | --- | --- | --- |
| C=1708; O=34; E=11.30; R=3.01; rawP=1.02e-09; adjP=1.01e-06 | | | | | | |
| Index | UserID | Value | Gene Symbol | Gene Name | EntrezGene | Ensembl |
| 1 | 222895\_s\_at | NA | BCL11B | B-cell CLL/lymphoma 11B (zinc finger protein) | 64919 | ENSG00000127152 |
| 2 | 209369\_at | NA | ANXA3 | annexin A3 | 306 | ENSG00000138772 |
| 3 | 212531\_at | NA | LCN2 | lipocalin 2 | 3934 | ENSG00000148346 |
| 4 | 208892\_s\_at | NA | DUSP6 | dual specificity phosphatase 6 | 1848 | ENSG00000139318 |
| 5 | 208891\_at | NA | DUSP6 | dual specificity phosphatase 6 | 1848 | ENSG00000139318 |
| 6 | 206804\_at | NA | CD3G | CD3g molecule, gamma (CD3-TCR complex) | 917 | ENSG00000160654 |
| 7 | 215078\_at | NA | SOD2 | superoxide dismutase 2, mitochondrial | 6648 | ENSG00000112096 |
| 8 | 232311\_at | NA | B2M | beta-2-microglobulin | 567 | ENSG00000166710 |
| 9 | 206676\_at | NA | CEACAM8 | carcinoembryonic antigen-related cell adhesion molecule 8 | 1088 | ENSG00000124469 |
| 10 | 219672\_at | NA | AHSP | alpha hemoglobin stabilizing protein | 51327 | ENSG00000169877 |
| 11 | 235567\_at | NA | RORA | RAR-related orphan receptor A | 6095 | ENSG00000069667 |
| 12 | 202018\_s\_at | NA | LTF | lactotransferrin | 4057 | ENSG00000012223 |
| 13 | 218394\_at | NA | ROGDI | rogdi homolog (Drosophila) | 79641 | ENSG00000067836 |
| 14 | 216834\_at | NA | RGS1 | regulator of G-protein signaling 1 | 5996 | ENSG00000090104 |
| 15 | 202988\_s\_at | NA | RGS1 | regulator of G-protein signaling 1 | 5996 | ENSG00000090104 |
| 16 | 201109\_s\_at | NA | THBS1 | thrombospondin 1 | 7057 | ENSG00000137801 |
| 17 | 235086\_at | NA | THBS1 | thrombospondin 1 | 7057 | ENSG00000137801 |
| 18 | 203628\_at | NA | IGF1R | insulin-like growth factor 1 receptor | 3480 | ENSG00000140443 |
| 19 | 204285\_s\_at | NA | PMAIP1 | phorbol-12-myristate-13-acetate-induced protein 1 | 5366 | ENSG00000141682 |
| 20 | 204863\_s\_at | NA | IL6ST | interleukin 6 signal transducer (gp130, oncostatin M receptor) | 3572 | ENSG00000134352 |
| 21 | 204103\_at | NA | CCL4 | chemokine (C-C motif) ligand 4 | 6351 | ENSG00000129277 |
| 22 | 219890\_at | NA | CLEC5A | C-type lectin domain family 5, member A | 23601 | ENSG00000258227 |
| 23 | 1569599\_at | NA | SAMSN1 | SAM domain, SH3 domain and nuclear localization signals 1 | 64092 | ENSG00000155307 |
| 24 | 224851\_at | NA | CDK6 | cyclin-dependent kinase 6 | 1021 | ENSG00000105810 |
| 25 | 213517\_at | NA | PCBP2 | poly(rC) binding protein 2 | 5094 | ENSG00000197111 |
| 26 | 230170\_at | NA | OSM | oncostatin M | 5008 | ENSG00000099985 |
| 27 | 207269\_at | NA | DEFA4 | defensin, alpha 4, corticostatin | 1669 | ENSG00000164821 |
| 28 | 243423\_at | NA | TNIP1 | TNFAIP3 interacting protein 1 | 10318 | ENSG00000145901 |
| 29 | 202644\_s\_at | NA | TNFAIP3 | tumor necrosis factor, alpha-induced protein 3 | 7128 | ENSG00000118503 |
| 30 | 202643\_s\_at | NA | TNFAIP3 | tumor necrosis factor, alpha-induced protein 3 | 7128 | ENSG00000118503 |
| 31 | 212240\_s\_at | NA | PIK3R1 | phosphoinositide-3-kinase, regulatory subunit 1 (alpha) | 5295 | ENSG00000145675 |
| 32 | 212249\_at | NA | PIK3R1 | phosphoinositide-3-kinase, regulatory subunit 1 (alpha) | 5295 | ENSG00000145675 |
| 33 | 207384\_at | NA | PGLYRP1 | peptidoglycan recognition protein 1 | 8993 | ENSG00000008438 |
| 34 | 214329\_x\_at | NA | TNFSF10 | tumor necrosis factor (ligand) superfamily, member 10 | 8743 | ENSG00000121858 |
| 35 | 231418\_at | NA | MS4A1 | membrane-spanning 4-domains, subfamily A, member 1 | 931 | ENSG00000156738 |
| 36 | 211919\_s\_at | NA | CXCR4 | chemokine (C-X-C motif) receptor 4 | 7852 | ENSG00000121966 |
| 37 | 209201\_x\_at | NA | CXCR4 | chemokine (C-X-C motif) receptor 4 | 7852 | ENSG00000121966 |
| 38 | 225116\_at | NA | HIPK2 | homeodomain interacting protein kinase 2 | 28996 | ENSG00000064393 |
| 39 | 211506\_s\_at | NA | IL8 | interleukin 8 | 3576 | ENSG00000169429 |
| 40 | 202859\_x\_at | NA | IL8 | interleukin 8 | 3576 | ENSG00000169429 |
| 41 | 207802\_at | NA | CRISP3 | cysteine-rich secretory protein 3 | 10321 | ENSG00000096006 |

  
  

| **Database:biological process      &nbspName:immune response      &nbspID:GO:0006955** | | | | | | |
| --- | --- | --- | --- | --- | --- | --- |
| C=1006; O=25; E=6.66; R=3.76; rawP=4.49e-09; adjP=2.23e-06 | | | | | | |
| Index | UserID | Value | Gene Symbol | Gene Name | EntrezGene | Ensembl |
| 1 | 207269\_at | NA | DEFA4 | defensin, alpha 4, corticostatin | 1669 | ENSG00000164821 |
| 2 | 209369\_at | NA | ANXA3 | annexin A3 | 306 | ENSG00000138772 |
| 3 | 202644\_s\_at | NA | TNFAIP3 | tumor necrosis factor, alpha-induced protein 3 | 7128 | ENSG00000118503 |
| 4 | 202643\_s\_at | NA | TNFAIP3 | tumor necrosis factor, alpha-induced protein 3 | 7128 | ENSG00000118503 |
| 5 | 243423\_at | NA | TNIP1 | TNFAIP3 interacting protein 1 | 10318 | ENSG00000145901 |
| 6 | 212531\_at | NA | LCN2 | lipocalin 2 | 3934 | ENSG00000148346 |
| 7 | 208892\_s\_at | NA | DUSP6 | dual specificity phosphatase 6 | 1848 | ENSG00000139318 |
| 8 | 208891\_at | NA | DUSP6 | dual specificity phosphatase 6 | 1848 | ENSG00000139318 |
| 9 | 212240\_s\_at | NA | PIK3R1 | phosphoinositide-3-kinase, regulatory subunit 1 (alpha) | 5295 | ENSG00000145675 |
| 10 | 212249\_at | NA | PIK3R1 | phosphoinositide-3-kinase, regulatory subunit 1 (alpha) | 5295 | ENSG00000145675 |
| 11 | 206804\_at | NA | CD3G | CD3g molecule, gamma (CD3-TCR complex) | 917 | ENSG00000160654 |
| 12 | 207384\_at | NA | PGLYRP1 | peptidoglycan recognition protein 1 | 8993 | ENSG00000008438 |
| 13 | 214329\_x\_at | NA | TNFSF10 | tumor necrosis factor (ligand) superfamily, member 10 | 8743 | ENSG00000121858 |
| 14 | 232311\_at | NA | B2M | beta-2-microglobulin | 567 | ENSG00000166710 |
| 15 | 206676\_at | NA | CEACAM8 | carcinoembryonic antigen-related cell adhesion molecule 8 | 1088 | ENSG00000124469 |
| 16 | 202018\_s\_at | NA | LTF | lactotransferrin | 4057 | ENSG00000012223 |
| 17 | 216834\_at | NA | RGS1 | regulator of G-protein signaling 1 | 5996 | ENSG00000090104 |
| 18 | 202988\_s\_at | NA | RGS1 | regulator of G-protein signaling 1 | 5996 | ENSG00000090104 |
| 19 | 231418\_at | NA | MS4A1 | membrane-spanning 4-domains, subfamily A, member 1 | 931 | ENSG00000156738 |
| 20 | 201109\_s\_at | NA | THBS1 | thrombospondin 1 | 7057 | ENSG00000137801 |
| 21 | 235086\_at | NA | THBS1 | thrombospondin 1 | 7057 | ENSG00000137801 |
| 22 | 203628\_at | NA | IGF1R | insulin-like growth factor 1 receptor | 3480 | ENSG00000140443 |
| 23 | 204863\_s\_at | NA | IL6ST | interleukin 6 signal transducer (gp130, oncostatin M receptor) | 3572 | ENSG00000134352 |
| 24 | 204103\_at | NA | CCL4 | chemokine (C-C motif) ligand 4 | 6351 | ENSG00000129277 |
| 25 | 211506\_s\_at | NA | IL8 | interleukin 8 | 3576 | ENSG00000169429 |
| 26 | 202859\_x\_at | NA | IL8 | interleukin 8 | 3576 | ENSG00000169429 |
| 27 | 219890\_at | NA | CLEC5A | C-type lectin domain family 5, member A | 23601 | ENSG00000258227 |
| 28 | 1569599\_at | NA | SAMSN1 | SAM domain, SH3 domain and nuclear localization signals 1 | 64092 | ENSG00000155307 |
| 29 | 213517\_at | NA | PCBP2 | poly(rC) binding protein 2 | 5094 | ENSG00000197111 |
| 30 | 207802\_at | NA | CRISP3 | cysteine-rich secretory protein 3 | 10321 | ENSG00000096006 |
| 31 | 230170\_at | NA | OSM | oncostatin M | 5008 | ENSG00000099985 |

  
  

| **Database:biological process      &nbspName:defense response      &nbspID:GO:0006952** | | | | | | |
| --- | --- | --- | --- | --- | --- | --- |
| C=1026; O=22; E=6.79; R=3.24; rawP=6.18e-07; adjP=0.0002 | | | | | | |
| Index | UserID | Value | Gene Symbol | Gene Name | EntrezGene | Ensembl |
| 1 | 207269\_at | NA | DEFA4 | defensin, alpha 4, corticostatin | 1669 | ENSG00000164821 |
| 2 | 209369\_at | NA | ANXA3 | annexin A3 | 306 | ENSG00000138772 |
| 3 | 202644\_s\_at | NA | TNFAIP3 | tumor necrosis factor, alpha-induced protein 3 | 7128 | ENSG00000118503 |
| 4 | 202643\_s\_at | NA | TNFAIP3 | tumor necrosis factor, alpha-induced protein 3 | 7128 | ENSG00000118503 |
| 5 | 243423\_at | NA | TNIP1 | TNFAIP3 interacting protein 1 | 10318 | ENSG00000145901 |
| 6 | 212531\_at | NA | LCN2 | lipocalin 2 | 3934 | ENSG00000148346 |
| 7 | 208892\_s\_at | NA | DUSP6 | dual specificity phosphatase 6 | 1848 | ENSG00000139318 |
| 8 | 208891\_at | NA | DUSP6 | dual specificity phosphatase 6 | 1848 | ENSG00000139318 |
| 9 | 207384\_at | NA | PGLYRP1 | peptidoglycan recognition protein 1 | 8993 | ENSG00000008438 |
| 10 | 232311\_at | NA | B2M | beta-2-microglobulin | 567 | ENSG00000166710 |
| 11 | 206871\_at | NA | ELANE | elastase, neutrophil expressed | 1991 | ENSG00000197561 |
| 12 | 210244\_at | NA | CAMP | cathelicidin antimicrobial peptide | 820 | ENSG00000164047 |
| 13 | 202018\_s\_at | NA | LTF | lactotransferrin | 4057 | ENSG00000012223 |
| 14 | 201109\_s\_at | NA | THBS1 | thrombospondin 1 | 7057 | ENSG00000137801 |
| 15 | 235086\_at | NA | THBS1 | thrombospondin 1 | 7057 | ENSG00000137801 |
| 16 | 211919\_s\_at | NA | CXCR4 | chemokine (C-X-C motif) receptor 4 | 7852 | ENSG00000121966 |
| 17 | 209201\_x\_at | NA | CXCR4 | chemokine (C-X-C motif) receptor 4 | 7852 | ENSG00000121966 |
| 18 | 204863\_s\_at | NA | IL6ST | interleukin 6 signal transducer (gp130, oncostatin M receptor) | 3572 | ENSG00000134352 |
| 19 | 204103\_at | NA | CCL4 | chemokine (C-C motif) ligand 4 | 6351 | ENSG00000129277 |
| 20 | 211506\_s\_at | NA | IL8 | interleukin 8 | 3576 | ENSG00000169429 |
| 21 | 202859\_x\_at | NA | IL8 | interleukin 8 | 3576 | ENSG00000169429 |
| 22 | 223217\_s\_at | NA | NFKBIZ | nuclear factor of kappa light polypeptide gene enhancer in B-cells inhibitor, zeta | 64332 | ENSG00000144802 |
| 23 | 219890\_at | NA | CLEC5A | C-type lectin domain family 5, member A | 23601 | ENSG00000258227 |
| 24 | 213517\_at | NA | PCBP2 | poly(rC) binding protein 2 | 5094 | ENSG00000197111 |
| 25 | 209396\_s\_at | NA | CHI3L1 | chitinase 3-like 1 (cartilage glycoprotein-39) | 1116 | ENSG00000133048 |
| 26 | 209395\_at | NA | CHI3L1 | chitinase 3-like 1 (cartilage glycoprotein-39) | 1116 | ENSG00000133048 |
| 27 | 207802\_at | NA | CRISP3 | cysteine-rich secretory protein 3 | 10321 | ENSG00000096006 |
| 28 | 230170\_at | NA | OSM | oncostatin M | 5008 | ENSG00000099985 |

  
  

| **Database:biological process      &nbspName:response to other organism      &nbspID:GO:0051707** | | | | | | |
| --- | --- | --- | --- | --- | --- | --- |
| C=547; O=15; E=3.62; R=4.14; rawP=2.73e-06; adjP=0.0005 | | | | | | |
| Index | UserID | Value | Gene Symbol | Gene Name | EntrezGene | Ensembl |
| 1 | 207269\_at | NA | DEFA4 | defensin, alpha 4, corticostatin | 1669 | ENSG00000164821 |
| 2 | 209369\_at | NA | ANXA3 | annexin A3 | 306 | ENSG00000138772 |
| 3 | 211919\_s\_at | NA | CXCR4 | chemokine (C-X-C motif) receptor 4 | 7852 | ENSG00000121966 |
| 4 | 209201\_x\_at | NA | CXCR4 | chemokine (C-X-C motif) receptor 4 | 7852 | ENSG00000121966 |
| 5 | 202644\_s\_at | NA | TNFAIP3 | tumor necrosis factor, alpha-induced protein 3 | 7128 | ENSG00000118503 |
| 6 | 202643\_s\_at | NA | TNFAIP3 | tumor necrosis factor, alpha-induced protein 3 | 7128 | ENSG00000118503 |
| 7 | 212531\_at | NA | LCN2 | lipocalin 2 | 3934 | ENSG00000148346 |
| 8 | 212240\_s\_at | NA | PIK3R1 | phosphoinositide-3-kinase, regulatory subunit 1 (alpha) | 5295 | ENSG00000145675 |
| 9 | 212249\_at | NA | PIK3R1 | phosphoinositide-3-kinase, regulatory subunit 1 (alpha) | 5295 | ENSG00000145675 |
| 10 | 204103\_at | NA | CCL4 | chemokine (C-C motif) ligand 4 | 6351 | ENSG00000129277 |
| 11 | 211506\_s\_at | NA | IL8 | interleukin 8 | 3576 | ENSG00000169429 |
| 12 | 202859\_x\_at | NA | IL8 | interleukin 8 | 3576 | ENSG00000169429 |
| 13 | 207384\_at | NA | PGLYRP1 | peptidoglycan recognition protein 1 | 8993 | ENSG00000008438 |
| 14 | 215078\_at | NA | SOD2 | superoxide dismutase 2, mitochondrial | 6648 | ENSG00000112096 |
| 15 | 232311\_at | NA | B2M | beta-2-microglobulin | 567 | ENSG00000166710 |
| 16 | 210244\_at | NA | CAMP | cathelicidin antimicrobial peptide | 820 | ENSG00000164047 |
| 17 | 202018\_s\_at | NA | LTF | lactotransferrin | 4057 | ENSG00000012223 |
| 18 | 224851\_at | NA | CDK6 | cyclin-dependent kinase 6 | 1021 | ENSG00000105810 |
| 19 | 213517\_at | NA | PCBP2 | poly(rC) binding protein 2 | 5094 | ENSG00000197111 |

  
  

| **Database:biological process      &nbspName:leukocyte activation      &nbspID:GO:0045321** | | | | | | |
| --- | --- | --- | --- | --- | --- | --- |
| C=537; O=15; E=3.55; R=4.22; rawP=2.18e-06; adjP=0.0005 | | | | | | |
| Index | UserID | Value | Gene Symbol | Gene Name | EntrezGene | Ensembl |
| 1 | 222895\_s\_at | NA | BCL11B | B-cell CLL/lymphoma 11B (zinc finger protein) | 64919 | ENSG00000127152 |
| 2 | 209369\_at | NA | ANXA3 | annexin A3 | 306 | ENSG00000138772 |
| 3 | 211919\_s\_at | NA | CXCR4 | chemokine (C-X-C motif) receptor 4 | 7852 | ENSG00000121966 |
| 4 | 209201\_x\_at | NA | CXCR4 | chemokine (C-X-C motif) receptor 4 | 7852 | ENSG00000121966 |
| 5 | 202644\_s\_at | NA | TNFAIP3 | tumor necrosis factor, alpha-induced protein 3 | 7128 | ENSG00000118503 |
| 6 | 202643\_s\_at | NA | TNFAIP3 | tumor necrosis factor, alpha-induced protein 3 | 7128 | ENSG00000118503 |
| 7 | 204863\_s\_at | NA | IL6ST | interleukin 6 signal transducer (gp130, oncostatin M receptor) | 3572 | ENSG00000134352 |
| 8 | 212240\_s\_at | NA | PIK3R1 | phosphoinositide-3-kinase, regulatory subunit 1 (alpha) | 5295 | ENSG00000145675 |
| 9 | 212249\_at | NA | PIK3R1 | phosphoinositide-3-kinase, regulatory subunit 1 (alpha) | 5295 | ENSG00000145675 |
| 10 | 206804\_at | NA | CD3G | CD3g molecule, gamma (CD3-TCR complex) | 917 | ENSG00000160654 |
| 11 | 211506\_s\_at | NA | IL8 | interleukin 8 | 3576 | ENSG00000169429 |
| 12 | 202859\_x\_at | NA | IL8 | interleukin 8 | 3576 | ENSG00000169429 |
| 13 | 207384\_at | NA | PGLYRP1 | peptidoglycan recognition protein 1 | 8993 | ENSG00000008438 |
| 14 | 232311\_at | NA | B2M | beta-2-microglobulin | 567 | ENSG00000166710 |
| 15 | 235567\_at | NA | RORA | RAR-related orphan receptor A | 6095 | ENSG00000069667 |
| 16 | 1569599\_at | NA | SAMSN1 | SAM domain, SH3 domain and nuclear localization signals 1 | 64092 | ENSG00000155307 |
| 17 | 224851\_at | NA | CDK6 | cyclin-dependent kinase 6 | 1021 | ENSG00000105810 |
| 18 | 231418\_at | NA | MS4A1 | membrane-spanning 4-domains, subfamily A, member 1 | 931 | ENSG00000156738 |
| 19 | 201109\_s\_at | NA | THBS1 | thrombospondin 1 | 7057 | ENSG00000137801 |
| 20 | 235086\_at | NA | THBS1 | thrombospondin 1 | 7057 | ENSG00000137801 |

  
  

| **Database:biological process      &nbspName:response to biotic stimulus      &nbspID:GO:0009607** | | | | | | |
| --- | --- | --- | --- | --- | --- | --- |
| C=574; O=15; E=3.80; R=3.95; rawP=4.92e-06; adjP=0.0008 | | | | | | |
| Index | UserID | Value | Gene Symbol | Gene Name | EntrezGene | Ensembl |
| 1 | 207269\_at | NA | DEFA4 | defensin, alpha 4, corticostatin | 1669 | ENSG00000164821 |
| 2 | 209369\_at | NA | ANXA3 | annexin A3 | 306 | ENSG00000138772 |
| 3 | 211919\_s\_at | NA | CXCR4 | chemokine (C-X-C motif) receptor 4 | 7852 | ENSG00000121966 |
| 4 | 209201\_x\_at | NA | CXCR4 | chemokine (C-X-C motif) receptor 4 | 7852 | ENSG00000121966 |
| 5 | 202644\_s\_at | NA | TNFAIP3 | tumor necrosis factor, alpha-induced protein 3 | 7128 | ENSG00000118503 |
| 6 | 202643\_s\_at | NA | TNFAIP3 | tumor necrosis factor, alpha-induced protein 3 | 7128 | ENSG00000118503 |
| 7 | 212531\_at | NA | LCN2 | lipocalin 2 | 3934 | ENSG00000148346 |
| 8 | 212240\_s\_at | NA | PIK3R1 | phosphoinositide-3-kinase, regulatory subunit 1 (alpha) | 5295 | ENSG00000145675 |
| 9 | 212249\_at | NA | PIK3R1 | phosphoinositide-3-kinase, regulatory subunit 1 (alpha) | 5295 | ENSG00000145675 |
| 10 | 204103\_at | NA | CCL4 | chemokine (C-C motif) ligand 4 | 6351 | ENSG00000129277 |
| 11 | 211506\_s\_at | NA | IL8 | interleukin 8 | 3576 | ENSG00000169429 |
| 12 | 202859\_x\_at | NA | IL8 | interleukin 8 | 3576 | ENSG00000169429 |
| 13 | 207384\_at | NA | PGLYRP1 | peptidoglycan recognition protein 1 | 8993 | ENSG00000008438 |
| 14 | 215078\_at | NA | SOD2 | superoxide dismutase 2, mitochondrial | 6648 | ENSG00000112096 |
| 15 | 232311\_at | NA | B2M | beta-2-microglobulin | 567 | ENSG00000166710 |
| 16 | 210244\_at | NA | CAMP | cathelicidin antimicrobial peptide | 820 | ENSG00000164047 |
| 17 | 202018\_s\_at | NA | LTF | lactotransferrin | 4057 | ENSG00000012223 |
| 18 | 224851\_at | NA | CDK6 | cyclin-dependent kinase 6 | 1021 | ENSG00000105810 |
| 19 | 213517\_at | NA | PCBP2 | poly(rC) binding protein 2 | 5094 | ENSG00000197111 |

  
  

| **Database:biological process      &nbspName:positive regulation of metabolic process      &nbspID:GO:0009893** | | | | | | |
| --- | --- | --- | --- | --- | --- | --- |
| C=1927; O=29; E=12.75; R=2.27; rawP=1.02e-05; adjP=0.0014 | | | | | | |
| Index | UserID | Value | Gene Symbol | Gene Name | EntrezGene | Ensembl |
| 1 | 222895\_s\_at | NA | BCL11B | B-cell CLL/lymphoma 11B (zinc finger protein) | 64919 | ENSG00000127152 |
| 2 | 209369\_at | NA | ANXA3 | annexin A3 | 306 | ENSG00000138772 |
| 3 | 202644\_s\_at | NA | TNFAIP3 | tumor necrosis factor, alpha-induced protein 3 | 7128 | ENSG00000118503 |
| 4 | 202643\_s\_at | NA | TNFAIP3 | tumor necrosis factor, alpha-induced protein 3 | 7128 | ENSG00000118503 |
| 5 | 243423\_at | NA | TNIP1 | TNFAIP3 interacting protein 1 | 10318 | ENSG00000145901 |
| 6 | 212531\_at | NA | LCN2 | lipocalin 2 | 3934 | ENSG00000148346 |
| 7 | 212240\_s\_at | NA | PIK3R1 | phosphoinositide-3-kinase, regulatory subunit 1 (alpha) | 5295 | ENSG00000145675 |
| 8 | 212249\_at | NA | PIK3R1 | phosphoinositide-3-kinase, regulatory subunit 1 (alpha) | 5295 | ENSG00000145675 |
| 9 | 209840\_s\_at | NA | LRRN3 | leucine rich repeat neuronal 3 | 54674 | ENSG00000173114 |
| 10 | 215078\_at | NA | SOD2 | superoxide dismutase 2, mitochondrial | 6648 | ENSG00000112096 |
| 11 | 206871\_at | NA | ELANE | elastase, neutrophil expressed | 1991 | ENSG00000197561 |
| 12 | 229228\_at | NA | CREB5 | cAMP responsive element binding protein 5 | 9586 | ENSG00000146592 |
| 13 | 235567\_at | NA | RORA | RAR-related orphan receptor A | 6095 | ENSG00000069667 |
| 14 | 243296\_at | NA | NAMPT | nicotinamide phosphoribosyltransferase | 10135 | ENSG00000105835 |
| 15 | 201109\_s\_at | NA | THBS1 | thrombospondin 1 | 7057 | ENSG00000137801 |
| 16 | 235086\_at | NA | THBS1 | thrombospondin 1 | 7057 | ENSG00000137801 |
| 17 | 213998\_s\_at | NA | DDX17 | DEAD (Asp-Glu-Ala-Asp) box helicase 17 | 10521 | ENSG00000100201 |
| 18 | 208719\_s\_at | NA | DDX17 | DEAD (Asp-Glu-Ala-Asp) box helicase 17 | 10521 | ENSG00000100201 |
| 19 | 208151\_x\_at | NA | DDX17 | DEAD (Asp-Glu-Ala-Asp) box helicase 17 | 10521 | ENSG00000100201 |
| 20 | 210461\_s\_at | NA | ABLIM1 | actin binding LIM protein 1 | 3983 | ENSG00000099204 |
| 21 | 211919\_s\_at | NA | CXCR4 | chemokine (C-X-C motif) receptor 4 | 7852 | ENSG00000121966 |
| 22 | 209201\_x\_at | NA | CXCR4 | chemokine (C-X-C motif) receptor 4 | 7852 | ENSG00000121966 |
| 23 | 219221\_at | NA | ZBTB38 | zinc finger and BTB domain containing 38 | 253461 | ENSG00000177311 |
| 24 | 203628\_at | NA | IGF1R | insulin-like growth factor 1 receptor | 3480 | ENSG00000140443 |
| 25 | 204285\_s\_at | NA | PMAIP1 | phorbol-12-myristate-13-acetate-induced protein 1 | 5366 | ENSG00000141682 |
| 26 | 219179\_at | NA | DACT1 | dapper, antagonist of beta-catenin, homolog 1 (Xenopus laevis) | 51339 | ENSG00000165617 |
| 27 | 204863\_s\_at | NA | IL6ST | interleukin 6 signal transducer (gp130, oncostatin M receptor) | 3572 | ENSG00000134352 |
| 28 | 225116\_at | NA | HIPK2 | homeodomain interacting protein kinase 2 | 28996 | ENSG00000064393 |
| 29 | 211506\_s\_at | NA | IL8 | interleukin 8 | 3576 | ENSG00000169429 |
| 30 | 202859\_x\_at | NA | IL8 | interleukin 8 | 3576 | ENSG00000169429 |
| 31 | 204621\_s\_at | NA | NR4A2 | nuclear receptor subfamily 4, group A, member 2 | 4929 | ENSG00000153234 |
| 32 | 200952\_s\_at | NA | CCND2 | cyclin D2 | 894 | ENSG00000118971 |
| 33 | 227740\_at | NA | UHMK1 | U2AF homology motif (UHM) kinase 1 | 127933 | ENSG00000152332 |
| 34 | 235003\_at | NA | UHMK1 | U2AF homology motif (UHM) kinase 1 | 127933 | ENSG00000152332 |
| 35 | 224851\_at | NA | CDK6 | cyclin-dependent kinase 6 | 1021 | ENSG00000105810 |
| 36 | 209396\_s\_at | NA | CHI3L1 | chitinase 3-like 1 (cartilage glycoprotein-39) | 1116 | ENSG00000133048 |
| 37 | 209395\_at | NA | CHI3L1 | chitinase 3-like 1 (cartilage glycoprotein-39) | 1116 | ENSG00000133048 |
| 38 | 230170\_at | NA | OSM | oncostatin M | 5008 | ENSG00000099985 |

  
  

| **Database:biological process      &nbspName:positive regulation of cellular process      &nbspID:GO:0048522** | | | | | | |
| --- | --- | --- | --- | --- | --- | --- |
| C=3007; O=38; E=19.90; R=1.91; rawP=1.53e-05; adjP=0.0019 | | | | | | |
| Index | UserID | Value | Gene Symbol | Gene Name | EntrezGene | Ensembl |
| 1 | 222895\_s\_at | NA | BCL11B | B-cell CLL/lymphoma 11B (zinc finger protein) | 64919 | ENSG00000127152 |
| 2 | 209369\_at | NA | ANXA3 | annexin A3 | 306 | ENSG00000138772 |
| 3 | 212531\_at | NA | LCN2 | lipocalin 2 | 3934 | ENSG00000148346 |
| 4 | 208892\_s\_at | NA | DUSP6 | dual specificity phosphatase 6 | 1848 | ENSG00000139318 |
| 5 | 208891\_at | NA | DUSP6 | dual specificity phosphatase 6 | 1848 | ENSG00000139318 |
| 6 | 206804\_at | NA | CD3G | CD3g molecule, gamma (CD3-TCR complex) | 917 | ENSG00000160654 |
| 7 | 215078\_at | NA | SOD2 | superoxide dismutase 2, mitochondrial | 6648 | ENSG00000112096 |
| 8 | 229228\_at | NA | CREB5 | cAMP responsive element binding protein 5 | 9586 | ENSG00000146592 |
| 9 | 226071\_at | NA | ADAMTSL4 | ADAMTS-like 4 | 54507 | ENSG00000143382 |
| 10 | 235567\_at | NA | RORA | RAR-related orphan receptor A | 6095 | ENSG00000069667 |
| 11 | 218394\_at | NA | ROGDI | rogdi homolog (Drosophila) | 79641 | ENSG00000067836 |
| 12 | 243296\_at | NA | NAMPT | nicotinamide phosphoribosyltransferase | 10135 | ENSG00000105835 |
| 13 | 201109\_s\_at | NA | THBS1 | thrombospondin 1 | 7057 | ENSG00000137801 |
| 14 | 235086\_at | NA | THBS1 | thrombospondin 1 | 7057 | ENSG00000137801 |
| 15 | 210461\_s\_at | NA | ABLIM1 | actin binding LIM protein 1 | 3983 | ENSG00000099204 |
| 16 | 219221\_at | NA | ZBTB38 | zinc finger and BTB domain containing 38 | 253461 | ENSG00000177311 |
| 17 | 203628\_at | NA | IGF1R | insulin-like growth factor 1 receptor | 3480 | ENSG00000140443 |
| 18 | 204285\_s\_at | NA | PMAIP1 | phorbol-12-myristate-13-acetate-induced protein 1 | 5366 | ENSG00000141682 |
| 19 | 219179\_at | NA | DACT1 | dapper, antagonist of beta-catenin, homolog 1 (Xenopus laevis) | 51339 | ENSG00000165617 |
| 20 | 204863\_s\_at | NA | IL6ST | interleukin 6 signal transducer (gp130, oncostatin M receptor) | 3572 | ENSG00000134352 |
| 21 | 204103\_at | NA | CCL4 | chemokine (C-C motif) ligand 4 | 6351 | ENSG00000129277 |
| 22 | 219890\_at | NA | CLEC5A | C-type lectin domain family 5, member A | 23601 | ENSG00000258227 |
| 23 | 200952\_s\_at | NA | CCND2 | cyclin D2 | 894 | ENSG00000118971 |
| 24 | 224851\_at | NA | CDK6 | cyclin-dependent kinase 6 | 1021 | ENSG00000105810 |
| 25 | 230170\_at | NA | OSM | oncostatin M | 5008 | ENSG00000099985 |
| 26 | 243423\_at | NA | TNIP1 | TNFAIP3 interacting protein 1 | 10318 | ENSG00000145901 |
| 27 | 202644\_s\_at | NA | TNFAIP3 | tumor necrosis factor, alpha-induced protein 3 | 7128 | ENSG00000118503 |
| 28 | 202643\_s\_at | NA | TNFAIP3 | tumor necrosis factor, alpha-induced protein 3 | 7128 | ENSG00000118503 |
| 29 | 212240\_s\_at | NA | PIK3R1 | phosphoinositide-3-kinase, regulatory subunit 1 (alpha) | 5295 | ENSG00000145675 |
| 30 | 212249\_at | NA | PIK3R1 | phosphoinositide-3-kinase, regulatory subunit 1 (alpha) | 5295 | ENSG00000145675 |
| 31 | 209840\_s\_at | NA | LRRN3 | leucine rich repeat neuronal 3 | 54674 | ENSG00000173114 |
| 32 | 242774\_at | NA | SYNE2 | spectrin repeat containing, nuclear envelope 2 | 23224 | ENSG00000054654 |
| 33 | 214329\_x\_at | NA | TNFSF10 | tumor necrosis factor (ligand) superfamily, member 10 | 8743 | ENSG00000121858 |
| 34 | 206871\_at | NA | ELANE | elastase, neutrophil expressed | 1991 | ENSG00000197561 |
| 35 | 213998\_s\_at | NA | DDX17 | DEAD (Asp-Glu-Ala-Asp) box helicase 17 | 10521 | ENSG00000100201 |
| 36 | 208719\_s\_at | NA | DDX17 | DEAD (Asp-Glu-Ala-Asp) box helicase 17 | 10521 | ENSG00000100201 |
| 37 | 208151\_x\_at | NA | DDX17 | DEAD (Asp-Glu-Ala-Asp) box helicase 17 | 10521 | ENSG00000100201 |
| 38 | 211919\_s\_at | NA | CXCR4 | chemokine (C-X-C motif) receptor 4 | 7852 | ENSG00000121966 |
| 39 | 209201\_x\_at | NA | CXCR4 | chemokine (C-X-C motif) receptor 4 | 7852 | ENSG00000121966 |
| 40 | 225116\_at | NA | HIPK2 | homeodomain interacting protein kinase 2 | 28996 | ENSG00000064393 |
| 41 | 213524\_s\_at | NA | G0S2 | G0/G1switch 2 | 50486 | ENSG00000123689 |
| 42 | 211506\_s\_at | NA | IL8 | interleukin 8 | 3576 | ENSG00000169429 |
| 43 | 202859\_x\_at | NA | IL8 | interleukin 8 | 3576 | ENSG00000169429 |
| 44 | 204621\_s\_at | NA | NR4A2 | nuclear receptor subfamily 4, group A, member 2 | 4929 | ENSG00000153234 |
| 45 | 227740\_at | NA | UHMK1 | U2AF homology motif (UHM) kinase 1 | 127933 | ENSG00000152332 |
| 46 | 235003\_at | NA | UHMK1 | U2AF homology motif (UHM) kinase 1 | 127933 | ENSG00000152332 |
| 47 | 209396\_s\_at | NA | CHI3L1 | chitinase 3-like 1 (cartilage glycoprotein-39) | 1116 | ENSG00000133048 |
| 48 | 209395\_at | NA | CHI3L1 | chitinase 3-like 1 (cartilage glycoprotein-39) | 1116 | ENSG00000133048 |

  
  

| **Database:biological process      &nbspName:positive regulation of macromolecule metabolic process      &nbspID:GO:0010604** | | | | | | |
| --- | --- | --- | --- | --- | --- | --- |
| C=1793; O=27; E=11.86; R=2.28; rawP=2.31e-05; adjP=0.0026 | | | | | | |
| Index | UserID | Value | Gene Symbol | Gene Name | EntrezGene | Ensembl |
| 1 | 222895\_s\_at | NA | BCL11B | B-cell CLL/lymphoma 11B (zinc finger protein) | 64919 | ENSG00000127152 |
| 2 | 209369\_at | NA | ANXA3 | annexin A3 | 306 | ENSG00000138772 |
| 3 | 202644\_s\_at | NA | TNFAIP3 | tumor necrosis factor, alpha-induced protein 3 | 7128 | ENSG00000118503 |
| 4 | 202643\_s\_at | NA | TNFAIP3 | tumor necrosis factor, alpha-induced protein 3 | 7128 | ENSG00000118503 |
| 5 | 243423\_at | NA | TNIP1 | TNFAIP3 interacting protein 1 | 10318 | ENSG00000145901 |
| 6 | 212531\_at | NA | LCN2 | lipocalin 2 | 3934 | ENSG00000148346 |
| 7 | 212240\_s\_at | NA | PIK3R1 | phosphoinositide-3-kinase, regulatory subunit 1 (alpha) | 5295 | ENSG00000145675 |
| 8 | 212249\_at | NA | PIK3R1 | phosphoinositide-3-kinase, regulatory subunit 1 (alpha) | 5295 | ENSG00000145675 |
| 9 | 209840\_s\_at | NA | LRRN3 | leucine rich repeat neuronal 3 | 54674 | ENSG00000173114 |
| 10 | 206871\_at | NA | ELANE | elastase, neutrophil expressed | 1991 | ENSG00000197561 |
| 11 | 229228\_at | NA | CREB5 | cAMP responsive element binding protein 5 | 9586 | ENSG00000146592 |
| 12 | 235567\_at | NA | RORA | RAR-related orphan receptor A | 6095 | ENSG00000069667 |
| 13 | 243296\_at | NA | NAMPT | nicotinamide phosphoribosyltransferase | 10135 | ENSG00000105835 |
| 14 | 201109\_s\_at | NA | THBS1 | thrombospondin 1 | 7057 | ENSG00000137801 |
| 15 | 235086\_at | NA | THBS1 | thrombospondin 1 | 7057 | ENSG00000137801 |
| 16 | 213998\_s\_at | NA | DDX17 | DEAD (Asp-Glu-Ala-Asp) box helicase 17 | 10521 | ENSG00000100201 |
| 17 | 208719\_s\_at | NA | DDX17 | DEAD (Asp-Glu-Ala-Asp) box helicase 17 | 10521 | ENSG00000100201 |
| 18 | 208151\_x\_at | NA | DDX17 | DEAD (Asp-Glu-Ala-Asp) box helicase 17 | 10521 | ENSG00000100201 |
| 19 | 210461\_s\_at | NA | ABLIM1 | actin binding LIM protein 1 | 3983 | ENSG00000099204 |
| 20 | 211919\_s\_at | NA | CXCR4 | chemokine (C-X-C motif) receptor 4 | 7852 | ENSG00000121966 |
| 21 | 209201\_x\_at | NA | CXCR4 | chemokine (C-X-C motif) receptor 4 | 7852 | ENSG00000121966 |
| 22 | 219221\_at | NA | ZBTB38 | zinc finger and BTB domain containing 38 | 253461 | ENSG00000177311 |
| 23 | 203628\_at | NA | IGF1R | insulin-like growth factor 1 receptor | 3480 | ENSG00000140443 |
| 24 | 219179\_at | NA | DACT1 | dapper, antagonist of beta-catenin, homolog 1 (Xenopus laevis) | 51339 | ENSG00000165617 |
| 25 | 204863\_s\_at | NA | IL6ST | interleukin 6 signal transducer (gp130, oncostatin M receptor) | 3572 | ENSG00000134352 |
| 26 | 225116\_at | NA | HIPK2 | homeodomain interacting protein kinase 2 | 28996 | ENSG00000064393 |
| 27 | 211506\_s\_at | NA | IL8 | interleukin 8 | 3576 | ENSG00000169429 |
| 28 | 202859\_x\_at | NA | IL8 | interleukin 8 | 3576 | ENSG00000169429 |
| 29 | 204621\_s\_at | NA | NR4A2 | nuclear receptor subfamily 4, group A, member 2 | 4929 | ENSG00000153234 |
| 30 | 200952\_s\_at | NA | CCND2 | cyclin D2 | 894 | ENSG00000118971 |
| 31 | 227740\_at | NA | UHMK1 | U2AF homology motif (UHM) kinase 1 | 127933 | ENSG00000152332 |
| 32 | 235003\_at | NA | UHMK1 | U2AF homology motif (UHM) kinase 1 | 127933 | ENSG00000152332 |
| 33 | 224851\_at | NA | CDK6 | cyclin-dependent kinase 6 | 1021 | ENSG00000105810 |
| 34 | 209396\_s\_at | NA | CHI3L1 | chitinase 3-like 1 (cartilage glycoprotein-39) | 1116 | ENSG00000133048 |
| 35 | 209395\_at | NA | CHI3L1 | chitinase 3-like 1 (cartilage glycoprotein-39) | 1116 | ENSG00000133048 |
| 36 | 230170\_at | NA | OSM | oncostatin M | 5008 | ENSG00000099985 |

  
  

| **Database:biological process      &nbspName:response to bacterium      &nbspID:GO:0009617** | | | | | | |
| --- | --- | --- | --- | --- | --- | --- |
| C=319; O=10; E=2.11; R=4.74; rawP=4.77e-05; adjP=0.0047 | | | | | | |
| Index | UserID | Value | Gene Symbol | Gene Name | EntrezGene | Ensembl |
| 1 | 207269\_at | NA | DEFA4 | defensin, alpha 4, corticostatin | 1669 | ENSG00000164821 |
| 2 | 209369\_at | NA | ANXA3 | annexin A3 | 306 | ENSG00000138772 |
| 3 | 202644\_s\_at | NA | TNFAIP3 | tumor necrosis factor, alpha-induced protein 3 | 7128 | ENSG00000118503 |
| 4 | 202643\_s\_at | NA | TNFAIP3 | tumor necrosis factor, alpha-induced protein 3 | 7128 | ENSG00000118503 |
| 5 | 212531\_at | NA | LCN2 | lipocalin 2 | 3934 | ENSG00000148346 |
| 6 | 211506\_s\_at | NA | IL8 | interleukin 8 | 3576 | ENSG00000169429 |
| 7 | 202859\_x\_at | NA | IL8 | interleukin 8 | 3576 | ENSG00000169429 |
| 8 | 207384\_at | NA | PGLYRP1 | peptidoglycan recognition protein 1 | 8993 | ENSG00000008438 |
| 9 | 215078\_at | NA | SOD2 | superoxide dismutase 2, mitochondrial | 6648 | ENSG00000112096 |
| 10 | 232311\_at | NA | B2M | beta-2-microglobulin | 567 | ENSG00000166710 |
| 11 | 210244\_at | NA | CAMP | cathelicidin antimicrobial peptide | 820 | ENSG00000164047 |
| 12 | 202018\_s\_at | NA | LTF | lactotransferrin | 4057 | ENSG00000012223 |

  
  

| **Database:molecular function      &nbspName:insulin binding      &nbspID:GO:0043559** | | | | | | |
| --- | --- | --- | --- | --- | --- | --- |
| C=5; O=2; E=0.03; R=67.45; rawP=0.0003; adjP=0.0237 | | | | | | |
| Index | UserID | Value | Gene Symbol | Gene Name | EntrezGene | Ensembl |
| 1 | 203628\_at | NA | IGF1R | insulin-like growth factor 1 receptor | 3480 | ENSG00000140443 |
| 2 | 212240\_s\_at | NA | PIK3R1 | phosphoinositide-3-kinase, regulatory subunit 1 (alpha) | 5295 | ENSG00000145675 |
| 3 | 212249\_at | NA | PIK3R1 | phosphoinositide-3-kinase, regulatory subunit 1 (alpha) | 5295 | ENSG00000145675 |

  
  

| **Database:molecular function      &nbspName:protease binding      &nbspID:GO:0002020** | | | | | | |
| --- | --- | --- | --- | --- | --- | --- |
| C=54; O=4; E=0.32; R=12.49; rawP=0.0003; adjP=0.0237 | | | | | | |
| Index | UserID | Value | Gene Symbol | Gene Name | EntrezGene | Ensembl |
| 1 | 206871\_at | NA | ELANE | elastase, neutrophil expressed | 1991 | ENSG00000197561 |
| 2 | 202644\_s\_at | NA | TNFAIP3 | tumor necrosis factor, alpha-induced protein 3 | 7128 | ENSG00000118503 |
| 3 | 202643\_s\_at | NA | TNFAIP3 | tumor necrosis factor, alpha-induced protein 3 | 7128 | ENSG00000118503 |
| 4 | 212531\_at | NA | LCN2 | lipocalin 2 | 3934 | ENSG00000148346 |
| 5 | 226071\_at | NA | ADAMTSL4 | ADAMTS-like 4 | 54507 | ENSG00000143382 |

  
  

| **Database:molecular function      &nbspName:cytokine receptor binding      &nbspID:GO:0005126** | | | | | | |
| --- | --- | --- | --- | --- | --- | --- |
| C=204; O=6; E=1.21; R=4.96; rawP=0.0013; adjP=0.0685 | | | | | | |
| Index | UserID | Value | Gene Symbol | Gene Name | EntrezGene | Ensembl |
| 1 | 214329\_x\_at | NA | TNFSF10 | tumor necrosis factor (ligand) superfamily, member 10 | 8743 | ENSG00000121858 |
| 2 | 204863\_s\_at | NA | IL6ST | interleukin 6 signal transducer (gp130, oncostatin M receptor) | 3572 | ENSG00000134352 |
| 3 | 212240\_s\_at | NA | PIK3R1 | phosphoinositide-3-kinase, regulatory subunit 1 (alpha) | 5295 | ENSG00000145675 |
| 4 | 212249\_at | NA | PIK3R1 | phosphoinositide-3-kinase, regulatory subunit 1 (alpha) | 5295 | ENSG00000145675 |
| 5 | 204103\_at | NA | CCL4 | chemokine (C-C motif) ligand 4 | 6351 | ENSG00000129277 |
| 6 | 211506\_s\_at | NA | IL8 | interleukin 8 | 3576 | ENSG00000169429 |
| 7 | 202859\_x\_at | NA | IL8 | interleukin 8 | 3576 | ENSG00000169429 |
| 8 | 230170\_at | NA | OSM | oncostatin M | 5008 | ENSG00000099985 |

  
  

| **Database:molecular function      &nbspName:cytokine binding      &nbspID:GO:0019955** | | | | | | |
| --- | --- | --- | --- | --- | --- | --- |
| C=60; O=3; E=0.36; R=8.43; rawP=0.0054; adjP=0.0833 | | | | | | |
| Index | UserID | Value | Gene Symbol | Gene Name | EntrezGene | Ensembl |
| 1 | 206871\_at | NA | ELANE | elastase, neutrophil expressed | 1991 | ENSG00000197561 |
| 2 | 204863\_s\_at | NA | IL6ST | interleukin 6 signal transducer (gp130, oncostatin M receptor) | 3572 | ENSG00000134352 |
| 3 | 201109\_s\_at | NA | THBS1 | thrombospondin 1 | 7057 | ENSG00000137801 |
| 4 | 235086\_at | NA | THBS1 | thrombospondin 1 | 7057 | ENSG00000137801 |

  
  

| **Database:molecular function      &nbspName:carbohydrate derivative binding      &nbspID:GO:0097367** | | | | | | |
| --- | --- | --- | --- | --- | --- | --- |
| C=187; O=5; E=1.11; R=4.51; rawP=0.0051; adjP=0.0833 | | | | | | |
| Index | UserID | Value | Gene Symbol | Gene Name | EntrezGene | Ensembl |
| 1 | 206871\_at | NA | ELANE | elastase, neutrophil expressed | 1991 | ENSG00000197561 |
| 2 | 202018\_s\_at | NA | LTF | lactotransferrin | 4057 | ENSG00000012223 |
| 3 | 209396\_s\_at | NA | CHI3L1 | chitinase 3-like 1 (cartilage glycoprotein-39) | 1116 | ENSG00000133048 |
| 4 | 209395\_at | NA | CHI3L1 | chitinase 3-like 1 (cartilage glycoprotein-39) | 1116 | ENSG00000133048 |
| 5 | 207384\_at | NA | PGLYRP1 | peptidoglycan recognition protein 1 | 8993 | ENSG00000008438 |
| 6 | 201109\_s\_at | NA | THBS1 | thrombospondin 1 | 7057 | ENSG00000137801 |
| 7 | 235086\_at | NA | THBS1 | thrombospondin 1 | 7057 | ENSG00000137801 |

  
  

| **Database:molecular function      &nbspName:phosphatidylinositol 3-kinase binding      &nbspID:GO:0043548** | | | | | | |
| --- | --- | --- | --- | --- | --- | --- |
| C=19; O=2; E=0.11; R=17.75; rawP=0.0056; adjP=0.0833 | | | | | | |
| Index | UserID | Value | Gene Symbol | Gene Name | EntrezGene | Ensembl |
| 1 | 203628\_at | NA | IGF1R | insulin-like growth factor 1 receptor | 3480 | ENSG00000140443 |
| 2 | 212240\_s\_at | NA | PIK3R1 | phosphoinositide-3-kinase, regulatory subunit 1 (alpha) | 5295 | ENSG00000145675 |
| 3 | 212249\_at | NA | PIK3R1 | phosphoinositide-3-kinase, regulatory subunit 1 (alpha) | 5295 | ENSG00000145675 |

  
  

| **Database:molecular function      &nbspName:bacterial cell surface binding      &nbspID:GO:0051635** | | | | | | |
| --- | --- | --- | --- | --- | --- | --- |
| C=17; O=2; E=0.10; R=19.84; rawP=0.0045; adjP=0.0833 | | | | | | |
| Index | UserID | Value | Gene Symbol | Gene Name | EntrezGene | Ensembl |
| 1 | 206871\_at | NA | ELANE | elastase, neutrophil expressed | 1991 | ENSG00000197561 |
| 2 | 207384\_at | NA | PGLYRP1 | peptidoglycan recognition protein 1 | 8993 | ENSG00000008438 |

  
  

| **Database:molecular function      &nbspName:cytokine activity      &nbspID:GO:0005125** | | | | | | |
| --- | --- | --- | --- | --- | --- | --- |
| C=193; O=5; E=1.14; R=4.37; rawP=0.0058; adjP=0.0833 | | | | | | |
| Index | UserID | Value | Gene Symbol | Gene Name | EntrezGene | Ensembl |
| 1 | 214329\_x\_at | NA | TNFSF10 | tumor necrosis factor (ligand) superfamily, member 10 | 8743 | ENSG00000121858 |
| 2 | 204103\_at | NA | CCL4 | chemokine (C-C motif) ligand 4 | 6351 | ENSG00000129277 |
| 3 | 211506\_s\_at | NA | IL8 | interleukin 8 | 3576 | ENSG00000169429 |
| 4 | 202859\_x\_at | NA | IL8 | interleukin 8 | 3576 | ENSG00000169429 |
| 5 | 243296\_at | NA | NAMPT | nicotinamide phosphoribosyltransferase | 10135 | ENSG00000105835 |
| 6 | 230170\_at | NA | OSM | oncostatin M | 5008 | ENSG00000099985 |

  
  

| **Database:molecular function      &nbspName:cell surface binding      &nbspID:GO:0043498** | | | | | | |
| --- | --- | --- | --- | --- | --- | --- |
| C=53; O=3; E=0.31; R=9.54; rawP=0.0038; adjP=0.0833 | | | | | | |
| Index | UserID | Value | Gene Symbol | Gene Name | EntrezGene | Ensembl |
| 1 | 206871\_at | NA | ELANE | elastase, neutrophil expressed | 1991 | ENSG00000197561 |
| 2 | 207384\_at | NA | PGLYRP1 | peptidoglycan recognition protein 1 | 8993 | ENSG00000008438 |
| 3 | 201109\_s\_at | NA | THBS1 | thrombospondin 1 | 7057 | ENSG00000137801 |
| 4 | 235086\_at | NA | THBS1 | thrombospondin 1 | 7057 | ENSG00000137801 |

  
  

| **Database:molecular function      &nbspName:insulin receptor substrate binding      &nbspID:GO:0043560** | | | | | | |
| --- | --- | --- | --- | --- | --- | --- |
| C=13; O=2; E=0.08; R=25.94; rawP=0.0026; adjP=0.0833 | | | | | | |
| Index | UserID | Value | Gene Symbol | Gene Name | EntrezGene | Ensembl |
| 1 | 203628\_at | NA | IGF1R | insulin-like growth factor 1 receptor | 3480 | ENSG00000140443 |
| 2 | 212240\_s\_at | NA | PIK3R1 | phosphoinositide-3-kinase, regulatory subunit 1 (alpha) | 5295 | ENSG00000145675 |
| 3 | 212249\_at | NA | PIK3R1 | phosphoinositide-3-kinase, regulatory subunit 1 (alpha) | 5295 | ENSG00000145675 |

  
  

| **Database:cellular component      &nbspName:oncostatin-M receptor complex      &nbspID:GO:0005900** | | | | | | |
| --- | --- | --- | --- | --- | --- | --- |
| C=3; O=2; E=0.02; R=109.72; rawP=0.0001; adjP=0.0115 | | | | | | |
| Index | UserID | Value | Gene Symbol | Gene Name | EntrezGene | Ensembl |
| 1 | 204863\_s\_at | NA | IL6ST | interleukin 6 signal transducer (gp130, oncostatin M receptor) | 3572 | ENSG00000134352 |
| 2 | 230170\_at | NA | OSM | oncostatin M | 5008 | ENSG00000099985 |

  
  

| **Database:cellular component      &nbspName:extracellular region part      &nbspID:GO:0044421** | | | | | | |
| --- | --- | --- | --- | --- | --- | --- |
| C=1039; O=17; E=6.31; R=2.69; rawP=0.0002; adjP=0.0115 | | | | | | |
| Index | UserID | Value | Gene Symbol | Gene Name | EntrezGene | Ensembl |
| 1 | 207269\_at | NA | DEFA4 | defensin, alpha 4, corticostatin | 1669 | ENSG00000164821 |
| 2 | 1554241\_at | NA | COCH | coagulation factor C homolog, cochlin (Limulus polyphemus) | 1690 | ENSG00000100473 |
| 3 | 212531\_at | NA | LCN2 | lipocalin 2 | 3934 | ENSG00000148346 |
| 4 | 214329\_x\_at | NA | TNFSF10 | tumor necrosis factor (ligand) superfamily, member 10 | 8743 | ENSG00000121858 |
| 5 | 232311\_at | NA | B2M | beta-2-microglobulin | 567 | ENSG00000166710 |
| 6 | 206676\_at | NA | CEACAM8 | carcinoembryonic antigen-related cell adhesion molecule 8 | 1088 | ENSG00000124469 |
| 7 | 226071\_at | NA | ADAMTSL4 | ADAMTS-like 4 | 54507 | ENSG00000143382 |
| 8 | 201109\_s\_at | NA | THBS1 | thrombospondin 1 | 7057 | ENSG00000137801 |
| 9 | 235086\_at | NA | THBS1 | thrombospondin 1 | 7057 | ENSG00000137801 |
| 10 | 212768\_s\_at | NA | OLFM4 | olfactomedin 4 | 10562 | ENSG00000102837 |
| 11 | 209257\_s\_at | NA | SMC3 | structural maintenance of chromosomes 3 | 9126 | ENSG00000108055 |
| 12 | 209258\_s\_at | NA | SMC3 | structural maintenance of chromosomes 3 | 9126 | ENSG00000108055 |
| 13 | 204863\_s\_at | NA | IL6ST | interleukin 6 signal transducer (gp130, oncostatin M receptor) | 3572 | ENSG00000134352 |
| 14 | 207329\_at | NA | MMP8 | matrix metallopeptidase 8 (neutrophil collagenase) | 4317 | ENSG00000118113 |
| 15 | 231688\_at | NA | MMP8 | matrix metallopeptidase 8 (neutrophil collagenase) | 4317 | ENSG00000118113 |
| 16 | 211506\_s\_at | NA | IL8 | interleukin 8 | 3576 | ENSG00000169429 |
| 17 | 202859\_x\_at | NA | IL8 | interleukin 8 | 3576 | ENSG00000169429 |
| 18 | 204103\_at | NA | CCL4 | chemokine (C-C motif) ligand 4 | 6351 | ENSG00000129277 |
| 19 | 209396\_s\_at | NA | CHI3L1 | chitinase 3-like 1 (cartilage glycoprotein-39) | 1116 | ENSG00000133048 |
| 20 | 209395\_at | NA | CHI3L1 | chitinase 3-like 1 (cartilage glycoprotein-39) | 1116 | ENSG00000133048 |
| 21 | 207802\_at | NA | CRISP3 | cysteine-rich secretory protein 3 | 10321 | ENSG00000096006 |
| 22 | 230170\_at | NA | OSM | oncostatin M | 5008 | ENSG00000099985 |

  
  

| **Database:cellular component      &nbspName:extracellular space      &nbspID:GO:0005615** | | | | | | |
| --- | --- | --- | --- | --- | --- | --- |
| C=811; O=13; E=4.93; R=2.64; rawP=0.0012; adjP=0.0249 | | | | | | |
| Index | UserID | Value | Gene Symbol | Gene Name | EntrezGene | Ensembl |
| 1 | 207269\_at | NA | DEFA4 | defensin, alpha 4, corticostatin | 1669 | ENSG00000164821 |
| 2 | 212768\_s\_at | NA | OLFM4 | olfactomedin 4 | 10562 | ENSG00000102837 |
| 3 | 212531\_at | NA | LCN2 | lipocalin 2 | 3934 | ENSG00000148346 |
| 4 | 207329\_at | NA | MMP8 | matrix metallopeptidase 8 (neutrophil collagenase) | 4317 | ENSG00000118113 |
| 5 | 231688\_at | NA | MMP8 | matrix metallopeptidase 8 (neutrophil collagenase) | 4317 | ENSG00000118113 |
| 6 | 204863\_s\_at | NA | IL6ST | interleukin 6 signal transducer (gp130, oncostatin M receptor) | 3572 | ENSG00000134352 |
| 7 | 204103\_at | NA | CCL4 | chemokine (C-C motif) ligand 4 | 6351 | ENSG00000129277 |
| 8 | 211506\_s\_at | NA | IL8 | interleukin 8 | 3576 | ENSG00000169429 |
| 9 | 202859\_x\_at | NA | IL8 | interleukin 8 | 3576 | ENSG00000169429 |
| 10 | 214329\_x\_at | NA | TNFSF10 | tumor necrosis factor (ligand) superfamily, member 10 | 8743 | ENSG00000121858 |
| 11 | 232311\_at | NA | B2M | beta-2-microglobulin | 567 | ENSG00000166710 |
| 12 | 206676\_at | NA | CEACAM8 | carcinoembryonic antigen-related cell adhesion molecule 8 | 1088 | ENSG00000124469 |
| 13 | 209396\_s\_at | NA | CHI3L1 | chitinase 3-like 1 (cartilage glycoprotein-39) | 1116 | ENSG00000133048 |
| 14 | 209395\_at | NA | CHI3L1 | chitinase 3-like 1 (cartilage glycoprotein-39) | 1116 | ENSG00000133048 |
| 15 | 201109\_s\_at | NA | THBS1 | thrombospondin 1 | 7057 | ENSG00000137801 |
| 16 | 235086\_at | NA | THBS1 | thrombospondin 1 | 7057 | ENSG00000137801 |
| 17 | 230170\_at | NA | OSM | oncostatin M | 5008 | ENSG00000099985 |

  
  

| **Database:cellular component      &nbspName:external side of plasma membrane      &nbspID:GO:0009897** | | | | | | |
| --- | --- | --- | --- | --- | --- | --- |
| C=193; O=6; E=1.17; R=5.12; rawP=0.0011; adjP=0.0249 | | | | | | |
| Index | UserID | Value | Gene Symbol | Gene Name | EntrezGene | Ensembl |
| 1 | 232311\_at | NA | B2M | beta-2-microglobulin | 567 | ENSG00000166710 |
| 2 | 211919\_s\_at | NA | CXCR4 | chemokine (C-X-C motif) receptor 4 | 7852 | ENSG00000121966 |
| 3 | 209201\_x\_at | NA | CXCR4 | chemokine (C-X-C motif) receptor 4 | 7852 | ENSG00000121966 |
| 4 | 204863\_s\_at | NA | IL6ST | interleukin 6 signal transducer (gp130, oncostatin M receptor) | 3572 | ENSG00000134352 |
| 5 | 231418\_at | NA | MS4A1 | membrane-spanning 4-domains, subfamily A, member 1 | 931 | ENSG00000156738 |
| 6 | 201109\_s\_at | NA | THBS1 | thrombospondin 1 | 7057 | ENSG00000137801 |
| 7 | 235086\_at | NA | THBS1 | thrombospondin 1 | 7057 | ENSG00000137801 |
| 8 | 202888\_s\_at | NA | ANPEP | alanyl (membrane) aminopeptidase | 290 | ENSG00000166825 |

  
  

| **Database:cellular component      &nbspName:specific granule      &nbspID:GO:0042581** | | | | | | |
| --- | --- | --- | --- | --- | --- | --- |
| C=9; O=2; E=0.05; R=36.57; rawP=0.0013; adjP=0.0249 | | | | | | |
| Index | UserID | Value | Gene Symbol | Gene Name | EntrezGene | Ensembl |
| 1 | 209369\_at | NA | ANXA3 | annexin A3 | 306 | ENSG00000138772 |
| 2 | 207802\_at | NA | CRISP3 | cysteine-rich secretory protein 3 | 10321 | ENSG00000096006 |

  
  

| **Database:cellular component      &nbspName:hemoglobin complex      &nbspID:GO:0005833** | | | | | | |
| --- | --- | --- | --- | --- | --- | --- |
| C=9; O=2; E=0.05; R=36.57; rawP=0.0013; adjP=0.0249 | | | | | | |
| Index | UserID | Value | Gene Symbol | Gene Name | EntrezGene | Ensembl |
| 1 | 219672\_at | NA | AHSP | alpha hemoglobin stabilizing protein | 51327 | ENSG00000169877 |
| 2 | 220807\_at | NA | HBQ1 | hemoglobin, theta 1 | 3049 | ENSG00000086506 |

  
  

| **Database:cellular component      &nbspName:extracellular region      &nbspID:GO:0005576** | | | | | | |
| --- | --- | --- | --- | --- | --- | --- |
| C=1914; O=22; E=11.63; R=1.89; rawP=0.0021; adjP=0.0345 | | | | | | |
| Index | UserID | Value | Gene Symbol | Gene Name | EntrezGene | Ensembl |
| 1 | 207269\_at | NA | DEFA4 | defensin, alpha 4, corticostatin | 1669 | ENSG00000164821 |
| 2 | 1554241\_at | NA | COCH | coagulation factor C homolog, cochlin (Limulus polyphemus) | 1690 | ENSG00000100473 |
| 3 | 212531\_at | NA | LCN2 | lipocalin 2 | 3934 | ENSG00000148346 |
| 4 | 207384\_at | NA | PGLYRP1 | peptidoglycan recognition protein 1 | 8993 | ENSG00000008438 |
| 5 | 214329\_x\_at | NA | TNFSF10 | tumor necrosis factor (ligand) superfamily, member 10 | 8743 | ENSG00000121858 |
| 6 | 232311\_at | NA | B2M | beta-2-microglobulin | 567 | ENSG00000166710 |
| 7 | 206871\_at | NA | ELANE | elastase, neutrophil expressed | 1991 | ENSG00000197561 |
| 8 | 206676\_at | NA | CEACAM8 | carcinoembryonic antigen-related cell adhesion molecule 8 | 1088 | ENSG00000124469 |
| 9 | 210244\_at | NA | CAMP | cathelicidin antimicrobial peptide | 820 | ENSG00000164047 |
| 10 | 226071\_at | NA | ADAMTSL4 | ADAMTS-like 4 | 54507 | ENSG00000143382 |
| 11 | 202018\_s\_at | NA | LTF | lactotransferrin | 4057 | ENSG00000012223 |
| 12 | 201109\_s\_at | NA | THBS1 | thrombospondin 1 | 7057 | ENSG00000137801 |
| 13 | 235086\_at | NA | THBS1 | thrombospondin 1 | 7057 | ENSG00000137801 |
| 14 | 209257\_s\_at | NA | SMC3 | structural maintenance of chromosomes 3 | 9126 | ENSG00000108055 |
| 15 | 209258\_s\_at | NA | SMC3 | structural maintenance of chromosomes 3 | 9126 | ENSG00000108055 |
| 16 | 212768\_s\_at | NA | OLFM4 | olfactomedin 4 | 10562 | ENSG00000102837 |
| 17 | 204863\_s\_at | NA | IL6ST | interleukin 6 signal transducer (gp130, oncostatin M receptor) | 3572 | ENSG00000134352 |
| 18 | 207329\_at | NA | MMP8 | matrix metallopeptidase 8 (neutrophil collagenase) | 4317 | ENSG00000118113 |
| 19 | 231688\_at | NA | MMP8 | matrix metallopeptidase 8 (neutrophil collagenase) | 4317 | ENSG00000118113 |
| 20 | 204103\_at | NA | CCL4 | chemokine (C-C motif) ligand 4 | 6351 | ENSG00000129277 |
| 21 | 211506\_s\_at | NA | IL8 | interleukin 8 | 3576 | ENSG00000169429 |
| 22 | 202859\_x\_at | NA | IL8 | interleukin 8 | 3576 | ENSG00000169429 |
| 23 | 205513\_at | NA | TCN1 | transcobalamin I (vitamin B12 binding protein, R binder family) | 6947 | ENSG00000134827 |
| 24 | 209396\_s\_at | NA | CHI3L1 | chitinase 3-like 1 (cartilage glycoprotein-39) | 1116 | ENSG00000133048 |
| 25 | 209395\_at | NA | CHI3L1 | chitinase 3-like 1 (cartilage glycoprotein-39) | 1116 | ENSG00000133048 |
| 26 | 207802\_at | NA | CRISP3 | cysteine-rich secretory protein 3 | 10321 | ENSG00000096006 |
| 27 | 230170\_at | NA | OSM | oncostatin M | 5008 | ENSG00000099985 |

  
  

| **Database:cellular component      &nbspName:vesicle lumen      &nbspID:GO:0031983** | | | | | | |
| --- | --- | --- | --- | --- | --- | --- |
| C=52; O=3; E=0.32; R=9.50; rawP=0.0039; adjP=0.0471 | | | | | | |
| Index | UserID | Value | Gene Symbol | Gene Name | EntrezGene | Ensembl |
| 1 | 202018\_s\_at | NA | LTF | lactotransferrin | 4057 | ENSG00000012223 |
| 2 | 201109\_s\_at | NA | THBS1 | thrombospondin 1 | 7057 | ENSG00000137801 |
| 3 | 235086\_at | NA | THBS1 | thrombospondin 1 | 7057 | ENSG00000137801 |
| 4 | 202888\_s\_at | NA | ANPEP | alanyl (membrane) aminopeptidase | 290 | ENSG00000166825 |

  
  

| **Database:cellular component      &nbspName:phagocytic vesicle      &nbspID:GO:0045335** | | | | | | |
| --- | --- | --- | --- | --- | --- | --- |
| C=49; O=3; E=0.30; R=10.08; rawP=0.0033; adjP=0.0471 | | | | | | |
| Index | UserID | Value | Gene Symbol | Gene Name | EntrezGene | Ensembl |
| 1 | 232311\_at | NA | B2M | beta-2-microglobulin | 567 | ENSG00000166710 |
| 2 | 209369\_at | NA | ANXA3 | annexin A3 | 306 | ENSG00000138772 |
| 3 | 202018\_s\_at | NA | LTF | lactotransferrin | 4057 | ENSG00000012223 |

  
  

| **Database:cellular component      &nbspName:cyclin-dependent protein kinase holoenzyme complex      &nbspID:GO:0000307** | | | | | | |
| --- | --- | --- | --- | --- | --- | --- |
| C=16; O=2; E=0.10; R=20.57; rawP=0.0041; adjP=0.0471 | | | | | | |
| Index | UserID | Value | Gene Symbol | Gene Name | EntrezGene | Ensembl |
| 1 | 200952\_s\_at | NA | CCND2 | cyclin D2 | 894 | ENSG00000118971 |
| 2 | 224851\_at | NA | CDK6 | cyclin-dependent kinase 6 | 1021 | ENSG00000105810 |

  
  
  
  


---

WebGestalt is currently developed and maintained by Jing Wang and Bing Zhang at the  Zhang Lab. Other people who have made significant contribution to the project include Dexter Duncan, Stefan Kirov, Zhiao Shi, and Jay Snoddy.  
  
**Funding credits:** NIH/NIAAA (U01 AA016662, U01 AA013512); NIH/NIDA (P01 DA015027); NIH/NIMH (P50 MH078028, P50 MH096972); NIH/NCI (U24 CA159988); NIH/NIGMS (R01 GM088822).
