# Supplementary material for: Behçet's: A Disease or a Syndrome? Answer from an Expression Profiling Study
Source: PLoS One. 2016 Feb 18;11(2):e0149052. doi: 10.1371/journal.pone.0149052 (PMC4758705; doi:10.1371/journal.pone.0149052)
Supplement: S7 File — (HTML) [file pone.0149052.s007.html]

Anchored HTML File of EIDs


|  |  |
| --- | --- |
|  | WEB-based GEne SeT AnaLysis Toolkit |
|  |  |
|  | ***Translating gene lists into biological insights...*** |
|  |  |

---

  

| **Database:biological process      &nbspName:protein modification process      &nbspID:GO:0036211** | | | | | | |
| --- | --- | --- | --- | --- | --- | --- |
| C=2409; O=57; E=29.60; R=1.93; rawP=2.39e-07; adjP=0.0001 | | | | | | |
| Index | UserID | Value | Gene Symbol | Gene Name | EntrezGene | Ensembl |
| 1 | 206919\_at | NA | ELK4 | ELK4, ETS-domain protein (SRF accessory protein 1) | 2005 | ENSG00000158711 |
| 2 | 202221\_s\_at | NA | EP300 | E1A binding protein p300 | 2033 | ENSG00000100393 |
| 3 | 204794\_at | NA | DUSP2 | dual specificity phosphatase 2 | 1844 | ENSG00000158050 |
| 4 | 208121\_s\_at | NA | PTPRO | protein tyrosine phosphatase, receptor type, O | 5800 | ENSG00000151490 |
| 5 | 228106\_at | NA | DCAF16 | DDB1 and CUL4 associated factor 16 | 54876 | ENSG00000163257 |
| 6 | 225760\_at | NA | MYSM1 | Myb-like, SWIRM and MPN domains 1 | 114803 | ENSG00000162601 |
| 7 | 202375\_at | NA | SEC24D | SEC24 family, member D (S. cerevisiae) | 9871 | ENSG00000150961 |
| 8 | 226366\_at | NA | SHPRH | SNF2 histone linker PHD RING helicase, E3 ubiquitin protein ligase | 257218 | ENSG00000146414 |
| 9 | 214917\_at | NA | PRKAA1 | protein kinase, AMP-activated, alpha 1 catalytic subunit | 5562 | ENSG00000132356 |
| 10 | 227449\_at | NA | EPHA4 | EPH receptor A4 | 2043 | ENSG00000116106 |
| 11 | 220018\_at | NA | CBLL1 | Cbl proto-oncogene, E3 ubiquitin protein ligase-like 1 | 79872 | ENSG00000105879 |
| 12 | 1554309\_at | NA | EIF4G3 | eukaryotic translation initiation factor 4 gamma, 3 | 8672 | ENSG00000075151 |
| 13 | 235300\_x\_at | NA | RCHY1 | ring finger and CHY zinc finger domain containing 1, E3 ubiquitin protein ligase | 25898 | ENSG00000163743 |
| 14 | 213225\_at | NA | PPM1B | protein phosphatase, Mg2+/Mn2+ dependent, 1B | 5495 | ENSG00000138032 |
| 15 | 210746\_s\_at | NA | EPB42 | erythrocyte membrane protein band 4.2 | 2038 | ENSG00000166947 |
| 16 | 200602\_at | NA | APP | amyloid beta (A4) precursor protein | 351 | ENSG00000142192 |
| 17 | 202843\_at | NA | DNAJB9 | DnaJ (Hsp40) homolog, subfamily B, member 9 | 4189 | ENSG00000128590 |
| 18 | 203358\_s\_at | NA | EZH2 | enhancer of zeste homolog 2 (Drosophila) | 2146 | ENSG00000106462 |
| 19 | 208762\_at | NA | SUMO1 | SMT3 suppressor of mif two 3 homolog 1 (S. cerevisiae) | 7341 | ENSG00000116030 |
| 20 | 202887\_s\_at | NA | DDIT4 | DNA-damage-inducible transcript 4 | 54541 | ENSG00000168209 |
| 21 | 216929\_x\_at | NA | ABO | ABO blood group (transferase A, alpha 1-3-N-acetylgalactosaminyltransferase; transferase B, alpha 1-3-galactosyltransferase) | 28 | NULL |
| 22 | 204176\_at | NA | KLHL20 | kelch-like 20 (Drosophila) | 27252 | ENSG00000076321 |
| 23 | 203552\_at | NA | MAP4K5 | mitogen-activated protein kinase kinase kinase kinase 5 | 11183 | ENSG00000012983 |
| 24 | 201177\_s\_at | NA | UBA2 | ubiquitin-like modifier activating enzyme 2 | 10054 | ENSG00000126261 |
| 25 | 1569136\_at | NA | MGAT4A | mannosyl (alpha-1,3-)-glycoprotein beta-1,4-N-acetylglucosaminyltransferase, isozyme A | 11320 | ENSG00000071073 |
| 26 | 238020\_at | NA | PSMC2 | proteasome (prosome, macropain) 26S subunit, ATPase, 2 | 5701 | ENSG00000161057 |
| 27 | 222858\_s\_at | NA | DAPP1 | dual adaptor of phosphotyrosine and 3-phosphoinositides | 27071 | ENSG00000070190 |
| 28 | 212637\_s\_at | NA | WWP1 | WW domain containing E3 ubiquitin protein ligase 1 | 11059 | ENSG00000123124 |
| 29 | 214590\_s\_at | NA | UBE2D1 | ubiquitin-conjugating enzyme E2D 1 | 7321 | ENSG00000072401 |
| 30 | 208078\_s\_at | NA | SIK1 | salt-inducible kinase 1 | 150094 | ENSG00000142178 |
| 31 | 232068\_s\_at | NA | TLR4 | toll-like receptor 4 | 7099 | ENSG00000136869 |
| 32 | 230170\_at | NA | OSM | oncostatin M | 5008 | ENSG00000099985 |
| 33 | 1555962\_at | NA | B3GNT7 | UDP-GlcNAc:betaGal beta-1,3-N-acetylglucosaminyltransferase 7 | 93010 | ENSG00000156966 |
| 34 | 229943\_at | NA | TRIM13 | tripartite motif containing 13 | 10206 | ENSG00000204977 |
| 35 | 202644\_s\_at | NA | TNFAIP3 | tumor necrosis factor, alpha-induced protein 3 | 7128 | ENSG00000118503 |
| 36 | 211698\_at | NA | EID1 | EP300 interacting inhibitor of differentiation 1 | 23741 | ENSG00000255302 |
| 37 | 202203\_s\_at | NA | AMFR | autocrine motility factor receptor, E3 ubiquitin protein ligase | 267 | ENSG00000159461 |
| 38 | 223266\_at | NA | STRADB | STE20-related kinase adaptor beta | 55437 | ENSG00000082146 |
| 39 | 231863\_at | NA | ING3 | inhibitor of growth family, member 3 | 54556 | ENSG00000071243 |
| 40 | 202933\_s\_at | NA | YES1 | v-yes-1 Yamaguchi sarcoma viral oncogene homolog 1 | 7525 | ENSG00000176105 |
| 41 | 205283\_at | NA | FKTN | fukutin | 2218 | ENSG00000106692 |
| 42 | 236539\_at | NA | PTPN22 | protein tyrosine phosphatase, non-receptor type 22 (lymphoid) | 26191 | ENSG00000134242 |
| 43 | 204995\_at | NA | CDK5R1 | cyclin-dependent kinase 5, regulatory subunit 1 (p35) | 8851 | ENSG00000176749 |
| 44 | 1557257\_at | NA | BCL10 | B-cell CLL/lymphoma 10 | 8915 | ENSG00000142867 |
| 45 | 211546\_x\_at | NA | SNCA | synuclein, alpha (non A4 component of amyloid precursor) | 6622 | ENSG00000145335 |
| 46 | 207827\_x\_at | NA | SNCA | synuclein, alpha (non A4 component of amyloid precursor) | 6622 | ENSG00000145335 |
| 47 | 236081\_at | NA | SNCA | synuclein, alpha (non A4 component of amyloid precursor) | 6622 | ENSG00000145335 |
| 48 | 204467\_s\_at | NA | SNCA | synuclein, alpha (non A4 component of amyloid precursor) | 6622 | ENSG00000145335 |
| 49 | 204466\_s\_at | NA | SNCA | synuclein, alpha (non A4 component of amyloid precursor) | 6622 | ENSG00000145335 |
| 50 | 227697\_at | NA | SOCS3 | suppressor of cytokine signaling 3 | 9021 | ENSG00000184557 |
| 51 | 1559975\_at | NA | BTG1 | B-cell translocation gene 1, anti-proliferative | 694 | ENSG00000133639 |
| 52 | 218411\_s\_at | NA | MBIP | MAP3K12 binding inhibitory protein 1 | 51562 | ENSG00000151332 |
| 53 | 239143\_x\_at | NA | RNF138 | ring finger protein 138, E3 ubiquitin protein ligase | 51444 | ENSG00000134758 |
| 54 | 205767\_at | NA | EREG | epiregulin | 2069 | ENSG00000124882 |
| 55 | 219304\_s\_at | NA | PDGFD | platelet derived growth factor D | 80310 | ENSG00000170962 |
| 56 | 201407\_s\_at | NA | PPP1CB | protein phosphatase 1, catalytic subunit, beta isozyme | 5500 | ENSG00000213639 |
| 57 | 230559\_x\_at | NA | FGD4 | FYVE, RhoGEF and PH domain containing 4 | 121512 | ENSG00000139132 |
| 58 | 212633\_at | NA | UFL1 | UFM1-specific ligase 1 | 23376 | ENSG00000014123 |
| 59 | 207300\_s\_at | NA | F7 | coagulation factor VII (serum prothrombin conversion accelerator) | 2155 | ENSG00000057593 |
| 60 | 232044\_at | NA | RBBP6 | retinoblastoma binding protein 6 | 5930 | ENSG00000122257 |
| 61 | 203603\_s\_at | NA | ZEB2 | zinc finger E-box binding homeobox 2 | 9839 | ENSG00000169554 |

  
  

| **Database:biological process      &nbspName:cellular protein metabolic process      &nbspID:GO:0044267** | | | | | | |
| --- | --- | --- | --- | --- | --- | --- |
| C=3150; O=69; E=38.71; R=1.78; rawP=1.31e-07; adjP=0.0001 | | | | | | |
| Index | UserID | Value | Gene Symbol | Gene Name | EntrezGene | Ensembl |
| 1 | 206919\_at | NA | ELK4 | ELK4, ETS-domain protein (SRF accessory protein 1) | 2005 | ENSG00000158711 |
| 2 | 202221\_s\_at | NA | EP300 | E1A binding protein p300 | 2033 | ENSG00000100393 |
| 3 | 208121\_s\_at | NA | PTPRO | protein tyrosine phosphatase, receptor type, O | 5800 | ENSG00000151490 |
| 4 | 228106\_at | NA | DCAF16 | DDB1 and CUL4 associated factor 16 | 54876 | ENSG00000163257 |
| 5 | 203821\_at | NA | HBEGF | heparin-binding EGF-like growth factor | 1839 | ENSG00000113070 |
| 6 | 225760\_at | NA | MYSM1 | Myb-like, SWIRM and MPN domains 1 | 114803 | ENSG00000162601 |
| 7 | 213725\_x\_at | NA | XYLT1 | xylosyltransferase I | 64131 | ENSG00000103489 |
| 8 | 227449\_at | NA | EPHA4 | EPH receptor A4 | 2043 | ENSG00000116106 |
| 9 | 201016\_at | NA | EIF1AX | eukaryotic translation initiation factor 1A, X-linked | 1964 | ENSG00000173674 |
| 10 | 235300\_x\_at | NA | RCHY1 | ring finger and CHY zinc finger domain containing 1, E3 ubiquitin protein ligase | 25898 | ENSG00000163743 |
| 11 | 213225\_at | NA | PPM1B | protein phosphatase, Mg2+/Mn2+ dependent, 1B | 5495 | ENSG00000138032 |
| 12 | 200602\_at | NA | APP | amyloid beta (A4) precursor protein | 351 | ENSG00000142192 |
| 13 | 201177\_s\_at | NA | UBA2 | ubiquitin-like modifier activating enzyme 2 | 10054 | ENSG00000126261 |
| 14 | 238020\_at | NA | PSMC2 | proteasome (prosome, macropain) 26S subunit, ATPase, 2 | 5701 | ENSG00000161057 |
| 15 | 213459\_at | NA | RPL37A | ribosomal protein L37a | 6168 | ENSG00000197756 |
| 16 | 205191\_at | NA | RP2 | retinitis pigmentosa 2 (X-linked recessive) | 6102 | ENSG00000102218 |
| 17 | 214590\_s\_at | NA | UBE2D1 | ubiquitin-conjugating enzyme E2D 1 | 7321 | ENSG00000072401 |
| 18 | 201437\_s\_at | NA | EIF4E | eukaryotic translation initiation factor 4E | 1977 | ENSG00000151247 |
| 19 | 232068\_s\_at | NA | TLR4 | toll-like receptor 4 | 7099 | ENSG00000136869 |
| 20 | 218871\_x\_at | NA | CSGALNACT2 | chondroitin sulfate N-acetylgalactosaminyltransferase 2 | 55454 | ENSG00000169826 |
| 21 | 230170\_at | NA | OSM | oncostatin M | 5008 | ENSG00000099985 |
| 22 | 229943\_at | NA | TRIM13 | tripartite motif containing 13 | 10206 | ENSG00000204977 |
| 23 | 211698\_at | NA | EID1 | EP300 interacting inhibitor of differentiation 1 | 23741 | ENSG00000255302 |
| 24 | 236539\_at | NA | PTPN22 | protein tyrosine phosphatase, non-receptor type 22 (lymphoid) | 26191 | ENSG00000134242 |
| 25 | 205283\_at | NA | FKTN | fukutin | 2218 | ENSG00000106692 |
| 26 | 226472\_at | NA | PPIL4 | peptidylprolyl isomerase (cyclophilin)-like 4 | 85313 | ENSG00000131013 |
| 27 | 211546\_x\_at | NA | SNCA | synuclein, alpha (non A4 component of amyloid precursor) | 6622 | ENSG00000145335 |
| 28 | 207827\_x\_at | NA | SNCA | synuclein, alpha (non A4 component of amyloid precursor) | 6622 | ENSG00000145335 |
| 29 | 236081\_at | NA | SNCA | synuclein, alpha (non A4 component of amyloid precursor) | 6622 | ENSG00000145335 |
| 30 | 204467\_s\_at | NA | SNCA | synuclein, alpha (non A4 component of amyloid precursor) | 6622 | ENSG00000145335 |
| 31 | 204466\_s\_at | NA | SNCA | synuclein, alpha (non A4 component of amyloid precursor) | 6622 | ENSG00000145335 |
| 32 | 227697\_at | NA | SOCS3 | suppressor of cytokine signaling 3 | 9021 | ENSG00000184557 |
| 33 | 201407\_s\_at | NA | PPP1CB | protein phosphatase 1, catalytic subunit, beta isozyme | 5500 | ENSG00000213639 |
| 34 | 203603\_s\_at | NA | ZEB2 | zinc finger E-box binding homeobox 2 | 9839 | ENSG00000169554 |
| 35 | 204794\_at | NA | DUSP2 | dual specificity phosphatase 2 | 1844 | ENSG00000158050 |
| 36 | 202375\_at | NA | SEC24D | SEC24 family, member D (S. cerevisiae) | 9871 | ENSG00000150961 |
| 37 | 226366\_at | NA | SHPRH | SNF2 histone linker PHD RING helicase, E3 ubiquitin protein ligase | 257218 | ENSG00000146414 |
| 38 | 214917\_at | NA | PRKAA1 | protein kinase, AMP-activated, alpha 1 catalytic subunit | 5562 | ENSG00000132356 |
| 39 | 1554309\_at | NA | EIF4G3 | eukaryotic translation initiation factor 4 gamma, 3 | 8672 | ENSG00000075151 |
| 40 | 220018\_at | NA | CBLL1 | Cbl proto-oncogene, E3 ubiquitin protein ligase-like 1 | 79872 | ENSG00000105879 |
| 41 | 210746\_s\_at | NA | EPB42 | erythrocyte membrane protein band 4.2 | 2038 | ENSG00000166947 |
| 42 | 202843\_at | NA | DNAJB9 | DnaJ (Hsp40) homolog, subfamily B, member 9 | 4189 | ENSG00000128590 |
| 43 | 202887\_s\_at | NA | DDIT4 | DNA-damage-inducible transcript 4 | 54541 | ENSG00000168209 |
| 44 | 208762\_at | NA | SUMO1 | SMT3 suppressor of mif two 3 homolog 1 (S. cerevisiae) | 7341 | ENSG00000116030 |
| 45 | 203358\_s\_at | NA | EZH2 | enhancer of zeste homolog 2 (Drosophila) | 2146 | ENSG00000106462 |
| 46 | 216929\_x\_at | NA | ABO | ABO blood group (transferase A, alpha 1-3-N-acetylgalactosaminyltransferase; transferase B, alpha 1-3-galactosyltransferase) | 28 | NULL |
| 47 | 203552\_at | NA | MAP4K5 | mitogen-activated protein kinase kinase kinase kinase 5 | 11183 | ENSG00000012983 |
| 48 | 204176\_at | NA | KLHL20 | kelch-like 20 (Drosophila) | 27252 | ENSG00000076321 |
| 49 | 1569136\_at | NA | MGAT4A | mannosyl (alpha-1,3-)-glycoprotein beta-1,4-N-acetylglucosaminyltransferase, isozyme A | 11320 | ENSG00000071073 |
| 50 | 222858\_s\_at | NA | DAPP1 | dual adaptor of phosphotyrosine and 3-phosphoinositides | 27071 | ENSG00000070190 |
| 51 | 212637\_s\_at | NA | WWP1 | WW domain containing E3 ubiquitin protein ligase 1 | 11059 | ENSG00000123124 |
| 52 | 208078\_s\_at | NA | SIK1 | salt-inducible kinase 1 | 150094 | ENSG00000142178 |
| 53 | 1555962\_at | NA | B3GNT7 | UDP-GlcNAc:betaGal beta-1,3-N-acetylglucosaminyltransferase 7 | 93010 | ENSG00000156966 |
| 54 | 202644\_s\_at | NA | TNFAIP3 | tumor necrosis factor, alpha-induced protein 3 | 7128 | ENSG00000118503 |
| 55 | 202203\_s\_at | NA | AMFR | autocrine motility factor receptor, E3 ubiquitin protein ligase | 267 | ENSG00000159461 |
| 56 | 231863\_at | NA | ING3 | inhibitor of growth family, member 3 | 54556 | ENSG00000071243 |
| 57 | 223266\_at | NA | STRADB | STE20-related kinase adaptor beta | 55437 | ENSG00000082146 |
| 58 | 1555878\_at | NA | RPS24 | ribosomal protein S24 | 6229 | ENSG00000138326 |
| 59 | 211560\_s\_at | NA | ALAS2 | aminolevulinate, delta-, synthase 2 | 212 | ENSG00000158578 |
| 60 | 202933\_s\_at | NA | YES1 | v-yes-1 Yamaguchi sarcoma viral oncogene homolog 1 | 7525 | ENSG00000176105 |
| 61 | 204995\_at | NA | CDK5R1 | cyclin-dependent kinase 5, regulatory subunit 1 (p35) | 8851 | ENSG00000176749 |
| 62 | 1557257\_at | NA | BCL10 | B-cell CLL/lymphoma 10 | 8915 | ENSG00000142867 |
| 63 | 218411\_s\_at | NA | MBIP | MAP3K12 binding inhibitory protein 1 | 51562 | ENSG00000151332 |
| 64 | 1559975\_at | NA | BTG1 | B-cell translocation gene 1, anti-proliferative | 694 | ENSG00000133639 |
| 65 | 1555476\_at | NA | IREB2 | iron-responsive element binding protein 2 | 3658 | ENSG00000136381 |
| 66 | 239143\_x\_at | NA | RNF138 | ring finger protein 138, E3 ubiquitin protein ligase | 51444 | ENSG00000134758 |
| 67 | 202232\_s\_at | NA | EIF3M | eukaryotic translation initiation factor 3, subunit M | 10480 | ENSG00000149100 |
| 68 | 205767\_at | NA | EREG | epiregulin | 2069 | ENSG00000124882 |
| 69 | 219304\_s\_at | NA | PDGFD | platelet derived growth factor D | 80310 | ENSG00000170962 |
| 70 | 230559\_x\_at | NA | FGD4 | FYVE, RhoGEF and PH domain containing 4 | 121512 | ENSG00000139132 |
| 71 | 212633\_at | NA | UFL1 | UFM1-specific ligase 1 | 23376 | ENSG00000014123 |
| 72 | 207300\_s\_at | NA | F7 | coagulation factor VII (serum prothrombin conversion accelerator) | 2155 | ENSG00000057593 |
| 73 | 232044\_at | NA | RBBP6 | retinoblastoma binding protein 6 | 5930 | ENSG00000122257 |

  
  

| **Database:biological process      &nbspName:single-organism metabolic process      &nbspID:GO:0044710** | | | | | | |
| --- | --- | --- | --- | --- | --- | --- |
| C=8718; O=137; E=107.14; R=1.28; rawP=3.79e-07; adjP=0.0001 | | | | | | |
| Index | UserID | Value | Gene Symbol | Gene Name | EntrezGene | Ensembl |
| 1 | 228106\_at | NA | DCAF16 | DDB1 and CUL4 associated factor 16 | 54876 | ENSG00000163257 |
| 2 | 219312\_s\_at | NA | ZBTB10 | zinc finger and BTB domain containing 10 | 65986 | ENSG00000205189 |
| 3 | 227449\_at | NA | EPHA4 | EPH receptor A4 | 2043 | ENSG00000116106 |
| 4 | 201016\_at | NA | EIF1AX | eukaryotic translation initiation factor 1A, X-linked | 1964 | ENSG00000173674 |
| 5 | 235300\_x\_at | NA | RCHY1 | ring finger and CHY zinc finger domain containing 1, E3 ubiquitin protein ligase | 25898 | ENSG00000163743 |
| 6 | 207078\_at | NA | MED6 | mediator complex subunit 6 | 10001 | ENSG00000133997 |
| 7 | 202660\_at | NA | ITPR2 | inositol 1,4,5-trisphosphate receptor, type 2 | 3709 | ENSG00000123104 |
| 8 | 202219\_at | NA | SLC6A8 | solute carrier family 6 (neurotransmitter transporter, creatine), member 8 | 6535 | ENSG00000130821 |
| 9 | 1558692\_at | NA | C1orf85 | chromosome 1 open reading frame 85 | 112770 | ENSG00000198715 |
| 10 | 203203\_s\_at | NA | KRR1 | KRR1, small subunit (SSU) processome component, homolog (yeast) | 11103 | ENSG00000111615 |
| 11 | 202314\_at | NA | CYP51A1 | cytochrome P450, family 51, subfamily A, polypeptide 1 | 1595 | ENSG00000001630 |
| 12 | 226680\_at | NA | IKZF5 | IKAROS family zinc finger 5 (Pegasus) | 64376 | ENSG00000095574 |
| 13 | 222243\_s\_at | NA | TOB2 | transducer of ERBB2, 2 | 10766 | ENSG00000183864 |
| 14 | 238020\_at | NA | PSMC2 | proteasome (prosome, macropain) 26S subunit, ATPase, 2 | 5701 | ENSG00000161057 |
| 15 | 213459\_at | NA | RPL37A | ribosomal protein L37a | 6168 | ENSG00000197756 |
| 16 | 205191\_at | NA | RP2 | retinitis pigmentosa 2 (X-linked recessive) | 6102 | ENSG00000102218 |
| 17 | 201437\_s\_at | NA | EIF4E | eukaryotic translation initiation factor 4E | 1977 | ENSG00000151247 |
| 18 | 232068\_s\_at | NA | TLR4 | toll-like receptor 4 | 7099 | ENSG00000136869 |
| 19 | 218871\_x\_at | NA | CSGALNACT2 | chondroitin sulfate N-acetylgalactosaminyltransferase 2 | 55454 | ENSG00000169826 |
| 20 | 204299\_at | NA | SRSF10 | serine/arginine-rich splicing factor 10 | 10772 | ENSG00000188529 |
| 21 | 230170\_at | NA | OSM | oncostatin M | 5008 | ENSG00000099985 |
| 22 | 229943\_at | NA | TRIM13 | tripartite motif containing 13 | 10206 | ENSG00000204977 |
| 23 | 227900\_at | NA | CBLB | Cbl proto-oncogene, E3 ubiquitin protein ligase B | 868 | ENSG00000114423 |
| 24 | 222848\_at | NA | CENPK | centromere protein K | 64105 | ENSG00000123219 |
| 25 | 205283\_at | NA | FKTN | fukutin | 2218 | ENSG00000106692 |
| 26 | 223200\_s\_at | NA | LSG1 | large subunit GTPase 1 homolog (S. cerevisiae) | 55341 | ENSG00000041802 |
| 27 | 218401\_s\_at | NA | ZNF281 | zinc finger protein 281 | 23528 | ENSG00000162702 |
| 28 | 228785\_at | NA | ZNF281 | zinc finger protein 281 | 23528 | ENSG00000162702 |
| 29 | 202861\_at | NA | PER1 | period homolog 1 (Drosophila) | 5187 | ENSG00000179094 |
| 30 | 205900\_at | NA | KRT1 | keratin 1 | 3848 | ENSG00000167768 |
| 31 | 201088\_at | NA | KPNA2 | karyopherin alpha 2 (RAG cohort 1, importin alpha 1) | 3838 | ENSG00000182481 |
| 32 | 229431\_at | NA | RFXAP | regulatory factor X-associated protein | 5994 | ENSG00000133111 |
| 33 | 36711\_at | NA | MAFF | v-maf musculoaponeurotic fibrosarcoma oncogene homolog F (avian) | 23764 | ENSG00000185022 |
| 34 | 212847\_at | NA | FUBP1 | far upstream element (FUSE) binding protein 1 | 8880 | ENSG00000162613 |
| 35 | 227391\_x\_at | NA | LRRFIP1 | leucine rich repeat (in FLII) interacting protein 1 | 9208 | ENSG00000124831 |
| 36 | 222876\_s\_at | NA | ADAP2 | ArfGAP with dual PH domains 2 | 55803 | ENSG00000184060 |
| 37 | 202375\_at | NA | SEC24D | SEC24 family, member D (S. cerevisiae) | 9871 | ENSG00000150961 |
| 38 | 214917\_at | NA | PRKAA1 | protein kinase, AMP-activated, alpha 1 catalytic subunit | 5562 | ENSG00000132356 |
| 39 | 204614\_at | NA | SERPINB2 | serpin peptidase inhibitor, clade B (ovalbumin), member 2 | 5055 | ENSG00000197632 |
| 40 | 217739\_s\_at | NA | NAMPT | nicotinamide phosphoribosyltransferase | 10135 | ENSG00000105835 |
| 41 | 202887\_s\_at | NA | DDIT4 | DNA-damage-inducible transcript 4 | 54541 | ENSG00000168209 |
| 42 | 202904\_s\_at | NA | LSM5 | LSM5 homolog, U6 small nuclear RNA associated (S. cerevisiae) | 23658 | ENSG00000106355 |
| 43 | 203552\_at | NA | MAP4K5 | mitogen-activated protein kinase kinase kinase kinase 5 | 11183 | ENSG00000012983 |
| 44 | 222765\_x\_at | NA | ESF1 | ESF1, nucleolar pre-rRNA processing protein, homolog (S. cerevisiae) | 51575 | ENSG00000089048 |
| 45 | 208078\_s\_at | NA | SIK1 | salt-inducible kinase 1 | 150094 | ENSG00000142178 |
| 46 | 1555962\_at | NA | B3GNT7 | UDP-GlcNAc:betaGal beta-1,3-N-acetylglucosaminyltransferase 7 | 93010 | ENSG00000156966 |
| 47 | 1555274\_a\_at | NA | EPT1 | ethanolaminephosphotransferase 1 (CDP-ethanolamine-specific) | 85465 | ENSG00000138018 |
| 48 | 202498\_s\_at | NA | SLC2A3 | solute carrier family 2 (facilitated glucose transporter), member 3 | 6515 | ENSG00000059804 |
| 49 | 226279\_at | NA | PRSS23 | protease, serine, 23 | 11098 | ENSG00000150687 |
| 50 | 238199\_x\_at | NA | COX3 | cytochrome c oxidase III | 4514 | NULL |
| 51 | 1555878\_at | NA | RPS24 | ribosomal protein S24 | 6229 | ENSG00000138326 |
| 52 | 223266\_at | NA | STRADB | STE20-related kinase adaptor beta | 55437 | ENSG00000082146 |
| 53 | 231863\_at | NA | ING3 | inhibitor of growth family, member 3 | 54556 | ENSG00000071243 |
| 54 | 211560\_s\_at | NA | ALAS2 | aminolevulinate, delta-, synthase 2 | 212 | ENSG00000158578 |
| 55 | 205063\_at | NA | GEMIN2 | gem (nuclear organelle) associated protein 2 | 8487 | ENSG00000092208 |
| 56 | 203634\_s\_at | NA | CPT1A | carnitine palmitoyltransferase 1A (liver) | 1374 | ENSG00000110090 |
| 57 | 204995\_at | NA | CDK5R1 | cyclin-dependent kinase 5, regulatory subunit 1 (p35) | 8851 | ENSG00000176749 |
| 58 | 228063\_s\_at | NA | NAP1L5 | nucleosome assembly protein 1-like 5 | 266812 | ENSG00000177432 |
| 59 | 228062\_at | NA | NAP1L5 | nucleosome assembly protein 1-like 5 | 266812 | ENSG00000177432 |
| 60 | 218411\_s\_at | NA | MBIP | MAP3K12 binding inhibitory protein 1 | 51562 | ENSG00000151332 |
| 61 | 203574\_at | NA | NFIL3 | nuclear factor, interleukin 3 regulated | 4783 | ENSG00000165030 |
| 62 | 205767\_at | NA | EREG | epiregulin | 2069 | ENSG00000124882 |
| 63 | 202232\_s\_at | NA | EIF3M | eukaryotic translation initiation factor 3, subunit M | 10480 | ENSG00000149100 |
| 64 | 230559\_x\_at | NA | FGD4 | FYVE, RhoGEF and PH domain containing 4 | 121512 | ENSG00000139132 |
| 65 | 238614\_x\_at | NA | ZNF430 | zinc finger protein 430 | 80264 | ENSG00000118620 |
| 66 | 216248\_s\_at | NA | NR4A2 | nuclear receptor subfamily 4, group A, member 2 | 4929 | ENSG00000153234 |
| 67 | 204621\_s\_at | NA | NR4A2 | nuclear receptor subfamily 4, group A, member 2 | 4929 | ENSG00000153234 |
| 68 | 204622\_x\_at | NA | NR4A2 | nuclear receptor subfamily 4, group A, member 2 | 4929 | ENSG00000153234 |
| 69 | 232044\_at | NA | RBBP6 | retinoblastoma binding protein 6 | 5930 | ENSG00000122257 |
| 70 | 200608\_s\_at | NA | RAD21 | RAD21 homolog (S. pombe) | 5885 | ENSG00000164754 |
| 71 | 202221\_s\_at | NA | EP300 | E1A binding protein p300 | 2033 | ENSG00000100393 |
| 72 | 206919\_at | NA | ELK4 | ELK4, ETS-domain protein (SRF accessory protein 1) | 2005 | ENSG00000158711 |
| 73 | 208121\_s\_at | NA | PTPRO | protein tyrosine phosphatase, receptor type, O | 5800 | ENSG00000151490 |
| 74 | 201304\_at | NA | NDUFA5 | NADH dehydrogenase (ubiquinone) 1 alpha subcomplex, 5, 13kDa | 4698 | ENSG00000128609 |
| 75 | 203821\_at | NA | HBEGF | heparin-binding EGF-like growth factor | 1839 | ENSG00000113070 |
| 76 | 225760\_at | NA | MYSM1 | Myb-like, SWIRM and MPN domains 1 | 114803 | ENSG00000162601 |
| 77 | 222310\_at | NA | SCAF4 | SR-related CTD-associated factor 4 | 57466 | ENSG00000156304 |
| 78 | 213725\_x\_at | NA | XYLT1 | xylosyltransferase I | 64131 | ENSG00000103489 |
| 79 | 224453\_s\_at | NA | ETNK1 | ethanolamine kinase 1 | 55500 | ENSG00000139163 |
| 80 | 225290\_at | NA | ETNK1 | ethanolamine kinase 1 | 55500 | ENSG00000139163 |
| 81 | 202979\_s\_at | NA | CREBZF | CREB/ATF bZIP transcription factor | 58487 | ENSG00000137504 |
| 82 | 204187\_at | NA | GMPR | guanosine monophosphate reductase | 2766 | ENSG00000137198 |
| 83 | 233019\_at | NA | CNOT7 | CCR4-NOT transcription complex, subunit 7 | 29883 | ENSG00000198791 |
| 84 | 213225\_at | NA | PPM1B | protein phosphatase, Mg2+/Mn2+ dependent, 1B | 5495 | ENSG00000138032 |
| 85 | 200602\_at | NA | APP | amyloid beta (A4) precursor protein | 351 | ENSG00000142192 |
| 86 | 225916\_at | NA | ZNF131 | zinc finger protein 131 | 7690 | ENSG00000172262 |
| 87 | 214741\_at | NA | ZNF131 | zinc finger protein 131 | 7690 | ENSG00000172262 |
| 88 | 201177\_s\_at | NA | UBA2 | ubiquitin-like modifier activating enzyme 2 | 10054 | ENSG00000126261 |
| 89 | 227375\_at | NA | ANKRD13C | ankyrin repeat domain 13C | 81573 | ENSG00000118454 |
| 90 | 202422\_s\_at | NA | ACSL4 | acyl-CoA synthetase long-chain family member 4 | 2182 | ENSG00000068366 |
| 91 | 214590\_s\_at | NA | UBE2D1 | ubiquitin-conjugating enzyme E2D 1 | 7321 | ENSG00000072401 |
| 92 | 221768\_at | NA | SFPQ | splicing factor proline/glutamine-rich | 6421 | ENSG00000116560 |
| 93 | 211698\_at | NA | EID1 | EP300 interacting inhibitor of differentiation 1 | 23741 | ENSG00000255302 |
| 94 | 202464\_s\_at | NA | PFKFB3 | 6-phosphofructo-2-kinase/fructose-2,6-biphosphatase 3 | 5209 | ENSG00000170525 |
| 95 | 236539\_at | NA | PTPN22 | protein tyrosine phosphatase, non-receptor type 22 (lymphoid) | 26191 | ENSG00000134242 |
| 96 | 226472\_at | NA | PPIL4 | peptidylprolyl isomerase (cyclophilin)-like 4 | 85313 | ENSG00000131013 |
| 97 | 227697\_at | NA | SOCS3 | suppressor of cytokine signaling 3 | 9021 | ENSG00000184557 |
| 98 | 211546\_x\_at | NA | SNCA | synuclein, alpha (non A4 component of amyloid precursor) | 6622 | ENSG00000145335 |
| 99 | 207827\_x\_at | NA | SNCA | synuclein, alpha (non A4 component of amyloid precursor) | 6622 | ENSG00000145335 |
| 100 | 236081\_at | NA | SNCA | synuclein, alpha (non A4 component of amyloid precursor) | 6622 | ENSG00000145335 |
| 101 | 204467\_s\_at | NA | SNCA | synuclein, alpha (non A4 component of amyloid precursor) | 6622 | ENSG00000145335 |
| 102 | 204466\_s\_at | NA | SNCA | synuclein, alpha (non A4 component of amyloid precursor) | 6622 | ENSG00000145335 |
| 103 | 213998\_s\_at | NA | DDX17 | DEAD (Asp-Glu-Ala-Asp) box helicase 17 | 10521 | ENSG00000100201 |
| 104 | 208719\_s\_at | NA | DDX17 | DEAD (Asp-Glu-Ala-Asp) box helicase 17 | 10521 | ENSG00000100201 |
| 105 | 208151\_x\_at | NA | DDX17 | DEAD (Asp-Glu-Ala-Asp) box helicase 17 | 10521 | ENSG00000100201 |
| 106 | 224009\_x\_at | NA | DHRS9 | dehydrogenase/reductase (SDR family) member 9 | 10170 | ENSG00000073737 |
| 107 | 223952\_x\_at | NA | DHRS9 | dehydrogenase/reductase (SDR family) member 9 | 10170 | ENSG00000073737 |
| 108 | 213524\_s\_at | NA | G0S2 | G0/G1switch 2 | 50486 | ENSG00000123689 |
| 109 | 201407\_s\_at | NA | PPP1CB | protein phosphatase 1, catalytic subunit, beta isozyme | 5500 | ENSG00000213639 |
| 110 | 1553581\_s\_at | NA | SREK1IP1 | SREK1-interacting protein 1 | 285672 | ENSG00000153006 |
| 111 | 223649\_s\_at | NA | SLC25A39 | solute carrier family 25, member 39 | 51629 | ENSG00000013306 |
| 112 | 203603\_s\_at | NA | ZEB2 | zinc finger E-box binding homeobox 2 | 9839 | ENSG00000169554 |
| 113 | 204794\_at | NA | DUSP2 | dual specificity phosphatase 2 | 1844 | ENSG00000158050 |
| 114 | 236140\_at | NA | GCLM | glutamate-cysteine ligase, modifier subunit | 2730 | ENSG00000023909 |
| 115 | 235412\_at | NA | ARHGEF7 | Rho guanine nucleotide exchange factor (GEF) 7 | 8874 | ENSG00000102606 |
| 116 | 206108\_s\_at | NA | SRSF6 | serine/arginine-rich splicing factor 6 | 6431 | ENSG00000124193 |
| 117 | 226366\_at | NA | SHPRH | SNF2 histone linker PHD RING helicase, E3 ubiquitin protein ligase | 257218 | ENSG00000146414 |
| 118 | 212989\_at | NA | SGMS1 | sphingomyelin synthase 1 | 259230 | ENSG00000198964 |
| 119 | 213786\_at | NA | TAX1BP1 | Tax1 (human T-cell leukemia virus type I) binding protein 1 | 8887 | ENSG00000106052 |
| 120 | 1554309\_at | NA | EIF4G3 | eukaryotic translation initiation factor 4 gamma, 3 | 8672 | ENSG00000075151 |
| 121 | 220018\_at | NA | CBLL1 | Cbl proto-oncogene, E3 ubiquitin protein ligase-like 1 | 79872 | ENSG00000105879 |
| 122 | 210746\_s\_at | NA | EPB42 | erythrocyte membrane protein band 4.2 | 2038 | ENSG00000166947 |
| 123 | 202843\_at | NA | DNAJB9 | DnaJ (Hsp40) homolog, subfamily B, member 9 | 4189 | ENSG00000128590 |
| 124 | 216834\_at | NA | RGS1 | regulator of G-protein signaling 1 | 5996 | ENSG00000090104 |
| 125 | 202988\_s\_at | NA | RGS1 | regulator of G-protein signaling 1 | 5996 | ENSG00000090104 |
| 126 | 208762\_at | NA | SUMO1 | SMT3 suppressor of mif two 3 homolog 1 (S. cerevisiae) | 7341 | ENSG00000116030 |
| 127 | 203358\_s\_at | NA | EZH2 | enhancer of zeste homolog 2 (Drosophila) | 2146 | ENSG00000106462 |
| 128 | 1558233\_s\_at | NA | ATF1 | activating transcription factor 1 | 466 | ENSG00000123268 |
| 129 | 216929\_x\_at | NA | ABO | ABO blood group (transferase A, alpha 1-3-N-acetylgalactosaminyltransferase; transferase B, alpha 1-3-galactosyltransferase) | 28 | NULL |
| 130 | 204176\_at | NA | KLHL20 | kelch-like 20 (Drosophila) | 27252 | ENSG00000076321 |
| 131 | 1569136\_at | NA | MGAT4A | mannosyl (alpha-1,3-)-glycoprotein beta-1,4-N-acetylglucosaminyltransferase, isozyme A | 11320 | ENSG00000071073 |
| 132 | 222858\_s\_at | NA | DAPP1 | dual adaptor of phosphotyrosine and 3-phosphoinositides | 27071 | ENSG00000070190 |
| 133 | 212637\_s\_at | NA | WWP1 | WW domain containing E3 ubiquitin protein ligase 1 | 11059 | ENSG00000123124 |
| 134 | 203543\_s\_at | NA | KLF9 | Kruppel-like factor 9 | 687 | ENSG00000119138 |
| 135 | 212579\_at | NA | SMCHD1 | structural maintenance of chromosomes flexible hinge domain containing 1 | 23347 | ENSG00000101596 |
| 136 | 215716\_s\_at | NA | ATP2B1 | ATPase, Ca++ transporting, plasma membrane 1 | 490 | ENSG00000070961 |
| 137 | 202644\_s\_at | NA | TNFAIP3 | tumor necrosis factor, alpha-induced protein 3 | 7128 | ENSG00000118503 |
| 138 | 202203\_s\_at | NA | AMFR | autocrine motility factor receptor, E3 ubiquitin protein ligase | 267 | ENSG00000159461 |
| 139 | 242960\_at | NA | EPC2 | enhancer of polycomb homolog 2 (Drosophila) | 26122 | ENSG00000135999 |
| 140 | 207794\_at | NA | CCR2 | chemokine (C-C motif) receptor 2 | 729230 | ENSG00000121807 |
| 141 | 202933\_s\_at | NA | YES1 | v-yes-1 Yamaguchi sarcoma viral oncogene homolog 1 | 7525 | ENSG00000176105 |
| 142 | 222317\_at | NA | PDE3B | phosphodiesterase 3B, cGMP-inhibited | 5140 | ENSG00000152270 |
| 143 | 1557257\_at | NA | BCL10 | B-cell CLL/lymphoma 10 | 8915 | ENSG00000142867 |
| 144 | 201091\_s\_at | NA | CBX3 | chromobox homolog 3 | 11335 | ENSG00000122565 |
| 145 | 1559975\_at | NA | BTG1 | B-cell translocation gene 1, anti-proliferative | 694 | ENSG00000133639 |
| 146 | 1555476\_at | NA | IREB2 | iron-responsive element binding protein 2 | 3658 | ENSG00000136381 |
| 147 | 239143\_x\_at | NA | RNF138 | ring finger protein 138, E3 ubiquitin protein ligase | 51444 | ENSG00000134758 |
| 148 | 214012\_at | NA | ERAP1 | endoplasmic reticulum aminopeptidase 1 | 51752 | ENSG00000164307 |
| 149 | 219304\_s\_at | NA | PDGFD | platelet derived growth factor D | 80310 | ENSG00000170962 |
| 150 | 212633\_at | NA | UFL1 | UFM1-specific ligase 1 | 23376 | ENSG00000014123 |
| 151 | 207300\_s\_at | NA | F7 | coagulation factor VII (serum prothrombin conversion accelerator) | 2155 | ENSG00000057593 |

  
  

| **Database:biological process      &nbspName:cellular protein modification process      &nbspID:GO:0006464** | | | | | | |
| --- | --- | --- | --- | --- | --- | --- |
| C=2409; O=57; E=29.60; R=1.93; rawP=2.39e-07; adjP=0.0001 | | | | | | |
| Index | UserID | Value | Gene Symbol | Gene Name | EntrezGene | Ensembl |
| 1 | 206919\_at | NA | ELK4 | ELK4, ETS-domain protein (SRF accessory protein 1) | 2005 | ENSG00000158711 |
| 2 | 202221\_s\_at | NA | EP300 | E1A binding protein p300 | 2033 | ENSG00000100393 |
| 3 | 204794\_at | NA | DUSP2 | dual specificity phosphatase 2 | 1844 | ENSG00000158050 |
| 4 | 208121\_s\_at | NA | PTPRO | protein tyrosine phosphatase, receptor type, O | 5800 | ENSG00000151490 |
| 5 | 228106\_at | NA | DCAF16 | DDB1 and CUL4 associated factor 16 | 54876 | ENSG00000163257 |
| 6 | 225760\_at | NA | MYSM1 | Myb-like, SWIRM and MPN domains 1 | 114803 | ENSG00000162601 |
| 7 | 202375\_at | NA | SEC24D | SEC24 family, member D (S. cerevisiae) | 9871 | ENSG00000150961 |
| 8 | 226366\_at | NA | SHPRH | SNF2 histone linker PHD RING helicase, E3 ubiquitin protein ligase | 257218 | ENSG00000146414 |
| 9 | 214917\_at | NA | PRKAA1 | protein kinase, AMP-activated, alpha 1 catalytic subunit | 5562 | ENSG00000132356 |
| 10 | 227449\_at | NA | EPHA4 | EPH receptor A4 | 2043 | ENSG00000116106 |
| 11 | 220018\_at | NA | CBLL1 | Cbl proto-oncogene, E3 ubiquitin protein ligase-like 1 | 79872 | ENSG00000105879 |
| 12 | 1554309\_at | NA | EIF4G3 | eukaryotic translation initiation factor 4 gamma, 3 | 8672 | ENSG00000075151 |
| 13 | 235300\_x\_at | NA | RCHY1 | ring finger and CHY zinc finger domain containing 1, E3 ubiquitin protein ligase | 25898 | ENSG00000163743 |
| 14 | 213225\_at | NA | PPM1B | protein phosphatase, Mg2+/Mn2+ dependent, 1B | 5495 | ENSG00000138032 |
| 15 | 210746\_s\_at | NA | EPB42 | erythrocyte membrane protein band 4.2 | 2038 | ENSG00000166947 |
| 16 | 200602\_at | NA | APP | amyloid beta (A4) precursor protein | 351 | ENSG00000142192 |
| 17 | 202843\_at | NA | DNAJB9 | DnaJ (Hsp40) homolog, subfamily B, member 9 | 4189 | ENSG00000128590 |
| 18 | 203358\_s\_at | NA | EZH2 | enhancer of zeste homolog 2 (Drosophila) | 2146 | ENSG00000106462 |
| 19 | 208762\_at | NA | SUMO1 | SMT3 suppressor of mif two 3 homolog 1 (S. cerevisiae) | 7341 | ENSG00000116030 |
| 20 | 202887\_s\_at | NA | DDIT4 | DNA-damage-inducible transcript 4 | 54541 | ENSG00000168209 |
| 21 | 216929\_x\_at | NA | ABO | ABO blood group (transferase A, alpha 1-3-N-acetylgalactosaminyltransferase; transferase B, alpha 1-3-galactosyltransferase) | 28 | NULL |
| 22 | 204176\_at | NA | KLHL20 | kelch-like 20 (Drosophila) | 27252 | ENSG00000076321 |
| 23 | 203552\_at | NA | MAP4K5 | mitogen-activated protein kinase kinase kinase kinase 5 | 11183 | ENSG00000012983 |
| 24 | 201177\_s\_at | NA | UBA2 | ubiquitin-like modifier activating enzyme 2 | 10054 | ENSG00000126261 |
| 25 | 1569136\_at | NA | MGAT4A | mannosyl (alpha-1,3-)-glycoprotein beta-1,4-N-acetylglucosaminyltransferase, isozyme A | 11320 | ENSG00000071073 |
| 26 | 238020\_at | NA | PSMC2 | proteasome (prosome, macropain) 26S subunit, ATPase, 2 | 5701 | ENSG00000161057 |
| 27 | 222858\_s\_at | NA | DAPP1 | dual adaptor of phosphotyrosine and 3-phosphoinositides | 27071 | ENSG00000070190 |
| 28 | 212637\_s\_at | NA | WWP1 | WW domain containing E3 ubiquitin protein ligase 1 | 11059 | ENSG00000123124 |
| 29 | 214590\_s\_at | NA | UBE2D1 | ubiquitin-conjugating enzyme E2D 1 | 7321 | ENSG00000072401 |
| 30 | 208078\_s\_at | NA | SIK1 | salt-inducible kinase 1 | 150094 | ENSG00000142178 |
| 31 | 232068\_s\_at | NA | TLR4 | toll-like receptor 4 | 7099 | ENSG00000136869 |
| 32 | 230170\_at | NA | OSM | oncostatin M | 5008 | ENSG00000099985 |
| 33 | 1555962\_at | NA | B3GNT7 | UDP-GlcNAc:betaGal beta-1,3-N-acetylglucosaminyltransferase 7 | 93010 | ENSG00000156966 |
| 34 | 229943\_at | NA | TRIM13 | tripartite motif containing 13 | 10206 | ENSG00000204977 |
| 35 | 202644\_s\_at | NA | TNFAIP3 | tumor necrosis factor, alpha-induced protein 3 | 7128 | ENSG00000118503 |
| 36 | 211698\_at | NA | EID1 | EP300 interacting inhibitor of differentiation 1 | 23741 | ENSG00000255302 |
| 37 | 202203\_s\_at | NA | AMFR | autocrine motility factor receptor, E3 ubiquitin protein ligase | 267 | ENSG00000159461 |
| 38 | 223266\_at | NA | STRADB | STE20-related kinase adaptor beta | 55437 | ENSG00000082146 |
| 39 | 231863\_at | NA | ING3 | inhibitor of growth family, member 3 | 54556 | ENSG00000071243 |
| 40 | 202933\_s\_at | NA | YES1 | v-yes-1 Yamaguchi sarcoma viral oncogene homolog 1 | 7525 | ENSG00000176105 |
| 41 | 205283\_at | NA | FKTN | fukutin | 2218 | ENSG00000106692 |
| 42 | 236539\_at | NA | PTPN22 | protein tyrosine phosphatase, non-receptor type 22 (lymphoid) | 26191 | ENSG00000134242 |
| 43 | 204995\_at | NA | CDK5R1 | cyclin-dependent kinase 5, regulatory subunit 1 (p35) | 8851 | ENSG00000176749 |
| 44 | 1557257\_at | NA | BCL10 | B-cell CLL/lymphoma 10 | 8915 | ENSG00000142867 |
| 45 | 211546\_x\_at | NA | SNCA | synuclein, alpha (non A4 component of amyloid precursor) | 6622 | ENSG00000145335 |
| 46 | 207827\_x\_at | NA | SNCA | synuclein, alpha (non A4 component of amyloid precursor) | 6622 | ENSG00000145335 |
| 47 | 236081\_at | NA | SNCA | synuclein, alpha (non A4 component of amyloid precursor) | 6622 | ENSG00000145335 |
| 48 | 204467\_s\_at | NA | SNCA | synuclein, alpha (non A4 component of amyloid precursor) | 6622 | ENSG00000145335 |
| 49 | 204466\_s\_at | NA | SNCA | synuclein, alpha (non A4 component of amyloid precursor) | 6622 | ENSG00000145335 |
| 50 | 227697\_at | NA | SOCS3 | suppressor of cytokine signaling 3 | 9021 | ENSG00000184557 |
| 51 | 1559975\_at | NA | BTG1 | B-cell translocation gene 1, anti-proliferative | 694 | ENSG00000133639 |
| 52 | 218411\_s\_at | NA | MBIP | MAP3K12 binding inhibitory protein 1 | 51562 | ENSG00000151332 |
| 53 | 239143\_x\_at | NA | RNF138 | ring finger protein 138, E3 ubiquitin protein ligase | 51444 | ENSG00000134758 |
| 54 | 205767\_at | NA | EREG | epiregulin | 2069 | ENSG00000124882 |
| 55 | 219304\_s\_at | NA | PDGFD | platelet derived growth factor D | 80310 | ENSG00000170962 |
| 56 | 201407\_s\_at | NA | PPP1CB | protein phosphatase 1, catalytic subunit, beta isozyme | 5500 | ENSG00000213639 |
| 57 | 230559\_x\_at | NA | FGD4 | FYVE, RhoGEF and PH domain containing 4 | 121512 | ENSG00000139132 |
| 58 | 212633\_at | NA | UFL1 | UFM1-specific ligase 1 | 23376 | ENSG00000014123 |
| 59 | 207300\_s\_at | NA | F7 | coagulation factor VII (serum prothrombin conversion accelerator) | 2155 | ENSG00000057593 |
| 60 | 232044\_at | NA | RBBP6 | retinoblastoma binding protein 6 | 5930 | ENSG00000122257 |
| 61 | 203603\_s\_at | NA | ZEB2 | zinc finger E-box binding homeobox 2 | 9839 | ENSG00000169554 |

  
  

| **Database:biological process      &nbspName:macromolecule modification      &nbspID:GO:0043412** | | | | | | |
| --- | --- | --- | --- | --- | --- | --- |
| C=2501; O=57; E=30.74; R=1.85; rawP=8.57e-07; adjP=0.0002 | | | | | | |
| Index | UserID | Value | Gene Symbol | Gene Name | EntrezGene | Ensembl |
| 1 | 206919\_at | NA | ELK4 | ELK4, ETS-domain protein (SRF accessory protein 1) | 2005 | ENSG00000158711 |
| 2 | 202221\_s\_at | NA | EP300 | E1A binding protein p300 | 2033 | ENSG00000100393 |
| 3 | 204794\_at | NA | DUSP2 | dual specificity phosphatase 2 | 1844 | ENSG00000158050 |
| 4 | 208121\_s\_at | NA | PTPRO | protein tyrosine phosphatase, receptor type, O | 5800 | ENSG00000151490 |
| 5 | 228106\_at | NA | DCAF16 | DDB1 and CUL4 associated factor 16 | 54876 | ENSG00000163257 |
| 6 | 225760\_at | NA | MYSM1 | Myb-like, SWIRM and MPN domains 1 | 114803 | ENSG00000162601 |
| 7 | 202375\_at | NA | SEC24D | SEC24 family, member D (S. cerevisiae) | 9871 | ENSG00000150961 |
| 8 | 226366\_at | NA | SHPRH | SNF2 histone linker PHD RING helicase, E3 ubiquitin protein ligase | 257218 | ENSG00000146414 |
| 9 | 214917\_at | NA | PRKAA1 | protein kinase, AMP-activated, alpha 1 catalytic subunit | 5562 | ENSG00000132356 |
| 10 | 227449\_at | NA | EPHA4 | EPH receptor A4 | 2043 | ENSG00000116106 |
| 11 | 220018\_at | NA | CBLL1 | Cbl proto-oncogene, E3 ubiquitin protein ligase-like 1 | 79872 | ENSG00000105879 |
| 12 | 1554309\_at | NA | EIF4G3 | eukaryotic translation initiation factor 4 gamma, 3 | 8672 | ENSG00000075151 |
| 13 | 235300\_x\_at | NA | RCHY1 | ring finger and CHY zinc finger domain containing 1, E3 ubiquitin protein ligase | 25898 | ENSG00000163743 |
| 14 | 213225\_at | NA | PPM1B | protein phosphatase, Mg2+/Mn2+ dependent, 1B | 5495 | ENSG00000138032 |
| 15 | 210746\_s\_at | NA | EPB42 | erythrocyte membrane protein band 4.2 | 2038 | ENSG00000166947 |
| 16 | 200602\_at | NA | APP | amyloid beta (A4) precursor protein | 351 | ENSG00000142192 |
| 17 | 202843\_at | NA | DNAJB9 | DnaJ (Hsp40) homolog, subfamily B, member 9 | 4189 | ENSG00000128590 |
| 18 | 203358\_s\_at | NA | EZH2 | enhancer of zeste homolog 2 (Drosophila) | 2146 | ENSG00000106462 |
| 19 | 208762\_at | NA | SUMO1 | SMT3 suppressor of mif two 3 homolog 1 (S. cerevisiae) | 7341 | ENSG00000116030 |
| 20 | 202887\_s\_at | NA | DDIT4 | DNA-damage-inducible transcript 4 | 54541 | ENSG00000168209 |
| 21 | 216929\_x\_at | NA | ABO | ABO blood group (transferase A, alpha 1-3-N-acetylgalactosaminyltransferase; transferase B, alpha 1-3-galactosyltransferase) | 28 | NULL |
| 22 | 204176\_at | NA | KLHL20 | kelch-like 20 (Drosophila) | 27252 | ENSG00000076321 |
| 23 | 203552\_at | NA | MAP4K5 | mitogen-activated protein kinase kinase kinase kinase 5 | 11183 | ENSG00000012983 |
| 24 | 201177\_s\_at | NA | UBA2 | ubiquitin-like modifier activating enzyme 2 | 10054 | ENSG00000126261 |
| 25 | 1569136\_at | NA | MGAT4A | mannosyl (alpha-1,3-)-glycoprotein beta-1,4-N-acetylglucosaminyltransferase, isozyme A | 11320 | ENSG00000071073 |
| 26 | 238020\_at | NA | PSMC2 | proteasome (prosome, macropain) 26S subunit, ATPase, 2 | 5701 | ENSG00000161057 |
| 27 | 222858\_s\_at | NA | DAPP1 | dual adaptor of phosphotyrosine and 3-phosphoinositides | 27071 | ENSG00000070190 |
| 28 | 212637\_s\_at | NA | WWP1 | WW domain containing E3 ubiquitin protein ligase 1 | 11059 | ENSG00000123124 |
| 29 | 214590\_s\_at | NA | UBE2D1 | ubiquitin-conjugating enzyme E2D 1 | 7321 | ENSG00000072401 |
| 30 | 208078\_s\_at | NA | SIK1 | salt-inducible kinase 1 | 150094 | ENSG00000142178 |
| 31 | 232068\_s\_at | NA | TLR4 | toll-like receptor 4 | 7099 | ENSG00000136869 |
| 32 | 230170\_at | NA | OSM | oncostatin M | 5008 | ENSG00000099985 |
| 33 | 1555962\_at | NA | B3GNT7 | UDP-GlcNAc:betaGal beta-1,3-N-acetylglucosaminyltransferase 7 | 93010 | ENSG00000156966 |
| 34 | 229943\_at | NA | TRIM13 | tripartite motif containing 13 | 10206 | ENSG00000204977 |
| 35 | 202644\_s\_at | NA | TNFAIP3 | tumor necrosis factor, alpha-induced protein 3 | 7128 | ENSG00000118503 |
| 36 | 211698\_at | NA | EID1 | EP300 interacting inhibitor of differentiation 1 | 23741 | ENSG00000255302 |
| 37 | 202203\_s\_at | NA | AMFR | autocrine motility factor receptor, E3 ubiquitin protein ligase | 267 | ENSG00000159461 |
| 38 | 223266\_at | NA | STRADB | STE20-related kinase adaptor beta | 55437 | ENSG00000082146 |
| 39 | 231863\_at | NA | ING3 | inhibitor of growth family, member 3 | 54556 | ENSG00000071243 |
| 40 | 202933\_s\_at | NA | YES1 | v-yes-1 Yamaguchi sarcoma viral oncogene homolog 1 | 7525 | ENSG00000176105 |
| 41 | 205283\_at | NA | FKTN | fukutin | 2218 | ENSG00000106692 |
| 42 | 236539\_at | NA | PTPN22 | protein tyrosine phosphatase, non-receptor type 22 (lymphoid) | 26191 | ENSG00000134242 |
| 43 | 204995\_at | NA | CDK5R1 | cyclin-dependent kinase 5, regulatory subunit 1 (p35) | 8851 | ENSG00000176749 |
| 44 | 1557257\_at | NA | BCL10 | B-cell CLL/lymphoma 10 | 8915 | ENSG00000142867 |
| 45 | 211546\_x\_at | NA | SNCA | synuclein, alpha (non A4 component of amyloid precursor) | 6622 | ENSG00000145335 |
| 46 | 207827\_x\_at | NA | SNCA | synuclein, alpha (non A4 component of amyloid precursor) | 6622 | ENSG00000145335 |
| 47 | 236081\_at | NA | SNCA | synuclein, alpha (non A4 component of amyloid precursor) | 6622 | ENSG00000145335 |
| 48 | 204467\_s\_at | NA | SNCA | synuclein, alpha (non A4 component of amyloid precursor) | 6622 | ENSG00000145335 |
| 49 | 204466\_s\_at | NA | SNCA | synuclein, alpha (non A4 component of amyloid precursor) | 6622 | ENSG00000145335 |
| 50 | 227697\_at | NA | SOCS3 | suppressor of cytokine signaling 3 | 9021 | ENSG00000184557 |
| 51 | 1559975\_at | NA | BTG1 | B-cell translocation gene 1, anti-proliferative | 694 | ENSG00000133639 |
| 52 | 218411\_s\_at | NA | MBIP | MAP3K12 binding inhibitory protein 1 | 51562 | ENSG00000151332 |
| 53 | 239143\_x\_at | NA | RNF138 | ring finger protein 138, E3 ubiquitin protein ligase | 51444 | ENSG00000134758 |
| 54 | 205767\_at | NA | EREG | epiregulin | 2069 | ENSG00000124882 |
| 55 | 219304\_s\_at | NA | PDGFD | platelet derived growth factor D | 80310 | ENSG00000170962 |
| 56 | 201407\_s\_at | NA | PPP1CB | protein phosphatase 1, catalytic subunit, beta isozyme | 5500 | ENSG00000213639 |
| 57 | 230559\_x\_at | NA | FGD4 | FYVE, RhoGEF and PH domain containing 4 | 121512 | ENSG00000139132 |
| 58 | 212633\_at | NA | UFL1 | UFM1-specific ligase 1 | 23376 | ENSG00000014123 |
| 59 | 207300\_s\_at | NA | F7 | coagulation factor VII (serum prothrombin conversion accelerator) | 2155 | ENSG00000057593 |
| 60 | 232044\_at | NA | RBBP6 | retinoblastoma binding protein 6 | 5930 | ENSG00000122257 |
| 61 | 203603\_s\_at | NA | ZEB2 | zinc finger E-box binding homeobox 2 | 9839 | ENSG00000169554 |

  
  

| **Database:biological process      &nbspName:protein metabolic process      &nbspID:GO:0019538** | | | | | | |
| --- | --- | --- | --- | --- | --- | --- |
| C=3730; O=75; E=45.84; R=1.64; rawP=9.45e-07; adjP=0.0002 | | | | | | |
| Index | UserID | Value | Gene Symbol | Gene Name | EntrezGene | Ensembl |
| 1 | 206919\_at | NA | ELK4 | ELK4, ETS-domain protein (SRF accessory protein 1) | 2005 | ENSG00000158711 |
| 2 | 202221\_s\_at | NA | EP300 | E1A binding protein p300 | 2033 | ENSG00000100393 |
| 3 | 208121\_s\_at | NA | PTPRO | protein tyrosine phosphatase, receptor type, O | 5800 | ENSG00000151490 |
| 4 | 228106\_at | NA | DCAF16 | DDB1 and CUL4 associated factor 16 | 54876 | ENSG00000163257 |
| 5 | 203821\_at | NA | HBEGF | heparin-binding EGF-like growth factor | 1839 | ENSG00000113070 |
| 6 | 225760\_at | NA | MYSM1 | Myb-like, SWIRM and MPN domains 1 | 114803 | ENSG00000162601 |
| 7 | 213725\_x\_at | NA | XYLT1 | xylosyltransferase I | 64131 | ENSG00000103489 |
| 8 | 227449\_at | NA | EPHA4 | EPH receptor A4 | 2043 | ENSG00000116106 |
| 9 | 201016\_at | NA | EIF1AX | eukaryotic translation initiation factor 1A, X-linked | 1964 | ENSG00000173674 |
| 10 | 235300\_x\_at | NA | RCHY1 | ring finger and CHY zinc finger domain containing 1, E3 ubiquitin protein ligase | 25898 | ENSG00000163743 |
| 11 | 213225\_at | NA | PPM1B | protein phosphatase, Mg2+/Mn2+ dependent, 1B | 5495 | ENSG00000138032 |
| 12 | 200602\_at | NA | APP | amyloid beta (A4) precursor protein | 351 | ENSG00000142192 |
| 13 | 201177\_s\_at | NA | UBA2 | ubiquitin-like modifier activating enzyme 2 | 10054 | ENSG00000126261 |
| 14 | 238020\_at | NA | PSMC2 | proteasome (prosome, macropain) 26S subunit, ATPase, 2 | 5701 | ENSG00000161057 |
| 15 | 213459\_at | NA | RPL37A | ribosomal protein L37a | 6168 | ENSG00000197756 |
| 16 | 205191\_at | NA | RP2 | retinitis pigmentosa 2 (X-linked recessive) | 6102 | ENSG00000102218 |
| 17 | 214590\_s\_at | NA | UBE2D1 | ubiquitin-conjugating enzyme E2D 1 | 7321 | ENSG00000072401 |
| 18 | 201437\_s\_at | NA | EIF4E | eukaryotic translation initiation factor 4E | 1977 | ENSG00000151247 |
| 19 | 232068\_s\_at | NA | TLR4 | toll-like receptor 4 | 7099 | ENSG00000136869 |
| 20 | 218871\_x\_at | NA | CSGALNACT2 | chondroitin sulfate N-acetylgalactosaminyltransferase 2 | 55454 | ENSG00000169826 |
| 21 | 230170\_at | NA | OSM | oncostatin M | 5008 | ENSG00000099985 |
| 22 | 229943\_at | NA | TRIM13 | tripartite motif containing 13 | 10206 | ENSG00000204977 |
| 23 | 211698\_at | NA | EID1 | EP300 interacting inhibitor of differentiation 1 | 23741 | ENSG00000255302 |
| 24 | 227900\_at | NA | CBLB | Cbl proto-oncogene, E3 ubiquitin protein ligase B | 868 | ENSG00000114423 |
| 25 | 236539\_at | NA | PTPN22 | protein tyrosine phosphatase, non-receptor type 22 (lymphoid) | 26191 | ENSG00000134242 |
| 26 | 205283\_at | NA | FKTN | fukutin | 2218 | ENSG00000106692 |
| 27 | 226472\_at | NA | PPIL4 | peptidylprolyl isomerase (cyclophilin)-like 4 | 85313 | ENSG00000131013 |
| 28 | 211546\_x\_at | NA | SNCA | synuclein, alpha (non A4 component of amyloid precursor) | 6622 | ENSG00000145335 |
| 29 | 207827\_x\_at | NA | SNCA | synuclein, alpha (non A4 component of amyloid precursor) | 6622 | ENSG00000145335 |
| 30 | 236081\_at | NA | SNCA | synuclein, alpha (non A4 component of amyloid precursor) | 6622 | ENSG00000145335 |
| 31 | 204467\_s\_at | NA | SNCA | synuclein, alpha (non A4 component of amyloid precursor) | 6622 | ENSG00000145335 |
| 32 | 204466\_s\_at | NA | SNCA | synuclein, alpha (non A4 component of amyloid precursor) | 6622 | ENSG00000145335 |
| 33 | 227697\_at | NA | SOCS3 | suppressor of cytokine signaling 3 | 9021 | ENSG00000184557 |
| 34 | 205900\_at | NA | KRT1 | keratin 1 | 3848 | ENSG00000167768 |
| 35 | 201407\_s\_at | NA | PPP1CB | protein phosphatase 1, catalytic subunit, beta isozyme | 5500 | ENSG00000213639 |
| 36 | 203603\_s\_at | NA | ZEB2 | zinc finger E-box binding homeobox 2 | 9839 | ENSG00000169554 |
| 37 | 204794\_at | NA | DUSP2 | dual specificity phosphatase 2 | 1844 | ENSG00000158050 |
| 38 | 202375\_at | NA | SEC24D | SEC24 family, member D (S. cerevisiae) | 9871 | ENSG00000150961 |
| 39 | 226366\_at | NA | SHPRH | SNF2 histone linker PHD RING helicase, E3 ubiquitin protein ligase | 257218 | ENSG00000146414 |
| 40 | 214917\_at | NA | PRKAA1 | protein kinase, AMP-activated, alpha 1 catalytic subunit | 5562 | ENSG00000132356 |
| 41 | 1554309\_at | NA | EIF4G3 | eukaryotic translation initiation factor 4 gamma, 3 | 8672 | ENSG00000075151 |
| 42 | 220018\_at | NA | CBLL1 | Cbl proto-oncogene, E3 ubiquitin protein ligase-like 1 | 79872 | ENSG00000105879 |
| 43 | 210746\_s\_at | NA | EPB42 | erythrocyte membrane protein band 4.2 | 2038 | ENSG00000166947 |
| 44 | 204614\_at | NA | SERPINB2 | serpin peptidase inhibitor, clade B (ovalbumin), member 2 | 5055 | ENSG00000197632 |
| 45 | 202843\_at | NA | DNAJB9 | DnaJ (Hsp40) homolog, subfamily B, member 9 | 4189 | ENSG00000128590 |
| 46 | 202887\_s\_at | NA | DDIT4 | DNA-damage-inducible transcript 4 | 54541 | ENSG00000168209 |
| 47 | 208762\_at | NA | SUMO1 | SMT3 suppressor of mif two 3 homolog 1 (S. cerevisiae) | 7341 | ENSG00000116030 |
| 48 | 203358\_s\_at | NA | EZH2 | enhancer of zeste homolog 2 (Drosophila) | 2146 | ENSG00000106462 |
| 49 | 216929\_x\_at | NA | ABO | ABO blood group (transferase A, alpha 1-3-N-acetylgalactosaminyltransferase; transferase B, alpha 1-3-galactosyltransferase) | 28 | NULL |
| 50 | 203552\_at | NA | MAP4K5 | mitogen-activated protein kinase kinase kinase kinase 5 | 11183 | ENSG00000012983 |
| 51 | 204176\_at | NA | KLHL20 | kelch-like 20 (Drosophila) | 27252 | ENSG00000076321 |
| 52 | 1569136\_at | NA | MGAT4A | mannosyl (alpha-1,3-)-glycoprotein beta-1,4-N-acetylglucosaminyltransferase, isozyme A | 11320 | ENSG00000071073 |
| 53 | 222858\_s\_at | NA | DAPP1 | dual adaptor of phosphotyrosine and 3-phosphoinositides | 27071 | ENSG00000070190 |
| 54 | 212637\_s\_at | NA | WWP1 | WW domain containing E3 ubiquitin protein ligase 1 | 11059 | ENSG00000123124 |
| 55 | 208078\_s\_at | NA | SIK1 | salt-inducible kinase 1 | 150094 | ENSG00000142178 |
| 56 | 1555962\_at | NA | B3GNT7 | UDP-GlcNAc:betaGal beta-1,3-N-acetylglucosaminyltransferase 7 | 93010 | ENSG00000156966 |
| 57 | 226279\_at | NA | PRSS23 | protease, serine, 23 | 11098 | ENSG00000150687 |
| 58 | 202644\_s\_at | NA | TNFAIP3 | tumor necrosis factor, alpha-induced protein 3 | 7128 | ENSG00000118503 |
| 59 | 202203\_s\_at | NA | AMFR | autocrine motility factor receptor, E3 ubiquitin protein ligase | 267 | ENSG00000159461 |
| 60 | 207794\_at | NA | CCR2 | chemokine (C-C motif) receptor 2 | 729230 | ENSG00000121807 |
| 61 | 231863\_at | NA | ING3 | inhibitor of growth family, member 3 | 54556 | ENSG00000071243 |
| 62 | 223266\_at | NA | STRADB | STE20-related kinase adaptor beta | 55437 | ENSG00000082146 |
| 63 | 1555878\_at | NA | RPS24 | ribosomal protein S24 | 6229 | ENSG00000138326 |
| 64 | 211560\_s\_at | NA | ALAS2 | aminolevulinate, delta-, synthase 2 | 212 | ENSG00000158578 |
| 65 | 202933\_s\_at | NA | YES1 | v-yes-1 Yamaguchi sarcoma viral oncogene homolog 1 | 7525 | ENSG00000176105 |
| 66 | 204995\_at | NA | CDK5R1 | cyclin-dependent kinase 5, regulatory subunit 1 (p35) | 8851 | ENSG00000176749 |
| 67 | 1557257\_at | NA | BCL10 | B-cell CLL/lymphoma 10 | 8915 | ENSG00000142867 |
| 68 | 1559975\_at | NA | BTG1 | B-cell translocation gene 1, anti-proliferative | 694 | ENSG00000133639 |
| 69 | 218411\_s\_at | NA | MBIP | MAP3K12 binding inhibitory protein 1 | 51562 | ENSG00000151332 |
| 70 | 1555476\_at | NA | IREB2 | iron-responsive element binding protein 2 | 3658 | ENSG00000136381 |
| 71 | 239143\_x\_at | NA | RNF138 | ring finger protein 138, E3 ubiquitin protein ligase | 51444 | ENSG00000134758 |
| 72 | 202232\_s\_at | NA | EIF3M | eukaryotic translation initiation factor 3, subunit M | 10480 | ENSG00000149100 |
| 73 | 205767\_at | NA | EREG | epiregulin | 2069 | ENSG00000124882 |
| 74 | 214012\_at | NA | ERAP1 | endoplasmic reticulum aminopeptidase 1 | 51752 | ENSG00000164307 |
| 75 | 219304\_s\_at | NA | PDGFD | platelet derived growth factor D | 80310 | ENSG00000170962 |
| 76 | 230559\_x\_at | NA | FGD4 | FYVE, RhoGEF and PH domain containing 4 | 121512 | ENSG00000139132 |
| 77 | 212633\_at | NA | UFL1 | UFM1-specific ligase 1 | 23376 | ENSG00000014123 |
| 78 | 207300\_s\_at | NA | F7 | coagulation factor VII (serum prothrombin conversion accelerator) | 2155 | ENSG00000057593 |
| 79 | 232044\_at | NA | RBBP6 | retinoblastoma binding protein 6 | 5930 | ENSG00000122257 |

  
  

| **Database:biological process      &nbspName:cellular metabolic process      &nbspID:GO:0044237** | | | | | | |
| --- | --- | --- | --- | --- | --- | --- |
| C=8161; O=130; E=100.29; R=1.30; rawP=9.68e-07; adjP=0.0002 | | | | | | |
| Index | UserID | Value | Gene Symbol | Gene Name | EntrezGene | Ensembl |
| 1 | 228106\_at | NA | DCAF16 | DDB1 and CUL4 associated factor 16 | 54876 | ENSG00000163257 |
| 2 | 219312\_s\_at | NA | ZBTB10 | zinc finger and BTB domain containing 10 | 65986 | ENSG00000205189 |
| 3 | 227449\_at | NA | EPHA4 | EPH receptor A4 | 2043 | ENSG00000116106 |
| 4 | 201016\_at | NA | EIF1AX | eukaryotic translation initiation factor 1A, X-linked | 1964 | ENSG00000173674 |
| 5 | 235300\_x\_at | NA | RCHY1 | ring finger and CHY zinc finger domain containing 1, E3 ubiquitin protein ligase | 25898 | ENSG00000163743 |
| 6 | 207078\_at | NA | MED6 | mediator complex subunit 6 | 10001 | ENSG00000133997 |
| 7 | 202660\_at | NA | ITPR2 | inositol 1,4,5-trisphosphate receptor, type 2 | 3709 | ENSG00000123104 |
| 8 | 202219\_at | NA | SLC6A8 | solute carrier family 6 (neurotransmitter transporter, creatine), member 8 | 6535 | ENSG00000130821 |
| 9 | 1558692\_at | NA | C1orf85 | chromosome 1 open reading frame 85 | 112770 | ENSG00000198715 |
| 10 | 203203\_s\_at | NA | KRR1 | KRR1, small subunit (SSU) processome component, homolog (yeast) | 11103 | ENSG00000111615 |
| 11 | 202314\_at | NA | CYP51A1 | cytochrome P450, family 51, subfamily A, polypeptide 1 | 1595 | ENSG00000001630 |
| 12 | 226680\_at | NA | IKZF5 | IKAROS family zinc finger 5 (Pegasus) | 64376 | ENSG00000095574 |
| 13 | 238020\_at | NA | PSMC2 | proteasome (prosome, macropain) 26S subunit, ATPase, 2 | 5701 | ENSG00000161057 |
| 14 | 213459\_at | NA | RPL37A | ribosomal protein L37a | 6168 | ENSG00000197756 |
| 15 | 205191\_at | NA | RP2 | retinitis pigmentosa 2 (X-linked recessive) | 6102 | ENSG00000102218 |
| 16 | 232068\_s\_at | NA | TLR4 | toll-like receptor 4 | 7099 | ENSG00000136869 |
| 17 | 201437\_s\_at | NA | EIF4E | eukaryotic translation initiation factor 4E | 1977 | ENSG00000151247 |
| 18 | 218871\_x\_at | NA | CSGALNACT2 | chondroitin sulfate N-acetylgalactosaminyltransferase 2 | 55454 | ENSG00000169826 |
| 19 | 204299\_at | NA | SRSF10 | serine/arginine-rich splicing factor 10 | 10772 | ENSG00000188529 |
| 20 | 230170\_at | NA | OSM | oncostatin M | 5008 | ENSG00000099985 |
| 21 | 229943\_at | NA | TRIM13 | tripartite motif containing 13 | 10206 | ENSG00000204977 |
| 22 | 222848\_at | NA | CENPK | centromere protein K | 64105 | ENSG00000123219 |
| 23 | 205283\_at | NA | FKTN | fukutin | 2218 | ENSG00000106692 |
| 24 | 223200\_s\_at | NA | LSG1 | large subunit GTPase 1 homolog (S. cerevisiae) | 55341 | ENSG00000041802 |
| 25 | 218401\_s\_at | NA | ZNF281 | zinc finger protein 281 | 23528 | ENSG00000162702 |
| 26 | 228785\_at | NA | ZNF281 | zinc finger protein 281 | 23528 | ENSG00000162702 |
| 27 | 202861\_at | NA | PER1 | period homolog 1 (Drosophila) | 5187 | ENSG00000179094 |
| 28 | 201088\_at | NA | KPNA2 | karyopherin alpha 2 (RAG cohort 1, importin alpha 1) | 3838 | ENSG00000182481 |
| 29 | 229431\_at | NA | RFXAP | regulatory factor X-associated protein | 5994 | ENSG00000133111 |
| 30 | 36711\_at | NA | MAFF | v-maf musculoaponeurotic fibrosarcoma oncogene homolog F (avian) | 23764 | ENSG00000185022 |
| 31 | 212847\_at | NA | FUBP1 | far upstream element (FUSE) binding protein 1 | 8880 | ENSG00000162613 |
| 32 | 227391\_x\_at | NA | LRRFIP1 | leucine rich repeat (in FLII) interacting protein 1 | 9208 | ENSG00000124831 |
| 33 | 222876\_s\_at | NA | ADAP2 | ArfGAP with dual PH domains 2 | 55803 | ENSG00000184060 |
| 34 | 202375\_at | NA | SEC24D | SEC24 family, member D (S. cerevisiae) | 9871 | ENSG00000150961 |
| 35 | 214917\_at | NA | PRKAA1 | protein kinase, AMP-activated, alpha 1 catalytic subunit | 5562 | ENSG00000132356 |
| 36 | 217739\_s\_at | NA | NAMPT | nicotinamide phosphoribosyltransferase | 10135 | ENSG00000105835 |
| 37 | 202887\_s\_at | NA | DDIT4 | DNA-damage-inducible transcript 4 | 54541 | ENSG00000168209 |
| 38 | 202904\_s\_at | NA | LSM5 | LSM5 homolog, U6 small nuclear RNA associated (S. cerevisiae) | 23658 | ENSG00000106355 |
| 39 | 203552\_at | NA | MAP4K5 | mitogen-activated protein kinase kinase kinase kinase 5 | 11183 | ENSG00000012983 |
| 40 | 222765\_x\_at | NA | ESF1 | ESF1, nucleolar pre-rRNA processing protein, homolog (S. cerevisiae) | 51575 | ENSG00000089048 |
| 41 | 208078\_s\_at | NA | SIK1 | salt-inducible kinase 1 | 150094 | ENSG00000142178 |
| 42 | 1555962\_at | NA | B3GNT7 | UDP-GlcNAc:betaGal beta-1,3-N-acetylglucosaminyltransferase 7 | 93010 | ENSG00000156966 |
| 43 | 1555274\_a\_at | NA | EPT1 | ethanolaminephosphotransferase 1 (CDP-ethanolamine-specific) | 85465 | ENSG00000138018 |
| 44 | 202498\_s\_at | NA | SLC2A3 | solute carrier family 2 (facilitated glucose transporter), member 3 | 6515 | ENSG00000059804 |
| 45 | 238199\_x\_at | NA | COX3 | cytochrome c oxidase III | 4514 | NULL |
| 46 | 1555878\_at | NA | RPS24 | ribosomal protein S24 | 6229 | ENSG00000138326 |
| 47 | 223266\_at | NA | STRADB | STE20-related kinase adaptor beta | 55437 | ENSG00000082146 |
| 48 | 231863\_at | NA | ING3 | inhibitor of growth family, member 3 | 54556 | ENSG00000071243 |
| 49 | 211560\_s\_at | NA | ALAS2 | aminolevulinate, delta-, synthase 2 | 212 | ENSG00000158578 |
| 50 | 205063\_at | NA | GEMIN2 | gem (nuclear organelle) associated protein 2 | 8487 | ENSG00000092208 |
| 51 | 203634\_s\_at | NA | CPT1A | carnitine palmitoyltransferase 1A (liver) | 1374 | ENSG00000110090 |
| 52 | 204995\_at | NA | CDK5R1 | cyclin-dependent kinase 5, regulatory subunit 1 (p35) | 8851 | ENSG00000176749 |
| 53 | 228063\_s\_at | NA | NAP1L5 | nucleosome assembly protein 1-like 5 | 266812 | ENSG00000177432 |
| 54 | 228062\_at | NA | NAP1L5 | nucleosome assembly protein 1-like 5 | 266812 | ENSG00000177432 |
| 55 | 218411\_s\_at | NA | MBIP | MAP3K12 binding inhibitory protein 1 | 51562 | ENSG00000151332 |
| 56 | 203574\_at | NA | NFIL3 | nuclear factor, interleukin 3 regulated | 4783 | ENSG00000165030 |
| 57 | 205767\_at | NA | EREG | epiregulin | 2069 | ENSG00000124882 |
| 58 | 202232\_s\_at | NA | EIF3M | eukaryotic translation initiation factor 3, subunit M | 10480 | ENSG00000149100 |
| 59 | 230559\_x\_at | NA | FGD4 | FYVE, RhoGEF and PH domain containing 4 | 121512 | ENSG00000139132 |
| 60 | 238614\_x\_at | NA | ZNF430 | zinc finger protein 430 | 80264 | ENSG00000118620 |
| 61 | 216248\_s\_at | NA | NR4A2 | nuclear receptor subfamily 4, group A, member 2 | 4929 | ENSG00000153234 |
| 62 | 204621\_s\_at | NA | NR4A2 | nuclear receptor subfamily 4, group A, member 2 | 4929 | ENSG00000153234 |
| 63 | 204622\_x\_at | NA | NR4A2 | nuclear receptor subfamily 4, group A, member 2 | 4929 | ENSG00000153234 |
| 64 | 232044\_at | NA | RBBP6 | retinoblastoma binding protein 6 | 5930 | ENSG00000122257 |
| 65 | 200608\_s\_at | NA | RAD21 | RAD21 homolog (S. pombe) | 5885 | ENSG00000164754 |
| 66 | 202221\_s\_at | NA | EP300 | E1A binding protein p300 | 2033 | ENSG00000100393 |
| 67 | 206919\_at | NA | ELK4 | ELK4, ETS-domain protein (SRF accessory protein 1) | 2005 | ENSG00000158711 |
| 68 | 208121\_s\_at | NA | PTPRO | protein tyrosine phosphatase, receptor type, O | 5800 | ENSG00000151490 |
| 69 | 201304\_at | NA | NDUFA5 | NADH dehydrogenase (ubiquinone) 1 alpha subcomplex, 5, 13kDa | 4698 | ENSG00000128609 |
| 70 | 203821\_at | NA | HBEGF | heparin-binding EGF-like growth factor | 1839 | ENSG00000113070 |
| 71 | 225760\_at | NA | MYSM1 | Myb-like, SWIRM and MPN domains 1 | 114803 | ENSG00000162601 |
| 72 | 222310\_at | NA | SCAF4 | SR-related CTD-associated factor 4 | 57466 | ENSG00000156304 |
| 73 | 213725\_x\_at | NA | XYLT1 | xylosyltransferase I | 64131 | ENSG00000103489 |
| 74 | 224453\_s\_at | NA | ETNK1 | ethanolamine kinase 1 | 55500 | ENSG00000139163 |
| 75 | 225290\_at | NA | ETNK1 | ethanolamine kinase 1 | 55500 | ENSG00000139163 |
| 76 | 202979\_s\_at | NA | CREBZF | CREB/ATF bZIP transcription factor | 58487 | ENSG00000137504 |
| 77 | 204187\_at | NA | GMPR | guanosine monophosphate reductase | 2766 | ENSG00000137198 |
| 78 | 233019\_at | NA | CNOT7 | CCR4-NOT transcription complex, subunit 7 | 29883 | ENSG00000198791 |
| 79 | 213225\_at | NA | PPM1B | protein phosphatase, Mg2+/Mn2+ dependent, 1B | 5495 | ENSG00000138032 |
| 80 | 200602\_at | NA | APP | amyloid beta (A4) precursor protein | 351 | ENSG00000142192 |
| 81 | 225916\_at | NA | ZNF131 | zinc finger protein 131 | 7690 | ENSG00000172262 |
| 82 | 214741\_at | NA | ZNF131 | zinc finger protein 131 | 7690 | ENSG00000172262 |
| 83 | 201177\_s\_at | NA | UBA2 | ubiquitin-like modifier activating enzyme 2 | 10054 | ENSG00000126261 |
| 84 | 227375\_at | NA | ANKRD13C | ankyrin repeat domain 13C | 81573 | ENSG00000118454 |
| 85 | 202422\_s\_at | NA | ACSL4 | acyl-CoA synthetase long-chain family member 4 | 2182 | ENSG00000068366 |
| 86 | 214590\_s\_at | NA | UBE2D1 | ubiquitin-conjugating enzyme E2D 1 | 7321 | ENSG00000072401 |
| 87 | 221768\_at | NA | SFPQ | splicing factor proline/glutamine-rich | 6421 | ENSG00000116560 |
| 88 | 211698\_at | NA | EID1 | EP300 interacting inhibitor of differentiation 1 | 23741 | ENSG00000255302 |
| 89 | 202464\_s\_at | NA | PFKFB3 | 6-phosphofructo-2-kinase/fructose-2,6-biphosphatase 3 | 5209 | ENSG00000170525 |
| 90 | 236539\_at | NA | PTPN22 | protein tyrosine phosphatase, non-receptor type 22 (lymphoid) | 26191 | ENSG00000134242 |
| 91 | 226472\_at | NA | PPIL4 | peptidylprolyl isomerase (cyclophilin)-like 4 | 85313 | ENSG00000131013 |
| 92 | 227697\_at | NA | SOCS3 | suppressor of cytokine signaling 3 | 9021 | ENSG00000184557 |
| 93 | 211546\_x\_at | NA | SNCA | synuclein, alpha (non A4 component of amyloid precursor) | 6622 | ENSG00000145335 |
| 94 | 207827\_x\_at | NA | SNCA | synuclein, alpha (non A4 component of amyloid precursor) | 6622 | ENSG00000145335 |
| 95 | 236081\_at | NA | SNCA | synuclein, alpha (non A4 component of amyloid precursor) | 6622 | ENSG00000145335 |
| 96 | 204467\_s\_at | NA | SNCA | synuclein, alpha (non A4 component of amyloid precursor) | 6622 | ENSG00000145335 |
| 97 | 204466\_s\_at | NA | SNCA | synuclein, alpha (non A4 component of amyloid precursor) | 6622 | ENSG00000145335 |
| 98 | 213998\_s\_at | NA | DDX17 | DEAD (Asp-Glu-Ala-Asp) box helicase 17 | 10521 | ENSG00000100201 |
| 99 | 208719\_s\_at | NA | DDX17 | DEAD (Asp-Glu-Ala-Asp) box helicase 17 | 10521 | ENSG00000100201 |
| 100 | 208151\_x\_at | NA | DDX17 | DEAD (Asp-Glu-Ala-Asp) box helicase 17 | 10521 | ENSG00000100201 |
| 101 | 224009\_x\_at | NA | DHRS9 | dehydrogenase/reductase (SDR family) member 9 | 10170 | ENSG00000073737 |
| 102 | 223952\_x\_at | NA | DHRS9 | dehydrogenase/reductase (SDR family) member 9 | 10170 | ENSG00000073737 |
| 103 | 213524\_s\_at | NA | G0S2 | G0/G1switch 2 | 50486 | ENSG00000123689 |
| 104 | 201407\_s\_at | NA | PPP1CB | protein phosphatase 1, catalytic subunit, beta isozyme | 5500 | ENSG00000213639 |
| 105 | 1553581\_s\_at | NA | SREK1IP1 | SREK1-interacting protein 1 | 285672 | ENSG00000153006 |
| 106 | 223649\_s\_at | NA | SLC25A39 | solute carrier family 25, member 39 | 51629 | ENSG00000013306 |
| 107 | 203603\_s\_at | NA | ZEB2 | zinc finger E-box binding homeobox 2 | 9839 | ENSG00000169554 |
| 108 | 204794\_at | NA | DUSP2 | dual specificity phosphatase 2 | 1844 | ENSG00000158050 |
| 109 | 236140\_at | NA | GCLM | glutamate-cysteine ligase, modifier subunit | 2730 | ENSG00000023909 |
| 110 | 235412\_at | NA | ARHGEF7 | Rho guanine nucleotide exchange factor (GEF) 7 | 8874 | ENSG00000102606 |
| 111 | 206108\_s\_at | NA | SRSF6 | serine/arginine-rich splicing factor 6 | 6431 | ENSG00000124193 |
| 112 | 226366\_at | NA | SHPRH | SNF2 histone linker PHD RING helicase, E3 ubiquitin protein ligase | 257218 | ENSG00000146414 |
| 113 | 212989\_at | NA | SGMS1 | sphingomyelin synthase 1 | 259230 | ENSG00000198964 |
| 114 | 213786\_at | NA | TAX1BP1 | Tax1 (human T-cell leukemia virus type I) binding protein 1 | 8887 | ENSG00000106052 |
| 115 | 1554309\_at | NA | EIF4G3 | eukaryotic translation initiation factor 4 gamma, 3 | 8672 | ENSG00000075151 |
| 116 | 220018\_at | NA | CBLL1 | Cbl proto-oncogene, E3 ubiquitin protein ligase-like 1 | 79872 | ENSG00000105879 |
| 117 | 210746\_s\_at | NA | EPB42 | erythrocyte membrane protein band 4.2 | 2038 | ENSG00000166947 |
| 118 | 202843\_at | NA | DNAJB9 | DnaJ (Hsp40) homolog, subfamily B, member 9 | 4189 | ENSG00000128590 |
| 119 | 216834\_at | NA | RGS1 | regulator of G-protein signaling 1 | 5996 | ENSG00000090104 |
| 120 | 202988\_s\_at | NA | RGS1 | regulator of G-protein signaling 1 | 5996 | ENSG00000090104 |
| 121 | 208762\_at | NA | SUMO1 | SMT3 suppressor of mif two 3 homolog 1 (S. cerevisiae) | 7341 | ENSG00000116030 |
| 122 | 203358\_s\_at | NA | EZH2 | enhancer of zeste homolog 2 (Drosophila) | 2146 | ENSG00000106462 |
| 123 | 1558233\_s\_at | NA | ATF1 | activating transcription factor 1 | 466 | ENSG00000123268 |
| 124 | 216929\_x\_at | NA | ABO | ABO blood group (transferase A, alpha 1-3-N-acetylgalactosaminyltransferase; transferase B, alpha 1-3-galactosyltransferase) | 28 | NULL |
| 125 | 204176\_at | NA | KLHL20 | kelch-like 20 (Drosophila) | 27252 | ENSG00000076321 |
| 126 | 1569136\_at | NA | MGAT4A | mannosyl (alpha-1,3-)-glycoprotein beta-1,4-N-acetylglucosaminyltransferase, isozyme A | 11320 | ENSG00000071073 |
| 127 | 222858\_s\_at | NA | DAPP1 | dual adaptor of phosphotyrosine and 3-phosphoinositides | 27071 | ENSG00000070190 |
| 128 | 212637\_s\_at | NA | WWP1 | WW domain containing E3 ubiquitin protein ligase 1 | 11059 | ENSG00000123124 |
| 129 | 203543\_s\_at | NA | KLF9 | Kruppel-like factor 9 | 687 | ENSG00000119138 |
| 130 | 215716\_s\_at | NA | ATP2B1 | ATPase, Ca++ transporting, plasma membrane 1 | 490 | ENSG00000070961 |
| 131 | 202644\_s\_at | NA | TNFAIP3 | tumor necrosis factor, alpha-induced protein 3 | 7128 | ENSG00000118503 |
| 132 | 202203\_s\_at | NA | AMFR | autocrine motility factor receptor, E3 ubiquitin protein ligase | 267 | ENSG00000159461 |
| 133 | 242960\_at | NA | EPC2 | enhancer of polycomb homolog 2 (Drosophila) | 26122 | ENSG00000135999 |
| 134 | 207794\_at | NA | CCR2 | chemokine (C-C motif) receptor 2 | 729230 | ENSG00000121807 |
| 135 | 202933\_s\_at | NA | YES1 | v-yes-1 Yamaguchi sarcoma viral oncogene homolog 1 | 7525 | ENSG00000176105 |
| 136 | 222317\_at | NA | PDE3B | phosphodiesterase 3B, cGMP-inhibited | 5140 | ENSG00000152270 |
| 137 | 201091\_s\_at | NA | CBX3 | chromobox homolog 3 | 11335 | ENSG00000122565 |
| 138 | 1557257\_at | NA | BCL10 | B-cell CLL/lymphoma 10 | 8915 | ENSG00000142867 |
| 139 | 1559975\_at | NA | BTG1 | B-cell translocation gene 1, anti-proliferative | 694 | ENSG00000133639 |
| 140 | 1555476\_at | NA | IREB2 | iron-responsive element binding protein 2 | 3658 | ENSG00000136381 |
| 141 | 239143\_x\_at | NA | RNF138 | ring finger protein 138, E3 ubiquitin protein ligase | 51444 | ENSG00000134758 |
| 142 | 219304\_s\_at | NA | PDGFD | platelet derived growth factor D | 80310 | ENSG00000170962 |
| 143 | 212633\_at | NA | UFL1 | UFM1-specific ligase 1 | 23376 | ENSG00000014123 |
| 144 | 207300\_s\_at | NA | F7 | coagulation factor VII (serum prothrombin conversion accelerator) | 2155 | ENSG00000057593 |

  
  

| **Database:biological process      &nbspName:organic substance metabolic process      &nbspID:GO:0071704** | | | | | | |
| --- | --- | --- | --- | --- | --- | --- |
| C=8521; O=134; E=104.72; R=1.28; rawP=8.73e-07; adjP=0.0002 | | | | | | |
| Index | UserID | Value | Gene Symbol | Gene Name | EntrezGene | Ensembl |
| 1 | 228106\_at | NA | DCAF16 | DDB1 and CUL4 associated factor 16 | 54876 | ENSG00000163257 |
| 2 | 219312\_s\_at | NA | ZBTB10 | zinc finger and BTB domain containing 10 | 65986 | ENSG00000205189 |
| 3 | 227449\_at | NA | EPHA4 | EPH receptor A4 | 2043 | ENSG00000116106 |
| 4 | 201016\_at | NA | EIF1AX | eukaryotic translation initiation factor 1A, X-linked | 1964 | ENSG00000173674 |
| 5 | 235300\_x\_at | NA | RCHY1 | ring finger and CHY zinc finger domain containing 1, E3 ubiquitin protein ligase | 25898 | ENSG00000163743 |
| 6 | 207078\_at | NA | MED6 | mediator complex subunit 6 | 10001 | ENSG00000133997 |
| 7 | 202219\_at | NA | SLC6A8 | solute carrier family 6 (neurotransmitter transporter, creatine), member 8 | 6535 | ENSG00000130821 |
| 8 | 1558692\_at | NA | C1orf85 | chromosome 1 open reading frame 85 | 112770 | ENSG00000198715 |
| 9 | 203203\_s\_at | NA | KRR1 | KRR1, small subunit (SSU) processome component, homolog (yeast) | 11103 | ENSG00000111615 |
| 10 | 202314\_at | NA | CYP51A1 | cytochrome P450, family 51, subfamily A, polypeptide 1 | 1595 | ENSG00000001630 |
| 11 | 226680\_at | NA | IKZF5 | IKAROS family zinc finger 5 (Pegasus) | 64376 | ENSG00000095574 |
| 12 | 222243\_s\_at | NA | TOB2 | transducer of ERBB2, 2 | 10766 | ENSG00000183864 |
| 13 | 238020\_at | NA | PSMC2 | proteasome (prosome, macropain) 26S subunit, ATPase, 2 | 5701 | ENSG00000161057 |
| 14 | 213459\_at | NA | RPL37A | ribosomal protein L37a | 6168 | ENSG00000197756 |
| 15 | 205191\_at | NA | RP2 | retinitis pigmentosa 2 (X-linked recessive) | 6102 | ENSG00000102218 |
| 16 | 232068\_s\_at | NA | TLR4 | toll-like receptor 4 | 7099 | ENSG00000136869 |
| 17 | 201437\_s\_at | NA | EIF4E | eukaryotic translation initiation factor 4E | 1977 | ENSG00000151247 |
| 18 | 218871\_x\_at | NA | CSGALNACT2 | chondroitin sulfate N-acetylgalactosaminyltransferase 2 | 55454 | ENSG00000169826 |
| 19 | 204299\_at | NA | SRSF10 | serine/arginine-rich splicing factor 10 | 10772 | ENSG00000188529 |
| 20 | 230170\_at | NA | OSM | oncostatin M | 5008 | ENSG00000099985 |
| 21 | 229943\_at | NA | TRIM13 | tripartite motif containing 13 | 10206 | ENSG00000204977 |
| 22 | 227900\_at | NA | CBLB | Cbl proto-oncogene, E3 ubiquitin protein ligase B | 868 | ENSG00000114423 |
| 23 | 222848\_at | NA | CENPK | centromere protein K | 64105 | ENSG00000123219 |
| 24 | 205283\_at | NA | FKTN | fukutin | 2218 | ENSG00000106692 |
| 25 | 223200\_s\_at | NA | LSG1 | large subunit GTPase 1 homolog (S. cerevisiae) | 55341 | ENSG00000041802 |
| 26 | 218401\_s\_at | NA | ZNF281 | zinc finger protein 281 | 23528 | ENSG00000162702 |
| 27 | 228785\_at | NA | ZNF281 | zinc finger protein 281 | 23528 | ENSG00000162702 |
| 28 | 202861\_at | NA | PER1 | period homolog 1 (Drosophila) | 5187 | ENSG00000179094 |
| 29 | 205900\_at | NA | KRT1 | keratin 1 | 3848 | ENSG00000167768 |
| 30 | 201088\_at | NA | KPNA2 | karyopherin alpha 2 (RAG cohort 1, importin alpha 1) | 3838 | ENSG00000182481 |
| 31 | 229431\_at | NA | RFXAP | regulatory factor X-associated protein | 5994 | ENSG00000133111 |
| 32 | 36711\_at | NA | MAFF | v-maf musculoaponeurotic fibrosarcoma oncogene homolog F (avian) | 23764 | ENSG00000185022 |
| 33 | 212847\_at | NA | FUBP1 | far upstream element (FUSE) binding protein 1 | 8880 | ENSG00000162613 |
| 34 | 227391\_x\_at | NA | LRRFIP1 | leucine rich repeat (in FLII) interacting protein 1 | 9208 | ENSG00000124831 |
| 35 | 222876\_s\_at | NA | ADAP2 | ArfGAP with dual PH domains 2 | 55803 | ENSG00000184060 |
| 36 | 202375\_at | NA | SEC24D | SEC24 family, member D (S. cerevisiae) | 9871 | ENSG00000150961 |
| 37 | 214917\_at | NA | PRKAA1 | protein kinase, AMP-activated, alpha 1 catalytic subunit | 5562 | ENSG00000132356 |
| 38 | 204614\_at | NA | SERPINB2 | serpin peptidase inhibitor, clade B (ovalbumin), member 2 | 5055 | ENSG00000197632 |
| 39 | 217739\_s\_at | NA | NAMPT | nicotinamide phosphoribosyltransferase | 10135 | ENSG00000105835 |
| 40 | 202887\_s\_at | NA | DDIT4 | DNA-damage-inducible transcript 4 | 54541 | ENSG00000168209 |
| 41 | 202904\_s\_at | NA | LSM5 | LSM5 homolog, U6 small nuclear RNA associated (S. cerevisiae) | 23658 | ENSG00000106355 |
| 42 | 203552\_at | NA | MAP4K5 | mitogen-activated protein kinase kinase kinase kinase 5 | 11183 | ENSG00000012983 |
| 43 | 222765\_x\_at | NA | ESF1 | ESF1, nucleolar pre-rRNA processing protein, homolog (S. cerevisiae) | 51575 | ENSG00000089048 |
| 44 | 208078\_s\_at | NA | SIK1 | salt-inducible kinase 1 | 150094 | ENSG00000142178 |
| 45 | 1555962\_at | NA | B3GNT7 | UDP-GlcNAc:betaGal beta-1,3-N-acetylglucosaminyltransferase 7 | 93010 | ENSG00000156966 |
| 46 | 1555274\_a\_at | NA | EPT1 | ethanolaminephosphotransferase 1 (CDP-ethanolamine-specific) | 85465 | ENSG00000138018 |
| 47 | 202498\_s\_at | NA | SLC2A3 | solute carrier family 2 (facilitated glucose transporter), member 3 | 6515 | ENSG00000059804 |
| 48 | 226279\_at | NA | PRSS23 | protease, serine, 23 | 11098 | ENSG00000150687 |
| 49 | 1555878\_at | NA | RPS24 | ribosomal protein S24 | 6229 | ENSG00000138326 |
| 50 | 223266\_at | NA | STRADB | STE20-related kinase adaptor beta | 55437 | ENSG00000082146 |
| 51 | 231863\_at | NA | ING3 | inhibitor of growth family, member 3 | 54556 | ENSG00000071243 |
| 52 | 211560\_s\_at | NA | ALAS2 | aminolevulinate, delta-, synthase 2 | 212 | ENSG00000158578 |
| 53 | 205063\_at | NA | GEMIN2 | gem (nuclear organelle) associated protein 2 | 8487 | ENSG00000092208 |
| 54 | 203634\_s\_at | NA | CPT1A | carnitine palmitoyltransferase 1A (liver) | 1374 | ENSG00000110090 |
| 55 | 204995\_at | NA | CDK5R1 | cyclin-dependent kinase 5, regulatory subunit 1 (p35) | 8851 | ENSG00000176749 |
| 56 | 228063\_s\_at | NA | NAP1L5 | nucleosome assembly protein 1-like 5 | 266812 | ENSG00000177432 |
| 57 | 228062\_at | NA | NAP1L5 | nucleosome assembly protein 1-like 5 | 266812 | ENSG00000177432 |
| 58 | 218411\_s\_at | NA | MBIP | MAP3K12 binding inhibitory protein 1 | 51562 | ENSG00000151332 |
| 59 | 203574\_at | NA | NFIL3 | nuclear factor, interleukin 3 regulated | 4783 | ENSG00000165030 |
| 60 | 205767\_at | NA | EREG | epiregulin | 2069 | ENSG00000124882 |
| 61 | 202232\_s\_at | NA | EIF3M | eukaryotic translation initiation factor 3, subunit M | 10480 | ENSG00000149100 |
| 62 | 230559\_x\_at | NA | FGD4 | FYVE, RhoGEF and PH domain containing 4 | 121512 | ENSG00000139132 |
| 63 | 238614\_x\_at | NA | ZNF430 | zinc finger protein 430 | 80264 | ENSG00000118620 |
| 64 | 216248\_s\_at | NA | NR4A2 | nuclear receptor subfamily 4, group A, member 2 | 4929 | ENSG00000153234 |
| 65 | 204621\_s\_at | NA | NR4A2 | nuclear receptor subfamily 4, group A, member 2 | 4929 | ENSG00000153234 |
| 66 | 204622\_x\_at | NA | NR4A2 | nuclear receptor subfamily 4, group A, member 2 | 4929 | ENSG00000153234 |
| 67 | 232044\_at | NA | RBBP6 | retinoblastoma binding protein 6 | 5930 | ENSG00000122257 |
| 68 | 200608\_s\_at | NA | RAD21 | RAD21 homolog (S. pombe) | 5885 | ENSG00000164754 |
| 69 | 202221\_s\_at | NA | EP300 | E1A binding protein p300 | 2033 | ENSG00000100393 |
| 70 | 206919\_at | NA | ELK4 | ELK4, ETS-domain protein (SRF accessory protein 1) | 2005 | ENSG00000158711 |
| 71 | 208121\_s\_at | NA | PTPRO | protein tyrosine phosphatase, receptor type, O | 5800 | ENSG00000151490 |
| 72 | 203821\_at | NA | HBEGF | heparin-binding EGF-like growth factor | 1839 | ENSG00000113070 |
| 73 | 225760\_at | NA | MYSM1 | Myb-like, SWIRM and MPN domains 1 | 114803 | ENSG00000162601 |
| 74 | 222310\_at | NA | SCAF4 | SR-related CTD-associated factor 4 | 57466 | ENSG00000156304 |
| 75 | 213725\_x\_at | NA | XYLT1 | xylosyltransferase I | 64131 | ENSG00000103489 |
| 76 | 224453\_s\_at | NA | ETNK1 | ethanolamine kinase 1 | 55500 | ENSG00000139163 |
| 77 | 225290\_at | NA | ETNK1 | ethanolamine kinase 1 | 55500 | ENSG00000139163 |
| 78 | 202979\_s\_at | NA | CREBZF | CREB/ATF bZIP transcription factor | 58487 | ENSG00000137504 |
| 79 | 204187\_at | NA | GMPR | guanosine monophosphate reductase | 2766 | ENSG00000137198 |
| 80 | 233019\_at | NA | CNOT7 | CCR4-NOT transcription complex, subunit 7 | 29883 | ENSG00000198791 |
| 81 | 213225\_at | NA | PPM1B | protein phosphatase, Mg2+/Mn2+ dependent, 1B | 5495 | ENSG00000138032 |
| 82 | 200602\_at | NA | APP | amyloid beta (A4) precursor protein | 351 | ENSG00000142192 |
| 83 | 225916\_at | NA | ZNF131 | zinc finger protein 131 | 7690 | ENSG00000172262 |
| 84 | 214741\_at | NA | ZNF131 | zinc finger protein 131 | 7690 | ENSG00000172262 |
| 85 | 201177\_s\_at | NA | UBA2 | ubiquitin-like modifier activating enzyme 2 | 10054 | ENSG00000126261 |
| 86 | 227375\_at | NA | ANKRD13C | ankyrin repeat domain 13C | 81573 | ENSG00000118454 |
| 87 | 202422\_s\_at | NA | ACSL4 | acyl-CoA synthetase long-chain family member 4 | 2182 | ENSG00000068366 |
| 88 | 214590\_s\_at | NA | UBE2D1 | ubiquitin-conjugating enzyme E2D 1 | 7321 | ENSG00000072401 |
| 89 | 221768\_at | NA | SFPQ | splicing factor proline/glutamine-rich | 6421 | ENSG00000116560 |
| 90 | 211698\_at | NA | EID1 | EP300 interacting inhibitor of differentiation 1 | 23741 | ENSG00000255302 |
| 91 | 202464\_s\_at | NA | PFKFB3 | 6-phosphofructo-2-kinase/fructose-2,6-biphosphatase 3 | 5209 | ENSG00000170525 |
| 92 | 236539\_at | NA | PTPN22 | protein tyrosine phosphatase, non-receptor type 22 (lymphoid) | 26191 | ENSG00000134242 |
| 93 | 226472\_at | NA | PPIL4 | peptidylprolyl isomerase (cyclophilin)-like 4 | 85313 | ENSG00000131013 |
| 94 | 227697\_at | NA | SOCS3 | suppressor of cytokine signaling 3 | 9021 | ENSG00000184557 |
| 95 | 211546\_x\_at | NA | SNCA | synuclein, alpha (non A4 component of amyloid precursor) | 6622 | ENSG00000145335 |
| 96 | 207827\_x\_at | NA | SNCA | synuclein, alpha (non A4 component of amyloid precursor) | 6622 | ENSG00000145335 |
| 97 | 236081\_at | NA | SNCA | synuclein, alpha (non A4 component of amyloid precursor) | 6622 | ENSG00000145335 |
| 98 | 204467\_s\_at | NA | SNCA | synuclein, alpha (non A4 component of amyloid precursor) | 6622 | ENSG00000145335 |
| 99 | 204466\_s\_at | NA | SNCA | synuclein, alpha (non A4 component of amyloid precursor) | 6622 | ENSG00000145335 |
| 100 | 213998\_s\_at | NA | DDX17 | DEAD (Asp-Glu-Ala-Asp) box helicase 17 | 10521 | ENSG00000100201 |
| 101 | 208719\_s\_at | NA | DDX17 | DEAD (Asp-Glu-Ala-Asp) box helicase 17 | 10521 | ENSG00000100201 |
| 102 | 208151\_x\_at | NA | DDX17 | DEAD (Asp-Glu-Ala-Asp) box helicase 17 | 10521 | ENSG00000100201 |
| 103 | 224009\_x\_at | NA | DHRS9 | dehydrogenase/reductase (SDR family) member 9 | 10170 | ENSG00000073737 |
| 104 | 223952\_x\_at | NA | DHRS9 | dehydrogenase/reductase (SDR family) member 9 | 10170 | ENSG00000073737 |
| 105 | 213524\_s\_at | NA | G0S2 | G0/G1switch 2 | 50486 | ENSG00000123689 |
| 106 | 201407\_s\_at | NA | PPP1CB | protein phosphatase 1, catalytic subunit, beta isozyme | 5500 | ENSG00000213639 |
| 107 | 1553581\_s\_at | NA | SREK1IP1 | SREK1-interacting protein 1 | 285672 | ENSG00000153006 |
| 108 | 223649\_s\_at | NA | SLC25A39 | solute carrier family 25, member 39 | 51629 | ENSG00000013306 |
| 109 | 203603\_s\_at | NA | ZEB2 | zinc finger E-box binding homeobox 2 | 9839 | ENSG00000169554 |
| 110 | 204794\_at | NA | DUSP2 | dual specificity phosphatase 2 | 1844 | ENSG00000158050 |
| 111 | 236140\_at | NA | GCLM | glutamate-cysteine ligase, modifier subunit | 2730 | ENSG00000023909 |
| 112 | 235412\_at | NA | ARHGEF7 | Rho guanine nucleotide exchange factor (GEF) 7 | 8874 | ENSG00000102606 |
| 113 | 206108\_s\_at | NA | SRSF6 | serine/arginine-rich splicing factor 6 | 6431 | ENSG00000124193 |
| 114 | 226366\_at | NA | SHPRH | SNF2 histone linker PHD RING helicase, E3 ubiquitin protein ligase | 257218 | ENSG00000146414 |
| 115 | 212989\_at | NA | SGMS1 | sphingomyelin synthase 1 | 259230 | ENSG00000198964 |
| 116 | 213786\_at | NA | TAX1BP1 | Tax1 (human T-cell leukemia virus type I) binding protein 1 | 8887 | ENSG00000106052 |
| 117 | 1554309\_at | NA | EIF4G3 | eukaryotic translation initiation factor 4 gamma, 3 | 8672 | ENSG00000075151 |
| 118 | 220018\_at | NA | CBLL1 | Cbl proto-oncogene, E3 ubiquitin protein ligase-like 1 | 79872 | ENSG00000105879 |
| 119 | 210746\_s\_at | NA | EPB42 | erythrocyte membrane protein band 4.2 | 2038 | ENSG00000166947 |
| 120 | 202843\_at | NA | DNAJB9 | DnaJ (Hsp40) homolog, subfamily B, member 9 | 4189 | ENSG00000128590 |
| 121 | 216834\_at | NA | RGS1 | regulator of G-protein signaling 1 | 5996 | ENSG00000090104 |
| 122 | 202988\_s\_at | NA | RGS1 | regulator of G-protein signaling 1 | 5996 | ENSG00000090104 |
| 123 | 208762\_at | NA | SUMO1 | SMT3 suppressor of mif two 3 homolog 1 (S. cerevisiae) | 7341 | ENSG00000116030 |
| 124 | 203358\_s\_at | NA | EZH2 | enhancer of zeste homolog 2 (Drosophila) | 2146 | ENSG00000106462 |
| 125 | 1558233\_s\_at | NA | ATF1 | activating transcription factor 1 | 466 | ENSG00000123268 |
| 126 | 216929\_x\_at | NA | ABO | ABO blood group (transferase A, alpha 1-3-N-acetylgalactosaminyltransferase; transferase B, alpha 1-3-galactosyltransferase) | 28 | NULL |
| 127 | 204176\_at | NA | KLHL20 | kelch-like 20 (Drosophila) | 27252 | ENSG00000076321 |
| 128 | 1569136\_at | NA | MGAT4A | mannosyl (alpha-1,3-)-glycoprotein beta-1,4-N-acetylglucosaminyltransferase, isozyme A | 11320 | ENSG00000071073 |
| 129 | 222858\_s\_at | NA | DAPP1 | dual adaptor of phosphotyrosine and 3-phosphoinositides | 27071 | ENSG00000070190 |
| 130 | 212637\_s\_at | NA | WWP1 | WW domain containing E3 ubiquitin protein ligase 1 | 11059 | ENSG00000123124 |
| 131 | 203543\_s\_at | NA | KLF9 | Kruppel-like factor 9 | 687 | ENSG00000119138 |
| 132 | 212579\_at | NA | SMCHD1 | structural maintenance of chromosomes flexible hinge domain containing 1 | 23347 | ENSG00000101596 |
| 133 | 215716\_s\_at | NA | ATP2B1 | ATPase, Ca++ transporting, plasma membrane 1 | 490 | ENSG00000070961 |
| 134 | 202644\_s\_at | NA | TNFAIP3 | tumor necrosis factor, alpha-induced protein 3 | 7128 | ENSG00000118503 |
| 135 | 202203\_s\_at | NA | AMFR | autocrine motility factor receptor, E3 ubiquitin protein ligase | 267 | ENSG00000159461 |
| 136 | 242960\_at | NA | EPC2 | enhancer of polycomb homolog 2 (Drosophila) | 26122 | ENSG00000135999 |
| 137 | 207794\_at | NA | CCR2 | chemokine (C-C motif) receptor 2 | 729230 | ENSG00000121807 |
| 138 | 202933\_s\_at | NA | YES1 | v-yes-1 Yamaguchi sarcoma viral oncogene homolog 1 | 7525 | ENSG00000176105 |
| 139 | 222317\_at | NA | PDE3B | phosphodiesterase 3B, cGMP-inhibited | 5140 | ENSG00000152270 |
| 140 | 201091\_s\_at | NA | CBX3 | chromobox homolog 3 | 11335 | ENSG00000122565 |
| 141 | 1557257\_at | NA | BCL10 | B-cell CLL/lymphoma 10 | 8915 | ENSG00000142867 |
| 142 | 1559975\_at | NA | BTG1 | B-cell translocation gene 1, anti-proliferative | 694 | ENSG00000133639 |
| 143 | 1555476\_at | NA | IREB2 | iron-responsive element binding protein 2 | 3658 | ENSG00000136381 |
| 144 | 239143\_x\_at | NA | RNF138 | ring finger protein 138, E3 ubiquitin protein ligase | 51444 | ENSG00000134758 |
| 145 | 214012\_at | NA | ERAP1 | endoplasmic reticulum aminopeptidase 1 | 51752 | ENSG00000164307 |
| 146 | 219304\_s\_at | NA | PDGFD | platelet derived growth factor D | 80310 | ENSG00000170962 |
| 147 | 212633\_at | NA | UFL1 | UFM1-specific ligase 1 | 23376 | ENSG00000014123 |
| 148 | 207300\_s\_at | NA | F7 | coagulation factor VII (serum prothrombin conversion accelerator) | 2155 | ENSG00000057593 |

  
  

| **Database:biological process      &nbspName:metabolic process      &nbspID:GO:0008152** | | | | | | |
| --- | --- | --- | --- | --- | --- | --- |
| C=8966; O=138; E=110.18; R=1.25; rawP=1.59e-06; adjP=0.0003 | | | | | | |
| Index | UserID | Value | Gene Symbol | Gene Name | EntrezGene | Ensembl |
| 1 | 228106\_at | NA | DCAF16 | DDB1 and CUL4 associated factor 16 | 54876 | ENSG00000163257 |
| 2 | 219312\_s\_at | NA | ZBTB10 | zinc finger and BTB domain containing 10 | 65986 | ENSG00000205189 |
| 3 | 227449\_at | NA | EPHA4 | EPH receptor A4 | 2043 | ENSG00000116106 |
| 4 | 201016\_at | NA | EIF1AX | eukaryotic translation initiation factor 1A, X-linked | 1964 | ENSG00000173674 |
| 5 | 235300\_x\_at | NA | RCHY1 | ring finger and CHY zinc finger domain containing 1, E3 ubiquitin protein ligase | 25898 | ENSG00000163743 |
| 6 | 207078\_at | NA | MED6 | mediator complex subunit 6 | 10001 | ENSG00000133997 |
| 7 | 202660\_at | NA | ITPR2 | inositol 1,4,5-trisphosphate receptor, type 2 | 3709 | ENSG00000123104 |
| 8 | 202219\_at | NA | SLC6A8 | solute carrier family 6 (neurotransmitter transporter, creatine), member 8 | 6535 | ENSG00000130821 |
| 9 | 1558692\_at | NA | C1orf85 | chromosome 1 open reading frame 85 | 112770 | ENSG00000198715 |
| 10 | 203203\_s\_at | NA | KRR1 | KRR1, small subunit (SSU) processome component, homolog (yeast) | 11103 | ENSG00000111615 |
| 11 | 202314\_at | NA | CYP51A1 | cytochrome P450, family 51, subfamily A, polypeptide 1 | 1595 | ENSG00000001630 |
| 12 | 226680\_at | NA | IKZF5 | IKAROS family zinc finger 5 (Pegasus) | 64376 | ENSG00000095574 |
| 13 | 222243\_s\_at | NA | TOB2 | transducer of ERBB2, 2 | 10766 | ENSG00000183864 |
| 14 | 238020\_at | NA | PSMC2 | proteasome (prosome, macropain) 26S subunit, ATPase, 2 | 5701 | ENSG00000161057 |
| 15 | 230379\_x\_at | NA | C2orf56 | chromosome 2 open reading frame 56 | 55471 | ENSG00000003509 |
| 16 | 213459\_at | NA | RPL37A | ribosomal protein L37a | 6168 | ENSG00000197756 |
| 17 | 205191\_at | NA | RP2 | retinitis pigmentosa 2 (X-linked recessive) | 6102 | ENSG00000102218 |
| 18 | 201437\_s\_at | NA | EIF4E | eukaryotic translation initiation factor 4E | 1977 | ENSG00000151247 |
| 19 | 232068\_s\_at | NA | TLR4 | toll-like receptor 4 | 7099 | ENSG00000136869 |
| 20 | 218871\_x\_at | NA | CSGALNACT2 | chondroitin sulfate N-acetylgalactosaminyltransferase 2 | 55454 | ENSG00000169826 |
| 21 | 204299\_at | NA | SRSF10 | serine/arginine-rich splicing factor 10 | 10772 | ENSG00000188529 |
| 22 | 230170\_at | NA | OSM | oncostatin M | 5008 | ENSG00000099985 |
| 23 | 229943\_at | NA | TRIM13 | tripartite motif containing 13 | 10206 | ENSG00000204977 |
| 24 | 227900\_at | NA | CBLB | Cbl proto-oncogene, E3 ubiquitin protein ligase B | 868 | ENSG00000114423 |
| 25 | 222848\_at | NA | CENPK | centromere protein K | 64105 | ENSG00000123219 |
| 26 | 205283\_at | NA | FKTN | fukutin | 2218 | ENSG00000106692 |
| 27 | 223200\_s\_at | NA | LSG1 | large subunit GTPase 1 homolog (S. cerevisiae) | 55341 | ENSG00000041802 |
| 28 | 218401\_s\_at | NA | ZNF281 | zinc finger protein 281 | 23528 | ENSG00000162702 |
| 29 | 228785\_at | NA | ZNF281 | zinc finger protein 281 | 23528 | ENSG00000162702 |
| 30 | 202861\_at | NA | PER1 | period homolog 1 (Drosophila) | 5187 | ENSG00000179094 |
| 31 | 205900\_at | NA | KRT1 | keratin 1 | 3848 | ENSG00000167768 |
| 32 | 201088\_at | NA | KPNA2 | karyopherin alpha 2 (RAG cohort 1, importin alpha 1) | 3838 | ENSG00000182481 |
| 33 | 229431\_at | NA | RFXAP | regulatory factor X-associated protein | 5994 | ENSG00000133111 |
| 34 | 36711\_at | NA | MAFF | v-maf musculoaponeurotic fibrosarcoma oncogene homolog F (avian) | 23764 | ENSG00000185022 |
| 35 | 212847\_at | NA | FUBP1 | far upstream element (FUSE) binding protein 1 | 8880 | ENSG00000162613 |
| 36 | 227391\_x\_at | NA | LRRFIP1 | leucine rich repeat (in FLII) interacting protein 1 | 9208 | ENSG00000124831 |
| 37 | 222876\_s\_at | NA | ADAP2 | ArfGAP with dual PH domains 2 | 55803 | ENSG00000184060 |
| 38 | 202375\_at | NA | SEC24D | SEC24 family, member D (S. cerevisiae) | 9871 | ENSG00000150961 |
| 39 | 214917\_at | NA | PRKAA1 | protein kinase, AMP-activated, alpha 1 catalytic subunit | 5562 | ENSG00000132356 |
| 40 | 204614\_at | NA | SERPINB2 | serpin peptidase inhibitor, clade B (ovalbumin), member 2 | 5055 | ENSG00000197632 |
| 41 | 217739\_s\_at | NA | NAMPT | nicotinamide phosphoribosyltransferase | 10135 | ENSG00000105835 |
| 42 | 202887\_s\_at | NA | DDIT4 | DNA-damage-inducible transcript 4 | 54541 | ENSG00000168209 |
| 43 | 202904\_s\_at | NA | LSM5 | LSM5 homolog, U6 small nuclear RNA associated (S. cerevisiae) | 23658 | ENSG00000106355 |
| 44 | 203552\_at | NA | MAP4K5 | mitogen-activated protein kinase kinase kinase kinase 5 | 11183 | ENSG00000012983 |
| 45 | 222765\_x\_at | NA | ESF1 | ESF1, nucleolar pre-rRNA processing protein, homolog (S. cerevisiae) | 51575 | ENSG00000089048 |
| 46 | 208078\_s\_at | NA | SIK1 | salt-inducible kinase 1 | 150094 | ENSG00000142178 |
| 47 | 1555962\_at | NA | B3GNT7 | UDP-GlcNAc:betaGal beta-1,3-N-acetylglucosaminyltransferase 7 | 93010 | ENSG00000156966 |
| 48 | 1555274\_a\_at | NA | EPT1 | ethanolaminephosphotransferase 1 (CDP-ethanolamine-specific) | 85465 | ENSG00000138018 |
| 49 | 202498\_s\_at | NA | SLC2A3 | solute carrier family 2 (facilitated glucose transporter), member 3 | 6515 | ENSG00000059804 |
| 50 | 226279\_at | NA | PRSS23 | protease, serine, 23 | 11098 | ENSG00000150687 |
| 51 | 238199\_x\_at | NA | COX3 | cytochrome c oxidase III | 4514 | NULL |
| 52 | 1555878\_at | NA | RPS24 | ribosomal protein S24 | 6229 | ENSG00000138326 |
| 53 | 223266\_at | NA | STRADB | STE20-related kinase adaptor beta | 55437 | ENSG00000082146 |
| 54 | 231863\_at | NA | ING3 | inhibitor of growth family, member 3 | 54556 | ENSG00000071243 |
| 55 | 211560\_s\_at | NA | ALAS2 | aminolevulinate, delta-, synthase 2 | 212 | ENSG00000158578 |
| 56 | 205063\_at | NA | GEMIN2 | gem (nuclear organelle) associated protein 2 | 8487 | ENSG00000092208 |
| 57 | 203634\_s\_at | NA | CPT1A | carnitine palmitoyltransferase 1A (liver) | 1374 | ENSG00000110090 |
| 58 | 204995\_at | NA | CDK5R1 | cyclin-dependent kinase 5, regulatory subunit 1 (p35) | 8851 | ENSG00000176749 |
| 59 | 228063\_s\_at | NA | NAP1L5 | nucleosome assembly protein 1-like 5 | 266812 | ENSG00000177432 |
| 60 | 228062\_at | NA | NAP1L5 | nucleosome assembly protein 1-like 5 | 266812 | ENSG00000177432 |
| 61 | 218411\_s\_at | NA | MBIP | MAP3K12 binding inhibitory protein 1 | 51562 | ENSG00000151332 |
| 62 | 203574\_at | NA | NFIL3 | nuclear factor, interleukin 3 regulated | 4783 | ENSG00000165030 |
| 63 | 205767\_at | NA | EREG | epiregulin | 2069 | ENSG00000124882 |
| 64 | 202232\_s\_at | NA | EIF3M | eukaryotic translation initiation factor 3, subunit M | 10480 | ENSG00000149100 |
| 65 | 230559\_x\_at | NA | FGD4 | FYVE, RhoGEF and PH domain containing 4 | 121512 | ENSG00000139132 |
| 66 | 238614\_x\_at | NA | ZNF430 | zinc finger protein 430 | 80264 | ENSG00000118620 |
| 67 | 216248\_s\_at | NA | NR4A2 | nuclear receptor subfamily 4, group A, member 2 | 4929 | ENSG00000153234 |
| 68 | 204621\_s\_at | NA | NR4A2 | nuclear receptor subfamily 4, group A, member 2 | 4929 | ENSG00000153234 |
| 69 | 204622\_x\_at | NA | NR4A2 | nuclear receptor subfamily 4, group A, member 2 | 4929 | ENSG00000153234 |
| 70 | 232044\_at | NA | RBBP6 | retinoblastoma binding protein 6 | 5930 | ENSG00000122257 |
| 71 | 200608\_s\_at | NA | RAD21 | RAD21 homolog (S. pombe) | 5885 | ENSG00000164754 |
| 72 | 202221\_s\_at | NA | EP300 | E1A binding protein p300 | 2033 | ENSG00000100393 |
| 73 | 206919\_at | NA | ELK4 | ELK4, ETS-domain protein (SRF accessory protein 1) | 2005 | ENSG00000158711 |
| 74 | 208121\_s\_at | NA | PTPRO | protein tyrosine phosphatase, receptor type, O | 5800 | ENSG00000151490 |
| 75 | 201304\_at | NA | NDUFA5 | NADH dehydrogenase (ubiquinone) 1 alpha subcomplex, 5, 13kDa | 4698 | ENSG00000128609 |
| 76 | 203821\_at | NA | HBEGF | heparin-binding EGF-like growth factor | 1839 | ENSG00000113070 |
| 77 | 225760\_at | NA | MYSM1 | Myb-like, SWIRM and MPN domains 1 | 114803 | ENSG00000162601 |
| 78 | 222310\_at | NA | SCAF4 | SR-related CTD-associated factor 4 | 57466 | ENSG00000156304 |
| 79 | 213725\_x\_at | NA | XYLT1 | xylosyltransferase I | 64131 | ENSG00000103489 |
| 80 | 224453\_s\_at | NA | ETNK1 | ethanolamine kinase 1 | 55500 | ENSG00000139163 |
| 81 | 225290\_at | NA | ETNK1 | ethanolamine kinase 1 | 55500 | ENSG00000139163 |
| 82 | 202979\_s\_at | NA | CREBZF | CREB/ATF bZIP transcription factor | 58487 | ENSG00000137504 |
| 83 | 204187\_at | NA | GMPR | guanosine monophosphate reductase | 2766 | ENSG00000137198 |
| 84 | 233019\_at | NA | CNOT7 | CCR4-NOT transcription complex, subunit 7 | 29883 | ENSG00000198791 |
| 85 | 213225\_at | NA | PPM1B | protein phosphatase, Mg2+/Mn2+ dependent, 1B | 5495 | ENSG00000138032 |
| 86 | 200602\_at | NA | APP | amyloid beta (A4) precursor protein | 351 | ENSG00000142192 |
| 87 | 225916\_at | NA | ZNF131 | zinc finger protein 131 | 7690 | ENSG00000172262 |
| 88 | 214741\_at | NA | ZNF131 | zinc finger protein 131 | 7690 | ENSG00000172262 |
| 89 | 201177\_s\_at | NA | UBA2 | ubiquitin-like modifier activating enzyme 2 | 10054 | ENSG00000126261 |
| 90 | 227375\_at | NA | ANKRD13C | ankyrin repeat domain 13C | 81573 | ENSG00000118454 |
| 91 | 202422\_s\_at | NA | ACSL4 | acyl-CoA synthetase long-chain family member 4 | 2182 | ENSG00000068366 |
| 92 | 214590\_s\_at | NA | UBE2D1 | ubiquitin-conjugating enzyme E2D 1 | 7321 | ENSG00000072401 |
| 93 | 221768\_at | NA | SFPQ | splicing factor proline/glutamine-rich | 6421 | ENSG00000116560 |
| 94 | 211698\_at | NA | EID1 | EP300 interacting inhibitor of differentiation 1 | 23741 | ENSG00000255302 |
| 95 | 202464\_s\_at | NA | PFKFB3 | 6-phosphofructo-2-kinase/fructose-2,6-biphosphatase 3 | 5209 | ENSG00000170525 |
| 96 | 236539\_at | NA | PTPN22 | protein tyrosine phosphatase, non-receptor type 22 (lymphoid) | 26191 | ENSG00000134242 |
| 97 | 226472\_at | NA | PPIL4 | peptidylprolyl isomerase (cyclophilin)-like 4 | 85313 | ENSG00000131013 |
| 98 | 227697\_at | NA | SOCS3 | suppressor of cytokine signaling 3 | 9021 | ENSG00000184557 |
| 99 | 211546\_x\_at | NA | SNCA | synuclein, alpha (non A4 component of amyloid precursor) | 6622 | ENSG00000145335 |
| 100 | 207827\_x\_at | NA | SNCA | synuclein, alpha (non A4 component of amyloid precursor) | 6622 | ENSG00000145335 |
| 101 | 236081\_at | NA | SNCA | synuclein, alpha (non A4 component of amyloid precursor) | 6622 | ENSG00000145335 |
| 102 | 204467\_s\_at | NA | SNCA | synuclein, alpha (non A4 component of amyloid precursor) | 6622 | ENSG00000145335 |
| 103 | 204466\_s\_at | NA | SNCA | synuclein, alpha (non A4 component of amyloid precursor) | 6622 | ENSG00000145335 |
| 104 | 213998\_s\_at | NA | DDX17 | DEAD (Asp-Glu-Ala-Asp) box helicase 17 | 10521 | ENSG00000100201 |
| 105 | 208719\_s\_at | NA | DDX17 | DEAD (Asp-Glu-Ala-Asp) box helicase 17 | 10521 | ENSG00000100201 |
| 106 | 208151\_x\_at | NA | DDX17 | DEAD (Asp-Glu-Ala-Asp) box helicase 17 | 10521 | ENSG00000100201 |
| 107 | 224009\_x\_at | NA | DHRS9 | dehydrogenase/reductase (SDR family) member 9 | 10170 | ENSG00000073737 |
| 108 | 223952\_x\_at | NA | DHRS9 | dehydrogenase/reductase (SDR family) member 9 | 10170 | ENSG00000073737 |
| 109 | 213524\_s\_at | NA | G0S2 | G0/G1switch 2 | 50486 | ENSG00000123689 |
| 110 | 201407\_s\_at | NA | PPP1CB | protein phosphatase 1, catalytic subunit, beta isozyme | 5500 | ENSG00000213639 |
| 111 | 1553581\_s\_at | NA | SREK1IP1 | SREK1-interacting protein 1 | 285672 | ENSG00000153006 |
| 112 | 223649\_s\_at | NA | SLC25A39 | solute carrier family 25, member 39 | 51629 | ENSG00000013306 |
| 113 | 203603\_s\_at | NA | ZEB2 | zinc finger E-box binding homeobox 2 | 9839 | ENSG00000169554 |
| 114 | 204794\_at | NA | DUSP2 | dual specificity phosphatase 2 | 1844 | ENSG00000158050 |
| 115 | 236140\_at | NA | GCLM | glutamate-cysteine ligase, modifier subunit | 2730 | ENSG00000023909 |
| 116 | 235412\_at | NA | ARHGEF7 | Rho guanine nucleotide exchange factor (GEF) 7 | 8874 | ENSG00000102606 |
| 117 | 206108\_s\_at | NA | SRSF6 | serine/arginine-rich splicing factor 6 | 6431 | ENSG00000124193 |
| 118 | 226366\_at | NA | SHPRH | SNF2 histone linker PHD RING helicase, E3 ubiquitin protein ligase | 257218 | ENSG00000146414 |
| 119 | 212989\_at | NA | SGMS1 | sphingomyelin synthase 1 | 259230 | ENSG00000198964 |
| 120 | 213786\_at | NA | TAX1BP1 | Tax1 (human T-cell leukemia virus type I) binding protein 1 | 8887 | ENSG00000106052 |
| 121 | 1554309\_at | NA | EIF4G3 | eukaryotic translation initiation factor 4 gamma, 3 | 8672 | ENSG00000075151 |
| 122 | 220018\_at | NA | CBLL1 | Cbl proto-oncogene, E3 ubiquitin protein ligase-like 1 | 79872 | ENSG00000105879 |
| 123 | 210746\_s\_at | NA | EPB42 | erythrocyte membrane protein band 4.2 | 2038 | ENSG00000166947 |
| 124 | 202843\_at | NA | DNAJB9 | DnaJ (Hsp40) homolog, subfamily B, member 9 | 4189 | ENSG00000128590 |
| 125 | 216834\_at | NA | RGS1 | regulator of G-protein signaling 1 | 5996 | ENSG00000090104 |
| 126 | 202988\_s\_at | NA | RGS1 | regulator of G-protein signaling 1 | 5996 | ENSG00000090104 |
| 127 | 208762\_at | NA | SUMO1 | SMT3 suppressor of mif two 3 homolog 1 (S. cerevisiae) | 7341 | ENSG00000116030 |
| 128 | 203358\_s\_at | NA | EZH2 | enhancer of zeste homolog 2 (Drosophila) | 2146 | ENSG00000106462 |
| 129 | 1558233\_s\_at | NA | ATF1 | activating transcription factor 1 | 466 | ENSG00000123268 |
| 130 | 216929\_x\_at | NA | ABO | ABO blood group (transferase A, alpha 1-3-N-acetylgalactosaminyltransferase; transferase B, alpha 1-3-galactosyltransferase) | 28 | NULL |
| 131 | 204176\_at | NA | KLHL20 | kelch-like 20 (Drosophila) | 27252 | ENSG00000076321 |
| 132 | 1569136\_at | NA | MGAT4A | mannosyl (alpha-1,3-)-glycoprotein beta-1,4-N-acetylglucosaminyltransferase, isozyme A | 11320 | ENSG00000071073 |
| 133 | 222858\_s\_at | NA | DAPP1 | dual adaptor of phosphotyrosine and 3-phosphoinositides | 27071 | ENSG00000070190 |
| 134 | 212637\_s\_at | NA | WWP1 | WW domain containing E3 ubiquitin protein ligase 1 | 11059 | ENSG00000123124 |
| 135 | 203543\_s\_at | NA | KLF9 | Kruppel-like factor 9 | 687 | ENSG00000119138 |
| 136 | 212579\_at | NA | SMCHD1 | structural maintenance of chromosomes flexible hinge domain containing 1 | 23347 | ENSG00000101596 |
| 137 | 215716\_s\_at | NA | ATP2B1 | ATPase, Ca++ transporting, plasma membrane 1 | 490 | ENSG00000070961 |
| 138 | 202644\_s\_at | NA | TNFAIP3 | tumor necrosis factor, alpha-induced protein 3 | 7128 | ENSG00000118503 |
| 139 | 202203\_s\_at | NA | AMFR | autocrine motility factor receptor, E3 ubiquitin protein ligase | 267 | ENSG00000159461 |
| 140 | 242960\_at | NA | EPC2 | enhancer of polycomb homolog 2 (Drosophila) | 26122 | ENSG00000135999 |
| 141 | 207794\_at | NA | CCR2 | chemokine (C-C motif) receptor 2 | 729230 | ENSG00000121807 |
| 142 | 202933\_s\_at | NA | YES1 | v-yes-1 Yamaguchi sarcoma viral oncogene homolog 1 | 7525 | ENSG00000176105 |
| 143 | 222317\_at | NA | PDE3B | phosphodiesterase 3B, cGMP-inhibited | 5140 | ENSG00000152270 |
| 144 | 1557257\_at | NA | BCL10 | B-cell CLL/lymphoma 10 | 8915 | ENSG00000142867 |
| 145 | 201091\_s\_at | NA | CBX3 | chromobox homolog 3 | 11335 | ENSG00000122565 |
| 146 | 1559975\_at | NA | BTG1 | B-cell translocation gene 1, anti-proliferative | 694 | ENSG00000133639 |
| 147 | 1555476\_at | NA | IREB2 | iron-responsive element binding protein 2 | 3658 | ENSG00000136381 |
| 148 | 239143\_x\_at | NA | RNF138 | ring finger protein 138, E3 ubiquitin protein ligase | 51444 | ENSG00000134758 |
| 149 | 214012\_at | NA | ERAP1 | endoplasmic reticulum aminopeptidase 1 | 51752 | ENSG00000164307 |
| 150 | 219304\_s\_at | NA | PDGFD | platelet derived growth factor D | 80310 | ENSG00000170962 |
| 151 | 212633\_at | NA | UFL1 | UFM1-specific ligase 1 | 23376 | ENSG00000014123 |
| 152 | 207300\_s\_at | NA | F7 | coagulation factor VII (serum prothrombin conversion accelerator) | 2155 | ENSG00000057593 |

  
  

| **Database:biological process      &nbspName:macromolecule metabolic process      &nbspID:GO:0043170** | | | | | | |
| --- | --- | --- | --- | --- | --- | --- |
| C=6922; O=114; E=85.07; R=1.34; rawP=4.38e-06; adjP=0.0005 | | | | | | |
| Index | UserID | Value | Gene Symbol | Gene Name | EntrezGene | Ensembl |
| 1 | 206919\_at | NA | ELK4 | ELK4, ETS-domain protein (SRF accessory protein 1) | 2005 | ENSG00000158711 |
| 2 | 202221\_s\_at | NA | EP300 | E1A binding protein p300 | 2033 | ENSG00000100393 |
| 3 | 208121\_s\_at | NA | PTPRO | protein tyrosine phosphatase, receptor type, O | 5800 | ENSG00000151490 |
| 4 | 228106\_at | NA | DCAF16 | DDB1 and CUL4 associated factor 16 | 54876 | ENSG00000163257 |
| 5 | 219312\_s\_at | NA | ZBTB10 | zinc finger and BTB domain containing 10 | 65986 | ENSG00000205189 |
| 6 | 203821\_at | NA | HBEGF | heparin-binding EGF-like growth factor | 1839 | ENSG00000113070 |
| 7 | 225760\_at | NA | MYSM1 | Myb-like, SWIRM and MPN domains 1 | 114803 | ENSG00000162601 |
| 8 | 222310\_at | NA | SCAF4 | SR-related CTD-associated factor 4 | 57466 | ENSG00000156304 |
| 9 | 213725\_x\_at | NA | XYLT1 | xylosyltransferase I | 64131 | ENSG00000103489 |
| 10 | 227449\_at | NA | EPHA4 | EPH receptor A4 | 2043 | ENSG00000116106 |
| 11 | 201016\_at | NA | EIF1AX | eukaryotic translation initiation factor 1A, X-linked | 1964 | ENSG00000173674 |
| 12 | 202979\_s\_at | NA | CREBZF | CREB/ATF bZIP transcription factor | 58487 | ENSG00000137504 |
| 13 | 235300\_x\_at | NA | RCHY1 | ring finger and CHY zinc finger domain containing 1, E3 ubiquitin protein ligase | 25898 | ENSG00000163743 |
| 14 | 207078\_at | NA | MED6 | mediator complex subunit 6 | 10001 | ENSG00000133997 |
| 15 | 233019\_at | NA | CNOT7 | CCR4-NOT transcription complex, subunit 7 | 29883 | ENSG00000198791 |
| 16 | 213225\_at | NA | PPM1B | protein phosphatase, Mg2+/Mn2+ dependent, 1B | 5495 | ENSG00000138032 |
| 17 | 200602\_at | NA | APP | amyloid beta (A4) precursor protein | 351 | ENSG00000142192 |
| 18 | 1558692\_at | NA | C1orf85 | chromosome 1 open reading frame 85 | 112770 | ENSG00000198715 |
| 19 | 203203\_s\_at | NA | KRR1 | KRR1, small subunit (SSU) processome component, homolog (yeast) | 11103 | ENSG00000111615 |
| 20 | 225916\_at | NA | ZNF131 | zinc finger protein 131 | 7690 | ENSG00000172262 |
| 21 | 214741\_at | NA | ZNF131 | zinc finger protein 131 | 7690 | ENSG00000172262 |
| 22 | 226680\_at | NA | IKZF5 | IKAROS family zinc finger 5 (Pegasus) | 64376 | ENSG00000095574 |
| 23 | 201177\_s\_at | NA | UBA2 | ubiquitin-like modifier activating enzyme 2 | 10054 | ENSG00000126261 |
| 24 | 222243\_s\_at | NA | TOB2 | transducer of ERBB2, 2 | 10766 | ENSG00000183864 |
| 25 | 238020\_at | NA | PSMC2 | proteasome (prosome, macropain) 26S subunit, ATPase, 2 | 5701 | ENSG00000161057 |
| 26 | 227375\_at | NA | ANKRD13C | ankyrin repeat domain 13C | 81573 | ENSG00000118454 |
| 27 | 213459\_at | NA | RPL37A | ribosomal protein L37a | 6168 | ENSG00000197756 |
| 28 | 205191\_at | NA | RP2 | retinitis pigmentosa 2 (X-linked recessive) | 6102 | ENSG00000102218 |
| 29 | 214590\_s\_at | NA | UBE2D1 | ubiquitin-conjugating enzyme E2D 1 | 7321 | ENSG00000072401 |
| 30 | 232068\_s\_at | NA | TLR4 | toll-like receptor 4 | 7099 | ENSG00000136869 |
| 31 | 201437\_s\_at | NA | EIF4E | eukaryotic translation initiation factor 4E | 1977 | ENSG00000151247 |
| 32 | 218871\_x\_at | NA | CSGALNACT2 | chondroitin sulfate N-acetylgalactosaminyltransferase 2 | 55454 | ENSG00000169826 |
| 33 | 221768\_at | NA | SFPQ | splicing factor proline/glutamine-rich | 6421 | ENSG00000116560 |
| 34 | 204299\_at | NA | SRSF10 | serine/arginine-rich splicing factor 10 | 10772 | ENSG00000188529 |
| 35 | 230170\_at | NA | OSM | oncostatin M | 5008 | ENSG00000099985 |
| 36 | 229943\_at | NA | TRIM13 | tripartite motif containing 13 | 10206 | ENSG00000204977 |
| 37 | 211698\_at | NA | EID1 | EP300 interacting inhibitor of differentiation 1 | 23741 | ENSG00000255302 |
| 38 | 227900\_at | NA | CBLB | Cbl proto-oncogene, E3 ubiquitin protein ligase B | 868 | ENSG00000114423 |
| 39 | 222848\_at | NA | CENPK | centromere protein K | 64105 | ENSG00000123219 |
| 40 | 236539\_at | NA | PTPN22 | protein tyrosine phosphatase, non-receptor type 22 (lymphoid) | 26191 | ENSG00000134242 |
| 41 | 205283\_at | NA | FKTN | fukutin | 2218 | ENSG00000106692 |
| 42 | 226472\_at | NA | PPIL4 | peptidylprolyl isomerase (cyclophilin)-like 4 | 85313 | ENSG00000131013 |
| 43 | 218401\_s\_at | NA | ZNF281 | zinc finger protein 281 | 23528 | ENSG00000162702 |
| 44 | 228785\_at | NA | ZNF281 | zinc finger protein 281 | 23528 | ENSG00000162702 |
| 45 | 213998\_s\_at | NA | DDX17 | DEAD (Asp-Glu-Ala-Asp) box helicase 17 | 10521 | ENSG00000100201 |
| 46 | 208719\_s\_at | NA | DDX17 | DEAD (Asp-Glu-Ala-Asp) box helicase 17 | 10521 | ENSG00000100201 |
| 47 | 208151\_x\_at | NA | DDX17 | DEAD (Asp-Glu-Ala-Asp) box helicase 17 | 10521 | ENSG00000100201 |
| 48 | 211546\_x\_at | NA | SNCA | synuclein, alpha (non A4 component of amyloid precursor) | 6622 | ENSG00000145335 |
| 49 | 207827\_x\_at | NA | SNCA | synuclein, alpha (non A4 component of amyloid precursor) | 6622 | ENSG00000145335 |
| 50 | 236081\_at | NA | SNCA | synuclein, alpha (non A4 component of amyloid precursor) | 6622 | ENSG00000145335 |
| 51 | 204467\_s\_at | NA | SNCA | synuclein, alpha (non A4 component of amyloid precursor) | 6622 | ENSG00000145335 |
| 52 | 204466\_s\_at | NA | SNCA | synuclein, alpha (non A4 component of amyloid precursor) | 6622 | ENSG00000145335 |
| 53 | 227697\_at | NA | SOCS3 | suppressor of cytokine signaling 3 | 9021 | ENSG00000184557 |
| 54 | 202861\_at | NA | PER1 | period homolog 1 (Drosophila) | 5187 | ENSG00000179094 |
| 55 | 205900\_at | NA | KRT1 | keratin 1 | 3848 | ENSG00000167768 |
| 56 | 201407\_s\_at | NA | PPP1CB | protein phosphatase 1, catalytic subunit, beta isozyme | 5500 | ENSG00000213639 |
| 57 | 201088\_at | NA | KPNA2 | karyopherin alpha 2 (RAG cohort 1, importin alpha 1) | 3838 | ENSG00000182481 |
| 58 | 1553581\_s\_at | NA | SREK1IP1 | SREK1-interacting protein 1 | 285672 | ENSG00000153006 |
| 59 | 229431\_at | NA | RFXAP | regulatory factor X-associated protein | 5994 | ENSG00000133111 |
| 60 | 36711\_at | NA | MAFF | v-maf musculoaponeurotic fibrosarcoma oncogene homolog F (avian) | 23764 | ENSG00000185022 |
| 61 | 203603\_s\_at | NA | ZEB2 | zinc finger E-box binding homeobox 2 | 9839 | ENSG00000169554 |
| 62 | 212847\_at | NA | FUBP1 | far upstream element (FUSE) binding protein 1 | 8880 | ENSG00000162613 |
| 63 | 227391\_x\_at | NA | LRRFIP1 | leucine rich repeat (in FLII) interacting protein 1 | 9208 | ENSG00000124831 |
| 64 | 204794\_at | NA | DUSP2 | dual specificity phosphatase 2 | 1844 | ENSG00000158050 |
| 65 | 202375\_at | NA | SEC24D | SEC24 family, member D (S. cerevisiae) | 9871 | ENSG00000150961 |
| 66 | 226366\_at | NA | SHPRH | SNF2 histone linker PHD RING helicase, E3 ubiquitin protein ligase | 257218 | ENSG00000146414 |
| 67 | 206108\_s\_at | NA | SRSF6 | serine/arginine-rich splicing factor 6 | 6431 | ENSG00000124193 |
| 68 | 214917\_at | NA | PRKAA1 | protein kinase, AMP-activated, alpha 1 catalytic subunit | 5562 | ENSG00000132356 |
| 69 | 213786\_at | NA | TAX1BP1 | Tax1 (human T-cell leukemia virus type I) binding protein 1 | 8887 | ENSG00000106052 |
| 70 | 220018\_at | NA | CBLL1 | Cbl proto-oncogene, E3 ubiquitin protein ligase-like 1 | 79872 | ENSG00000105879 |
| 71 | 1554309\_at | NA | EIF4G3 | eukaryotic translation initiation factor 4 gamma, 3 | 8672 | ENSG00000075151 |
| 72 | 210746\_s\_at | NA | EPB42 | erythrocyte membrane protein band 4.2 | 2038 | ENSG00000166947 |
| 73 | 202843\_at | NA | DNAJB9 | DnaJ (Hsp40) homolog, subfamily B, member 9 | 4189 | ENSG00000128590 |
| 74 | 204614\_at | NA | SERPINB2 | serpin peptidase inhibitor, clade B (ovalbumin), member 2 | 5055 | ENSG00000197632 |
| 75 | 203358\_s\_at | NA | EZH2 | enhancer of zeste homolog 2 (Drosophila) | 2146 | ENSG00000106462 |
| 76 | 208762\_at | NA | SUMO1 | SMT3 suppressor of mif two 3 homolog 1 (S. cerevisiae) | 7341 | ENSG00000116030 |
| 77 | 217739\_s\_at | NA | NAMPT | nicotinamide phosphoribosyltransferase | 10135 | ENSG00000105835 |
| 78 | 202887\_s\_at | NA | DDIT4 | DNA-damage-inducible transcript 4 | 54541 | ENSG00000168209 |
| 79 | 1558233\_s\_at | NA | ATF1 | activating transcription factor 1 | 466 | ENSG00000123268 |
| 80 | 216929\_x\_at | NA | ABO | ABO blood group (transferase A, alpha 1-3-N-acetylgalactosaminyltransferase; transferase B, alpha 1-3-galactosyltransferase) | 28 | NULL |
| 81 | 204176\_at | NA | KLHL20 | kelch-like 20 (Drosophila) | 27252 | ENSG00000076321 |
| 82 | 202904\_s\_at | NA | LSM5 | LSM5 homolog, U6 small nuclear RNA associated (S. cerevisiae) | 23658 | ENSG00000106355 |
| 83 | 203552\_at | NA | MAP4K5 | mitogen-activated protein kinase kinase kinase kinase 5 | 11183 | ENSG00000012983 |
| 84 | 1569136\_at | NA | MGAT4A | mannosyl (alpha-1,3-)-glycoprotein beta-1,4-N-acetylglucosaminyltransferase, isozyme A | 11320 | ENSG00000071073 |
| 85 | 222858\_s\_at | NA | DAPP1 | dual adaptor of phosphotyrosine and 3-phosphoinositides | 27071 | ENSG00000070190 |
| 86 | 212637\_s\_at | NA | WWP1 | WW domain containing E3 ubiquitin protein ligase 1 | 11059 | ENSG00000123124 |
| 87 | 222765\_x\_at | NA | ESF1 | ESF1, nucleolar pre-rRNA processing protein, homolog (S. cerevisiae) | 51575 | ENSG00000089048 |
| 88 | 203543\_s\_at | NA | KLF9 | Kruppel-like factor 9 | 687 | ENSG00000119138 |
| 89 | 208078\_s\_at | NA | SIK1 | salt-inducible kinase 1 | 150094 | ENSG00000142178 |
| 90 | 212579\_at | NA | SMCHD1 | structural maintenance of chromosomes flexible hinge domain containing 1 | 23347 | ENSG00000101596 |
| 91 | 1555962\_at | NA | B3GNT7 | UDP-GlcNAc:betaGal beta-1,3-N-acetylglucosaminyltransferase 7 | 93010 | ENSG00000156966 |
| 92 | 202644\_s\_at | NA | TNFAIP3 | tumor necrosis factor, alpha-induced protein 3 | 7128 | ENSG00000118503 |
| 93 | 226279\_at | NA | PRSS23 | protease, serine, 23 | 11098 | ENSG00000150687 |
| 94 | 202203\_s\_at | NA | AMFR | autocrine motility factor receptor, E3 ubiquitin protein ligase | 267 | ENSG00000159461 |
| 95 | 207794\_at | NA | CCR2 | chemokine (C-C motif) receptor 2 | 729230 | ENSG00000121807 |
| 96 | 242960\_at | NA | EPC2 | enhancer of polycomb homolog 2 (Drosophila) | 26122 | ENSG00000135999 |
| 97 | 1555878\_at | NA | RPS24 | ribosomal protein S24 | 6229 | ENSG00000138326 |
| 98 | 223266\_at | NA | STRADB | STE20-related kinase adaptor beta | 55437 | ENSG00000082146 |
| 99 | 231863\_at | NA | ING3 | inhibitor of growth family, member 3 | 54556 | ENSG00000071243 |
| 100 | 211560\_s\_at | NA | ALAS2 | aminolevulinate, delta-, synthase 2 | 212 | ENSG00000158578 |
| 101 | 202933\_s\_at | NA | YES1 | v-yes-1 Yamaguchi sarcoma viral oncogene homolog 1 | 7525 | ENSG00000176105 |
| 102 | 205063\_at | NA | GEMIN2 | gem (nuclear organelle) associated protein 2 | 8487 | ENSG00000092208 |
| 103 | 204995\_at | NA | CDK5R1 | cyclin-dependent kinase 5, regulatory subunit 1 (p35) | 8851 | ENSG00000176749 |
| 104 | 1557257\_at | NA | BCL10 | B-cell CLL/lymphoma 10 | 8915 | ENSG00000142867 |
| 105 | 201091\_s\_at | NA | CBX3 | chromobox homolog 3 | 11335 | ENSG00000122565 |
| 106 | 228063\_s\_at | NA | NAP1L5 | nucleosome assembly protein 1-like 5 | 266812 | ENSG00000177432 |
| 107 | 228062\_at | NA | NAP1L5 | nucleosome assembly protein 1-like 5 | 266812 | ENSG00000177432 |
| 108 | 1559975\_at | NA | BTG1 | B-cell translocation gene 1, anti-proliferative | 694 | ENSG00000133639 |
| 109 | 218411\_s\_at | NA | MBIP | MAP3K12 binding inhibitory protein 1 | 51562 | ENSG00000151332 |
| 110 | 1555476\_at | NA | IREB2 | iron-responsive element binding protein 2 | 3658 | ENSG00000136381 |
| 111 | 203574\_at | NA | NFIL3 | nuclear factor, interleukin 3 regulated | 4783 | ENSG00000165030 |
| 112 | 239143\_x\_at | NA | RNF138 | ring finger protein 138, E3 ubiquitin protein ligase | 51444 | ENSG00000134758 |
| 113 | 205767\_at | NA | EREG | epiregulin | 2069 | ENSG00000124882 |
| 114 | 202232\_s\_at | NA | EIF3M | eukaryotic translation initiation factor 3, subunit M | 10480 | ENSG00000149100 |
| 115 | 214012\_at | NA | ERAP1 | endoplasmic reticulum aminopeptidase 1 | 51752 | ENSG00000164307 |
| 116 | 219304\_s\_at | NA | PDGFD | platelet derived growth factor D | 80310 | ENSG00000170962 |
| 117 | 230559\_x\_at | NA | FGD4 | FYVE, RhoGEF and PH domain containing 4 | 121512 | ENSG00000139132 |
| 118 | 238614\_x\_at | NA | ZNF430 | zinc finger protein 430 | 80264 | ENSG00000118620 |
| 119 | 216248\_s\_at | NA | NR4A2 | nuclear receptor subfamily 4, group A, member 2 | 4929 | ENSG00000153234 |
| 120 | 204621\_s\_at | NA | NR4A2 | nuclear receptor subfamily 4, group A, member 2 | 4929 | ENSG00000153234 |
| 121 | 204622\_x\_at | NA | NR4A2 | nuclear receptor subfamily 4, group A, member 2 | 4929 | ENSG00000153234 |
| 122 | 212633\_at | NA | UFL1 | UFM1-specific ligase 1 | 23376 | ENSG00000014123 |
| 123 | 207300\_s\_at | NA | F7 | coagulation factor VII (serum prothrombin conversion accelerator) | 2155 | ENSG00000057593 |
| 124 | 232044\_at | NA | RBBP6 | retinoblastoma binding protein 6 | 5930 | ENSG00000122257 |
| 125 | 200608\_s\_at | NA | RAD21 | RAD21 homolog (S. pombe) | 5885 | ENSG00000164754 |

  
  

| **Database:molecular function      &nbspName:kinase binding      &nbspID:GO:0019900** | | | | | | |
| --- | --- | --- | --- | --- | --- | --- |
| C=393; O=17; E=4.69; R=3.63; rawP=4.68e-06; adjP=0.0011 | | | | | | |
| Index | UserID | Value | Gene Symbol | Gene Name | EntrezGene | Ensembl |
| 1 | 202221\_s\_at | NA | EP300 | E1A binding protein p300 | 2033 | ENSG00000100393 |
| 2 | 204794\_at | NA | DUSP2 | dual specificity phosphatase 2 | 1844 | ENSG00000158050 |
| 3 | 202644\_s\_at | NA | TNFAIP3 | tumor necrosis factor, alpha-induced protein 3 | 7128 | ENSG00000118503 |
| 4 | 235412\_at | NA | ARHGEF7 | Rho guanine nucleotide exchange factor (GEF) 7 | 8874 | ENSG00000102606 |
| 5 | 213786\_at | NA | TAX1BP1 | Tax1 (human T-cell leukemia virus type I) binding protein 1 | 8887 | ENSG00000106052 |
| 6 | 227900\_at | NA | CBLB | Cbl proto-oncogene, E3 ubiquitin protein ligase B | 868 | ENSG00000114423 |
| 7 | 236539\_at | NA | PTPN22 | protein tyrosine phosphatase, non-receptor type 22 (lymphoid) | 26191 | ENSG00000134242 |
| 8 | 222317\_at | NA | PDE3B | phosphodiesterase 3B, cGMP-inhibited | 5140 | ENSG00000152270 |
| 9 | 213404\_s\_at | NA | RHEB | Ras homolog enriched in brain | 6009 | ENSG00000106615 |
| 10 | 204995\_at | NA | CDK5R1 | cyclin-dependent kinase 5, regulatory subunit 1 (p35) | 8851 | ENSG00000176749 |
| 11 | 1557257\_at | NA | BCL10 | B-cell CLL/lymphoma 10 | 8915 | ENSG00000142867 |
| 12 | 1559975\_at | NA | BTG1 | B-cell translocation gene 1, anti-proliferative | 694 | ENSG00000133639 |
| 13 | 1555960\_at | NA | HINT1 | histidine triad nucleotide binding protein 1 | 3094 | ENSG00000169567 |
| 14 | 203884\_s\_at | NA | RAB11FIP2 | RAB11 family interacting protein 2 (class I) | 22841 | ENSG00000107560 |
| 15 | 239143\_x\_at | NA | RNF138 | ring finger protein 138, E3 ubiquitin protein ligase | 51444 | ENSG00000134758 |
| 16 | 201407\_s\_at | NA | PPP1CB | protein phosphatase 1, catalytic subunit, beta isozyme | 5500 | ENSG00000213639 |
| 17 | 208078\_s\_at | NA | SIK1 | salt-inducible kinase 1 | 150094 | ENSG00000142178 |

  
  

| **Database:molecular function      &nbspName:enzyme binding      &nbspID:GO:0019899** | | | | | | |
| --- | --- | --- | --- | --- | --- | --- |
| C=1062; O=27; E=12.67; R=2.13; rawP=0.0001; adjP=0.0038 | | | | | | |
| Index | UserID | Value | Gene Symbol | Gene Name | EntrezGene | Ensembl |
| 1 | 202221\_s\_at | NA | EP300 | E1A binding protein p300 | 2033 | ENSG00000100393 |
| 2 | 236140\_at | NA | GCLM | glutamate-cysteine ligase, modifier subunit | 2730 | ENSG00000023909 |
| 3 | 204794\_at | NA | DUSP2 | dual specificity phosphatase 2 | 1844 | ENSG00000158050 |
| 4 | 202644\_s\_at | NA | TNFAIP3 | tumor necrosis factor, alpha-induced protein 3 | 7128 | ENSG00000118503 |
| 5 | 211698\_at | NA | EID1 | EP300 interacting inhibitor of differentiation 1 | 23741 | ENSG00000255302 |
| 6 | 235412\_at | NA | ARHGEF7 | Rho guanine nucleotide exchange factor (GEF) 7 | 8874 | ENSG00000102606 |
| 7 | 213786\_at | NA | TAX1BP1 | Tax1 (human T-cell leukemia virus type I) binding protein 1 | 8887 | ENSG00000106052 |
| 8 | 227900\_at | NA | CBLB | Cbl proto-oncogene, E3 ubiquitin protein ligase B | 868 | ENSG00000114423 |
| 9 | 202933\_s\_at | NA | YES1 | v-yes-1 Yamaguchi sarcoma viral oncogene homolog 1 | 7525 | ENSG00000176105 |
| 10 | 236539\_at | NA | PTPN22 | protein tyrosine phosphatase, non-receptor type 22 (lymphoid) | 26191 | ENSG00000134242 |
| 11 | 222317\_at | NA | PDE3B | phosphodiesterase 3B, cGMP-inhibited | 5140 | ENSG00000152270 |
| 12 | 213404\_s\_at | NA | RHEB | Ras homolog enriched in brain | 6009 | ENSG00000106615 |
| 13 | 204995\_at | NA | CDK5R1 | cyclin-dependent kinase 5, regulatory subunit 1 (p35) | 8851 | ENSG00000176749 |
| 14 | 1557257\_at | NA | BCL10 | B-cell CLL/lymphoma 10 | 8915 | ENSG00000142867 |
| 15 | 201091\_s\_at | NA | CBX3 | chromobox homolog 3 | 11335 | ENSG00000122565 |
| 16 | 208762\_at | NA | SUMO1 | SMT3 suppressor of mif two 3 homolog 1 (S. cerevisiae) | 7341 | ENSG00000116030 |
| 17 | 1559975\_at | NA | BTG1 | B-cell translocation gene 1, anti-proliferative | 694 | ENSG00000133639 |
| 18 | 1555960\_at | NA | HINT1 | histidine triad nucleotide binding protein 1 | 3094 | ENSG00000169567 |
| 19 | 203884\_s\_at | NA | RAB11FIP2 | RAB11 family interacting protein 2 (class I) | 22841 | ENSG00000107560 |
| 20 | 209451\_at | NA | TANK | TRAF family member-associated NFKB activator | 10010 | ENSG00000136560 |
| 21 | 239143\_x\_at | NA | RNF138 | ring finger protein 138, E3 ubiquitin protein ligase | 51444 | ENSG00000134758 |
| 22 | 230379\_x\_at | NA | C2orf56 | chromosome 2 open reading frame 56 | 55471 | ENSG00000003509 |
| 23 | 230559\_x\_at | NA | FGD4 | FYVE, RhoGEF and PH domain containing 4 | 121512 | ENSG00000139132 |
| 24 | 201407\_s\_at | NA | PPP1CB | protein phosphatase 1, catalytic subunit, beta isozyme | 5500 | ENSG00000213639 |
| 25 | 201088\_at | NA | KPNA2 | karyopherin alpha 2 (RAG cohort 1, importin alpha 1) | 3838 | ENSG00000182481 |
| 26 | 208078\_s\_at | NA | SIK1 | salt-inducible kinase 1 | 150094 | ENSG00000142178 |
| 27 | 242109\_at | NA | SYTL3 | synaptotagmin-like 3 | 94120 | ENSG00000164674 |

  
  

| **Database:molecular function      &nbspName:ligase activity      &nbspID:GO:0016874** | | | | | | |
| --- | --- | --- | --- | --- | --- | --- |
| C=474; O=17; E=5.66; R=3.01; rawP=5.23e-05; adjP=0.0038 | | | | | | |
| Index | UserID | Value | Gene Symbol | Gene Name | EntrezGene | Ensembl |
| 1 | 236140\_at | NA | GCLM | glutamate-cysteine ligase, modifier subunit | 2730 | ENSG00000023909 |
| 2 | 229943\_at | NA | TRIM13 | tripartite motif containing 13 | 10206 | ENSG00000204977 |
| 3 | 202644\_s\_at | NA | TNFAIP3 | tumor necrosis factor, alpha-induced protein 3 | 7128 | ENSG00000118503 |
| 4 | 202203\_s\_at | NA | AMFR | autocrine motility factor receptor, E3 ubiquitin protein ligase | 267 | ENSG00000159461 |
| 5 | 226366\_at | NA | SHPRH | SNF2 histone linker PHD RING helicase, E3 ubiquitin protein ligase | 257218 | ENSG00000146414 |
| 6 | 227900\_at | NA | CBLB | Cbl proto-oncogene, E3 ubiquitin protein ligase B | 868 | ENSG00000114423 |
| 7 | 235300\_x\_at | NA | RCHY1 | ring finger and CHY zinc finger domain containing 1, E3 ubiquitin protein ligase | 25898 | ENSG00000163743 |
| 8 | 220018\_at | NA | CBLL1 | Cbl proto-oncogene, E3 ubiquitin protein ligase-like 1 | 79872 | ENSG00000105879 |
| 9 | 208762\_at | NA | SUMO1 | SMT3 suppressor of mif two 3 homolog 1 (S. cerevisiae) | 7341 | ENSG00000116030 |
| 10 | 204176\_at | NA | KLHL20 | kelch-like 20 (Drosophila) | 27252 | ENSG00000076321 |
| 11 | 201177\_s\_at | NA | UBA2 | ubiquitin-like modifier activating enzyme 2 | 10054 | ENSG00000126261 |
| 12 | 239143\_x\_at | NA | RNF138 | ring finger protein 138, E3 ubiquitin protein ligase | 51444 | ENSG00000134758 |
| 13 | 212637\_s\_at | NA | WWP1 | WW domain containing E3 ubiquitin protein ligase 1 | 11059 | ENSG00000123124 |
| 14 | 212633\_at | NA | UFL1 | UFM1-specific ligase 1 | 23376 | ENSG00000014123 |
| 15 | 202422\_s\_at | NA | ACSL4 | acyl-CoA synthetase long-chain family member 4 | 2182 | ENSG00000068366 |
| 16 | 214590\_s\_at | NA | UBE2D1 | ubiquitin-conjugating enzyme E2D 1 | 7321 | ENSG00000072401 |
| 17 | 232044\_at | NA | RBBP6 | retinoblastoma binding protein 6 | 5930 | ENSG00000122257 |

  
  

| **Database:molecular function      &nbspName:ligase activity, forming carbon-nitrogen bonds      &nbspID:GO:0016879** | | | | | | |
| --- | --- | --- | --- | --- | --- | --- |
| C=325; O=13; E=3.88; R=3.35; rawP=0.0001; adjP=0.0038 | | | | | | |
| Index | UserID | Value | Gene Symbol | Gene Name | EntrezGene | Ensembl |
| 1 | 236140\_at | NA | GCLM | glutamate-cysteine ligase, modifier subunit | 2730 | ENSG00000023909 |
| 2 | 229943\_at | NA | TRIM13 | tripartite motif containing 13 | 10206 | ENSG00000204977 |
| 3 | 202644\_s\_at | NA | TNFAIP3 | tumor necrosis factor, alpha-induced protein 3 | 7128 | ENSG00000118503 |
| 4 | 204176\_at | NA | KLHL20 | kelch-like 20 (Drosophila) | 27252 | ENSG00000076321 |
| 5 | 202203\_s\_at | NA | AMFR | autocrine motility factor receptor, E3 ubiquitin protein ligase | 267 | ENSG00000159461 |
| 6 | 227900\_at | NA | CBLB | Cbl proto-oncogene, E3 ubiquitin protein ligase B | 868 | ENSG00000114423 |
| 7 | 235300\_x\_at | NA | RCHY1 | ring finger and CHY zinc finger domain containing 1, E3 ubiquitin protein ligase | 25898 | ENSG00000163743 |
| 8 | 220018\_at | NA | CBLL1 | Cbl proto-oncogene, E3 ubiquitin protein ligase-like 1 | 79872 | ENSG00000105879 |
| 9 | 212637\_s\_at | NA | WWP1 | WW domain containing E3 ubiquitin protein ligase 1 | 11059 | ENSG00000123124 |
| 10 | 212633\_at | NA | UFL1 | UFM1-specific ligase 1 | 23376 | ENSG00000014123 |
| 11 | 214590\_s\_at | NA | UBE2D1 | ubiquitin-conjugating enzyme E2D 1 | 7321 | ENSG00000072401 |
| 12 | 232044\_at | NA | RBBP6 | retinoblastoma binding protein 6 | 5930 | ENSG00000122257 |
| 13 | 208762\_at | NA | SUMO1 | SMT3 suppressor of mif two 3 homolog 1 (S. cerevisiae) | 7341 | ENSG00000116030 |

  
  

| **Database:molecular function      &nbspName:acid-amino acid ligase activity      &nbspID:GO:0016881** | | | | | | |
| --- | --- | --- | --- | --- | --- | --- |
| C=293; O=13; E=3.50; R=3.72; rawP=4.99e-05; adjP=0.0038 | | | | | | |
| Index | UserID | Value | Gene Symbol | Gene Name | EntrezGene | Ensembl |
| 1 | 236140\_at | NA | GCLM | glutamate-cysteine ligase, modifier subunit | 2730 | ENSG00000023909 |
| 2 | 229943\_at | NA | TRIM13 | tripartite motif containing 13 | 10206 | ENSG00000204977 |
| 3 | 202644\_s\_at | NA | TNFAIP3 | tumor necrosis factor, alpha-induced protein 3 | 7128 | ENSG00000118503 |
| 4 | 204176\_at | NA | KLHL20 | kelch-like 20 (Drosophila) | 27252 | ENSG00000076321 |
| 5 | 202203\_s\_at | NA | AMFR | autocrine motility factor receptor, E3 ubiquitin protein ligase | 267 | ENSG00000159461 |
| 6 | 227900\_at | NA | CBLB | Cbl proto-oncogene, E3 ubiquitin protein ligase B | 868 | ENSG00000114423 |
| 7 | 235300\_x\_at | NA | RCHY1 | ring finger and CHY zinc finger domain containing 1, E3 ubiquitin protein ligase | 25898 | ENSG00000163743 |
| 8 | 220018\_at | NA | CBLL1 | Cbl proto-oncogene, E3 ubiquitin protein ligase-like 1 | 79872 | ENSG00000105879 |
| 9 | 212637\_s\_at | NA | WWP1 | WW domain containing E3 ubiquitin protein ligase 1 | 11059 | ENSG00000123124 |
| 10 | 212633\_at | NA | UFL1 | UFM1-specific ligase 1 | 23376 | ENSG00000014123 |
| 11 | 214590\_s\_at | NA | UBE2D1 | ubiquitin-conjugating enzyme E2D 1 | 7321 | ENSG00000072401 |
| 12 | 232044\_at | NA | RBBP6 | retinoblastoma binding protein 6 | 5930 | ENSG00000122257 |
| 13 | 208762\_at | NA | SUMO1 | SMT3 suppressor of mif two 3 homolog 1 (S. cerevisiae) | 7341 | ENSG00000116030 |

  
  

| **Database:molecular function      &nbspName:small conjugating protein ligase activity      &nbspID:GO:0019787** | | | | | | |
| --- | --- | --- | --- | --- | --- | --- |
| C=267; O=12; E=3.19; R=3.77; rawP=8.66e-05; adjP=0.0038 | | | | | | |
| Index | UserID | Value | Gene Symbol | Gene Name | EntrezGene | Ensembl |
| 1 | 229943\_at | NA | TRIM13 | tripartite motif containing 13 | 10206 | ENSG00000204977 |
| 2 | 202644\_s\_at | NA | TNFAIP3 | tumor necrosis factor, alpha-induced protein 3 | 7128 | ENSG00000118503 |
| 3 | 204176\_at | NA | KLHL20 | kelch-like 20 (Drosophila) | 27252 | ENSG00000076321 |
| 4 | 202203\_s\_at | NA | AMFR | autocrine motility factor receptor, E3 ubiquitin protein ligase | 267 | ENSG00000159461 |
| 5 | 227900\_at | NA | CBLB | Cbl proto-oncogene, E3 ubiquitin protein ligase B | 868 | ENSG00000114423 |
| 6 | 235300\_x\_at | NA | RCHY1 | ring finger and CHY zinc finger domain containing 1, E3 ubiquitin protein ligase | 25898 | ENSG00000163743 |
| 7 | 220018\_at | NA | CBLL1 | Cbl proto-oncogene, E3 ubiquitin protein ligase-like 1 | 79872 | ENSG00000105879 |
| 8 | 212637\_s\_at | NA | WWP1 | WW domain containing E3 ubiquitin protein ligase 1 | 11059 | ENSG00000123124 |
| 9 | 212633\_at | NA | UFL1 | UFM1-specific ligase 1 | 23376 | ENSG00000014123 |
| 10 | 214590\_s\_at | NA | UBE2D1 | ubiquitin-conjugating enzyme E2D 1 | 7321 | ENSG00000072401 |
| 11 | 232044\_at | NA | RBBP6 | retinoblastoma binding protein 6 | 5930 | ENSG00000122257 |
| 12 | 208762\_at | NA | SUMO1 | SMT3 suppressor of mif two 3 homolog 1 (S. cerevisiae) | 7341 | ENSG00000116030 |

  
  

| **Database:molecular function      &nbspName:protein kinase binding      &nbspID:GO:0019901** | | | | | | |
| --- | --- | --- | --- | --- | --- | --- |
| C=355; O=13; E=4.24; R=3.07; rawP=0.0003; adjP=0.0086 | | | | | | |
| Index | UserID | Value | Gene Symbol | Gene Name | EntrezGene | Ensembl |
| 1 | 202221\_s\_at | NA | EP300 | E1A binding protein p300 | 2033 | ENSG00000100393 |
| 2 | 204794\_at | NA | DUSP2 | dual specificity phosphatase 2 | 1844 | ENSG00000158050 |
| 3 | 1555960\_at | NA | HINT1 | histidine triad nucleotide binding protein 1 | 3094 | ENSG00000169567 |
| 4 | 203884\_s\_at | NA | RAB11FIP2 | RAB11 family interacting protein 2 (class I) | 22841 | ENSG00000107560 |
| 5 | 235412\_at | NA | ARHGEF7 | Rho guanine nucleotide exchange factor (GEF) 7 | 8874 | ENSG00000102606 |
| 6 | 239143\_x\_at | NA | RNF138 | ring finger protein 138, E3 ubiquitin protein ligase | 51444 | ENSG00000134758 |
| 7 | 227900\_at | NA | CBLB | Cbl proto-oncogene, E3 ubiquitin protein ligase B | 868 | ENSG00000114423 |
| 8 | 201407\_s\_at | NA | PPP1CB | protein phosphatase 1, catalytic subunit, beta isozyme | 5500 | ENSG00000213639 |
| 9 | 208078\_s\_at | NA | SIK1 | salt-inducible kinase 1 | 150094 | ENSG00000142178 |
| 10 | 222317\_at | NA | PDE3B | phosphodiesterase 3B, cGMP-inhibited | 5140 | ENSG00000152270 |
| 11 | 213404\_s\_at | NA | RHEB | Ras homolog enriched in brain | 6009 | ENSG00000106615 |
| 12 | 204995\_at | NA | CDK5R1 | cyclin-dependent kinase 5, regulatory subunit 1 (p35) | 8851 | ENSG00000176749 |
| 13 | 1557257\_at | NA | BCL10 | B-cell CLL/lymphoma 10 | 8915 | ENSG00000142867 |

  
  

| **Database:molecular function      &nbspName:core promoter binding      &nbspID:GO:0001047** | | | | | | |
| --- | --- | --- | --- | --- | --- | --- |
| C=48; O=5; E=0.57; R=8.73; rawP=0.0003; adjP=0.0086 | | | | | | |
| Index | UserID | Value | Gene Symbol | Gene Name | EntrezGene | Ensembl |
| 1 | 206919\_at | NA | ELK4 | ELK4, ETS-domain protein (SRF accessory protein 1) | 2005 | ENSG00000158711 |
| 2 | 202221\_s\_at | NA | EP300 | E1A binding protein p300 | 2033 | ENSG00000100393 |
| 3 | 203574\_at | NA | NFIL3 | nuclear factor, interleukin 3 regulated | 4783 | ENSG00000165030 |
| 4 | 218401\_s\_at | NA | ZNF281 | zinc finger protein 281 | 23528 | ENSG00000162702 |
| 5 | 228785\_at | NA | ZNF281 | zinc finger protein 281 | 23528 | ENSG00000162702 |
| 6 | 203358\_s\_at | NA | EZH2 | enhancer of zeste homolog 2 (Drosophila) | 2146 | ENSG00000106462 |

  
  

| **Database:molecular function      &nbspName:ubiquitin-protein ligase activity      &nbspID:GO:0004842** | | | | | | |
| --- | --- | --- | --- | --- | --- | --- |
| C=251; O=10; E=2.99; R=3.34; rawP=0.0009; adjP=0.0230 | | | | | | |
| Index | UserID | Value | Gene Symbol | Gene Name | EntrezGene | Ensembl |
| 1 | 229943\_at | NA | TRIM13 | tripartite motif containing 13 | 10206 | ENSG00000204977 |
| 2 | 202644\_s\_at | NA | TNFAIP3 | tumor necrosis factor, alpha-induced protein 3 | 7128 | ENSG00000118503 |
| 3 | 204176\_at | NA | KLHL20 | kelch-like 20 (Drosophila) | 27252 | ENSG00000076321 |
| 4 | 202203\_s\_at | NA | AMFR | autocrine motility factor receptor, E3 ubiquitin protein ligase | 267 | ENSG00000159461 |
| 5 | 227900\_at | NA | CBLB | Cbl proto-oncogene, E3 ubiquitin protein ligase B | 868 | ENSG00000114423 |
| 6 | 235300\_x\_at | NA | RCHY1 | ring finger and CHY zinc finger domain containing 1, E3 ubiquitin protein ligase | 25898 | ENSG00000163743 |
| 7 | 220018\_at | NA | CBLL1 | Cbl proto-oncogene, E3 ubiquitin protein ligase-like 1 | 79872 | ENSG00000105879 |
| 8 | 212637\_s\_at | NA | WWP1 | WW domain containing E3 ubiquitin protein ligase 1 | 11059 | ENSG00000123124 |
| 9 | 214590\_s\_at | NA | UBE2D1 | ubiquitin-conjugating enzyme E2D 1 | 7321 | ENSG00000072401 |
| 10 | 232044\_at | NA | RBBP6 | retinoblastoma binding protein 6 | 5930 | ENSG00000122257 |

  
  

| **Database:molecular function      &nbspName:protein binding transcription factor activity      &nbspID:GO:0000988** | | | | | | |
| --- | --- | --- | --- | --- | --- | --- |
| C=490; O=14; E=5.85; R=2.39; rawP=0.0022; adjP=0.0506 | | | | | | |
| Index | UserID | Value | Gene Symbol | Gene Name | EntrezGene | Ensembl |
| 1 | 206919\_at | NA | ELK4 | ELK4, ETS-domain protein (SRF accessory protein 1) | 2005 | ENSG00000158711 |
| 2 | 202221\_s\_at | NA | EP300 | E1A binding protein p300 | 2033 | ENSG00000100393 |
| 3 | 1559975\_at | NA | BTG1 | B-cell translocation gene 1, anti-proliferative | 694 | ENSG00000133639 |
| 4 | 211698\_at | NA | EID1 | EP300 interacting inhibitor of differentiation 1 | 23741 | ENSG00000255302 |
| 5 | 225760\_at | NA | MYSM1 | Myb-like, SWIRM and MPN domains 1 | 114803 | ENSG00000162601 |
| 6 | 202861\_at | NA | PER1 | period homolog 1 (Drosophila) | 5187 | ENSG00000179094 |
| 7 | 203574\_at | NA | NFIL3 | nuclear factor, interleukin 3 regulated | 4783 | ENSG00000165030 |
| 8 | 207078\_at | NA | MED6 | mediator complex subunit 6 | 10001 | ENSG00000133997 |
| 9 | 208078\_s\_at | NA | SIK1 | salt-inducible kinase 1 | 150094 | ENSG00000142178 |
| 10 | 229431\_at | NA | RFXAP | regulatory factor X-associated protein | 5994 | ENSG00000133111 |
| 11 | 1558692\_at | NA | C1orf85 | chromosome 1 open reading frame 85 | 112770 | ENSG00000198715 |
| 12 | 218401\_s\_at | NA | ZNF281 | zinc finger protein 281 | 23528 | ENSG00000162702 |
| 13 | 228785\_at | NA | ZNF281 | zinc finger protein 281 | 23528 | ENSG00000162702 |
| 14 | 1557257\_at | NA | BCL10 | B-cell CLL/lymphoma 10 | 8915 | ENSG00000142867 |
| 15 | 213998\_s\_at | NA | DDX17 | DEAD (Asp-Glu-Ala-Asp) box helicase 17 | 10521 | ENSG00000100201 |
| 16 | 208719\_s\_at | NA | DDX17 | DEAD (Asp-Glu-Ala-Asp) box helicase 17 | 10521 | ENSG00000100201 |
| 17 | 208151\_x\_at | NA | DDX17 | DEAD (Asp-Glu-Ala-Asp) box helicase 17 | 10521 | ENSG00000100201 |

  
  

| **Database:cellular component      &nbspName:intracellular      &nbspID:GO:0005622** | | | | | | |
| --- | --- | --- | --- | --- | --- | --- |
| C=11842; O=163; E=141.60; R=1.15; rawP=4.86e-05; adjP=0.0050 | | | | | | |
| Index | UserID | Value | Gene Symbol | Gene Name | EntrezGene | Ensembl |
| 1 | 228106\_at | NA | DCAF16 | DDB1 and CUL4 associated factor 16 | 54876 | ENSG00000163257 |
| 2 | 214658\_at | NA | TMED7 | transmembrane emp24 protein transport domain containing 7 | 51014 | ENSG00000134970 |
| 3 | 219312\_s\_at | NA | ZBTB10 | zinc finger and BTB domain containing 10 | 65986 | ENSG00000205189 |
| 4 | 227449\_at | NA | EPHA4 | EPH receptor A4 | 2043 | ENSG00000116106 |
| 5 | 201016\_at | NA | EIF1AX | eukaryotic translation initiation factor 1A, X-linked | 1964 | ENSG00000173674 |
| 6 | 235300\_x\_at | NA | RCHY1 | ring finger and CHY zinc finger domain containing 1, E3 ubiquitin protein ligase | 25898 | ENSG00000163743 |
| 7 | 226181\_at | NA | TUBE1 | tubulin, epsilon 1 | 51175 | ENSG00000074935 |
| 8 | 207078\_at | NA | MED6 | mediator complex subunit 6 | 10001 | ENSG00000133997 |
| 9 | 218701\_at | NA | LACTB2 | lactamase, beta 2 | 51110 | ENSG00000147592 |
| 10 | 202660\_at | NA | ITPR2 | inositol 1,4,5-trisphosphate receptor, type 2 | 3709 | ENSG00000123104 |
| 11 | 222528\_s\_at | NA | SLC25A37 | solute carrier family 25 (mitochondrial iron transporter), member 37 | 51312 | ENSG00000147454 |
| 12 | 226928\_x\_at | NA | SLC25A37 | solute carrier family 25 (mitochondrial iron transporter), member 37 | 51312 | ENSG00000147454 |
| 13 | 228527\_s\_at | NA | SLC25A37 | solute carrier family 25 (mitochondrial iron transporter), member 37 | 51312 | ENSG00000147454 |
| 14 | 226179\_at | NA | SLC25A37 | solute carrier family 25 (mitochondrial iron transporter), member 37 | 51312 | ENSG00000147454 |
| 15 | 1558692\_at | NA | C1orf85 | chromosome 1 open reading frame 85 | 112770 | ENSG00000198715 |
| 16 | 203203\_s\_at | NA | KRR1 | KRR1, small subunit (SSU) processome component, homolog (yeast) | 11103 | ENSG00000111615 |
| 17 | 243916\_x\_at | NA | UBLCP1 | ubiquitin-like domain containing CTD phosphatase 1 | 134510 | ENSG00000164332 |
| 18 | 202314\_at | NA | CYP51A1 | cytochrome P450, family 51, subfamily A, polypeptide 1 | 1595 | ENSG00000001630 |
| 19 | 235683\_at | NA | SESN3 | sestrin 3 | 143686 | ENSG00000149212 |
| 20 | 226680\_at | NA | IKZF5 | IKAROS family zinc finger 5 (Pegasus) | 64376 | ENSG00000095574 |
| 21 | 238020\_at | NA | PSMC2 | proteasome (prosome, macropain) 26S subunit, ATPase, 2 | 5701 | ENSG00000161057 |
| 22 | 222243\_s\_at | NA | TOB2 | transducer of ERBB2, 2 | 10766 | ENSG00000183864 |
| 23 | 230379\_x\_at | NA | C2orf56 | chromosome 2 open reading frame 56 | 55471 | ENSG00000003509 |
| 24 | 213459\_at | NA | RPL37A | ribosomal protein L37a | 6168 | ENSG00000197756 |
| 25 | 205191\_at | NA | RP2 | retinitis pigmentosa 2 (X-linked recessive) | 6102 | ENSG00000102218 |
| 26 | 201437\_s\_at | NA | EIF4E | eukaryotic translation initiation factor 4E | 1977 | ENSG00000151247 |
| 27 | 232068\_s\_at | NA | TLR4 | toll-like receptor 4 | 7099 | ENSG00000136869 |
| 28 | 218871\_x\_at | NA | CSGALNACT2 | chondroitin sulfate N-acetylgalactosaminyltransferase 2 | 55454 | ENSG00000169826 |
| 29 | 204299\_at | NA | SRSF10 | serine/arginine-rich splicing factor 10 | 10772 | ENSG00000188529 |
| 30 | 229943\_at | NA | TRIM13 | tripartite motif containing 13 | 10206 | ENSG00000204977 |
| 31 | 227900\_at | NA | CBLB | Cbl proto-oncogene, E3 ubiquitin protein ligase B | 868 | ENSG00000114423 |
| 32 | 222848\_at | NA | CENPK | centromere protein K | 64105 | ENSG00000123219 |
| 33 | 205283\_at | NA | FKTN | fukutin | 2218 | ENSG00000106692 |
| 34 | 223200\_s\_at | NA | LSG1 | large subunit GTPase 1 homolog (S. cerevisiae) | 55341 | ENSG00000041802 |
| 35 | 218401\_s\_at | NA | ZNF281 | zinc finger protein 281 | 23528 | ENSG00000162702 |
| 36 | 228785\_at | NA | ZNF281 | zinc finger protein 281 | 23528 | ENSG00000162702 |
| 37 | 213404\_s\_at | NA | RHEB | Ras homolog enriched in brain | 6009 | ENSG00000106615 |
| 38 | 213418\_at | NA | HSPA6 | heat shock 70kDa protein 6 (HSP70B') | 3310 | ENSG00000173110 |
| 39 | 205900\_at | NA | KRT1 | keratin 1 | 3848 | ENSG00000167768 |
| 40 | 202861\_at | NA | PER1 | period homolog 1 (Drosophila) | 5187 | ENSG00000179094 |
| 41 | 203310\_at | NA | STXBP3 | syntaxin binding protein 3 | 6814 | ENSG00000116266 |
| 42 | 201088\_at | NA | KPNA2 | karyopherin alpha 2 (RAG cohort 1, importin alpha 1) | 3838 | ENSG00000182481 |
| 43 | 1553132\_a\_at | NA | TC2N | tandem C2 domains, nuclear | 123036 | ENSG00000165929 |
| 44 | 36711\_at | NA | MAFF | v-maf musculoaponeurotic fibrosarcoma oncogene homolog F (avian) | 23764 | ENSG00000185022 |
| 45 | 229431\_at | NA | RFXAP | regulatory factor X-associated protein | 5994 | ENSG00000133111 |
| 46 | 207545\_s\_at | NA | NUMB | numb homolog (Drosophila) | 8650 | ENSG00000133961 |
| 47 | 212847\_at | NA | FUBP1 | far upstream element (FUSE) binding protein 1 | 8880 | ENSG00000162613 |
| 48 | 240336\_at | NA | HBM | hemoglobin, mu | 3042 | ENSG00000206177 |
| 49 | 227391\_x\_at | NA | LRRFIP1 | leucine rich repeat (in FLII) interacting protein 1 | 9208 | ENSG00000124831 |
| 50 | 222876\_s\_at | NA | ADAP2 | ArfGAP with dual PH domains 2 | 55803 | ENSG00000184060 |
| 51 | 202375\_at | NA | SEC24D | SEC24 family, member D (S. cerevisiae) | 9871 | ENSG00000150961 |
| 52 | 214917\_at | NA | PRKAA1 | protein kinase, AMP-activated, alpha 1 catalytic subunit | 5562 | ENSG00000132356 |
| 53 | 204614\_at | NA | SERPINB2 | serpin peptidase inhibitor, clade B (ovalbumin), member 2 | 5055 | ENSG00000197632 |
| 54 | 217739\_s\_at | NA | NAMPT | nicotinamide phosphoribosyltransferase | 10135 | ENSG00000105835 |
| 55 | 202887\_s\_at | NA | DDIT4 | DNA-damage-inducible transcript 4 | 54541 | ENSG00000168209 |
| 56 | 202904\_s\_at | NA | LSM5 | LSM5 homolog, U6 small nuclear RNA associated (S. cerevisiae) | 23658 | ENSG00000106355 |
| 57 | 203552\_at | NA | MAP4K5 | mitogen-activated protein kinase kinase kinase kinase 5 | 11183 | ENSG00000012983 |
| 58 | 222765\_x\_at | NA | ESF1 | ESF1, nucleolar pre-rRNA processing protein, homolog (S. cerevisiae) | 51575 | ENSG00000089048 |
| 59 | 226965\_at | NA | FAM116A | family with sequence similarity 116, member A | 201627 | ENSG00000174839 |
| 60 | 208078\_s\_at | NA | SIK1 | salt-inducible kinase 1 | 150094 | ENSG00000142178 |
| 61 | 242109\_at | NA | SYTL3 | synaptotagmin-like 3 | 94120 | ENSG00000164674 |
| 62 | 1555962\_at | NA | B3GNT7 | UDP-GlcNAc:betaGal beta-1,3-N-acetylglucosaminyltransferase 7 | 93010 | ENSG00000156966 |
| 63 | 1555274\_a\_at | NA | EPT1 | ethanolaminephosphotransferase 1 (CDP-ethanolamine-specific) | 85465 | ENSG00000138018 |
| 64 | 226279\_at | NA | PRSS23 | protease, serine, 23 | 11098 | ENSG00000150687 |
| 65 | 238199\_x\_at | NA | COX3 | cytochrome c oxidase III | 4514 | NULL |
| 66 | 225189\_s\_at | NA | RAPH1 | Ras association (RalGDS/AF-6) and pleckstrin homology domains 1 | 65059 | ENSG00000173166 |
| 67 | 231863\_at | NA | ING3 | inhibitor of growth family, member 3 | 54556 | ENSG00000071243 |
| 68 | 1555878\_at | NA | RPS24 | ribosomal protein S24 | 6229 | ENSG00000138326 |
| 69 | 223266\_at | NA | STRADB | STE20-related kinase adaptor beta | 55437 | ENSG00000082146 |
| 70 | 209686\_at | NA | S100B | S100 calcium binding protein B | 6285 | ENSG00000160307 |
| 71 | 211560\_s\_at | NA | ALAS2 | aminolevulinate, delta-, synthase 2 | 212 | ENSG00000158578 |
| 72 | 205063\_at | NA | GEMIN2 | gem (nuclear organelle) associated protein 2 | 8487 | ENSG00000092208 |
| 73 | 203634\_s\_at | NA | CPT1A | carnitine palmitoyltransferase 1A (liver) | 1374 | ENSG00000110090 |
| 74 | 204995\_at | NA | CDK5R1 | cyclin-dependent kinase 5, regulatory subunit 1 (p35) | 8851 | ENSG00000176749 |
| 75 | 228063\_s\_at | NA | NAP1L5 | nucleosome assembly protein 1-like 5 | 266812 | ENSG00000177432 |
| 76 | 228062\_at | NA | NAP1L5 | nucleosome assembly protein 1-like 5 | 266812 | ENSG00000177432 |
| 77 | 218411\_s\_at | NA | MBIP | MAP3K12 binding inhibitory protein 1 | 51562 | ENSG00000151332 |
| 78 | 236487\_at | NA | SCLT1 | sodium channel and clathrin linker 1 | 132320 | ENSG00000151466 |
| 79 | 203574\_at | NA | NFIL3 | nuclear factor, interleukin 3 regulated | 4783 | ENSG00000165030 |
| 80 | 202232\_s\_at | NA | EIF3M | eukaryotic translation initiation factor 3, subunit M | 10480 | ENSG00000149100 |
| 81 | 238614\_x\_at | NA | ZNF430 | zinc finger protein 430 | 80264 | ENSG00000118620 |
| 82 | 230559\_x\_at | NA | FGD4 | FYVE, RhoGEF and PH domain containing 4 | 121512 | ENSG00000139132 |
| 83 | 216248\_s\_at | NA | NR4A2 | nuclear receptor subfamily 4, group A, member 2 | 4929 | ENSG00000153234 |
| 84 | 204621\_s\_at | NA | NR4A2 | nuclear receptor subfamily 4, group A, member 2 | 4929 | ENSG00000153234 |
| 85 | 204622\_x\_at | NA | NR4A2 | nuclear receptor subfamily 4, group A, member 2 | 4929 | ENSG00000153234 |
| 86 | 222309\_at | NA | C6orf62 | chromosome 6 open reading frame 62 | 81688 | ENSG00000112308 |
| 87 | 232044\_at | NA | RBBP6 | retinoblastoma binding protein 6 | 5930 | ENSG00000122257 |
| 88 | 200608\_s\_at | NA | RAD21 | RAD21 homolog (S. pombe) | 5885 | ENSG00000164754 |
| 89 | 202221\_s\_at | NA | EP300 | E1A binding protein p300 | 2033 | ENSG00000100393 |
| 90 | 206919\_at | NA | ELK4 | ELK4, ETS-domain protein (SRF accessory protein 1) | 2005 | ENSG00000158711 |
| 91 | 201304\_at | NA | NDUFA5 | NADH dehydrogenase (ubiquinone) 1 alpha subcomplex, 5, 13kDa | 4698 | ENSG00000128609 |
| 92 | 225760\_at | NA | MYSM1 | Myb-like, SWIRM and MPN domains 1 | 114803 | ENSG00000162601 |
| 93 | 222310\_at | NA | SCAF4 | SR-related CTD-associated factor 4 | 57466 | ENSG00000156304 |
| 94 | 213725\_x\_at | NA | XYLT1 | xylosyltransferase I | 64131 | ENSG00000103489 |
| 95 | 224453\_s\_at | NA | ETNK1 | ethanolamine kinase 1 | 55500 | ENSG00000139163 |
| 96 | 225290\_at | NA | ETNK1 | ethanolamine kinase 1 | 55500 | ENSG00000139163 |
| 97 | 202979\_s\_at | NA | CREBZF | CREB/ATF bZIP transcription factor | 58487 | ENSG00000137504 |
| 98 | 204187\_at | NA | GMPR | guanosine monophosphate reductase | 2766 | ENSG00000137198 |
| 99 | 233019\_at | NA | CNOT7 | CCR4-NOT transcription complex, subunit 7 | 29883 | ENSG00000198791 |
| 100 | 213225\_at | NA | PPM1B | protein phosphatase, Mg2+/Mn2+ dependent, 1B | 5495 | ENSG00000138032 |
| 101 | 200602\_at | NA | APP | amyloid beta (A4) precursor protein | 351 | ENSG00000142192 |
| 102 | 225580\_at | NA | MRPL50 | mitochondrial ribosomal protein L50 | 54534 | ENSG00000136897 |
| 103 | 225916\_at | NA | ZNF131 | zinc finger protein 131 | 7690 | ENSG00000172262 |
| 104 | 214741\_at | NA | ZNF131 | zinc finger protein 131 | 7690 | ENSG00000172262 |
| 105 | 201177\_s\_at | NA | UBA2 | ubiquitin-like modifier activating enzyme 2 | 10054 | ENSG00000126261 |
| 106 | 206834\_at | NA | HBD | hemoglobin, delta | 3045 | ENSG00000223609 |
| 107 | 227375\_at | NA | ANKRD13C | ankyrin repeat domain 13C | 81573 | ENSG00000118454 |
| 108 | 202422\_s\_at | NA | ACSL4 | acyl-CoA synthetase long-chain family member 4 | 2182 | ENSG00000068366 |
| 109 | 214590\_s\_at | NA | UBE2D1 | ubiquitin-conjugating enzyme E2D 1 | 7321 | ENSG00000072401 |
| 110 | 221768\_at | NA | SFPQ | splicing factor proline/glutamine-rich | 6421 | ENSG00000116560 |
| 111 | 226931\_at | NA | TMTC1 | transmembrane and tetratricopeptide repeat containing 1 | 83857 | ENSG00000133687 |
| 112 | 211698\_at | NA | EID1 | EP300 interacting inhibitor of differentiation 1 | 23741 | ENSG00000255302 |
| 113 | 202464\_s\_at | NA | PFKFB3 | 6-phosphofructo-2-kinase/fructose-2,6-biphosphatase 3 | 5209 | ENSG00000170525 |
| 114 | 240413\_at | NA | PYHIN1 | pyrin and HIN domain family, member 1 | 149628 | ENSG00000163564 |
| 115 | 236539\_at | NA | PTPN22 | protein tyrosine phosphatase, non-receptor type 22 (lymphoid) | 26191 | ENSG00000134242 |
| 116 | 226472\_at | NA | PPIL4 | peptidylprolyl isomerase (cyclophilin)-like 4 | 85313 | ENSG00000131013 |
| 117 | 227697\_at | NA | SOCS3 | suppressor of cytokine signaling 3 | 9021 | ENSG00000184557 |
| 118 | 211546\_x\_at | NA | SNCA | synuclein, alpha (non A4 component of amyloid precursor) | 6622 | ENSG00000145335 |
| 119 | 207827\_x\_at | NA | SNCA | synuclein, alpha (non A4 component of amyloid precursor) | 6622 | ENSG00000145335 |
| 120 | 236081\_at | NA | SNCA | synuclein, alpha (non A4 component of amyloid precursor) | 6622 | ENSG00000145335 |
| 121 | 204467\_s\_at | NA | SNCA | synuclein, alpha (non A4 component of amyloid precursor) | 6622 | ENSG00000145335 |
| 122 | 204466\_s\_at | NA | SNCA | synuclein, alpha (non A4 component of amyloid precursor) | 6622 | ENSG00000145335 |
| 123 | 213998\_s\_at | NA | DDX17 | DEAD (Asp-Glu-Ala-Asp) box helicase 17 | 10521 | ENSG00000100201 |
| 124 | 208719\_s\_at | NA | DDX17 | DEAD (Asp-Glu-Ala-Asp) box helicase 17 | 10521 | ENSG00000100201 |
| 125 | 208151\_x\_at | NA | DDX17 | DEAD (Asp-Glu-Ala-Asp) box helicase 17 | 10521 | ENSG00000100201 |
| 126 | 1555960\_at | NA | HINT1 | histidine triad nucleotide binding protein 1 | 3094 | ENSG00000169567 |
| 127 | 224009\_x\_at | NA | DHRS9 | dehydrogenase/reductase (SDR family) member 9 | 10170 | ENSG00000073737 |
| 128 | 223952\_x\_at | NA | DHRS9 | dehydrogenase/reductase (SDR family) member 9 | 10170 | ENSG00000073737 |
| 129 | 213524\_s\_at | NA | G0S2 | G0/G1switch 2 | 50486 | ENSG00000123689 |
| 130 | 201407\_s\_at | NA | PPP1CB | protein phosphatase 1, catalytic subunit, beta isozyme | 5500 | ENSG00000213639 |
| 131 | 223649\_s\_at | NA | SLC25A39 | solute carrier family 25, member 39 | 51629 | ENSG00000013306 |
| 132 | 203603\_s\_at | NA | ZEB2 | zinc finger E-box binding homeobox 2 | 9839 | ENSG00000169554 |
| 133 | 204794\_at | NA | DUSP2 | dual specificity phosphatase 2 | 1844 | ENSG00000158050 |
| 134 | 236140\_at | NA | GCLM | glutamate-cysteine ligase, modifier subunit | 2730 | ENSG00000023909 |
| 135 | 235412\_at | NA | ARHGEF7 | Rho guanine nucleotide exchange factor (GEF) 7 | 8874 | ENSG00000102606 |
| 136 | 206108\_s\_at | NA | SRSF6 | serine/arginine-rich splicing factor 6 | 6431 | ENSG00000124193 |
| 137 | 226366\_at | NA | SHPRH | SNF2 histone linker PHD RING helicase, E3 ubiquitin protein ligase | 257218 | ENSG00000146414 |
| 138 | 212989\_at | NA | SGMS1 | sphingomyelin synthase 1 | 259230 | ENSG00000198964 |
| 139 | 213786\_at | NA | TAX1BP1 | Tax1 (human T-cell leukemia virus type I) binding protein 1 | 8887 | ENSG00000106052 |
| 140 | 1554309\_at | NA | EIF4G3 | eukaryotic translation initiation factor 4 gamma, 3 | 8672 | ENSG00000075151 |
| 141 | 220018\_at | NA | CBLL1 | Cbl proto-oncogene, E3 ubiquitin protein ligase-like 1 | 79872 | ENSG00000105879 |
| 142 | 217851\_s\_at | NA | SLMO2 | slowmo homolog 2 (Drosophila) | 51012 | ENSG00000101166 |
| 143 | 210746\_s\_at | NA | EPB42 | erythrocyte membrane protein band 4.2 | 2038 | ENSG00000166947 |
| 144 | 230380\_at | NA | THAP2 | THAP domain containing, apoptosis associated protein 2 | 83591 | ENSG00000173451 |
| 145 | 216834\_at | NA | RGS1 | regulator of G-protein signaling 1 | 5996 | ENSG00000090104 |
| 146 | 202988\_s\_at | NA | RGS1 | regulator of G-protein signaling 1 | 5996 | ENSG00000090104 |
| 147 | 202843\_at | NA | DNAJB9 | DnaJ (Hsp40) homolog, subfamily B, member 9 | 4189 | ENSG00000128590 |
| 148 | 203358\_s\_at | NA | EZH2 | enhancer of zeste homolog 2 (Drosophila) | 2146 | ENSG00000106462 |
| 149 | 208762\_at | NA | SUMO1 | SMT3 suppressor of mif two 3 homolog 1 (S. cerevisiae) | 7341 | ENSG00000116030 |
| 150 | 1558233\_s\_at | NA | ATF1 | activating transcription factor 1 | 466 | ENSG00000123268 |
| 151 | 216929\_x\_at | NA | ABO | ABO blood group (transferase A, alpha 1-3-N-acetylgalactosaminyltransferase; transferase B, alpha 1-3-galactosyltransferase) | 28 | NULL |
| 152 | 204176\_at | NA | KLHL20 | kelch-like 20 (Drosophila) | 27252 | ENSG00000076321 |
| 153 | 1569136\_at | NA | MGAT4A | mannosyl (alpha-1,3-)-glycoprotein beta-1,4-N-acetylglucosaminyltransferase, isozyme A | 11320 | ENSG00000071073 |
| 154 | 209451\_at | NA | TANK | TRAF family member-associated NFKB activator | 10010 | ENSG00000136560 |
| 155 | 222858\_s\_at | NA | DAPP1 | dual adaptor of phosphotyrosine and 3-phosphoinositides | 27071 | ENSG00000070190 |
| 156 | 203049\_s\_at | NA | TTC37 | tetratricopeptide repeat domain 37 | 9652 | ENSG00000198677 |
| 157 | 212637\_s\_at | NA | WWP1 | WW domain containing E3 ubiquitin protein ligase 1 | 11059 | ENSG00000123124 |
| 158 | 203543\_s\_at | NA | KLF9 | Kruppel-like factor 9 | 687 | ENSG00000119138 |
| 159 | 212579\_at | NA | SMCHD1 | structural maintenance of chromosomes flexible hinge domain containing 1 | 23347 | ENSG00000101596 |
| 160 | 211760\_s\_at | NA | VAMP4 | vesicle-associated membrane protein 4 | 8674 | ENSG00000117533 |
| 161 | 202644\_s\_at | NA | TNFAIP3 | tumor necrosis factor, alpha-induced protein 3 | 7128 | ENSG00000118503 |
| 162 | 202203\_s\_at | NA | AMFR | autocrine motility factor receptor, E3 ubiquitin protein ligase | 267 | ENSG00000159461 |
| 163 | 207794\_at | NA | CCR2 | chemokine (C-C motif) receptor 2 | 729230 | ENSG00000121807 |
| 164 | 242960\_at | NA | EPC2 | enhancer of polycomb homolog 2 (Drosophila) | 26122 | ENSG00000135999 |
| 165 | 230875\_s\_at | NA | ATP11A | ATPase, class VI, type 11A | 23250 | ENSG00000068650 |
| 166 | 202933\_s\_at | NA | YES1 | v-yes-1 Yamaguchi sarcoma viral oncogene homolog 1 | 7525 | ENSG00000176105 |
| 167 | 222317\_at | NA | PDE3B | phosphodiesterase 3B, cGMP-inhibited | 5140 | ENSG00000152270 |
| 168 | 1558956\_s\_at | NA | IFT80 | intraflagellar transport 80 homolog (Chlamydomonas) | 57560 | ENSG00000248710 ENSG00000068885 |
| 169 | 201091\_s\_at | NA | CBX3 | chromobox homolog 3 | 11335 | ENSG00000122565 |
| 170 | 1557257\_at | NA | BCL10 | B-cell CLL/lymphoma 10 | 8915 | ENSG00000142867 |
| 171 | 1559975\_at | NA | BTG1 | B-cell translocation gene 1, anti-proliferative | 694 | ENSG00000133639 |
| 172 | 1555476\_at | NA | IREB2 | iron-responsive element binding protein 2 | 3658 | ENSG00000136381 |
| 173 | 203884\_s\_at | NA | RAB11FIP2 | RAB11 family interacting protein 2 (class I) | 22841 | ENSG00000107560 |
| 174 | 227012\_at | NA | SLC25A40 | solute carrier family 25, member 40 | 55972 | ENSG00000075303 |
| 175 | 239143\_x\_at | NA | RNF138 | ring finger protein 138, E3 ubiquitin protein ligase | 51444 | ENSG00000134758 |
| 176 | 214012\_at | NA | ERAP1 | endoplasmic reticulum aminopeptidase 1 | 51752 | ENSG00000164307 |
| 177 | 219304\_s\_at | NA | PDGFD | platelet derived growth factor D | 80310 | ENSG00000170962 |
| 178 | 213372\_at | NA | PAQR3 | progestin and adipoQ receptor family member III | 152559 | ENSG00000163291 |
| 179 | 212633\_at | NA | UFL1 | UFM1-specific ligase 1 | 23376 | ENSG00000014123 |
| 180 | 207300\_s\_at | NA | F7 | coagulation factor VII (serum prothrombin conversion accelerator) | 2155 | ENSG00000057593 |

  
  

| **Database:cellular component      &nbspName:intracellular part      &nbspID:GO:0044424** | | | | | | |
| --- | --- | --- | --- | --- | --- | --- |
| C=11551; O=160; E=138.13; R=1.16; rawP=5.73e-05; adjP=0.0050 | | | | | | |
| Index | UserID | Value | Gene Symbol | Gene Name | EntrezGene | Ensembl |
| 1 | 228106\_at | NA | DCAF16 | DDB1 and CUL4 associated factor 16 | 54876 | ENSG00000163257 |
| 2 | 214658\_at | NA | TMED7 | transmembrane emp24 protein transport domain containing 7 | 51014 | ENSG00000134970 |
| 3 | 219312\_s\_at | NA | ZBTB10 | zinc finger and BTB domain containing 10 | 65986 | ENSG00000205189 |
| 4 | 227449\_at | NA | EPHA4 | EPH receptor A4 | 2043 | ENSG00000116106 |
| 5 | 201016\_at | NA | EIF1AX | eukaryotic translation initiation factor 1A, X-linked | 1964 | ENSG00000173674 |
| 6 | 235300\_x\_at | NA | RCHY1 | ring finger and CHY zinc finger domain containing 1, E3 ubiquitin protein ligase | 25898 | ENSG00000163743 |
| 7 | 226181\_at | NA | TUBE1 | tubulin, epsilon 1 | 51175 | ENSG00000074935 |
| 8 | 207078\_at | NA | MED6 | mediator complex subunit 6 | 10001 | ENSG00000133997 |
| 9 | 218701\_at | NA | LACTB2 | lactamase, beta 2 | 51110 | ENSG00000147592 |
| 10 | 202660\_at | NA | ITPR2 | inositol 1,4,5-trisphosphate receptor, type 2 | 3709 | ENSG00000123104 |
| 11 | 222528\_s\_at | NA | SLC25A37 | solute carrier family 25 (mitochondrial iron transporter), member 37 | 51312 | ENSG00000147454 |
| 12 | 226928\_x\_at | NA | SLC25A37 | solute carrier family 25 (mitochondrial iron transporter), member 37 | 51312 | ENSG00000147454 |
| 13 | 228527\_s\_at | NA | SLC25A37 | solute carrier family 25 (mitochondrial iron transporter), member 37 | 51312 | ENSG00000147454 |
| 14 | 226179\_at | NA | SLC25A37 | solute carrier family 25 (mitochondrial iron transporter), member 37 | 51312 | ENSG00000147454 |
| 15 | 1558692\_at | NA | C1orf85 | chromosome 1 open reading frame 85 | 112770 | ENSG00000198715 |
| 16 | 203203\_s\_at | NA | KRR1 | KRR1, small subunit (SSU) processome component, homolog (yeast) | 11103 | ENSG00000111615 |
| 17 | 243916\_x\_at | NA | UBLCP1 | ubiquitin-like domain containing CTD phosphatase 1 | 134510 | ENSG00000164332 |
| 18 | 202314\_at | NA | CYP51A1 | cytochrome P450, family 51, subfamily A, polypeptide 1 | 1595 | ENSG00000001630 |
| 19 | 235683\_at | NA | SESN3 | sestrin 3 | 143686 | ENSG00000149212 |
| 20 | 226680\_at | NA | IKZF5 | IKAROS family zinc finger 5 (Pegasus) | 64376 | ENSG00000095574 |
| 21 | 238020\_at | NA | PSMC2 | proteasome (prosome, macropain) 26S subunit, ATPase, 2 | 5701 | ENSG00000161057 |
| 22 | 222243\_s\_at | NA | TOB2 | transducer of ERBB2, 2 | 10766 | ENSG00000183864 |
| 23 | 230379\_x\_at | NA | C2orf56 | chromosome 2 open reading frame 56 | 55471 | ENSG00000003509 |
| 24 | 213459\_at | NA | RPL37A | ribosomal protein L37a | 6168 | ENSG00000197756 |
| 25 | 205191\_at | NA | RP2 | retinitis pigmentosa 2 (X-linked recessive) | 6102 | ENSG00000102218 |
| 26 | 201437\_s\_at | NA | EIF4E | eukaryotic translation initiation factor 4E | 1977 | ENSG00000151247 |
| 27 | 232068\_s\_at | NA | TLR4 | toll-like receptor 4 | 7099 | ENSG00000136869 |
| 28 | 218871\_x\_at | NA | CSGALNACT2 | chondroitin sulfate N-acetylgalactosaminyltransferase 2 | 55454 | ENSG00000169826 |
| 29 | 204299\_at | NA | SRSF10 | serine/arginine-rich splicing factor 10 | 10772 | ENSG00000188529 |
| 30 | 229943\_at | NA | TRIM13 | tripartite motif containing 13 | 10206 | ENSG00000204977 |
| 31 | 227900\_at | NA | CBLB | Cbl proto-oncogene, E3 ubiquitin protein ligase B | 868 | ENSG00000114423 |
| 32 | 222848\_at | NA | CENPK | centromere protein K | 64105 | ENSG00000123219 |
| 33 | 205283\_at | NA | FKTN | fukutin | 2218 | ENSG00000106692 |
| 34 | 223200\_s\_at | NA | LSG1 | large subunit GTPase 1 homolog (S. cerevisiae) | 55341 | ENSG00000041802 |
| 35 | 218401\_s\_at | NA | ZNF281 | zinc finger protein 281 | 23528 | ENSG00000162702 |
| 36 | 228785\_at | NA | ZNF281 | zinc finger protein 281 | 23528 | ENSG00000162702 |
| 37 | 213404\_s\_at | NA | RHEB | Ras homolog enriched in brain | 6009 | ENSG00000106615 |
| 38 | 213418\_at | NA | HSPA6 | heat shock 70kDa protein 6 (HSP70B') | 3310 | ENSG00000173110 |
| 39 | 205900\_at | NA | KRT1 | keratin 1 | 3848 | ENSG00000167768 |
| 40 | 202861\_at | NA | PER1 | period homolog 1 (Drosophila) | 5187 | ENSG00000179094 |
| 41 | 203310\_at | NA | STXBP3 | syntaxin binding protein 3 | 6814 | ENSG00000116266 |
| 42 | 201088\_at | NA | KPNA2 | karyopherin alpha 2 (RAG cohort 1, importin alpha 1) | 3838 | ENSG00000182481 |
| 43 | 1553132\_a\_at | NA | TC2N | tandem C2 domains, nuclear | 123036 | ENSG00000165929 |
| 44 | 36711\_at | NA | MAFF | v-maf musculoaponeurotic fibrosarcoma oncogene homolog F (avian) | 23764 | ENSG00000185022 |
| 45 | 229431\_at | NA | RFXAP | regulatory factor X-associated protein | 5994 | ENSG00000133111 |
| 46 | 207545\_s\_at | NA | NUMB | numb homolog (Drosophila) | 8650 | ENSG00000133961 |
| 47 | 212847\_at | NA | FUBP1 | far upstream element (FUSE) binding protein 1 | 8880 | ENSG00000162613 |
| 48 | 240336\_at | NA | HBM | hemoglobin, mu | 3042 | ENSG00000206177 |
| 49 | 227391\_x\_at | NA | LRRFIP1 | leucine rich repeat (in FLII) interacting protein 1 | 9208 | ENSG00000124831 |
| 50 | 222876\_s\_at | NA | ADAP2 | ArfGAP with dual PH domains 2 | 55803 | ENSG00000184060 |
| 51 | 202375\_at | NA | SEC24D | SEC24 family, member D (S. cerevisiae) | 9871 | ENSG00000150961 |
| 52 | 214917\_at | NA | PRKAA1 | protein kinase, AMP-activated, alpha 1 catalytic subunit | 5562 | ENSG00000132356 |
| 53 | 204614\_at | NA | SERPINB2 | serpin peptidase inhibitor, clade B (ovalbumin), member 2 | 5055 | ENSG00000197632 |
| 54 | 217739\_s\_at | NA | NAMPT | nicotinamide phosphoribosyltransferase | 10135 | ENSG00000105835 |
| 55 | 202887\_s\_at | NA | DDIT4 | DNA-damage-inducible transcript 4 | 54541 | ENSG00000168209 |
| 56 | 202904\_s\_at | NA | LSM5 | LSM5 homolog, U6 small nuclear RNA associated (S. cerevisiae) | 23658 | ENSG00000106355 |
| 57 | 203552\_at | NA | MAP4K5 | mitogen-activated protein kinase kinase kinase kinase 5 | 11183 | ENSG00000012983 |
| 58 | 222765\_x\_at | NA | ESF1 | ESF1, nucleolar pre-rRNA processing protein, homolog (S. cerevisiae) | 51575 | ENSG00000089048 |
| 59 | 226965\_at | NA | FAM116A | family with sequence similarity 116, member A | 201627 | ENSG00000174839 |
| 60 | 208078\_s\_at | NA | SIK1 | salt-inducible kinase 1 | 150094 | ENSG00000142178 |
| 61 | 1555962\_at | NA | B3GNT7 | UDP-GlcNAc:betaGal beta-1,3-N-acetylglucosaminyltransferase 7 | 93010 | ENSG00000156966 |
| 62 | 1555274\_a\_at | NA | EPT1 | ethanolaminephosphotransferase 1 (CDP-ethanolamine-specific) | 85465 | ENSG00000138018 |
| 63 | 226279\_at | NA | PRSS23 | protease, serine, 23 | 11098 | ENSG00000150687 |
| 64 | 238199\_x\_at | NA | COX3 | cytochrome c oxidase III | 4514 | NULL |
| 65 | 225189\_s\_at | NA | RAPH1 | Ras association (RalGDS/AF-6) and pleckstrin homology domains 1 | 65059 | ENSG00000173166 |
| 66 | 1555878\_at | NA | RPS24 | ribosomal protein S24 | 6229 | ENSG00000138326 |
| 67 | 223266\_at | NA | STRADB | STE20-related kinase adaptor beta | 55437 | ENSG00000082146 |
| 68 | 231863\_at | NA | ING3 | inhibitor of growth family, member 3 | 54556 | ENSG00000071243 |
| 69 | 209686\_at | NA | S100B | S100 calcium binding protein B | 6285 | ENSG00000160307 |
| 70 | 211560\_s\_at | NA | ALAS2 | aminolevulinate, delta-, synthase 2 | 212 | ENSG00000158578 |
| 71 | 205063\_at | NA | GEMIN2 | gem (nuclear organelle) associated protein 2 | 8487 | ENSG00000092208 |
| 72 | 203634\_s\_at | NA | CPT1A | carnitine palmitoyltransferase 1A (liver) | 1374 | ENSG00000110090 |
| 73 | 204995\_at | NA | CDK5R1 | cyclin-dependent kinase 5, regulatory subunit 1 (p35) | 8851 | ENSG00000176749 |
| 74 | 228063\_s\_at | NA | NAP1L5 | nucleosome assembly protein 1-like 5 | 266812 | ENSG00000177432 |
| 75 | 228062\_at | NA | NAP1L5 | nucleosome assembly protein 1-like 5 | 266812 | ENSG00000177432 |
| 76 | 218411\_s\_at | NA | MBIP | MAP3K12 binding inhibitory protein 1 | 51562 | ENSG00000151332 |
| 77 | 236487\_at | NA | SCLT1 | sodium channel and clathrin linker 1 | 132320 | ENSG00000151466 |
| 78 | 203574\_at | NA | NFIL3 | nuclear factor, interleukin 3 regulated | 4783 | ENSG00000165030 |
| 79 | 202232\_s\_at | NA | EIF3M | eukaryotic translation initiation factor 3, subunit M | 10480 | ENSG00000149100 |
| 80 | 238614\_x\_at | NA | ZNF430 | zinc finger protein 430 | 80264 | ENSG00000118620 |
| 81 | 230559\_x\_at | NA | FGD4 | FYVE, RhoGEF and PH domain containing 4 | 121512 | ENSG00000139132 |
| 82 | 216248\_s\_at | NA | NR4A2 | nuclear receptor subfamily 4, group A, member 2 | 4929 | ENSG00000153234 |
| 83 | 204621\_s\_at | NA | NR4A2 | nuclear receptor subfamily 4, group A, member 2 | 4929 | ENSG00000153234 |
| 84 | 204622\_x\_at | NA | NR4A2 | nuclear receptor subfamily 4, group A, member 2 | 4929 | ENSG00000153234 |
| 85 | 232044\_at | NA | RBBP6 | retinoblastoma binding protein 6 | 5930 | ENSG00000122257 |
| 86 | 200608\_s\_at | NA | RAD21 | RAD21 homolog (S. pombe) | 5885 | ENSG00000164754 |
| 87 | 202221\_s\_at | NA | EP300 | E1A binding protein p300 | 2033 | ENSG00000100393 |
| 88 | 206919\_at | NA | ELK4 | ELK4, ETS-domain protein (SRF accessory protein 1) | 2005 | ENSG00000158711 |
| 89 | 201304\_at | NA | NDUFA5 | NADH dehydrogenase (ubiquinone) 1 alpha subcomplex, 5, 13kDa | 4698 | ENSG00000128609 |
| 90 | 225760\_at | NA | MYSM1 | Myb-like, SWIRM and MPN domains 1 | 114803 | ENSG00000162601 |
| 91 | 222310\_at | NA | SCAF4 | SR-related CTD-associated factor 4 | 57466 | ENSG00000156304 |
| 92 | 213725\_x\_at | NA | XYLT1 | xylosyltransferase I | 64131 | ENSG00000103489 |
| 93 | 224453\_s\_at | NA | ETNK1 | ethanolamine kinase 1 | 55500 | ENSG00000139163 |
| 94 | 225290\_at | NA | ETNK1 | ethanolamine kinase 1 | 55500 | ENSG00000139163 |
| 95 | 202979\_s\_at | NA | CREBZF | CREB/ATF bZIP transcription factor | 58487 | ENSG00000137504 |
| 96 | 204187\_at | NA | GMPR | guanosine monophosphate reductase | 2766 | ENSG00000137198 |
| 97 | 233019\_at | NA | CNOT7 | CCR4-NOT transcription complex, subunit 7 | 29883 | ENSG00000198791 |
| 98 | 213225\_at | NA | PPM1B | protein phosphatase, Mg2+/Mn2+ dependent, 1B | 5495 | ENSG00000138032 |
| 99 | 200602\_at | NA | APP | amyloid beta (A4) precursor protein | 351 | ENSG00000142192 |
| 100 | 225580\_at | NA | MRPL50 | mitochondrial ribosomal protein L50 | 54534 | ENSG00000136897 |
| 101 | 225916\_at | NA | ZNF131 | zinc finger protein 131 | 7690 | ENSG00000172262 |
| 102 | 214741\_at | NA | ZNF131 | zinc finger protein 131 | 7690 | ENSG00000172262 |
| 103 | 201177\_s\_at | NA | UBA2 | ubiquitin-like modifier activating enzyme 2 | 10054 | ENSG00000126261 |
| 104 | 206834\_at | NA | HBD | hemoglobin, delta | 3045 | ENSG00000223609 |
| 105 | 227375\_at | NA | ANKRD13C | ankyrin repeat domain 13C | 81573 | ENSG00000118454 |
| 106 | 202422\_s\_at | NA | ACSL4 | acyl-CoA synthetase long-chain family member 4 | 2182 | ENSG00000068366 |
| 107 | 214590\_s\_at | NA | UBE2D1 | ubiquitin-conjugating enzyme E2D 1 | 7321 | ENSG00000072401 |
| 108 | 221768\_at | NA | SFPQ | splicing factor proline/glutamine-rich | 6421 | ENSG00000116560 |
| 109 | 226931\_at | NA | TMTC1 | transmembrane and tetratricopeptide repeat containing 1 | 83857 | ENSG00000133687 |
| 110 | 211698\_at | NA | EID1 | EP300 interacting inhibitor of differentiation 1 | 23741 | ENSG00000255302 |
| 111 | 202464\_s\_at | NA | PFKFB3 | 6-phosphofructo-2-kinase/fructose-2,6-biphosphatase 3 | 5209 | ENSG00000170525 |
| 112 | 240413\_at | NA | PYHIN1 | pyrin and HIN domain family, member 1 | 149628 | ENSG00000163564 |
| 113 | 236539\_at | NA | PTPN22 | protein tyrosine phosphatase, non-receptor type 22 (lymphoid) | 26191 | ENSG00000134242 |
| 114 | 226472\_at | NA | PPIL4 | peptidylprolyl isomerase (cyclophilin)-like 4 | 85313 | ENSG00000131013 |
| 115 | 227697\_at | NA | SOCS3 | suppressor of cytokine signaling 3 | 9021 | ENSG00000184557 |
| 116 | 211546\_x\_at | NA | SNCA | synuclein, alpha (non A4 component of amyloid precursor) | 6622 | ENSG00000145335 |
| 117 | 207827\_x\_at | NA | SNCA | synuclein, alpha (non A4 component of amyloid precursor) | 6622 | ENSG00000145335 |
| 118 | 236081\_at | NA | SNCA | synuclein, alpha (non A4 component of amyloid precursor) | 6622 | ENSG00000145335 |
| 119 | 204467\_s\_at | NA | SNCA | synuclein, alpha (non A4 component of amyloid precursor) | 6622 | ENSG00000145335 |
| 120 | 204466\_s\_at | NA | SNCA | synuclein, alpha (non A4 component of amyloid precursor) | 6622 | ENSG00000145335 |
| 121 | 213998\_s\_at | NA | DDX17 | DEAD (Asp-Glu-Ala-Asp) box helicase 17 | 10521 | ENSG00000100201 |
| 122 | 208719\_s\_at | NA | DDX17 | DEAD (Asp-Glu-Ala-Asp) box helicase 17 | 10521 | ENSG00000100201 |
| 123 | 208151\_x\_at | NA | DDX17 | DEAD (Asp-Glu-Ala-Asp) box helicase 17 | 10521 | ENSG00000100201 |
| 124 | 1555960\_at | NA | HINT1 | histidine triad nucleotide binding protein 1 | 3094 | ENSG00000169567 |
| 125 | 224009\_x\_at | NA | DHRS9 | dehydrogenase/reductase (SDR family) member 9 | 10170 | ENSG00000073737 |
| 126 | 223952\_x\_at | NA | DHRS9 | dehydrogenase/reductase (SDR family) member 9 | 10170 | ENSG00000073737 |
| 127 | 213524\_s\_at | NA | G0S2 | G0/G1switch 2 | 50486 | ENSG00000123689 |
| 128 | 201407\_s\_at | NA | PPP1CB | protein phosphatase 1, catalytic subunit, beta isozyme | 5500 | ENSG00000213639 |
| 129 | 223649\_s\_at | NA | SLC25A39 | solute carrier family 25, member 39 | 51629 | ENSG00000013306 |
| 130 | 203603\_s\_at | NA | ZEB2 | zinc finger E-box binding homeobox 2 | 9839 | ENSG00000169554 |
| 131 | 204794\_at | NA | DUSP2 | dual specificity phosphatase 2 | 1844 | ENSG00000158050 |
| 132 | 236140\_at | NA | GCLM | glutamate-cysteine ligase, modifier subunit | 2730 | ENSG00000023909 |
| 133 | 235412\_at | NA | ARHGEF7 | Rho guanine nucleotide exchange factor (GEF) 7 | 8874 | ENSG00000102606 |
| 134 | 206108\_s\_at | NA | SRSF6 | serine/arginine-rich splicing factor 6 | 6431 | ENSG00000124193 |
| 135 | 226366\_at | NA | SHPRH | SNF2 histone linker PHD RING helicase, E3 ubiquitin protein ligase | 257218 | ENSG00000146414 |
| 136 | 212989\_at | NA | SGMS1 | sphingomyelin synthase 1 | 259230 | ENSG00000198964 |
| 137 | 213786\_at | NA | TAX1BP1 | Tax1 (human T-cell leukemia virus type I) binding protein 1 | 8887 | ENSG00000106052 |
| 138 | 1554309\_at | NA | EIF4G3 | eukaryotic translation initiation factor 4 gamma, 3 | 8672 | ENSG00000075151 |
| 139 | 220018\_at | NA | CBLL1 | Cbl proto-oncogene, E3 ubiquitin protein ligase-like 1 | 79872 | ENSG00000105879 |
| 140 | 217851\_s\_at | NA | SLMO2 | slowmo homolog 2 (Drosophila) | 51012 | ENSG00000101166 |
| 141 | 210746\_s\_at | NA | EPB42 | erythrocyte membrane protein band 4.2 | 2038 | ENSG00000166947 |
| 142 | 230380\_at | NA | THAP2 | THAP domain containing, apoptosis associated protein 2 | 83591 | ENSG00000173451 |
| 143 | 202843\_at | NA | DNAJB9 | DnaJ (Hsp40) homolog, subfamily B, member 9 | 4189 | ENSG00000128590 |
| 144 | 216834\_at | NA | RGS1 | regulator of G-protein signaling 1 | 5996 | ENSG00000090104 |
| 145 | 202988\_s\_at | NA | RGS1 | regulator of G-protein signaling 1 | 5996 | ENSG00000090104 |
| 146 | 203358\_s\_at | NA | EZH2 | enhancer of zeste homolog 2 (Drosophila) | 2146 | ENSG00000106462 |
| 147 | 208762\_at | NA | SUMO1 | SMT3 suppressor of mif two 3 homolog 1 (S. cerevisiae) | 7341 | ENSG00000116030 |
| 148 | 1558233\_s\_at | NA | ATF1 | activating transcription factor 1 | 466 | ENSG00000123268 |
| 149 | 216929\_x\_at | NA | ABO | ABO blood group (transferase A, alpha 1-3-N-acetylgalactosaminyltransferase; transferase B, alpha 1-3-galactosyltransferase) | 28 | NULL |
| 150 | 204176\_at | NA | KLHL20 | kelch-like 20 (Drosophila) | 27252 | ENSG00000076321 |
| 151 | 1569136\_at | NA | MGAT4A | mannosyl (alpha-1,3-)-glycoprotein beta-1,4-N-acetylglucosaminyltransferase, isozyme A | 11320 | ENSG00000071073 |
| 152 | 209451\_at | NA | TANK | TRAF family member-associated NFKB activator | 10010 | ENSG00000136560 |
| 153 | 222858\_s\_at | NA | DAPP1 | dual adaptor of phosphotyrosine and 3-phosphoinositides | 27071 | ENSG00000070190 |
| 154 | 203049\_s\_at | NA | TTC37 | tetratricopeptide repeat domain 37 | 9652 | ENSG00000198677 |
| 155 | 212637\_s\_at | NA | WWP1 | WW domain containing E3 ubiquitin protein ligase 1 | 11059 | ENSG00000123124 |
| 156 | 203543\_s\_at | NA | KLF9 | Kruppel-like factor 9 | 687 | ENSG00000119138 |
| 157 | 212579\_at | NA | SMCHD1 | structural maintenance of chromosomes flexible hinge domain containing 1 | 23347 | ENSG00000101596 |
| 158 | 211760\_s\_at | NA | VAMP4 | vesicle-associated membrane protein 4 | 8674 | ENSG00000117533 |
| 159 | 202644\_s\_at | NA | TNFAIP3 | tumor necrosis factor, alpha-induced protein 3 | 7128 | ENSG00000118503 |
| 160 | 202203\_s\_at | NA | AMFR | autocrine motility factor receptor, E3 ubiquitin protein ligase | 267 | ENSG00000159461 |
| 161 | 207794\_at | NA | CCR2 | chemokine (C-C motif) receptor 2 | 729230 | ENSG00000121807 |
| 162 | 242960\_at | NA | EPC2 | enhancer of polycomb homolog 2 (Drosophila) | 26122 | ENSG00000135999 |
| 163 | 230875\_s\_at | NA | ATP11A | ATPase, class VI, type 11A | 23250 | ENSG00000068650 |
| 164 | 202933\_s\_at | NA | YES1 | v-yes-1 Yamaguchi sarcoma viral oncogene homolog 1 | 7525 | ENSG00000176105 |
| 165 | 222317\_at | NA | PDE3B | phosphodiesterase 3B, cGMP-inhibited | 5140 | ENSG00000152270 |
| 166 | 1558956\_s\_at | NA | IFT80 | intraflagellar transport 80 homolog (Chlamydomonas) | 57560 | ENSG00000248710 ENSG00000068885 |
| 167 | 201091\_s\_at | NA | CBX3 | chromobox homolog 3 | 11335 | ENSG00000122565 |
| 168 | 1557257\_at | NA | BCL10 | B-cell CLL/lymphoma 10 | 8915 | ENSG00000142867 |
| 169 | 1559975\_at | NA | BTG1 | B-cell translocation gene 1, anti-proliferative | 694 | ENSG00000133639 |
| 170 | 1555476\_at | NA | IREB2 | iron-responsive element binding protein 2 | 3658 | ENSG00000136381 |
| 171 | 203884\_s\_at | NA | RAB11FIP2 | RAB11 family interacting protein 2 (class I) | 22841 | ENSG00000107560 |
| 172 | 227012\_at | NA | SLC25A40 | solute carrier family 25, member 40 | 55972 | ENSG00000075303 |
| 173 | 214012\_at | NA | ERAP1 | endoplasmic reticulum aminopeptidase 1 | 51752 | ENSG00000164307 |
| 174 | 219304\_s\_at | NA | PDGFD | platelet derived growth factor D | 80310 | ENSG00000170962 |
| 175 | 213372\_at | NA | PAQR3 | progestin and adipoQ receptor family member III | 152559 | ENSG00000163291 |
| 176 | 212633\_at | NA | UFL1 | UFM1-specific ligase 1 | 23376 | ENSG00000014123 |
| 177 | 207300\_s\_at | NA | F7 | coagulation factor VII (serum prothrombin conversion accelerator) | 2155 | ENSG00000057593 |

  
  

| **Database:cellular component      &nbspName:membrane-bounded organelle      &nbspID:GO:0043227** | | | | | | |
| --- | --- | --- | --- | --- | --- | --- |
| C=9128; O=130; E=109.15; R=1.19; rawP=0.0009; adjP=0.0175 | | | | | | |
| Index | UserID | Value | Gene Symbol | Gene Name | EntrezGene | Ensembl |
| 1 | 214658\_at | NA | TMED7 | transmembrane emp24 protein transport domain containing 7 | 51014 | ENSG00000134970 |
| 2 | 219312\_s\_at | NA | ZBTB10 | zinc finger and BTB domain containing 10 | 65986 | ENSG00000205189 |
| 3 | 227449\_at | NA | EPHA4 | EPH receptor A4 | 2043 | ENSG00000116106 |
| 4 | 235300\_x\_at | NA | RCHY1 | ring finger and CHY zinc finger domain containing 1, E3 ubiquitin protein ligase | 25898 | ENSG00000163743 |
| 5 | 207078\_at | NA | MED6 | mediator complex subunit 6 | 10001 | ENSG00000133997 |
| 6 | 202660\_at | NA | ITPR2 | inositol 1,4,5-trisphosphate receptor, type 2 | 3709 | ENSG00000123104 |
| 7 | 218701\_at | NA | LACTB2 | lactamase, beta 2 | 51110 | ENSG00000147592 |
| 8 | 222528\_s\_at | NA | SLC25A37 | solute carrier family 25 (mitochondrial iron transporter), member 37 | 51312 | ENSG00000147454 |
| 9 | 226928\_x\_at | NA | SLC25A37 | solute carrier family 25 (mitochondrial iron transporter), member 37 | 51312 | ENSG00000147454 |
| 10 | 228527\_s\_at | NA | SLC25A37 | solute carrier family 25 (mitochondrial iron transporter), member 37 | 51312 | ENSG00000147454 |
| 11 | 226179\_at | NA | SLC25A37 | solute carrier family 25 (mitochondrial iron transporter), member 37 | 51312 | ENSG00000147454 |
| 12 | 1558692\_at | NA | C1orf85 | chromosome 1 open reading frame 85 | 112770 | ENSG00000198715 |
| 13 | 203203\_s\_at | NA | KRR1 | KRR1, small subunit (SSU) processome component, homolog (yeast) | 11103 | ENSG00000111615 |
| 14 | 243916\_x\_at | NA | UBLCP1 | ubiquitin-like domain containing CTD phosphatase 1 | 134510 | ENSG00000164332 |
| 15 | 202314\_at | NA | CYP51A1 | cytochrome P450, family 51, subfamily A, polypeptide 1 | 1595 | ENSG00000001630 |
| 16 | 235683\_at | NA | SESN3 | sestrin 3 | 143686 | ENSG00000149212 |
| 17 | 226680\_at | NA | IKZF5 | IKAROS family zinc finger 5 (Pegasus) | 64376 | ENSG00000095574 |
| 18 | 222243\_s\_at | NA | TOB2 | transducer of ERBB2, 2 | 10766 | ENSG00000183864 |
| 19 | 238020\_at | NA | PSMC2 | proteasome (prosome, macropain) 26S subunit, ATPase, 2 | 5701 | ENSG00000161057 |
| 20 | 230379\_x\_at | NA | C2orf56 | chromosome 2 open reading frame 56 | 55471 | ENSG00000003509 |
| 21 | 232068\_s\_at | NA | TLR4 | toll-like receptor 4 | 7099 | ENSG00000136869 |
| 22 | 218871\_x\_at | NA | CSGALNACT2 | chondroitin sulfate N-acetylgalactosaminyltransferase 2 | 55454 | ENSG00000169826 |
| 23 | 204299\_at | NA | SRSF10 | serine/arginine-rich splicing factor 10 | 10772 | ENSG00000188529 |
| 24 | 229943\_at | NA | TRIM13 | tripartite motif containing 13 | 10206 | ENSG00000204977 |
| 25 | 227900\_at | NA | CBLB | Cbl proto-oncogene, E3 ubiquitin protein ligase B | 868 | ENSG00000114423 |
| 26 | 222848\_at | NA | CENPK | centromere protein K | 64105 | ENSG00000123219 |
| 27 | 205283\_at | NA | FKTN | fukutin | 2218 | ENSG00000106692 |
| 28 | 223200\_s\_at | NA | LSG1 | large subunit GTPase 1 homolog (S. cerevisiae) | 55341 | ENSG00000041802 |
| 29 | 213404\_s\_at | NA | RHEB | Ras homolog enriched in brain | 6009 | ENSG00000106615 |
| 30 | 218401\_s\_at | NA | ZNF281 | zinc finger protein 281 | 23528 | ENSG00000162702 |
| 31 | 228785\_at | NA | ZNF281 | zinc finger protein 281 | 23528 | ENSG00000162702 |
| 32 | 213418\_at | NA | HSPA6 | heat shock 70kDa protein 6 (HSP70B') | 3310 | ENSG00000173110 |
| 33 | 202861\_at | NA | PER1 | period homolog 1 (Drosophila) | 5187 | ENSG00000179094 |
| 34 | 203310\_at | NA | STXBP3 | syntaxin binding protein 3 | 6814 | ENSG00000116266 |
| 35 | 201088\_at | NA | KPNA2 | karyopherin alpha 2 (RAG cohort 1, importin alpha 1) | 3838 | ENSG00000182481 |
| 36 | 1553132\_a\_at | NA | TC2N | tandem C2 domains, nuclear | 123036 | ENSG00000165929 |
| 37 | 229431\_at | NA | RFXAP | regulatory factor X-associated protein | 5994 | ENSG00000133111 |
| 38 | 36711\_at | NA | MAFF | v-maf musculoaponeurotic fibrosarcoma oncogene homolog F (avian) | 23764 | ENSG00000185022 |
| 39 | 207545\_s\_at | NA | NUMB | numb homolog (Drosophila) | 8650 | ENSG00000133961 |
| 40 | 212847\_at | NA | FUBP1 | far upstream element (FUSE) binding protein 1 | 8880 | ENSG00000162613 |
| 41 | 227391\_x\_at | NA | LRRFIP1 | leucine rich repeat (in FLII) interacting protein 1 | 9208 | ENSG00000124831 |
| 42 | 222876\_s\_at | NA | ADAP2 | ArfGAP with dual PH domains 2 | 55803 | ENSG00000184060 |
| 43 | 202375\_at | NA | SEC24D | SEC24 family, member D (S. cerevisiae) | 9871 | ENSG00000150961 |
| 44 | 214917\_at | NA | PRKAA1 | protein kinase, AMP-activated, alpha 1 catalytic subunit | 5562 | ENSG00000132356 |
| 45 | 202887\_s\_at | NA | DDIT4 | DNA-damage-inducible transcript 4 | 54541 | ENSG00000168209 |
| 46 | 202904\_s\_at | NA | LSM5 | LSM5 homolog, U6 small nuclear RNA associated (S. cerevisiae) | 23658 | ENSG00000106355 |
| 47 | 222765\_x\_at | NA | ESF1 | ESF1, nucleolar pre-rRNA processing protein, homolog (S. cerevisiae) | 51575 | ENSG00000089048 |
| 48 | 226965\_at | NA | FAM116A | family with sequence similarity 116, member A | 201627 | ENSG00000174839 |
| 49 | 208078\_s\_at | NA | SIK1 | salt-inducible kinase 1 | 150094 | ENSG00000142178 |
| 50 | 1555962\_at | NA | B3GNT7 | UDP-GlcNAc:betaGal beta-1,3-N-acetylglucosaminyltransferase 7 | 93010 | ENSG00000156966 |
| 51 | 1555274\_a\_at | NA | EPT1 | ethanolaminephosphotransferase 1 (CDP-ethanolamine-specific) | 85465 | ENSG00000138018 |
| 52 | 226279\_at | NA | PRSS23 | protease, serine, 23 | 11098 | ENSG00000150687 |
| 53 | 238199\_x\_at | NA | COX3 | cytochrome c oxidase III | 4514 | NULL |
| 54 | 1555878\_at | NA | RPS24 | ribosomal protein S24 | 6229 | ENSG00000138326 |
| 55 | 223266\_at | NA | STRADB | STE20-related kinase adaptor beta | 55437 | ENSG00000082146 |
| 56 | 231863\_at | NA | ING3 | inhibitor of growth family, member 3 | 54556 | ENSG00000071243 |
| 57 | 211560\_s\_at | NA | ALAS2 | aminolevulinate, delta-, synthase 2 | 212 | ENSG00000158578 |
| 58 | 209686\_at | NA | S100B | S100 calcium binding protein B | 6285 | ENSG00000160307 |
| 59 | 205063\_at | NA | GEMIN2 | gem (nuclear organelle) associated protein 2 | 8487 | ENSG00000092208 |
| 60 | 203634\_s\_at | NA | CPT1A | carnitine palmitoyltransferase 1A (liver) | 1374 | ENSG00000110090 |
| 61 | 204995\_at | NA | CDK5R1 | cyclin-dependent kinase 5, regulatory subunit 1 (p35) | 8851 | ENSG00000176749 |
| 62 | 228063\_s\_at | NA | NAP1L5 | nucleosome assembly protein 1-like 5 | 266812 | ENSG00000177432 |
| 63 | 228062\_at | NA | NAP1L5 | nucleosome assembly protein 1-like 5 | 266812 | ENSG00000177432 |
| 64 | 218411\_s\_at | NA | MBIP | MAP3K12 binding inhibitory protein 1 | 51562 | ENSG00000151332 |
| 65 | 203574\_at | NA | NFIL3 | nuclear factor, interleukin 3 regulated | 4783 | ENSG00000165030 |
| 66 | 230559\_x\_at | NA | FGD4 | FYVE, RhoGEF and PH domain containing 4 | 121512 | ENSG00000139132 |
| 67 | 238614\_x\_at | NA | ZNF430 | zinc finger protein 430 | 80264 | ENSG00000118620 |
| 68 | 216248\_s\_at | NA | NR4A2 | nuclear receptor subfamily 4, group A, member 2 | 4929 | ENSG00000153234 |
| 69 | 204621\_s\_at | NA | NR4A2 | nuclear receptor subfamily 4, group A, member 2 | 4929 | ENSG00000153234 |
| 70 | 204622\_x\_at | NA | NR4A2 | nuclear receptor subfamily 4, group A, member 2 | 4929 | ENSG00000153234 |
| 71 | 232044\_at | NA | RBBP6 | retinoblastoma binding protein 6 | 5930 | ENSG00000122257 |
| 72 | 200608\_s\_at | NA | RAD21 | RAD21 homolog (S. pombe) | 5885 | ENSG00000164754 |
| 73 | 202221\_s\_at | NA | EP300 | E1A binding protein p300 | 2033 | ENSG00000100393 |
| 74 | 206919\_at | NA | ELK4 | ELK4, ETS-domain protein (SRF accessory protein 1) | 2005 | ENSG00000158711 |
| 75 | 201304\_at | NA | NDUFA5 | NADH dehydrogenase (ubiquinone) 1 alpha subcomplex, 5, 13kDa | 4698 | ENSG00000128609 |
| 76 | 225760\_at | NA | MYSM1 | Myb-like, SWIRM and MPN domains 1 | 114803 | ENSG00000162601 |
| 77 | 222310\_at | NA | SCAF4 | SR-related CTD-associated factor 4 | 57466 | ENSG00000156304 |
| 78 | 213725\_x\_at | NA | XYLT1 | xylosyltransferase I | 64131 | ENSG00000103489 |
| 79 | 202979\_s\_at | NA | CREBZF | CREB/ATF bZIP transcription factor | 58487 | ENSG00000137504 |
| 80 | 233019\_at | NA | CNOT7 | CCR4-NOT transcription complex, subunit 7 | 29883 | ENSG00000198791 |
| 81 | 200602\_at | NA | APP | amyloid beta (A4) precursor protein | 351 | ENSG00000142192 |
| 82 | 225580\_at | NA | MRPL50 | mitochondrial ribosomal protein L50 | 54534 | ENSG00000136897 |
| 83 | 225916\_at | NA | ZNF131 | zinc finger protein 131 | 7690 | ENSG00000172262 |
| 84 | 214741\_at | NA | ZNF131 | zinc finger protein 131 | 7690 | ENSG00000172262 |
| 85 | 201177\_s\_at | NA | UBA2 | ubiquitin-like modifier activating enzyme 2 | 10054 | ENSG00000126261 |
| 86 | 227375\_at | NA | ANKRD13C | ankyrin repeat domain 13C | 81573 | ENSG00000118454 |
| 87 | 202422\_s\_at | NA | ACSL4 | acyl-CoA synthetase long-chain family member 4 | 2182 | ENSG00000068366 |
| 88 | 214590\_s\_at | NA | UBE2D1 | ubiquitin-conjugating enzyme E2D 1 | 7321 | ENSG00000072401 |
| 89 | 221768\_at | NA | SFPQ | splicing factor proline/glutamine-rich | 6421 | ENSG00000116560 |
| 90 | 226931\_at | NA | TMTC1 | transmembrane and tetratricopeptide repeat containing 1 | 83857 | ENSG00000133687 |
| 91 | 211698\_at | NA | EID1 | EP300 interacting inhibitor of differentiation 1 | 23741 | ENSG00000255302 |
| 92 | 240413\_at | NA | PYHIN1 | pyrin and HIN domain family, member 1 | 149628 | ENSG00000163564 |
| 93 | 236539\_at | NA | PTPN22 | protein tyrosine phosphatase, non-receptor type 22 (lymphoid) | 26191 | ENSG00000134242 |
| 94 | 226472\_at | NA | PPIL4 | peptidylprolyl isomerase (cyclophilin)-like 4 | 85313 | ENSG00000131013 |
| 95 | 211546\_x\_at | NA | SNCA | synuclein, alpha (non A4 component of amyloid precursor) | 6622 | ENSG00000145335 |
| 96 | 207827\_x\_at | NA | SNCA | synuclein, alpha (non A4 component of amyloid precursor) | 6622 | ENSG00000145335 |
| 97 | 236081\_at | NA | SNCA | synuclein, alpha (non A4 component of amyloid precursor) | 6622 | ENSG00000145335 |
| 98 | 204467\_s\_at | NA | SNCA | synuclein, alpha (non A4 component of amyloid precursor) | 6622 | ENSG00000145335 |
| 99 | 204466\_s\_at | NA | SNCA | synuclein, alpha (non A4 component of amyloid precursor) | 6622 | ENSG00000145335 |
| 100 | 213998\_s\_at | NA | DDX17 | DEAD (Asp-Glu-Ala-Asp) box helicase 17 | 10521 | ENSG00000100201 |
| 101 | 208719\_s\_at | NA | DDX17 | DEAD (Asp-Glu-Ala-Asp) box helicase 17 | 10521 | ENSG00000100201 |
| 102 | 208151\_x\_at | NA | DDX17 | DEAD (Asp-Glu-Ala-Asp) box helicase 17 | 10521 | ENSG00000100201 |
| 103 | 1555960\_at | NA | HINT1 | histidine triad nucleotide binding protein 1 | 3094 | ENSG00000169567 |
| 104 | 224009\_x\_at | NA | DHRS9 | dehydrogenase/reductase (SDR family) member 9 | 10170 | ENSG00000073737 |
| 105 | 223952\_x\_at | NA | DHRS9 | dehydrogenase/reductase (SDR family) member 9 | 10170 | ENSG00000073737 |
| 106 | 213524\_s\_at | NA | G0S2 | G0/G1switch 2 | 50486 | ENSG00000123689 |
| 107 | 201407\_s\_at | NA | PPP1CB | protein phosphatase 1, catalytic subunit, beta isozyme | 5500 | ENSG00000213639 |
| 108 | 223649\_s\_at | NA | SLC25A39 | solute carrier family 25, member 39 | 51629 | ENSG00000013306 |
| 109 | 203603\_s\_at | NA | ZEB2 | zinc finger E-box binding homeobox 2 | 9839 | ENSG00000169554 |
| 110 | 204794\_at | NA | DUSP2 | dual specificity phosphatase 2 | 1844 | ENSG00000158050 |
| 111 | 206108\_s\_at | NA | SRSF6 | serine/arginine-rich splicing factor 6 | 6431 | ENSG00000124193 |
| 112 | 226366\_at | NA | SHPRH | SNF2 histone linker PHD RING helicase, E3 ubiquitin protein ligase | 257218 | ENSG00000146414 |
| 113 | 212989\_at | NA | SGMS1 | sphingomyelin synthase 1 | 259230 | ENSG00000198964 |
| 114 | 217851\_s\_at | NA | SLMO2 | slowmo homolog 2 (Drosophila) | 51012 | ENSG00000101166 |
| 115 | 230380\_at | NA | THAP2 | THAP domain containing, apoptosis associated protein 2 | 83591 | ENSG00000173451 |
| 116 | 202843\_at | NA | DNAJB9 | DnaJ (Hsp40) homolog, subfamily B, member 9 | 4189 | ENSG00000128590 |
| 117 | 208762\_at | NA | SUMO1 | SMT3 suppressor of mif two 3 homolog 1 (S. cerevisiae) | 7341 | ENSG00000116030 |
| 118 | 203358\_s\_at | NA | EZH2 | enhancer of zeste homolog 2 (Drosophila) | 2146 | ENSG00000106462 |
| 119 | 1558233\_s\_at | NA | ATF1 | activating transcription factor 1 | 466 | ENSG00000123268 |
| 120 | 216929\_x\_at | NA | ABO | ABO blood group (transferase A, alpha 1-3-N-acetylgalactosaminyltransferase; transferase B, alpha 1-3-galactosyltransferase) | 28 | NULL |
| 121 | 204176\_at | NA | KLHL20 | kelch-like 20 (Drosophila) | 27252 | ENSG00000076321 |
| 122 | 1569136\_at | NA | MGAT4A | mannosyl (alpha-1,3-)-glycoprotein beta-1,4-N-acetylglucosaminyltransferase, isozyme A | 11320 | ENSG00000071073 |
| 123 | 203049\_s\_at | NA | TTC37 | tetratricopeptide repeat domain 37 | 9652 | ENSG00000198677 |
| 124 | 212637\_s\_at | NA | WWP1 | WW domain containing E3 ubiquitin protein ligase 1 | 11059 | ENSG00000123124 |
| 125 | 203543\_s\_at | NA | KLF9 | Kruppel-like factor 9 | 687 | ENSG00000119138 |
| 126 | 212579\_at | NA | SMCHD1 | structural maintenance of chromosomes flexible hinge domain containing 1 | 23347 | ENSG00000101596 |
| 127 | 211760\_s\_at | NA | VAMP4 | vesicle-associated membrane protein 4 | 8674 | ENSG00000117533 |
| 128 | 202644\_s\_at | NA | TNFAIP3 | tumor necrosis factor, alpha-induced protein 3 | 7128 | ENSG00000118503 |
| 129 | 202203\_s\_at | NA | AMFR | autocrine motility factor receptor, E3 ubiquitin protein ligase | 267 | ENSG00000159461 |
| 130 | 242960\_at | NA | EPC2 | enhancer of polycomb homolog 2 (Drosophila) | 26122 | ENSG00000135999 |
| 131 | 202933\_s\_at | NA | YES1 | v-yes-1 Yamaguchi sarcoma viral oncogene homolog 1 | 7525 | ENSG00000176105 |
| 132 | 230875\_s\_at | NA | ATP11A | ATPase, class VI, type 11A | 23250 | ENSG00000068650 |
| 133 | 222317\_at | NA | PDE3B | phosphodiesterase 3B, cGMP-inhibited | 5140 | ENSG00000152270 |
| 134 | 1558956\_s\_at | NA | IFT80 | intraflagellar transport 80 homolog (Chlamydomonas) | 57560 | ENSG00000248710 ENSG00000068885 |
| 135 | 1557257\_at | NA | BCL10 | B-cell CLL/lymphoma 10 | 8915 | ENSG00000142867 |
| 136 | 201091\_s\_at | NA | CBX3 | chromobox homolog 3 | 11335 | ENSG00000122565 |
| 137 | 1559975\_at | NA | BTG1 | B-cell translocation gene 1, anti-proliferative | 694 | ENSG00000133639 |
| 138 | 1555476\_at | NA | IREB2 | iron-responsive element binding protein 2 | 3658 | ENSG00000136381 |
| 139 | 203884\_s\_at | NA | RAB11FIP2 | RAB11 family interacting protein 2 (class I) | 22841 | ENSG00000107560 |
| 140 | 227012\_at | NA | SLC25A40 | solute carrier family 25, member 40 | 55972 | ENSG00000075303 |
| 141 | 219304\_s\_at | NA | PDGFD | platelet derived growth factor D | 80310 | ENSG00000170962 |
| 142 | 214012\_at | NA | ERAP1 | endoplasmic reticulum aminopeptidase 1 | 51752 | ENSG00000164307 |
| 143 | 213372\_at | NA | PAQR3 | progestin and adipoQ receptor family member III | 152559 | ENSG00000163291 |
| 144 | 212633\_at | NA | UFL1 | UFM1-specific ligase 1 | 23376 | ENSG00000014123 |
| 145 | 207300\_s\_at | NA | F7 | coagulation factor VII (serum prothrombin conversion accelerator) | 2155 | ENSG00000057593 |

  
  

| **Database:cellular component      &nbspName:cytosol      &nbspID:GO:0005829** | | | | | | |
| --- | --- | --- | --- | --- | --- | --- |
| C=2312; O=46; E=27.65; R=1.66; rawP=0.0003; adjP=0.0175 | | | | | | |
| Index | UserID | Value | Gene Symbol | Gene Name | EntrezGene | Ensembl |
| 1 | 240336\_at | NA | HBM | hemoglobin, mu | 3042 | ENSG00000206177 |
| 2 | 236140\_at | NA | GCLM | glutamate-cysteine ligase, modifier subunit | 2730 | ENSG00000023909 |
| 3 | 202375\_at | NA | SEC24D | SEC24 family, member D (S. cerevisiae) | 9871 | ENSG00000150961 |
| 4 | 235412\_at | NA | ARHGEF7 | Rho guanine nucleotide exchange factor (GEF) 7 | 8874 | ENSG00000102606 |
| 5 | 214917\_at | NA | PRKAA1 | protein kinase, AMP-activated, alpha 1 catalytic subunit | 5562 | ENSG00000132356 |
| 6 | 201016\_at | NA | EIF1AX | eukaryotic translation initiation factor 1A, X-linked | 1964 | ENSG00000173674 |
| 7 | 213786\_at | NA | TAX1BP1 | Tax1 (human T-cell leukemia virus type I) binding protein 1 | 8887 | ENSG00000106052 |
| 8 | 224453\_s\_at | NA | ETNK1 | ethanolamine kinase 1 | 55500 | ENSG00000139163 |
| 9 | 225290\_at | NA | ETNK1 | ethanolamine kinase 1 | 55500 | ENSG00000139163 |
| 10 | 1554309\_at | NA | EIF4G3 | eukaryotic translation initiation factor 4 gamma, 3 | 8672 | ENSG00000075151 |
| 11 | 204187\_at | NA | GMPR | guanosine monophosphate reductase | 2766 | ENSG00000137198 |
| 12 | 233019\_at | NA | CNOT7 | CCR4-NOT transcription complex, subunit 7 | 29883 | ENSG00000198791 |
| 13 | 213225\_at | NA | PPM1B | protein phosphatase, Mg2+/Mn2+ dependent, 1B | 5495 | ENSG00000138032 |
| 14 | 200602\_at | NA | APP | amyloid beta (A4) precursor protein | 351 | ENSG00000142192 |
| 15 | 1558692\_at | NA | C1orf85 | chromosome 1 open reading frame 85 | 112770 | ENSG00000198715 |
| 16 | 217739\_s\_at | NA | NAMPT | nicotinamide phosphoribosyltransferase | 10135 | ENSG00000105835 |
| 17 | 202904\_s\_at | NA | LSM5 | LSM5 homolog, U6 small nuclear RNA associated (S. cerevisiae) | 23658 | ENSG00000106355 |
| 18 | 209451\_at | NA | TANK | TRAF family member-associated NFKB activator | 10010 | ENSG00000136560 |
| 19 | 238020\_at | NA | PSMC2 | proteasome (prosome, macropain) 26S subunit, ATPase, 2 | 5701 | ENSG00000161057 |
| 20 | 222858\_s\_at | NA | DAPP1 | dual adaptor of phosphotyrosine and 3-phosphoinositides | 27071 | ENSG00000070190 |
| 21 | 212637\_s\_at | NA | WWP1 | WW domain containing E3 ubiquitin protein ligase 1 | 11059 | ENSG00000123124 |
| 22 | 206834\_at | NA | HBD | hemoglobin, delta | 3045 | ENSG00000223609 |
| 23 | 213459\_at | NA | RPL37A | ribosomal protein L37a | 6168 | ENSG00000197756 |
| 24 | 214590\_s\_at | NA | UBE2D1 | ubiquitin-conjugating enzyme E2D 1 | 7321 | ENSG00000072401 |
| 25 | 208078\_s\_at | NA | SIK1 | salt-inducible kinase 1 | 150094 | ENSG00000142178 |
| 26 | 201437\_s\_at | NA | EIF4E | eukaryotic translation initiation factor 4E | 1977 | ENSG00000151247 |
| 27 | 202644\_s\_at | NA | TNFAIP3 | tumor necrosis factor, alpha-induced protein 3 | 7128 | ENSG00000118503 |
| 28 | 207794\_at | NA | CCR2 | chemokine (C-C motif) receptor 2 | 729230 | ENSG00000121807 |
| 29 | 227900\_at | NA | CBLB | Cbl proto-oncogene, E3 ubiquitin protein ligase B | 868 | ENSG00000114423 |
| 30 | 1555878\_at | NA | RPS24 | ribosomal protein S24 | 6229 | ENSG00000138326 |
| 31 | 223266\_at | NA | STRADB | STE20-related kinase adaptor beta | 55437 | ENSG00000082146 |
| 32 | 202464\_s\_at | NA | PFKFB3 | 6-phosphofructo-2-kinase/fructose-2,6-biphosphatase 3 | 5209 | ENSG00000170525 |
| 33 | 222848\_at | NA | CENPK | centromere protein K | 64105 | ENSG00000123219 |
| 34 | 202933\_s\_at | NA | YES1 | v-yes-1 Yamaguchi sarcoma viral oncogene homolog 1 | 7525 | ENSG00000176105 |
| 35 | 205063\_at | NA | GEMIN2 | gem (nuclear organelle) associated protein 2 | 8487 | ENSG00000092208 |
| 36 | 222317\_at | NA | PDE3B | phosphodiesterase 3B, cGMP-inhibited | 5140 | ENSG00000152270 |
| 37 | 223200\_s\_at | NA | LSG1 | large subunit GTPase 1 homolog (S. cerevisiae) | 55341 | ENSG00000041802 |
| 38 | 213404\_s\_at | NA | RHEB | Ras homolog enriched in brain | 6009 | ENSG00000106615 |
| 39 | 204995\_at | NA | CDK5R1 | cyclin-dependent kinase 5, regulatory subunit 1 (p35) | 8851 | ENSG00000176749 |
| 40 | 1557257\_at | NA | BCL10 | B-cell CLL/lymphoma 10 | 8915 | ENSG00000142867 |
| 41 | 227697\_at | NA | SOCS3 | suppressor of cytokine signaling 3 | 9021 | ENSG00000184557 |
| 42 | 211546\_x\_at | NA | SNCA | synuclein, alpha (non A4 component of amyloid precursor) | 6622 | ENSG00000145335 |
| 43 | 207827\_x\_at | NA | SNCA | synuclein, alpha (non A4 component of amyloid precursor) | 6622 | ENSG00000145335 |
| 44 | 236081\_at | NA | SNCA | synuclein, alpha (non A4 component of amyloid precursor) | 6622 | ENSG00000145335 |
| 45 | 204467\_s\_at | NA | SNCA | synuclein, alpha (non A4 component of amyloid precursor) | 6622 | ENSG00000145335 |
| 46 | 204466\_s\_at | NA | SNCA | synuclein, alpha (non A4 component of amyloid precursor) | 6622 | ENSG00000145335 |
| 47 | 1555476\_at | NA | IREB2 | iron-responsive element binding protein 2 | 3658 | ENSG00000136381 |
| 48 | 214012\_at | NA | ERAP1 | endoplasmic reticulum aminopeptidase 1 | 51752 | ENSG00000164307 |
| 49 | 203310\_at | NA | STXBP3 | syntaxin binding protein 3 | 6814 | ENSG00000116266 |
| 50 | 230559\_x\_at | NA | FGD4 | FYVE, RhoGEF and PH domain containing 4 | 121512 | ENSG00000139132 |
| 51 | 201088\_at | NA | KPNA2 | karyopherin alpha 2 (RAG cohort 1, importin alpha 1) | 3838 | ENSG00000182481 |

  
  

| **Database:cellular component      &nbspName:Piccolo NuA4 histone acetyltransferase complex      &nbspID:GO:0032777** | | | | | | |
| --- | --- | --- | --- | --- | --- | --- |
| C=4; O=2; E=0.05; R=41.81; rawP=0.0008; adjP=0.0175 | | | | | | |
| Index | UserID | Value | Gene Symbol | Gene Name | EntrezGene | Ensembl |
| 1 | 231863\_at | NA | ING3 | inhibitor of growth family, member 3 | 54556 | ENSG00000071243 |
| 2 | 242960\_at | NA | EPC2 | enhancer of polycomb homolog 2 (Drosophila) | 26122 | ENSG00000135999 |

  
  

| **Database:cellular component      &nbspName:cell part      &nbspID:GO:0044464** | | | | | | |
| --- | --- | --- | --- | --- | --- | --- |
| C=13444; O=175; E=160.76; R=1.09; rawP=0.0005; adjP=0.0175 | | | | | | |
| Index | UserID | Value | Gene Symbol | Gene Name | EntrezGene | Ensembl |
| 1 | 228106\_at | NA | DCAF16 | DDB1 and CUL4 associated factor 16 | 54876 | ENSG00000163257 |
| 2 | 214658\_at | NA | TMED7 | transmembrane emp24 protein transport domain containing 7 | 51014 | ENSG00000134970 |
| 3 | 219312\_s\_at | NA | ZBTB10 | zinc finger and BTB domain containing 10 | 65986 | ENSG00000205189 |
| 4 | 227449\_at | NA | EPHA4 | EPH receptor A4 | 2043 | ENSG00000116106 |
| 5 | 201016\_at | NA | EIF1AX | eukaryotic translation initiation factor 1A, X-linked | 1964 | ENSG00000173674 |
| 6 | 235300\_x\_at | NA | RCHY1 | ring finger and CHY zinc finger domain containing 1, E3 ubiquitin protein ligase | 25898 | ENSG00000163743 |
| 7 | 226181\_at | NA | TUBE1 | tubulin, epsilon 1 | 51175 | ENSG00000074935 |
| 8 | 207078\_at | NA | MED6 | mediator complex subunit 6 | 10001 | ENSG00000133997 |
| 9 | 218701\_at | NA | LACTB2 | lactamase, beta 2 | 51110 | ENSG00000147592 |
| 10 | 202660\_at | NA | ITPR2 | inositol 1,4,5-trisphosphate receptor, type 2 | 3709 | ENSG00000123104 |
| 11 | 202219\_at | NA | SLC6A8 | solute carrier family 6 (neurotransmitter transporter, creatine), member 8 | 6535 | ENSG00000130821 |
| 12 | 222528\_s\_at | NA | SLC25A37 | solute carrier family 25 (mitochondrial iron transporter), member 37 | 51312 | ENSG00000147454 |
| 13 | 226928\_x\_at | NA | SLC25A37 | solute carrier family 25 (mitochondrial iron transporter), member 37 | 51312 | ENSG00000147454 |
| 14 | 228527\_s\_at | NA | SLC25A37 | solute carrier family 25 (mitochondrial iron transporter), member 37 | 51312 | ENSG00000147454 |
| 15 | 226179\_at | NA | SLC25A37 | solute carrier family 25 (mitochondrial iron transporter), member 37 | 51312 | ENSG00000147454 |
| 16 | 1558692\_at | NA | C1orf85 | chromosome 1 open reading frame 85 | 112770 | ENSG00000198715 |
| 17 | 203203\_s\_at | NA | KRR1 | KRR1, small subunit (SSU) processome component, homolog (yeast) | 11103 | ENSG00000111615 |
| 18 | 243916\_x\_at | NA | UBLCP1 | ubiquitin-like domain containing CTD phosphatase 1 | 134510 | ENSG00000164332 |
| 19 | 202314\_at | NA | CYP51A1 | cytochrome P450, family 51, subfamily A, polypeptide 1 | 1595 | ENSG00000001630 |
| 20 | 235683\_at | NA | SESN3 | sestrin 3 | 143686 | ENSG00000149212 |
| 21 | 226680\_at | NA | IKZF5 | IKAROS family zinc finger 5 (Pegasus) | 64376 | ENSG00000095574 |
| 22 | 238020\_at | NA | PSMC2 | proteasome (prosome, macropain) 26S subunit, ATPase, 2 | 5701 | ENSG00000161057 |
| 23 | 222243\_s\_at | NA | TOB2 | transducer of ERBB2, 2 | 10766 | ENSG00000183864 |
| 24 | 230379\_x\_at | NA | C2orf56 | chromosome 2 open reading frame 56 | 55471 | ENSG00000003509 |
| 25 | 213459\_at | NA | RPL37A | ribosomal protein L37a | 6168 | ENSG00000197756 |
| 26 | 205191\_at | NA | RP2 | retinitis pigmentosa 2 (X-linked recessive) | 6102 | ENSG00000102218 |
| 27 | 201437\_s\_at | NA | EIF4E | eukaryotic translation initiation factor 4E | 1977 | ENSG00000151247 |
| 28 | 232068\_s\_at | NA | TLR4 | toll-like receptor 4 | 7099 | ENSG00000136869 |
| 29 | 241881\_at | NA | OR2W3 | olfactory receptor, family 2, subfamily W, member 3 | 343171 | ENSG00000238243 |
| 30 | 218871\_x\_at | NA | CSGALNACT2 | chondroitin sulfate N-acetylgalactosaminyltransferase 2 | 55454 | ENSG00000169826 |
| 31 | 204299\_at | NA | SRSF10 | serine/arginine-rich splicing factor 10 | 10772 | ENSG00000188529 |
| 32 | 207996\_s\_at | NA | C18orf1 | chromosome 18 open reading frame 1 | 753 | ENSG00000168675 |
| 33 | 230170\_at | NA | OSM | oncostatin M | 5008 | ENSG00000099985 |
| 34 | 229943\_at | NA | TRIM13 | tripartite motif containing 13 | 10206 | ENSG00000204977 |
| 35 | 227900\_at | NA | CBLB | Cbl proto-oncogene, E3 ubiquitin protein ligase B | 868 | ENSG00000114423 |
| 36 | 222848\_at | NA | CENPK | centromere protein K | 64105 | ENSG00000123219 |
| 37 | 205283\_at | NA | FKTN | fukutin | 2218 | ENSG00000106692 |
| 38 | 223200\_s\_at | NA | LSG1 | large subunit GTPase 1 homolog (S. cerevisiae) | 55341 | ENSG00000041802 |
| 39 | 218401\_s\_at | NA | ZNF281 | zinc finger protein 281 | 23528 | ENSG00000162702 |
| 40 | 228785\_at | NA | ZNF281 | zinc finger protein 281 | 23528 | ENSG00000162702 |
| 41 | 213404\_s\_at | NA | RHEB | Ras homolog enriched in brain | 6009 | ENSG00000106615 |
| 42 | 213418\_at | NA | HSPA6 | heat shock 70kDa protein 6 (HSP70B') | 3310 | ENSG00000173110 |
| 43 | 223939\_at | NA | SUCNR1 | succinate receptor 1 | 56670 | ENSG00000198829 |
| 44 | 205900\_at | NA | KRT1 | keratin 1 | 3848 | ENSG00000167768 |
| 45 | 202861\_at | NA | PER1 | period homolog 1 (Drosophila) | 5187 | ENSG00000179094 |
| 46 | 203310\_at | NA | STXBP3 | syntaxin binding protein 3 | 6814 | ENSG00000116266 |
| 47 | 201088\_at | NA | KPNA2 | karyopherin alpha 2 (RAG cohort 1, importin alpha 1) | 3838 | ENSG00000182481 |
| 48 | 1553132\_a\_at | NA | TC2N | tandem C2 domains, nuclear | 123036 | ENSG00000165929 |
| 49 | 36711\_at | NA | MAFF | v-maf musculoaponeurotic fibrosarcoma oncogene homolog F (avian) | 23764 | ENSG00000185022 |
| 50 | 229431\_at | NA | RFXAP | regulatory factor X-associated protein | 5994 | ENSG00000133111 |
| 51 | 207545\_s\_at | NA | NUMB | numb homolog (Drosophila) | 8650 | ENSG00000133961 |
| 52 | 212847\_at | NA | FUBP1 | far upstream element (FUSE) binding protein 1 | 8880 | ENSG00000162613 |
| 53 | 240336\_at | NA | HBM | hemoglobin, mu | 3042 | ENSG00000206177 |
| 54 | 227391\_x\_at | NA | LRRFIP1 | leucine rich repeat (in FLII) interacting protein 1 | 9208 | ENSG00000124831 |
| 55 | 222876\_s\_at | NA | ADAP2 | ArfGAP with dual PH domains 2 | 55803 | ENSG00000184060 |
| 56 | 202375\_at | NA | SEC24D | SEC24 family, member D (S. cerevisiae) | 9871 | ENSG00000150961 |
| 57 | 214917\_at | NA | PRKAA1 | protein kinase, AMP-activated, alpha 1 catalytic subunit | 5562 | ENSG00000132356 |
| 58 | 204614\_at | NA | SERPINB2 | serpin peptidase inhibitor, clade B (ovalbumin), member 2 | 5055 | ENSG00000197632 |
| 59 | 217739\_s\_at | NA | NAMPT | nicotinamide phosphoribosyltransferase | 10135 | ENSG00000105835 |
| 60 | 202887\_s\_at | NA | DDIT4 | DNA-damage-inducible transcript 4 | 54541 | ENSG00000168209 |
| 61 | 206206\_at | NA | CD180 | CD180 molecule | 4064 | ENSG00000134061 |
| 62 | 202904\_s\_at | NA | LSM5 | LSM5 homolog, U6 small nuclear RNA associated (S. cerevisiae) | 23658 | ENSG00000106355 |
| 63 | 203552\_at | NA | MAP4K5 | mitogen-activated protein kinase kinase kinase kinase 5 | 11183 | ENSG00000012983 |
| 64 | 222765\_x\_at | NA | ESF1 | ESF1, nucleolar pre-rRNA processing protein, homolog (S. cerevisiae) | 51575 | ENSG00000089048 |
| 65 | 226965\_at | NA | FAM116A | family with sequence similarity 116, member A | 201627 | ENSG00000174839 |
| 66 | 208078\_s\_at | NA | SIK1 | salt-inducible kinase 1 | 150094 | ENSG00000142178 |
| 67 | 242109\_at | NA | SYTL3 | synaptotagmin-like 3 | 94120 | ENSG00000164674 |
| 68 | 1555962\_at | NA | B3GNT7 | UDP-GlcNAc:betaGal beta-1,3-N-acetylglucosaminyltransferase 7 | 93010 | ENSG00000156966 |
| 69 | 1555274\_a\_at | NA | EPT1 | ethanolaminephosphotransferase 1 (CDP-ethanolamine-specific) | 85465 | ENSG00000138018 |
| 70 | 202498\_s\_at | NA | SLC2A3 | solute carrier family 2 (facilitated glucose transporter), member 3 | 6515 | ENSG00000059804 |
| 71 | 238199\_x\_at | NA | COX3 | cytochrome c oxidase III | 4514 | NULL |
| 72 | 226279\_at | NA | PRSS23 | protease, serine, 23 | 11098 | ENSG00000150687 |
| 73 | 225189\_s\_at | NA | RAPH1 | Ras association (RalGDS/AF-6) and pleckstrin homology domains 1 | 65059 | ENSG00000173166 |
| 74 | 231863\_at | NA | ING3 | inhibitor of growth family, member 3 | 54556 | ENSG00000071243 |
| 75 | 223266\_at | NA | STRADB | STE20-related kinase adaptor beta | 55437 | ENSG00000082146 |
| 76 | 1555878\_at | NA | RPS24 | ribosomal protein S24 | 6229 | ENSG00000138326 |
| 77 | 209686\_at | NA | S100B | S100 calcium binding protein B | 6285 | ENSG00000160307 |
| 78 | 211560\_s\_at | NA | ALAS2 | aminolevulinate, delta-, synthase 2 | 212 | ENSG00000158578 |
| 79 | 205063\_at | NA | GEMIN2 | gem (nuclear organelle) associated protein 2 | 8487 | ENSG00000092208 |
| 80 | 203634\_s\_at | NA | CPT1A | carnitine palmitoyltransferase 1A (liver) | 1374 | ENSG00000110090 |
| 81 | 204995\_at | NA | CDK5R1 | cyclin-dependent kinase 5, regulatory subunit 1 (p35) | 8851 | ENSG00000176749 |
| 82 | 228063\_s\_at | NA | NAP1L5 | nucleosome assembly protein 1-like 5 | 266812 | ENSG00000177432 |
| 83 | 228062\_at | NA | NAP1L5 | nucleosome assembly protein 1-like 5 | 266812 | ENSG00000177432 |
| 84 | 218411\_s\_at | NA | MBIP | MAP3K12 binding inhibitory protein 1 | 51562 | ENSG00000151332 |
| 85 | 236487\_at | NA | SCLT1 | sodium channel and clathrin linker 1 | 132320 | ENSG00000151466 |
| 86 | 203574\_at | NA | NFIL3 | nuclear factor, interleukin 3 regulated | 4783 | ENSG00000165030 |
| 87 | 202232\_s\_at | NA | EIF3M | eukaryotic translation initiation factor 3, subunit M | 10480 | ENSG00000149100 |
| 88 | 205767\_at | NA | EREG | epiregulin | 2069 | ENSG00000124882 |
| 89 | 238614\_x\_at | NA | ZNF430 | zinc finger protein 430 | 80264 | ENSG00000118620 |
| 90 | 230559\_x\_at | NA | FGD4 | FYVE, RhoGEF and PH domain containing 4 | 121512 | ENSG00000139132 |
| 91 | 216248\_s\_at | NA | NR4A2 | nuclear receptor subfamily 4, group A, member 2 | 4929 | ENSG00000153234 |
| 92 | 204621\_s\_at | NA | NR4A2 | nuclear receptor subfamily 4, group A, member 2 | 4929 | ENSG00000153234 |
| 93 | 204622\_x\_at | NA | NR4A2 | nuclear receptor subfamily 4, group A, member 2 | 4929 | ENSG00000153234 |
| 94 | 222309\_at | NA | C6orf62 | chromosome 6 open reading frame 62 | 81688 | ENSG00000112308 |
| 95 | 232044\_at | NA | RBBP6 | retinoblastoma binding protein 6 | 5930 | ENSG00000122257 |
| 96 | 200608\_s\_at | NA | RAD21 | RAD21 homolog (S. pombe) | 5885 | ENSG00000164754 |
| 97 | 202221\_s\_at | NA | EP300 | E1A binding protein p300 | 2033 | ENSG00000100393 |
| 98 | 206919\_at | NA | ELK4 | ELK4, ETS-domain protein (SRF accessory protein 1) | 2005 | ENSG00000158711 |
| 99 | 208121\_s\_at | NA | PTPRO | protein tyrosine phosphatase, receptor type, O | 5800 | ENSG00000151490 |
| 100 | 201304\_at | NA | NDUFA5 | NADH dehydrogenase (ubiquinone) 1 alpha subcomplex, 5, 13kDa | 4698 | ENSG00000128609 |
| 101 | 203821\_at | NA | HBEGF | heparin-binding EGF-like growth factor | 1839 | ENSG00000113070 |
| 102 | 225760\_at | NA | MYSM1 | Myb-like, SWIRM and MPN domains 1 | 114803 | ENSG00000162601 |
| 103 | 222310\_at | NA | SCAF4 | SR-related CTD-associated factor 4 | 57466 | ENSG00000156304 |
| 104 | 213725\_x\_at | NA | XYLT1 | xylosyltransferase I | 64131 | ENSG00000103489 |
| 105 | 224453\_s\_at | NA | ETNK1 | ethanolamine kinase 1 | 55500 | ENSG00000139163 |
| 106 | 225290\_at | NA | ETNK1 | ethanolamine kinase 1 | 55500 | ENSG00000139163 |
| 107 | 202979\_s\_at | NA | CREBZF | CREB/ATF bZIP transcription factor | 58487 | ENSG00000137504 |
| 108 | 204187\_at | NA | GMPR | guanosine monophosphate reductase | 2766 | ENSG00000137198 |
| 109 | 233019\_at | NA | CNOT7 | CCR4-NOT transcription complex, subunit 7 | 29883 | ENSG00000198791 |
| 110 | 213225\_at | NA | PPM1B | protein phosphatase, Mg2+/Mn2+ dependent, 1B | 5495 | ENSG00000138032 |
| 111 | 200602\_at | NA | APP | amyloid beta (A4) precursor protein | 351 | ENSG00000142192 |
| 112 | 225580\_at | NA | MRPL50 | mitochondrial ribosomal protein L50 | 54534 | ENSG00000136897 |
| 113 | 225916\_at | NA | ZNF131 | zinc finger protein 131 | 7690 | ENSG00000172262 |
| 114 | 214741\_at | NA | ZNF131 | zinc finger protein 131 | 7690 | ENSG00000172262 |
| 115 | 201177\_s\_at | NA | UBA2 | ubiquitin-like modifier activating enzyme 2 | 10054 | ENSG00000126261 |
| 116 | 206834\_at | NA | HBD | hemoglobin, delta | 3045 | ENSG00000223609 |
| 117 | 227375\_at | NA | ANKRD13C | ankyrin repeat domain 13C | 81573 | ENSG00000118454 |
| 118 | 202422\_s\_at | NA | ACSL4 | acyl-CoA synthetase long-chain family member 4 | 2182 | ENSG00000068366 |
| 119 | 214590\_s\_at | NA | UBE2D1 | ubiquitin-conjugating enzyme E2D 1 | 7321 | ENSG00000072401 |
| 120 | 221768\_at | NA | SFPQ | splicing factor proline/glutamine-rich | 6421 | ENSG00000116560 |
| 121 | 226931\_at | NA | TMTC1 | transmembrane and tetratricopeptide repeat containing 1 | 83857 | ENSG00000133687 |
| 122 | 211698\_at | NA | EID1 | EP300 interacting inhibitor of differentiation 1 | 23741 | ENSG00000255302 |
| 123 | 202464\_s\_at | NA | PFKFB3 | 6-phosphofructo-2-kinase/fructose-2,6-biphosphatase 3 | 5209 | ENSG00000170525 |
| 124 | 240413\_at | NA | PYHIN1 | pyrin and HIN domain family, member 1 | 149628 | ENSG00000163564 |
| 125 | 236539\_at | NA | PTPN22 | protein tyrosine phosphatase, non-receptor type 22 (lymphoid) | 26191 | ENSG00000134242 |
| 126 | 226472\_at | NA | PPIL4 | peptidylprolyl isomerase (cyclophilin)-like 4 | 85313 | ENSG00000131013 |
| 127 | 227697\_at | NA | SOCS3 | suppressor of cytokine signaling 3 | 9021 | ENSG00000184557 |
| 128 | 211546\_x\_at | NA | SNCA | synuclein, alpha (non A4 component of amyloid precursor) | 6622 | ENSG00000145335 |
| 129 | 207827\_x\_at | NA | SNCA | synuclein, alpha (non A4 component of amyloid precursor) | 6622 | ENSG00000145335 |
| 130 | 236081\_at | NA | SNCA | synuclein, alpha (non A4 component of amyloid precursor) | 6622 | ENSG00000145335 |
| 131 | 204467\_s\_at | NA | SNCA | synuclein, alpha (non A4 component of amyloid precursor) | 6622 | ENSG00000145335 |
| 132 | 204466\_s\_at | NA | SNCA | synuclein, alpha (non A4 component of amyloid precursor) | 6622 | ENSG00000145335 |
| 133 | 213998\_s\_at | NA | DDX17 | DEAD (Asp-Glu-Ala-Asp) box helicase 17 | 10521 | ENSG00000100201 |
| 134 | 208719\_s\_at | NA | DDX17 | DEAD (Asp-Glu-Ala-Asp) box helicase 17 | 10521 | ENSG00000100201 |
| 135 | 208151\_x\_at | NA | DDX17 | DEAD (Asp-Glu-Ala-Asp) box helicase 17 | 10521 | ENSG00000100201 |
| 136 | 1555960\_at | NA | HINT1 | histidine triad nucleotide binding protein 1 | 3094 | ENSG00000169567 |
| 137 | 224009\_x\_at | NA | DHRS9 | dehydrogenase/reductase (SDR family) member 9 | 10170 | ENSG00000073737 |
| 138 | 223952\_x\_at | NA | DHRS9 | dehydrogenase/reductase (SDR family) member 9 | 10170 | ENSG00000073737 |
| 139 | 213524\_s\_at | NA | G0S2 | G0/G1switch 2 | 50486 | ENSG00000123689 |
| 140 | 209795\_at | NA | CD69 | CD69 molecule | 969 | ENSG00000110848 |
| 141 | 201407\_s\_at | NA | PPP1CB | protein phosphatase 1, catalytic subunit, beta isozyme | 5500 | ENSG00000213639 |
| 142 | 223649\_s\_at | NA | SLC25A39 | solute carrier family 25, member 39 | 51629 | ENSG00000013306 |
| 143 | 203603\_s\_at | NA | ZEB2 | zinc finger E-box binding homeobox 2 | 9839 | ENSG00000169554 |
| 144 | 204794\_at | NA | DUSP2 | dual specificity phosphatase 2 | 1844 | ENSG00000158050 |
| 145 | 236140\_at | NA | GCLM | glutamate-cysteine ligase, modifier subunit | 2730 | ENSG00000023909 |
| 146 | 226366\_at | NA | SHPRH | SNF2 histone linker PHD RING helicase, E3 ubiquitin protein ligase | 257218 | ENSG00000146414 |
| 147 | 206108\_s\_at | NA | SRSF6 | serine/arginine-rich splicing factor 6 | 6431 | ENSG00000124193 |
| 148 | 235412\_at | NA | ARHGEF7 | Rho guanine nucleotide exchange factor (GEF) 7 | 8874 | ENSG00000102606 |
| 149 | 212989\_at | NA | SGMS1 | sphingomyelin synthase 1 | 259230 | ENSG00000198964 |
| 150 | 213786\_at | NA | TAX1BP1 | Tax1 (human T-cell leukemia virus type I) binding protein 1 | 8887 | ENSG00000106052 |
| 151 | 220018\_at | NA | CBLL1 | Cbl proto-oncogene, E3 ubiquitin protein ligase-like 1 | 79872 | ENSG00000105879 |
| 152 | 1554309\_at | NA | EIF4G3 | eukaryotic translation initiation factor 4 gamma, 3 | 8672 | ENSG00000075151 |
| 153 | 217851\_s\_at | NA | SLMO2 | slowmo homolog 2 (Drosophila) | 51012 | ENSG00000101166 |
| 154 | 230380\_at | NA | THAP2 | THAP domain containing, apoptosis associated protein 2 | 83591 | ENSG00000173451 |
| 155 | 210746\_s\_at | NA | EPB42 | erythrocyte membrane protein band 4.2 | 2038 | ENSG00000166947 |
| 156 | 216834\_at | NA | RGS1 | regulator of G-protein signaling 1 | 5996 | ENSG00000090104 |
| 157 | 202988\_s\_at | NA | RGS1 | regulator of G-protein signaling 1 | 5996 | ENSG00000090104 |
| 158 | 202843\_at | NA | DNAJB9 | DnaJ (Hsp40) homolog, subfamily B, member 9 | 4189 | ENSG00000128590 |
| 159 | 203358\_s\_at | NA | EZH2 | enhancer of zeste homolog 2 (Drosophila) | 2146 | ENSG00000106462 |
| 160 | 208762\_at | NA | SUMO1 | SMT3 suppressor of mif two 3 homolog 1 (S. cerevisiae) | 7341 | ENSG00000116030 |
| 161 | 1558233\_s\_at | NA | ATF1 | activating transcription factor 1 | 466 | ENSG00000123268 |
| 162 | 216929\_x\_at | NA | ABO | ABO blood group (transferase A, alpha 1-3-N-acetylgalactosaminyltransferase; transferase B, alpha 1-3-galactosyltransferase) | 28 | NULL |
| 163 | 204176\_at | NA | KLHL20 | kelch-like 20 (Drosophila) | 27252 | ENSG00000076321 |
| 164 | 1569136\_at | NA | MGAT4A | mannosyl (alpha-1,3-)-glycoprotein beta-1,4-N-acetylglucosaminyltransferase, isozyme A | 11320 | ENSG00000071073 |
| 165 | 209451\_at | NA | TANK | TRAF family member-associated NFKB activator | 10010 | ENSG00000136560 |
| 166 | 222858\_s\_at | NA | DAPP1 | dual adaptor of phosphotyrosine and 3-phosphoinositides | 27071 | ENSG00000070190 |
| 167 | 203049\_s\_at | NA | TTC37 | tetratricopeptide repeat domain 37 | 9652 | ENSG00000198677 |
| 168 | 212637\_s\_at | NA | WWP1 | WW domain containing E3 ubiquitin protein ligase 1 | 11059 | ENSG00000123124 |
| 169 | 203543\_s\_at | NA | KLF9 | Kruppel-like factor 9 | 687 | ENSG00000119138 |
| 170 | 212579\_at | NA | SMCHD1 | structural maintenance of chromosomes flexible hinge domain containing 1 | 23347 | ENSG00000101596 |
| 171 | 215716\_s\_at | NA | ATP2B1 | ATPase, Ca++ transporting, plasma membrane 1 | 490 | ENSG00000070961 |
| 172 | 211760\_s\_at | NA | VAMP4 | vesicle-associated membrane protein 4 | 8674 | ENSG00000117533 |
| 173 | 202644\_s\_at | NA | TNFAIP3 | tumor necrosis factor, alpha-induced protein 3 | 7128 | ENSG00000118503 |
| 174 | 202203\_s\_at | NA | AMFR | autocrine motility factor receptor, E3 ubiquitin protein ligase | 267 | ENSG00000159461 |
| 175 | 207794\_at | NA | CCR2 | chemokine (C-C motif) receptor 2 | 729230 | ENSG00000121807 |
| 176 | 242960\_at | NA | EPC2 | enhancer of polycomb homolog 2 (Drosophila) | 26122 | ENSG00000135999 |
| 177 | 230875\_s\_at | NA | ATP11A | ATPase, class VI, type 11A | 23250 | ENSG00000068650 |
| 178 | 202933\_s\_at | NA | YES1 | v-yes-1 Yamaguchi sarcoma viral oncogene homolog 1 | 7525 | ENSG00000176105 |
| 179 | 222317\_at | NA | PDE3B | phosphodiesterase 3B, cGMP-inhibited | 5140 | ENSG00000152270 |
| 180 | 1558956\_s\_at | NA | IFT80 | intraflagellar transport 80 homolog (Chlamydomonas) | 57560 | ENSG00000248710 ENSG00000068885 |
| 181 | 201091\_s\_at | NA | CBX3 | chromobox homolog 3 | 11335 | ENSG00000122565 |
| 182 | 1557257\_at | NA | BCL10 | B-cell CLL/lymphoma 10 | 8915 | ENSG00000142867 |
| 183 | 1559975\_at | NA | BTG1 | B-cell translocation gene 1, anti-proliferative | 694 | ENSG00000133639 |
| 184 | 1555476\_at | NA | IREB2 | iron-responsive element binding protein 2 | 3658 | ENSG00000136381 |
| 185 | 203884\_s\_at | NA | RAB11FIP2 | RAB11 family interacting protein 2 (class I) | 22841 | ENSG00000107560 |
| 186 | 227012\_at | NA | SLC25A40 | solute carrier family 25, member 40 | 55972 | ENSG00000075303 |
| 187 | 239143\_x\_at | NA | RNF138 | ring finger protein 138, E3 ubiquitin protein ligase | 51444 | ENSG00000134758 |
| 188 | 214012\_at | NA | ERAP1 | endoplasmic reticulum aminopeptidase 1 | 51752 | ENSG00000164307 |
| 189 | 219304\_s\_at | NA | PDGFD | platelet derived growth factor D | 80310 | ENSG00000170962 |
| 190 | 213372\_at | NA | PAQR3 | progestin and adipoQ receptor family member III | 152559 | ENSG00000163291 |
| 191 | 212633\_at | NA | UFL1 | UFM1-specific ligase 1 | 23376 | ENSG00000014123 |
| 192 | 207300\_s\_at | NA | F7 | coagulation factor VII (serum prothrombin conversion accelerator) | 2155 | ENSG00000057593 |

  
  

| **Database:cellular component      &nbspName:intracellular membrane-bounded organelle      &nbspID:GO:0043231** | | | | | | |
| --- | --- | --- | --- | --- | --- | --- |
| C=9117; O=130; E=109.02; R=1.19; rawP=0.0009; adjP=0.0175 | | | | | | |
| Index | UserID | Value | Gene Symbol | Gene Name | EntrezGene | Ensembl |
| 1 | 214658\_at | NA | TMED7 | transmembrane emp24 protein transport domain containing 7 | 51014 | ENSG00000134970 |
| 2 | 219312\_s\_at | NA | ZBTB10 | zinc finger and BTB domain containing 10 | 65986 | ENSG00000205189 |
| 3 | 227449\_at | NA | EPHA4 | EPH receptor A4 | 2043 | ENSG00000116106 |
| 4 | 235300\_x\_at | NA | RCHY1 | ring finger and CHY zinc finger domain containing 1, E3 ubiquitin protein ligase | 25898 | ENSG00000163743 |
| 5 | 207078\_at | NA | MED6 | mediator complex subunit 6 | 10001 | ENSG00000133997 |
| 6 | 202660\_at | NA | ITPR2 | inositol 1,4,5-trisphosphate receptor, type 2 | 3709 | ENSG00000123104 |
| 7 | 218701\_at | NA | LACTB2 | lactamase, beta 2 | 51110 | ENSG00000147592 |
| 8 | 222528\_s\_at | NA | SLC25A37 | solute carrier family 25 (mitochondrial iron transporter), member 37 | 51312 | ENSG00000147454 |
| 9 | 226928\_x\_at | NA | SLC25A37 | solute carrier family 25 (mitochondrial iron transporter), member 37 | 51312 | ENSG00000147454 |
| 10 | 228527\_s\_at | NA | SLC25A37 | solute carrier family 25 (mitochondrial iron transporter), member 37 | 51312 | ENSG00000147454 |
| 11 | 226179\_at | NA | SLC25A37 | solute carrier family 25 (mitochondrial iron transporter), member 37 | 51312 | ENSG00000147454 |
| 12 | 1558692\_at | NA | C1orf85 | chromosome 1 open reading frame 85 | 112770 | ENSG00000198715 |
| 13 | 203203\_s\_at | NA | KRR1 | KRR1, small subunit (SSU) processome component, homolog (yeast) | 11103 | ENSG00000111615 |
| 14 | 243916\_x\_at | NA | UBLCP1 | ubiquitin-like domain containing CTD phosphatase 1 | 134510 | ENSG00000164332 |
| 15 | 202314\_at | NA | CYP51A1 | cytochrome P450, family 51, subfamily A, polypeptide 1 | 1595 | ENSG00000001630 |
| 16 | 235683\_at | NA | SESN3 | sestrin 3 | 143686 | ENSG00000149212 |
| 17 | 226680\_at | NA | IKZF5 | IKAROS family zinc finger 5 (Pegasus) | 64376 | ENSG00000095574 |
| 18 | 222243\_s\_at | NA | TOB2 | transducer of ERBB2, 2 | 10766 | ENSG00000183864 |
| 19 | 238020\_at | NA | PSMC2 | proteasome (prosome, macropain) 26S subunit, ATPase, 2 | 5701 | ENSG00000161057 |
| 20 | 230379\_x\_at | NA | C2orf56 | chromosome 2 open reading frame 56 | 55471 | ENSG00000003509 |
| 21 | 232068\_s\_at | NA | TLR4 | toll-like receptor 4 | 7099 | ENSG00000136869 |
| 22 | 218871\_x\_at | NA | CSGALNACT2 | chondroitin sulfate N-acetylgalactosaminyltransferase 2 | 55454 | ENSG00000169826 |
| 23 | 204299\_at | NA | SRSF10 | serine/arginine-rich splicing factor 10 | 10772 | ENSG00000188529 |
| 24 | 229943\_at | NA | TRIM13 | tripartite motif containing 13 | 10206 | ENSG00000204977 |
| 25 | 227900\_at | NA | CBLB | Cbl proto-oncogene, E3 ubiquitin protein ligase B | 868 | ENSG00000114423 |
| 26 | 222848\_at | NA | CENPK | centromere protein K | 64105 | ENSG00000123219 |
| 27 | 205283\_at | NA | FKTN | fukutin | 2218 | ENSG00000106692 |
| 28 | 223200\_s\_at | NA | LSG1 | large subunit GTPase 1 homolog (S. cerevisiae) | 55341 | ENSG00000041802 |
| 29 | 213404\_s\_at | NA | RHEB | Ras homolog enriched in brain | 6009 | ENSG00000106615 |
| 30 | 218401\_s\_at | NA | ZNF281 | zinc finger protein 281 | 23528 | ENSG00000162702 |
| 31 | 228785\_at | NA | ZNF281 | zinc finger protein 281 | 23528 | ENSG00000162702 |
| 32 | 213418\_at | NA | HSPA6 | heat shock 70kDa protein 6 (HSP70B') | 3310 | ENSG00000173110 |
| 33 | 202861\_at | NA | PER1 | period homolog 1 (Drosophila) | 5187 | ENSG00000179094 |
| 34 | 203310\_at | NA | STXBP3 | syntaxin binding protein 3 | 6814 | ENSG00000116266 |
| 35 | 201088\_at | NA | KPNA2 | karyopherin alpha 2 (RAG cohort 1, importin alpha 1) | 3838 | ENSG00000182481 |
| 36 | 1553132\_a\_at | NA | TC2N | tandem C2 domains, nuclear | 123036 | ENSG00000165929 |
| 37 | 229431\_at | NA | RFXAP | regulatory factor X-associated protein | 5994 | ENSG00000133111 |
| 38 | 36711\_at | NA | MAFF | v-maf musculoaponeurotic fibrosarcoma oncogene homolog F (avian) | 23764 | ENSG00000185022 |
| 39 | 207545\_s\_at | NA | NUMB | numb homolog (Drosophila) | 8650 | ENSG00000133961 |
| 40 | 212847\_at | NA | FUBP1 | far upstream element (FUSE) binding protein 1 | 8880 | ENSG00000162613 |
| 41 | 227391\_x\_at | NA | LRRFIP1 | leucine rich repeat (in FLII) interacting protein 1 | 9208 | ENSG00000124831 |
| 42 | 222876\_s\_at | NA | ADAP2 | ArfGAP with dual PH domains 2 | 55803 | ENSG00000184060 |
| 43 | 202375\_at | NA | SEC24D | SEC24 family, member D (S. cerevisiae) | 9871 | ENSG00000150961 |
| 44 | 214917\_at | NA | PRKAA1 | protein kinase, AMP-activated, alpha 1 catalytic subunit | 5562 | ENSG00000132356 |
| 45 | 202887\_s\_at | NA | DDIT4 | DNA-damage-inducible transcript 4 | 54541 | ENSG00000168209 |
| 46 | 202904\_s\_at | NA | LSM5 | LSM5 homolog, U6 small nuclear RNA associated (S. cerevisiae) | 23658 | ENSG00000106355 |
| 47 | 222765\_x\_at | NA | ESF1 | ESF1, nucleolar pre-rRNA processing protein, homolog (S. cerevisiae) | 51575 | ENSG00000089048 |
| 48 | 226965\_at | NA | FAM116A | family with sequence similarity 116, member A | 201627 | ENSG00000174839 |
| 49 | 208078\_s\_at | NA | SIK1 | salt-inducible kinase 1 | 150094 | ENSG00000142178 |
| 50 | 1555962\_at | NA | B3GNT7 | UDP-GlcNAc:betaGal beta-1,3-N-acetylglucosaminyltransferase 7 | 93010 | ENSG00000156966 |
| 51 | 1555274\_a\_at | NA | EPT1 | ethanolaminephosphotransferase 1 (CDP-ethanolamine-specific) | 85465 | ENSG00000138018 |
| 52 | 226279\_at | NA | PRSS23 | protease, serine, 23 | 11098 | ENSG00000150687 |
| 53 | 238199\_x\_at | NA | COX3 | cytochrome c oxidase III | 4514 | NULL |
| 54 | 1555878\_at | NA | RPS24 | ribosomal protein S24 | 6229 | ENSG00000138326 |
| 55 | 223266\_at | NA | STRADB | STE20-related kinase adaptor beta | 55437 | ENSG00000082146 |
| 56 | 231863\_at | NA | ING3 | inhibitor of growth family, member 3 | 54556 | ENSG00000071243 |
| 57 | 211560\_s\_at | NA | ALAS2 | aminolevulinate, delta-, synthase 2 | 212 | ENSG00000158578 |
| 58 | 209686\_at | NA | S100B | S100 calcium binding protein B | 6285 | ENSG00000160307 |
| 59 | 205063\_at | NA | GEMIN2 | gem (nuclear organelle) associated protein 2 | 8487 | ENSG00000092208 |
| 60 | 203634\_s\_at | NA | CPT1A | carnitine palmitoyltransferase 1A (liver) | 1374 | ENSG00000110090 |
| 61 | 204995\_at | NA | CDK5R1 | cyclin-dependent kinase 5, regulatory subunit 1 (p35) | 8851 | ENSG00000176749 |
| 62 | 228063\_s\_at | NA | NAP1L5 | nucleosome assembly protein 1-like 5 | 266812 | ENSG00000177432 |
| 63 | 228062\_at | NA | NAP1L5 | nucleosome assembly protein 1-like 5 | 266812 | ENSG00000177432 |
| 64 | 218411\_s\_at | NA | MBIP | MAP3K12 binding inhibitory protein 1 | 51562 | ENSG00000151332 |
| 65 | 203574\_at | NA | NFIL3 | nuclear factor, interleukin 3 regulated | 4783 | ENSG00000165030 |
| 66 | 230559\_x\_at | NA | FGD4 | FYVE, RhoGEF and PH domain containing 4 | 121512 | ENSG00000139132 |
| 67 | 238614\_x\_at | NA | ZNF430 | zinc finger protein 430 | 80264 | ENSG00000118620 |
| 68 | 216248\_s\_at | NA | NR4A2 | nuclear receptor subfamily 4, group A, member 2 | 4929 | ENSG00000153234 |
| 69 | 204621\_s\_at | NA | NR4A2 | nuclear receptor subfamily 4, group A, member 2 | 4929 | ENSG00000153234 |
| 70 | 204622\_x\_at | NA | NR4A2 | nuclear receptor subfamily 4, group A, member 2 | 4929 | ENSG00000153234 |
| 71 | 232044\_at | NA | RBBP6 | retinoblastoma binding protein 6 | 5930 | ENSG00000122257 |
| 72 | 200608\_s\_at | NA | RAD21 | RAD21 homolog (S. pombe) | 5885 | ENSG00000164754 |
| 73 | 202221\_s\_at | NA | EP300 | E1A binding protein p300 | 2033 | ENSG00000100393 |
| 74 | 206919\_at | NA | ELK4 | ELK4, ETS-domain protein (SRF accessory protein 1) | 2005 | ENSG00000158711 |
| 75 | 201304\_at | NA | NDUFA5 | NADH dehydrogenase (ubiquinone) 1 alpha subcomplex, 5, 13kDa | 4698 | ENSG00000128609 |
| 76 | 225760\_at | NA | MYSM1 | Myb-like, SWIRM and MPN domains 1 | 114803 | ENSG00000162601 |
| 77 | 222310\_at | NA | SCAF4 | SR-related CTD-associated factor 4 | 57466 | ENSG00000156304 |
| 78 | 213725\_x\_at | NA | XYLT1 | xylosyltransferase I | 64131 | ENSG00000103489 |
| 79 | 202979\_s\_at | NA | CREBZF | CREB/ATF bZIP transcription factor | 58487 | ENSG00000137504 |
| 80 | 233019\_at | NA | CNOT7 | CCR4-NOT transcription complex, subunit 7 | 29883 | ENSG00000198791 |
| 81 | 200602\_at | NA | APP | amyloid beta (A4) precursor protein | 351 | ENSG00000142192 |
| 82 | 225580\_at | NA | MRPL50 | mitochondrial ribosomal protein L50 | 54534 | ENSG00000136897 |
| 83 | 225916\_at | NA | ZNF131 | zinc finger protein 131 | 7690 | ENSG00000172262 |
| 84 | 214741\_at | NA | ZNF131 | zinc finger protein 131 | 7690 | ENSG00000172262 |
| 85 | 201177\_s\_at | NA | UBA2 | ubiquitin-like modifier activating enzyme 2 | 10054 | ENSG00000126261 |
| 86 | 227375\_at | NA | ANKRD13C | ankyrin repeat domain 13C | 81573 | ENSG00000118454 |
| 87 | 202422\_s\_at | NA | ACSL4 | acyl-CoA synthetase long-chain family member 4 | 2182 | ENSG00000068366 |
| 88 | 214590\_s\_at | NA | UBE2D1 | ubiquitin-conjugating enzyme E2D 1 | 7321 | ENSG00000072401 |
| 89 | 221768\_at | NA | SFPQ | splicing factor proline/glutamine-rich | 6421 | ENSG00000116560 |
| 90 | 226931\_at | NA | TMTC1 | transmembrane and tetratricopeptide repeat containing 1 | 83857 | ENSG00000133687 |
| 91 | 211698\_at | NA | EID1 | EP300 interacting inhibitor of differentiation 1 | 23741 | ENSG00000255302 |
| 92 | 240413\_at | NA | PYHIN1 | pyrin and HIN domain family, member 1 | 149628 | ENSG00000163564 |
| 93 | 236539\_at | NA | PTPN22 | protein tyrosine phosphatase, non-receptor type 22 (lymphoid) | 26191 | ENSG00000134242 |
| 94 | 226472\_at | NA | PPIL4 | peptidylprolyl isomerase (cyclophilin)-like 4 | 85313 | ENSG00000131013 |
| 95 | 211546\_x\_at | NA | SNCA | synuclein, alpha (non A4 component of amyloid precursor) | 6622 | ENSG00000145335 |
| 96 | 207827\_x\_at | NA | SNCA | synuclein, alpha (non A4 component of amyloid precursor) | 6622 | ENSG00000145335 |
| 97 | 236081\_at | NA | SNCA | synuclein, alpha (non A4 component of amyloid precursor) | 6622 | ENSG00000145335 |
| 98 | 204467\_s\_at | NA | SNCA | synuclein, alpha (non A4 component of amyloid precursor) | 6622 | ENSG00000145335 |
| 99 | 204466\_s\_at | NA | SNCA | synuclein, alpha (non A4 component of amyloid precursor) | 6622 | ENSG00000145335 |
| 100 | 213998\_s\_at | NA | DDX17 | DEAD (Asp-Glu-Ala-Asp) box helicase 17 | 10521 | ENSG00000100201 |
| 101 | 208719\_s\_at | NA | DDX17 | DEAD (Asp-Glu-Ala-Asp) box helicase 17 | 10521 | ENSG00000100201 |
| 102 | 208151\_x\_at | NA | DDX17 | DEAD (Asp-Glu-Ala-Asp) box helicase 17 | 10521 | ENSG00000100201 |
| 103 | 1555960\_at | NA | HINT1 | histidine triad nucleotide binding protein 1 | 3094 | ENSG00000169567 |
| 104 | 224009\_x\_at | NA | DHRS9 | dehydrogenase/reductase (SDR family) member 9 | 10170 | ENSG00000073737 |
| 105 | 223952\_x\_at | NA | DHRS9 | dehydrogenase/reductase (SDR family) member 9 | 10170 | ENSG00000073737 |
| 106 | 213524\_s\_at | NA | G0S2 | G0/G1switch 2 | 50486 | ENSG00000123689 |
| 107 | 201407\_s\_at | NA | PPP1CB | protein phosphatase 1, catalytic subunit, beta isozyme | 5500 | ENSG00000213639 |
| 108 | 223649\_s\_at | NA | SLC25A39 | solute carrier family 25, member 39 | 51629 | ENSG00000013306 |
| 109 | 203603\_s\_at | NA | ZEB2 | zinc finger E-box binding homeobox 2 | 9839 | ENSG00000169554 |
| 110 | 204794\_at | NA | DUSP2 | dual specificity phosphatase 2 | 1844 | ENSG00000158050 |
| 111 | 206108\_s\_at | NA | SRSF6 | serine/arginine-rich splicing factor 6 | 6431 | ENSG00000124193 |
| 112 | 226366\_at | NA | SHPRH | SNF2 histone linker PHD RING helicase, E3 ubiquitin protein ligase | 257218 | ENSG00000146414 |
| 113 | 212989\_at | NA | SGMS1 | sphingomyelin synthase 1 | 259230 | ENSG00000198964 |
| 114 | 217851\_s\_at | NA | SLMO2 | slowmo homolog 2 (Drosophila) | 51012 | ENSG00000101166 |
| 115 | 230380\_at | NA | THAP2 | THAP domain containing, apoptosis associated protein 2 | 83591 | ENSG00000173451 |
| 116 | 202843\_at | NA | DNAJB9 | DnaJ (Hsp40) homolog, subfamily B, member 9 | 4189 | ENSG00000128590 |
| 117 | 208762\_at | NA | SUMO1 | SMT3 suppressor of mif two 3 homolog 1 (S. cerevisiae) | 7341 | ENSG00000116030 |
| 118 | 203358\_s\_at | NA | EZH2 | enhancer of zeste homolog 2 (Drosophila) | 2146 | ENSG00000106462 |
| 119 | 1558233\_s\_at | NA | ATF1 | activating transcription factor 1 | 466 | ENSG00000123268 |
| 120 | 216929\_x\_at | NA | ABO | ABO blood group (transferase A, alpha 1-3-N-acetylgalactosaminyltransferase; transferase B, alpha 1-3-galactosyltransferase) | 28 | NULL |
| 121 | 204176\_at | NA | KLHL20 | kelch-like 20 (Drosophila) | 27252 | ENSG00000076321 |
| 122 | 1569136\_at | NA | MGAT4A | mannosyl (alpha-1,3-)-glycoprotein beta-1,4-N-acetylglucosaminyltransferase, isozyme A | 11320 | ENSG00000071073 |
| 123 | 203049\_s\_at | NA | TTC37 | tetratricopeptide repeat domain 37 | 9652 | ENSG00000198677 |
| 124 | 212637\_s\_at | NA | WWP1 | WW domain containing E3 ubiquitin protein ligase 1 | 11059 | ENSG00000123124 |
| 125 | 203543\_s\_at | NA | KLF9 | Kruppel-like factor 9 | 687 | ENSG00000119138 |
| 126 | 212579\_at | NA | SMCHD1 | structural maintenance of chromosomes flexible hinge domain containing 1 | 23347 | ENSG00000101596 |
| 127 | 211760\_s\_at | NA | VAMP4 | vesicle-associated membrane protein 4 | 8674 | ENSG00000117533 |
| 128 | 202644\_s\_at | NA | TNFAIP3 | tumor necrosis factor, alpha-induced protein 3 | 7128 | ENSG00000118503 |
| 129 | 202203\_s\_at | NA | AMFR | autocrine motility factor receptor, E3 ubiquitin protein ligase | 267 | ENSG00000159461 |
| 130 | 242960\_at | NA | EPC2 | enhancer of polycomb homolog 2 (Drosophila) | 26122 | ENSG00000135999 |
| 131 | 202933\_s\_at | NA | YES1 | v-yes-1 Yamaguchi sarcoma viral oncogene homolog 1 | 7525 | ENSG00000176105 |
| 132 | 230875\_s\_at | NA | ATP11A | ATPase, class VI, type 11A | 23250 | ENSG00000068650 |
| 133 | 222317\_at | NA | PDE3B | phosphodiesterase 3B, cGMP-inhibited | 5140 | ENSG00000152270 |
| 134 | 1558956\_s\_at | NA | IFT80 | intraflagellar transport 80 homolog (Chlamydomonas) | 57560 | ENSG00000248710 ENSG00000068885 |
| 135 | 1557257\_at | NA | BCL10 | B-cell CLL/lymphoma 10 | 8915 | ENSG00000142867 |
| 136 | 201091\_s\_at | NA | CBX3 | chromobox homolog 3 | 11335 | ENSG00000122565 |
| 137 | 1559975\_at | NA | BTG1 | B-cell translocation gene 1, anti-proliferative | 694 | ENSG00000133639 |
| 138 | 1555476\_at | NA | IREB2 | iron-responsive element binding protein 2 | 3658 | ENSG00000136381 |
| 139 | 203884\_s\_at | NA | RAB11FIP2 | RAB11 family interacting protein 2 (class I) | 22841 | ENSG00000107560 |
| 140 | 227012\_at | NA | SLC25A40 | solute carrier family 25, member 40 | 55972 | ENSG00000075303 |
| 141 | 219304\_s\_at | NA | PDGFD | platelet derived growth factor D | 80310 | ENSG00000170962 |
| 142 | 214012\_at | NA | ERAP1 | endoplasmic reticulum aminopeptidase 1 | 51752 | ENSG00000164307 |
| 143 | 213372\_at | NA | PAQR3 | progestin and adipoQ receptor family member III | 152559 | ENSG00000163291 |
| 144 | 212633\_at | NA | UFL1 | UFM1-specific ligase 1 | 23376 | ENSG00000014123 |
| 145 | 207300\_s\_at | NA | F7 | coagulation factor VII (serum prothrombin conversion accelerator) | 2155 | ENSG00000057593 |

  
  

| **Database:cellular component      &nbspName:cytoplasmic part      &nbspID:GO:0044444** | | | | | | |
| --- | --- | --- | --- | --- | --- | --- |
| C=6542; O=100; E=78.23; R=1.28; rawP=0.0008; adjP=0.0175 | | | | | | |
| Index | UserID | Value | Gene Symbol | Gene Name | EntrezGene | Ensembl |
| 1 | 201304\_at | NA | NDUFA5 | NADH dehydrogenase (ubiquinone) 1 alpha subcomplex, 5, 13kDa | 4698 | ENSG00000128609 |
| 2 | 214658\_at | NA | TMED7 | transmembrane emp24 protein transport domain containing 7 | 51014 | ENSG00000134970 |
| 3 | 213725\_x\_at | NA | XYLT1 | xylosyltransferase I | 64131 | ENSG00000103489 |
| 4 | 227449\_at | NA | EPHA4 | EPH receptor A4 | 2043 | ENSG00000116106 |
| 5 | 201016\_at | NA | EIF1AX | eukaryotic translation initiation factor 1A, X-linked | 1964 | ENSG00000173674 |
| 6 | 224453\_s\_at | NA | ETNK1 | ethanolamine kinase 1 | 55500 | ENSG00000139163 |
| 7 | 225290\_at | NA | ETNK1 | ethanolamine kinase 1 | 55500 | ENSG00000139163 |
| 8 | 204187\_at | NA | GMPR | guanosine monophosphate reductase | 2766 | ENSG00000137198 |
| 9 | 226181\_at | NA | TUBE1 | tubulin, epsilon 1 | 51175 | ENSG00000074935 |
| 10 | 202660\_at | NA | ITPR2 | inositol 1,4,5-trisphosphate receptor, type 2 | 3709 | ENSG00000123104 |
| 11 | 218701\_at | NA | LACTB2 | lactamase, beta 2 | 51110 | ENSG00000147592 |
| 12 | 233019\_at | NA | CNOT7 | CCR4-NOT transcription complex, subunit 7 | 29883 | ENSG00000198791 |
| 13 | 213225\_at | NA | PPM1B | protein phosphatase, Mg2+/Mn2+ dependent, 1B | 5495 | ENSG00000138032 |
| 14 | 200602\_at | NA | APP | amyloid beta (A4) precursor protein | 351 | ENSG00000142192 |
| 15 | 1558692\_at | NA | C1orf85 | chromosome 1 open reading frame 85 | 112770 | ENSG00000198715 |
| 16 | 222528\_s\_at | NA | SLC25A37 | solute carrier family 25 (mitochondrial iron transporter), member 37 | 51312 | ENSG00000147454 |
| 17 | 226928\_x\_at | NA | SLC25A37 | solute carrier family 25 (mitochondrial iron transporter), member 37 | 51312 | ENSG00000147454 |
| 18 | 228527\_s\_at | NA | SLC25A37 | solute carrier family 25 (mitochondrial iron transporter), member 37 | 51312 | ENSG00000147454 |
| 19 | 226179\_at | NA | SLC25A37 | solute carrier family 25 (mitochondrial iron transporter), member 37 | 51312 | ENSG00000147454 |
| 20 | 225580\_at | NA | MRPL50 | mitochondrial ribosomal protein L50 | 54534 | ENSG00000136897 |
| 21 | 202314\_at | NA | CYP51A1 | cytochrome P450, family 51, subfamily A, polypeptide 1 | 1595 | ENSG00000001630 |
| 22 | 238020\_at | NA | PSMC2 | proteasome (prosome, macropain) 26S subunit, ATPase, 2 | 5701 | ENSG00000161057 |
| 23 | 230379\_x\_at | NA | C2orf56 | chromosome 2 open reading frame 56 | 55471 | ENSG00000003509 |
| 24 | 227375\_at | NA | ANKRD13C | ankyrin repeat domain 13C | 81573 | ENSG00000118454 |
| 25 | 206834\_at | NA | HBD | hemoglobin, delta | 3045 | ENSG00000223609 |
| 26 | 213459\_at | NA | RPL37A | ribosomal protein L37a | 6168 | ENSG00000197756 |
| 27 | 202422\_s\_at | NA | ACSL4 | acyl-CoA synthetase long-chain family member 4 | 2182 | ENSG00000068366 |
| 28 | 214590\_s\_at | NA | UBE2D1 | ubiquitin-conjugating enzyme E2D 1 | 7321 | ENSG00000072401 |
| 29 | 232068\_s\_at | NA | TLR4 | toll-like receptor 4 | 7099 | ENSG00000136869 |
| 30 | 201437\_s\_at | NA | EIF4E | eukaryotic translation initiation factor 4E | 1977 | ENSG00000151247 |
| 31 | 218871\_x\_at | NA | CSGALNACT2 | chondroitin sulfate N-acetylgalactosaminyltransferase 2 | 55454 | ENSG00000169826 |
| 32 | 226931\_at | NA | TMTC1 | transmembrane and tetratricopeptide repeat containing 1 | 83857 | ENSG00000133687 |
| 33 | 229943\_at | NA | TRIM13 | tripartite motif containing 13 | 10206 | ENSG00000204977 |
| 34 | 227900\_at | NA | CBLB | Cbl proto-oncogene, E3 ubiquitin protein ligase B | 868 | ENSG00000114423 |
| 35 | 202464\_s\_at | NA | PFKFB3 | 6-phosphofructo-2-kinase/fructose-2,6-biphosphatase 3 | 5209 | ENSG00000170525 |
| 36 | 222848\_at | NA | CENPK | centromere protein K | 64105 | ENSG00000123219 |
| 37 | 236539\_at | NA | PTPN22 | protein tyrosine phosphatase, non-receptor type 22 (lymphoid) | 26191 | ENSG00000134242 |
| 38 | 205283\_at | NA | FKTN | fukutin | 2218 | ENSG00000106692 |
| 39 | 223200\_s\_at | NA | LSG1 | large subunit GTPase 1 homolog (S. cerevisiae) | 55341 | ENSG00000041802 |
| 40 | 213404\_s\_at | NA | RHEB | Ras homolog enriched in brain | 6009 | ENSG00000106615 |
| 41 | 211546\_x\_at | NA | SNCA | synuclein, alpha (non A4 component of amyloid precursor) | 6622 | ENSG00000145335 |
| 42 | 207827\_x\_at | NA | SNCA | synuclein, alpha (non A4 component of amyloid precursor) | 6622 | ENSG00000145335 |
| 43 | 236081\_at | NA | SNCA | synuclein, alpha (non A4 component of amyloid precursor) | 6622 | ENSG00000145335 |
| 44 | 204467\_s\_at | NA | SNCA | synuclein, alpha (non A4 component of amyloid precursor) | 6622 | ENSG00000145335 |
| 45 | 204466\_s\_at | NA | SNCA | synuclein, alpha (non A4 component of amyloid precursor) | 6622 | ENSG00000145335 |
| 46 | 227697\_at | NA | SOCS3 | suppressor of cytokine signaling 3 | 9021 | ENSG00000184557 |
| 47 | 224009\_x\_at | NA | DHRS9 | dehydrogenase/reductase (SDR family) member 9 | 10170 | ENSG00000073737 |
| 48 | 223952\_x\_at | NA | DHRS9 | dehydrogenase/reductase (SDR family) member 9 | 10170 | ENSG00000073737 |
| 49 | 213524\_s\_at | NA | G0S2 | G0/G1switch 2 | 50486 | ENSG00000123689 |
| 50 | 201407\_s\_at | NA | PPP1CB | protein phosphatase 1, catalytic subunit, beta isozyme | 5500 | ENSG00000213639 |
| 51 | 203310\_at | NA | STXBP3 | syntaxin binding protein 3 | 6814 | ENSG00000116266 |
| 52 | 201088\_at | NA | KPNA2 | karyopherin alpha 2 (RAG cohort 1, importin alpha 1) | 3838 | ENSG00000182481 |
| 53 | 223649\_s\_at | NA | SLC25A39 | solute carrier family 25, member 39 | 51629 | ENSG00000013306 |
| 54 | 207545\_s\_at | NA | NUMB | numb homolog (Drosophila) | 8650 | ENSG00000133961 |
| 55 | 240336\_at | NA | HBM | hemoglobin, mu | 3042 | ENSG00000206177 |
| 56 | 222876\_s\_at | NA | ADAP2 | ArfGAP with dual PH domains 2 | 55803 | ENSG00000184060 |
| 57 | 236140\_at | NA | GCLM | glutamate-cysteine ligase, modifier subunit | 2730 | ENSG00000023909 |
| 58 | 202375\_at | NA | SEC24D | SEC24 family, member D (S. cerevisiae) | 9871 | ENSG00000150961 |
| 59 | 235412\_at | NA | ARHGEF7 | Rho guanine nucleotide exchange factor (GEF) 7 | 8874 | ENSG00000102606 |
| 60 | 212989\_at | NA | SGMS1 | sphingomyelin synthase 1 | 259230 | ENSG00000198964 |
| 61 | 214917\_at | NA | PRKAA1 | protein kinase, AMP-activated, alpha 1 catalytic subunit | 5562 | ENSG00000132356 |
| 62 | 213786\_at | NA | TAX1BP1 | Tax1 (human T-cell leukemia virus type I) binding protein 1 | 8887 | ENSG00000106052 |
| 63 | 1554309\_at | NA | EIF4G3 | eukaryotic translation initiation factor 4 gamma, 3 | 8672 | ENSG00000075151 |
| 64 | 217851\_s\_at | NA | SLMO2 | slowmo homolog 2 (Drosophila) | 51012 | ENSG00000101166 |
| 65 | 210746\_s\_at | NA | EPB42 | erythrocyte membrane protein band 4.2 | 2038 | ENSG00000166947 |
| 66 | 202843\_at | NA | DNAJB9 | DnaJ (Hsp40) homolog, subfamily B, member 9 | 4189 | ENSG00000128590 |
| 67 | 217739\_s\_at | NA | NAMPT | nicotinamide phosphoribosyltransferase | 10135 | ENSG00000105835 |
| 68 | 202887\_s\_at | NA | DDIT4 | DNA-damage-inducible transcript 4 | 54541 | ENSG00000168209 |
| 69 | 216929\_x\_at | NA | ABO | ABO blood group (transferase A, alpha 1-3-N-acetylgalactosaminyltransferase; transferase B, alpha 1-3-galactosyltransferase) | 28 | NULL |
| 70 | 204176\_at | NA | KLHL20 | kelch-like 20 (Drosophila) | 27252 | ENSG00000076321 |
| 71 | 202904\_s\_at | NA | LSM5 | LSM5 homolog, U6 small nuclear RNA associated (S. cerevisiae) | 23658 | ENSG00000106355 |
| 72 | 1569136\_at | NA | MGAT4A | mannosyl (alpha-1,3-)-glycoprotein beta-1,4-N-acetylglucosaminyltransferase, isozyme A | 11320 | ENSG00000071073 |
| 73 | 209451\_at | NA | TANK | TRAF family member-associated NFKB activator | 10010 | ENSG00000136560 |
| 74 | 222858\_s\_at | NA | DAPP1 | dual adaptor of phosphotyrosine and 3-phosphoinositides | 27071 | ENSG00000070190 |
| 75 | 203049\_s\_at | NA | TTC37 | tetratricopeptide repeat domain 37 | 9652 | ENSG00000198677 |
| 76 | 212637\_s\_at | NA | WWP1 | WW domain containing E3 ubiquitin protein ligase 1 | 11059 | ENSG00000123124 |
| 77 | 226965\_at | NA | FAM116A | family with sequence similarity 116, member A | 201627 | ENSG00000174839 |
| 78 | 208078\_s\_at | NA | SIK1 | salt-inducible kinase 1 | 150094 | ENSG00000142178 |
| 79 | 211760\_s\_at | NA | VAMP4 | vesicle-associated membrane protein 4 | 8674 | ENSG00000117533 |
| 80 | 1555962\_at | NA | B3GNT7 | UDP-GlcNAc:betaGal beta-1,3-N-acetylglucosaminyltransferase 7 | 93010 | ENSG00000156966 |
| 81 | 1555274\_a\_at | NA | EPT1 | ethanolaminephosphotransferase 1 (CDP-ethanolamine-specific) | 85465 | ENSG00000138018 |
| 82 | 202644\_s\_at | NA | TNFAIP3 | tumor necrosis factor, alpha-induced protein 3 | 7128 | ENSG00000118503 |
| 83 | 238199\_x\_at | NA | COX3 | cytochrome c oxidase III | 4514 | NULL |
| 84 | 202203\_s\_at | NA | AMFR | autocrine motility factor receptor, E3 ubiquitin protein ligase | 267 | ENSG00000159461 |
| 85 | 207794\_at | NA | CCR2 | chemokine (C-C motif) receptor 2 | 729230 | ENSG00000121807 |
| 86 | 1555878\_at | NA | RPS24 | ribosomal protein S24 | 6229 | ENSG00000138326 |
| 87 | 223266\_at | NA | STRADB | STE20-related kinase adaptor beta | 55437 | ENSG00000082146 |
| 88 | 211560\_s\_at | NA | ALAS2 | aminolevulinate, delta-, synthase 2 | 212 | ENSG00000158578 |
| 89 | 209686\_at | NA | S100B | S100 calcium binding protein B | 6285 | ENSG00000160307 |
| 90 | 230875\_s\_at | NA | ATP11A | ATPase, class VI, type 11A | 23250 | ENSG00000068650 |
| 91 | 202933\_s\_at | NA | YES1 | v-yes-1 Yamaguchi sarcoma viral oncogene homolog 1 | 7525 | ENSG00000176105 |
| 92 | 205063\_at | NA | GEMIN2 | gem (nuclear organelle) associated protein 2 | 8487 | ENSG00000092208 |
| 93 | 222317\_at | NA | PDE3B | phosphodiesterase 3B, cGMP-inhibited | 5140 | ENSG00000152270 |
| 94 | 203634\_s\_at | NA | CPT1A | carnitine palmitoyltransferase 1A (liver) | 1374 | ENSG00000110090 |
| 95 | 1558956\_s\_at | NA | IFT80 | intraflagellar transport 80 homolog (Chlamydomonas) | 57560 | ENSG00000248710 ENSG00000068885 |
| 96 | 204995\_at | NA | CDK5R1 | cyclin-dependent kinase 5, regulatory subunit 1 (p35) | 8851 | ENSG00000176749 |
| 97 | 1557257\_at | NA | BCL10 | B-cell CLL/lymphoma 10 | 8915 | ENSG00000142867 |
| 98 | 236487\_at | NA | SCLT1 | sodium channel and clathrin linker 1 | 132320 | ENSG00000151466 |
| 99 | 1555476\_at | NA | IREB2 | iron-responsive element binding protein 2 | 3658 | ENSG00000136381 |
| 100 | 203884\_s\_at | NA | RAB11FIP2 | RAB11 family interacting protein 2 (class I) | 22841 | ENSG00000107560 |
| 101 | 227012\_at | NA | SLC25A40 | solute carrier family 25, member 40 | 55972 | ENSG00000075303 |
| 102 | 202232\_s\_at | NA | EIF3M | eukaryotic translation initiation factor 3, subunit M | 10480 | ENSG00000149100 |
| 103 | 214012\_at | NA | ERAP1 | endoplasmic reticulum aminopeptidase 1 | 51752 | ENSG00000164307 |
| 104 | 219304\_s\_at | NA | PDGFD | platelet derived growth factor D | 80310 | ENSG00000170962 |
| 105 | 230559\_x\_at | NA | FGD4 | FYVE, RhoGEF and PH domain containing 4 | 121512 | ENSG00000139132 |
| 106 | 213372\_at | NA | PAQR3 | progestin and adipoQ receptor family member III | 152559 | ENSG00000163291 |
| 107 | 212633\_at | NA | UFL1 | UFM1-specific ligase 1 | 23376 | ENSG00000014123 |
| 108 | 207300\_s\_at | NA | F7 | coagulation factor VII (serum prothrombin conversion accelerator) | 2155 | ENSG00000057593 |
| 109 | 232044\_at | NA | RBBP6 | retinoblastoma binding protein 6 | 5930 | ENSG00000122257 |

  
  

| **Database:cellular component      &nbspName:cell      &nbspID:GO:0005623** | | | | | | |
| --- | --- | --- | --- | --- | --- | --- |
| C=13445; O=175; E=160.77; R=1.09; rawP=0.0005; adjP=0.0175 | | | | | | |
| Index | UserID | Value | Gene Symbol | Gene Name | EntrezGene | Ensembl |
| 1 | 228106\_at | NA | DCAF16 | DDB1 and CUL4 associated factor 16 | 54876 | ENSG00000163257 |
| 2 | 214658\_at | NA | TMED7 | transmembrane emp24 protein transport domain containing 7 | 51014 | ENSG00000134970 |
| 3 | 219312\_s\_at | NA | ZBTB10 | zinc finger and BTB domain containing 10 | 65986 | ENSG00000205189 |
| 4 | 227449\_at | NA | EPHA4 | EPH receptor A4 | 2043 | ENSG00000116106 |
| 5 | 201016\_at | NA | EIF1AX | eukaryotic translation initiation factor 1A, X-linked | 1964 | ENSG00000173674 |
| 6 | 235300\_x\_at | NA | RCHY1 | ring finger and CHY zinc finger domain containing 1, E3 ubiquitin protein ligase | 25898 | ENSG00000163743 |
| 7 | 226181\_at | NA | TUBE1 | tubulin, epsilon 1 | 51175 | ENSG00000074935 |
| 8 | 207078\_at | NA | MED6 | mediator complex subunit 6 | 10001 | ENSG00000133997 |
| 9 | 218701\_at | NA | LACTB2 | lactamase, beta 2 | 51110 | ENSG00000147592 |
| 10 | 202660\_at | NA | ITPR2 | inositol 1,4,5-trisphosphate receptor, type 2 | 3709 | ENSG00000123104 |
| 11 | 202219\_at | NA | SLC6A8 | solute carrier family 6 (neurotransmitter transporter, creatine), member 8 | 6535 | ENSG00000130821 |
| 12 | 222528\_s\_at | NA | SLC25A37 | solute carrier family 25 (mitochondrial iron transporter), member 37 | 51312 | ENSG00000147454 |
| 13 | 226928\_x\_at | NA | SLC25A37 | solute carrier family 25 (mitochondrial iron transporter), member 37 | 51312 | ENSG00000147454 |
| 14 | 228527\_s\_at | NA | SLC25A37 | solute carrier family 25 (mitochondrial iron transporter), member 37 | 51312 | ENSG00000147454 |
| 15 | 226179\_at | NA | SLC25A37 | solute carrier family 25 (mitochondrial iron transporter), member 37 | 51312 | ENSG00000147454 |
| 16 | 1558692\_at | NA | C1orf85 | chromosome 1 open reading frame 85 | 112770 | ENSG00000198715 |
| 17 | 203203\_s\_at | NA | KRR1 | KRR1, small subunit (SSU) processome component, homolog (yeast) | 11103 | ENSG00000111615 |
| 18 | 243916\_x\_at | NA | UBLCP1 | ubiquitin-like domain containing CTD phosphatase 1 | 134510 | ENSG00000164332 |
| 19 | 202314\_at | NA | CYP51A1 | cytochrome P450, family 51, subfamily A, polypeptide 1 | 1595 | ENSG00000001630 |
| 20 | 235683\_at | NA | SESN3 | sestrin 3 | 143686 | ENSG00000149212 |
| 21 | 226680\_at | NA | IKZF5 | IKAROS family zinc finger 5 (Pegasus) | 64376 | ENSG00000095574 |
| 22 | 238020\_at | NA | PSMC2 | proteasome (prosome, macropain) 26S subunit, ATPase, 2 | 5701 | ENSG00000161057 |
| 23 | 222243\_s\_at | NA | TOB2 | transducer of ERBB2, 2 | 10766 | ENSG00000183864 |
| 24 | 230379\_x\_at | NA | C2orf56 | chromosome 2 open reading frame 56 | 55471 | ENSG00000003509 |
| 25 | 213459\_at | NA | RPL37A | ribosomal protein L37a | 6168 | ENSG00000197756 |
| 26 | 205191\_at | NA | RP2 | retinitis pigmentosa 2 (X-linked recessive) | 6102 | ENSG00000102218 |
| 27 | 201437\_s\_at | NA | EIF4E | eukaryotic translation initiation factor 4E | 1977 | ENSG00000151247 |
| 28 | 232068\_s\_at | NA | TLR4 | toll-like receptor 4 | 7099 | ENSG00000136869 |
| 29 | 241881\_at | NA | OR2W3 | olfactory receptor, family 2, subfamily W, member 3 | 343171 | ENSG00000238243 |
| 30 | 218871\_x\_at | NA | CSGALNACT2 | chondroitin sulfate N-acetylgalactosaminyltransferase 2 | 55454 | ENSG00000169826 |
| 31 | 204299\_at | NA | SRSF10 | serine/arginine-rich splicing factor 10 | 10772 | ENSG00000188529 |
| 32 | 207996\_s\_at | NA | C18orf1 | chromosome 18 open reading frame 1 | 753 | ENSG00000168675 |
| 33 | 230170\_at | NA | OSM | oncostatin M | 5008 | ENSG00000099985 |
| 34 | 229943\_at | NA | TRIM13 | tripartite motif containing 13 | 10206 | ENSG00000204977 |
| 35 | 227900\_at | NA | CBLB | Cbl proto-oncogene, E3 ubiquitin protein ligase B | 868 | ENSG00000114423 |
| 36 | 222848\_at | NA | CENPK | centromere protein K | 64105 | ENSG00000123219 |
| 37 | 205283\_at | NA | FKTN | fukutin | 2218 | ENSG00000106692 |
| 38 | 223200\_s\_at | NA | LSG1 | large subunit GTPase 1 homolog (S. cerevisiae) | 55341 | ENSG00000041802 |
| 39 | 218401\_s\_at | NA | ZNF281 | zinc finger protein 281 | 23528 | ENSG00000162702 |
| 40 | 228785\_at | NA | ZNF281 | zinc finger protein 281 | 23528 | ENSG00000162702 |
| 41 | 213404\_s\_at | NA | RHEB | Ras homolog enriched in brain | 6009 | ENSG00000106615 |
| 42 | 213418\_at | NA | HSPA6 | heat shock 70kDa protein 6 (HSP70B') | 3310 | ENSG00000173110 |
| 43 | 223939\_at | NA | SUCNR1 | succinate receptor 1 | 56670 | ENSG00000198829 |
| 44 | 205900\_at | NA | KRT1 | keratin 1 | 3848 | ENSG00000167768 |
| 45 | 202861\_at | NA | PER1 | period homolog 1 (Drosophila) | 5187 | ENSG00000179094 |
| 46 | 203310\_at | NA | STXBP3 | syntaxin binding protein 3 | 6814 | ENSG00000116266 |
| 47 | 201088\_at | NA | KPNA2 | karyopherin alpha 2 (RAG cohort 1, importin alpha 1) | 3838 | ENSG00000182481 |
| 48 | 1553132\_a\_at | NA | TC2N | tandem C2 domains, nuclear | 123036 | ENSG00000165929 |
| 49 | 36711\_at | NA | MAFF | v-maf musculoaponeurotic fibrosarcoma oncogene homolog F (avian) | 23764 | ENSG00000185022 |
| 50 | 229431\_at | NA | RFXAP | regulatory factor X-associated protein | 5994 | ENSG00000133111 |
| 51 | 207545\_s\_at | NA | NUMB | numb homolog (Drosophila) | 8650 | ENSG00000133961 |
| 52 | 212847\_at | NA | FUBP1 | far upstream element (FUSE) binding protein 1 | 8880 | ENSG00000162613 |
| 53 | 240336\_at | NA | HBM | hemoglobin, mu | 3042 | ENSG00000206177 |
| 54 | 227391\_x\_at | NA | LRRFIP1 | leucine rich repeat (in FLII) interacting protein 1 | 9208 | ENSG00000124831 |
| 55 | 222876\_s\_at | NA | ADAP2 | ArfGAP with dual PH domains 2 | 55803 | ENSG00000184060 |
| 56 | 202375\_at | NA | SEC24D | SEC24 family, member D (S. cerevisiae) | 9871 | ENSG00000150961 |
| 57 | 214917\_at | NA | PRKAA1 | protein kinase, AMP-activated, alpha 1 catalytic subunit | 5562 | ENSG00000132356 |
| 58 | 204614\_at | NA | SERPINB2 | serpin peptidase inhibitor, clade B (ovalbumin), member 2 | 5055 | ENSG00000197632 |
| 59 | 217739\_s\_at | NA | NAMPT | nicotinamide phosphoribosyltransferase | 10135 | ENSG00000105835 |
| 60 | 202887\_s\_at | NA | DDIT4 | DNA-damage-inducible transcript 4 | 54541 | ENSG00000168209 |
| 61 | 206206\_at | NA | CD180 | CD180 molecule | 4064 | ENSG00000134061 |
| 62 | 202904\_s\_at | NA | LSM5 | LSM5 homolog, U6 small nuclear RNA associated (S. cerevisiae) | 23658 | ENSG00000106355 |
| 63 | 203552\_at | NA | MAP4K5 | mitogen-activated protein kinase kinase kinase kinase 5 | 11183 | ENSG00000012983 |
| 64 | 222765\_x\_at | NA | ESF1 | ESF1, nucleolar pre-rRNA processing protein, homolog (S. cerevisiae) | 51575 | ENSG00000089048 |
| 65 | 226965\_at | NA | FAM116A | family with sequence similarity 116, member A | 201627 | ENSG00000174839 |
| 66 | 208078\_s\_at | NA | SIK1 | salt-inducible kinase 1 | 150094 | ENSG00000142178 |
| 67 | 242109\_at | NA | SYTL3 | synaptotagmin-like 3 | 94120 | ENSG00000164674 |
| 68 | 1555962\_at | NA | B3GNT7 | UDP-GlcNAc:betaGal beta-1,3-N-acetylglucosaminyltransferase 7 | 93010 | ENSG00000156966 |
| 69 | 1555274\_a\_at | NA | EPT1 | ethanolaminephosphotransferase 1 (CDP-ethanolamine-specific) | 85465 | ENSG00000138018 |
| 70 | 202498\_s\_at | NA | SLC2A3 | solute carrier family 2 (facilitated glucose transporter), member 3 | 6515 | ENSG00000059804 |
| 71 | 238199\_x\_at | NA | COX3 | cytochrome c oxidase III | 4514 | NULL |
| 72 | 226279\_at | NA | PRSS23 | protease, serine, 23 | 11098 | ENSG00000150687 |
| 73 | 225189\_s\_at | NA | RAPH1 | Ras association (RalGDS/AF-6) and pleckstrin homology domains 1 | 65059 | ENSG00000173166 |
| 74 | 231863\_at | NA | ING3 | inhibitor of growth family, member 3 | 54556 | ENSG00000071243 |
| 75 | 223266\_at | NA | STRADB | STE20-related kinase adaptor beta | 55437 | ENSG00000082146 |
| 76 | 1555878\_at | NA | RPS24 | ribosomal protein S24 | 6229 | ENSG00000138326 |
| 77 | 209686\_at | NA | S100B | S100 calcium binding protein B | 6285 | ENSG00000160307 |
| 78 | 211560\_s\_at | NA | ALAS2 | aminolevulinate, delta-, synthase 2 | 212 | ENSG00000158578 |
| 79 | 205063\_at | NA | GEMIN2 | gem (nuclear organelle) associated protein 2 | 8487 | ENSG00000092208 |
| 80 | 203634\_s\_at | NA | CPT1A | carnitine palmitoyltransferase 1A (liver) | 1374 | ENSG00000110090 |
| 81 | 204995\_at | NA | CDK5R1 | cyclin-dependent kinase 5, regulatory subunit 1 (p35) | 8851 | ENSG00000176749 |
| 82 | 228063\_s\_at | NA | NAP1L5 | nucleosome assembly protein 1-like 5 | 266812 | ENSG00000177432 |
| 83 | 228062\_at | NA | NAP1L5 | nucleosome assembly protein 1-like 5 | 266812 | ENSG00000177432 |
| 84 | 218411\_s\_at | NA | MBIP | MAP3K12 binding inhibitory protein 1 | 51562 | ENSG00000151332 |
| 85 | 236487\_at | NA | SCLT1 | sodium channel and clathrin linker 1 | 132320 | ENSG00000151466 |
| 86 | 203574\_at | NA | NFIL3 | nuclear factor, interleukin 3 regulated | 4783 | ENSG00000165030 |
| 87 | 202232\_s\_at | NA | EIF3M | eukaryotic translation initiation factor 3, subunit M | 10480 | ENSG00000149100 |
| 88 | 205767\_at | NA | EREG | epiregulin | 2069 | ENSG00000124882 |
| 89 | 238614\_x\_at | NA | ZNF430 | zinc finger protein 430 | 80264 | ENSG00000118620 |
| 90 | 230559\_x\_at | NA | FGD4 | FYVE, RhoGEF and PH domain containing 4 | 121512 | ENSG00000139132 |
| 91 | 216248\_s\_at | NA | NR4A2 | nuclear receptor subfamily 4, group A, member 2 | 4929 | ENSG00000153234 |
| 92 | 204621\_s\_at | NA | NR4A2 | nuclear receptor subfamily 4, group A, member 2 | 4929 | ENSG00000153234 |
| 93 | 204622\_x\_at | NA | NR4A2 | nuclear receptor subfamily 4, group A, member 2 | 4929 | ENSG00000153234 |
| 94 | 222309\_at | NA | C6orf62 | chromosome 6 open reading frame 62 | 81688 | ENSG00000112308 |
| 95 | 232044\_at | NA | RBBP6 | retinoblastoma binding protein 6 | 5930 | ENSG00000122257 |
| 96 | 200608\_s\_at | NA | RAD21 | RAD21 homolog (S. pombe) | 5885 | ENSG00000164754 |
| 97 | 202221\_s\_at | NA | EP300 | E1A binding protein p300 | 2033 | ENSG00000100393 |
| 98 | 206919\_at | NA | ELK4 | ELK4, ETS-domain protein (SRF accessory protein 1) | 2005 | ENSG00000158711 |
| 99 | 208121\_s\_at | NA | PTPRO | protein tyrosine phosphatase, receptor type, O | 5800 | ENSG00000151490 |
| 100 | 201304\_at | NA | NDUFA5 | NADH dehydrogenase (ubiquinone) 1 alpha subcomplex, 5, 13kDa | 4698 | ENSG00000128609 |
| 101 | 203821\_at | NA | HBEGF | heparin-binding EGF-like growth factor | 1839 | ENSG00000113070 |
| 102 | 225760\_at | NA | MYSM1 | Myb-like, SWIRM and MPN domains 1 | 114803 | ENSG00000162601 |
| 103 | 222310\_at | NA | SCAF4 | SR-related CTD-associated factor 4 | 57466 | ENSG00000156304 |
| 104 | 213725\_x\_at | NA | XYLT1 | xylosyltransferase I | 64131 | ENSG00000103489 |
| 105 | 224453\_s\_at | NA | ETNK1 | ethanolamine kinase 1 | 55500 | ENSG00000139163 |
| 106 | 225290\_at | NA | ETNK1 | ethanolamine kinase 1 | 55500 | ENSG00000139163 |
| 107 | 202979\_s\_at | NA | CREBZF | CREB/ATF bZIP transcription factor | 58487 | ENSG00000137504 |
| 108 | 204187\_at | NA | GMPR | guanosine monophosphate reductase | 2766 | ENSG00000137198 |
| 109 | 233019\_at | NA | CNOT7 | CCR4-NOT transcription complex, subunit 7 | 29883 | ENSG00000198791 |
| 110 | 213225\_at | NA | PPM1B | protein phosphatase, Mg2+/Mn2+ dependent, 1B | 5495 | ENSG00000138032 |
| 111 | 200602\_at | NA | APP | amyloid beta (A4) precursor protein | 351 | ENSG00000142192 |
| 112 | 225580\_at | NA | MRPL50 | mitochondrial ribosomal protein L50 | 54534 | ENSG00000136897 |
| 113 | 225916\_at | NA | ZNF131 | zinc finger protein 131 | 7690 | ENSG00000172262 |
| 114 | 214741\_at | NA | ZNF131 | zinc finger protein 131 | 7690 | ENSG00000172262 |
| 115 | 201177\_s\_at | NA | UBA2 | ubiquitin-like modifier activating enzyme 2 | 10054 | ENSG00000126261 |
| 116 | 206834\_at | NA | HBD | hemoglobin, delta | 3045 | ENSG00000223609 |
| 117 | 227375\_at | NA | ANKRD13C | ankyrin repeat domain 13C | 81573 | ENSG00000118454 |
| 118 | 202422\_s\_at | NA | ACSL4 | acyl-CoA synthetase long-chain family member 4 | 2182 | ENSG00000068366 |
| 119 | 214590\_s\_at | NA | UBE2D1 | ubiquitin-conjugating enzyme E2D 1 | 7321 | ENSG00000072401 |
| 120 | 221768\_at | NA | SFPQ | splicing factor proline/glutamine-rich | 6421 | ENSG00000116560 |
| 121 | 226931\_at | NA | TMTC1 | transmembrane and tetratricopeptide repeat containing 1 | 83857 | ENSG00000133687 |
| 122 | 211698\_at | NA | EID1 | EP300 interacting inhibitor of differentiation 1 | 23741 | ENSG00000255302 |
| 123 | 202464\_s\_at | NA | PFKFB3 | 6-phosphofructo-2-kinase/fructose-2,6-biphosphatase 3 | 5209 | ENSG00000170525 |
| 124 | 240413\_at | NA | PYHIN1 | pyrin and HIN domain family, member 1 | 149628 | ENSG00000163564 |
| 125 | 236539\_at | NA | PTPN22 | protein tyrosine phosphatase, non-receptor type 22 (lymphoid) | 26191 | ENSG00000134242 |
| 126 | 226472\_at | NA | PPIL4 | peptidylprolyl isomerase (cyclophilin)-like 4 | 85313 | ENSG00000131013 |
| 127 | 227697\_at | NA | SOCS3 | suppressor of cytokine signaling 3 | 9021 | ENSG00000184557 |
| 128 | 211546\_x\_at | NA | SNCA | synuclein, alpha (non A4 component of amyloid precursor) | 6622 | ENSG00000145335 |
| 129 | 207827\_x\_at | NA | SNCA | synuclein, alpha (non A4 component of amyloid precursor) | 6622 | ENSG00000145335 |
| 130 | 236081\_at | NA | SNCA | synuclein, alpha (non A4 component of amyloid precursor) | 6622 | ENSG00000145335 |
| 131 | 204467\_s\_at | NA | SNCA | synuclein, alpha (non A4 component of amyloid precursor) | 6622 | ENSG00000145335 |
| 132 | 204466\_s\_at | NA | SNCA | synuclein, alpha (non A4 component of amyloid precursor) | 6622 | ENSG00000145335 |
| 133 | 213998\_s\_at | NA | DDX17 | DEAD (Asp-Glu-Ala-Asp) box helicase 17 | 10521 | ENSG00000100201 |
| 134 | 208719\_s\_at | NA | DDX17 | DEAD (Asp-Glu-Ala-Asp) box helicase 17 | 10521 | ENSG00000100201 |
| 135 | 208151\_x\_at | NA | DDX17 | DEAD (Asp-Glu-Ala-Asp) box helicase 17 | 10521 | ENSG00000100201 |
| 136 | 1555960\_at | NA | HINT1 | histidine triad nucleotide binding protein 1 | 3094 | ENSG00000169567 |
| 137 | 224009\_x\_at | NA | DHRS9 | dehydrogenase/reductase (SDR family) member 9 | 10170 | ENSG00000073737 |
| 138 | 223952\_x\_at | NA | DHRS9 | dehydrogenase/reductase (SDR family) member 9 | 10170 | ENSG00000073737 |
| 139 | 213524\_s\_at | NA | G0S2 | G0/G1switch 2 | 50486 | ENSG00000123689 |
| 140 | 209795\_at | NA | CD69 | CD69 molecule | 969 | ENSG00000110848 |
| 141 | 201407\_s\_at | NA | PPP1CB | protein phosphatase 1, catalytic subunit, beta isozyme | 5500 | ENSG00000213639 |
| 142 | 223649\_s\_at | NA | SLC25A39 | solute carrier family 25, member 39 | 51629 | ENSG00000013306 |
| 143 | 203603\_s\_at | NA | ZEB2 | zinc finger E-box binding homeobox 2 | 9839 | ENSG00000169554 |
| 144 | 204794\_at | NA | DUSP2 | dual specificity phosphatase 2 | 1844 | ENSG00000158050 |
| 145 | 236140\_at | NA | GCLM | glutamate-cysteine ligase, modifier subunit | 2730 | ENSG00000023909 |
| 146 | 226366\_at | NA | SHPRH | SNF2 histone linker PHD RING helicase, E3 ubiquitin protein ligase | 257218 | ENSG00000146414 |
| 147 | 206108\_s\_at | NA | SRSF6 | serine/arginine-rich splicing factor 6 | 6431 | ENSG00000124193 |
| 148 | 235412\_at | NA | ARHGEF7 | Rho guanine nucleotide exchange factor (GEF) 7 | 8874 | ENSG00000102606 |
| 149 | 212989\_at | NA | SGMS1 | sphingomyelin synthase 1 | 259230 | ENSG00000198964 |
| 150 | 213786\_at | NA | TAX1BP1 | Tax1 (human T-cell leukemia virus type I) binding protein 1 | 8887 | ENSG00000106052 |
| 151 | 220018\_at | NA | CBLL1 | Cbl proto-oncogene, E3 ubiquitin protein ligase-like 1 | 79872 | ENSG00000105879 |
| 152 | 1554309\_at | NA | EIF4G3 | eukaryotic translation initiation factor 4 gamma, 3 | 8672 | ENSG00000075151 |
| 153 | 217851\_s\_at | NA | SLMO2 | slowmo homolog 2 (Drosophila) | 51012 | ENSG00000101166 |
| 154 | 230380\_at | NA | THAP2 | THAP domain containing, apoptosis associated protein 2 | 83591 | ENSG00000173451 |
| 155 | 210746\_s\_at | NA | EPB42 | erythrocyte membrane protein band 4.2 | 2038 | ENSG00000166947 |
| 156 | 216834\_at | NA | RGS1 | regulator of G-protein signaling 1 | 5996 | ENSG00000090104 |
| 157 | 202988\_s\_at | NA | RGS1 | regulator of G-protein signaling 1 | 5996 | ENSG00000090104 |
| 158 | 202843\_at | NA | DNAJB9 | DnaJ (Hsp40) homolog, subfamily B, member 9 | 4189 | ENSG00000128590 |
| 159 | 203358\_s\_at | NA | EZH2 | enhancer of zeste homolog 2 (Drosophila) | 2146 | ENSG00000106462 |
| 160 | 208762\_at | NA | SUMO1 | SMT3 suppressor of mif two 3 homolog 1 (S. cerevisiae) | 7341 | ENSG00000116030 |
| 161 | 1558233\_s\_at | NA | ATF1 | activating transcription factor 1 | 466 | ENSG00000123268 |
| 162 | 216929\_x\_at | NA | ABO | ABO blood group (transferase A, alpha 1-3-N-acetylgalactosaminyltransferase; transferase B, alpha 1-3-galactosyltransferase) | 28 | NULL |
| 163 | 204176\_at | NA | KLHL20 | kelch-like 20 (Drosophila) | 27252 | ENSG00000076321 |
| 164 | 1569136\_at | NA | MGAT4A | mannosyl (alpha-1,3-)-glycoprotein beta-1,4-N-acetylglucosaminyltransferase, isozyme A | 11320 | ENSG00000071073 |
| 165 | 209451\_at | NA | TANK | TRAF family member-associated NFKB activator | 10010 | ENSG00000136560 |
| 166 | 222858\_s\_at | NA | DAPP1 | dual adaptor of phosphotyrosine and 3-phosphoinositides | 27071 | ENSG00000070190 |
| 167 | 203049\_s\_at | NA | TTC37 | tetratricopeptide repeat domain 37 | 9652 | ENSG00000198677 |
| 168 | 212637\_s\_at | NA | WWP1 | WW domain containing E3 ubiquitin protein ligase 1 | 11059 | ENSG00000123124 |
| 169 | 203543\_s\_at | NA | KLF9 | Kruppel-like factor 9 | 687 | ENSG00000119138 |
| 170 | 212579\_at | NA | SMCHD1 | structural maintenance of chromosomes flexible hinge domain containing 1 | 23347 | ENSG00000101596 |
| 171 | 215716\_s\_at | NA | ATP2B1 | ATPase, Ca++ transporting, plasma membrane 1 | 490 | ENSG00000070961 |
| 172 | 211760\_s\_at | NA | VAMP4 | vesicle-associated membrane protein 4 | 8674 | ENSG00000117533 |
| 173 | 202644\_s\_at | NA | TNFAIP3 | tumor necrosis factor, alpha-induced protein 3 | 7128 | ENSG00000118503 |
| 174 | 202203\_s\_at | NA | AMFR | autocrine motility factor receptor, E3 ubiquitin protein ligase | 267 | ENSG00000159461 |
| 175 | 207794\_at | NA | CCR2 | chemokine (C-C motif) receptor 2 | 729230 | ENSG00000121807 |
| 176 | 242960\_at | NA | EPC2 | enhancer of polycomb homolog 2 (Drosophila) | 26122 | ENSG00000135999 |
| 177 | 230875\_s\_at | NA | ATP11A | ATPase, class VI, type 11A | 23250 | ENSG00000068650 |
| 178 | 202933\_s\_at | NA | YES1 | v-yes-1 Yamaguchi sarcoma viral oncogene homolog 1 | 7525 | ENSG00000176105 |
| 179 | 222317\_at | NA | PDE3B | phosphodiesterase 3B, cGMP-inhibited | 5140 | ENSG00000152270 |
| 180 | 1558956\_s\_at | NA | IFT80 | intraflagellar transport 80 homolog (Chlamydomonas) | 57560 | ENSG00000248710 ENSG00000068885 |
| 181 | 201091\_s\_at | NA | CBX3 | chromobox homolog 3 | 11335 | ENSG00000122565 |
| 182 | 1557257\_at | NA | BCL10 | B-cell CLL/lymphoma 10 | 8915 | ENSG00000142867 |
| 183 | 1559975\_at | NA | BTG1 | B-cell translocation gene 1, anti-proliferative | 694 | ENSG00000133639 |
| 184 | 1555476\_at | NA | IREB2 | iron-responsive element binding protein 2 | 3658 | ENSG00000136381 |
| 185 | 203884\_s\_at | NA | RAB11FIP2 | RAB11 family interacting protein 2 (class I) | 22841 | ENSG00000107560 |
| 186 | 227012\_at | NA | SLC25A40 | solute carrier family 25, member 40 | 55972 | ENSG00000075303 |
| 187 | 239143\_x\_at | NA | RNF138 | ring finger protein 138, E3 ubiquitin protein ligase | 51444 | ENSG00000134758 |
| 188 | 214012\_at | NA | ERAP1 | endoplasmic reticulum aminopeptidase 1 | 51752 | ENSG00000164307 |
| 189 | 219304\_s\_at | NA | PDGFD | platelet derived growth factor D | 80310 | ENSG00000170962 |
| 190 | 213372\_at | NA | PAQR3 | progestin and adipoQ receptor family member III | 152559 | ENSG00000163291 |
| 191 | 212633\_at | NA | UFL1 | UFM1-specific ligase 1 | 23376 | ENSG00000014123 |
| 192 | 207300\_s\_at | NA | F7 | coagulation factor VII (serum prothrombin conversion accelerator) | 2155 | ENSG00000057593 |

  
  

| **Database:cellular component      &nbspName:lipopolysaccharide receptor complex      &nbspID:GO:0046696** | | | | | | |
| --- | --- | --- | --- | --- | --- | --- |
| C=5; O=2; E=0.06; R=33.45; rawP=0.0014; adjP=0.0245 | | | | | | |
| Index | UserID | Value | Gene Symbol | Gene Name | EntrezGene | Ensembl |
| 1 | 232068\_s\_at | NA | TLR4 | toll-like receptor 4 | 7099 | ENSG00000136869 |
| 2 | 1557257\_at | NA | BCL10 | B-cell CLL/lymphoma 10 | 8915 | ENSG00000142867 |

  
  
  
  


---

WebGestalt is currently developed and maintained by Jing Wang and Bing Zhang at the  Zhang Lab. Other people who have made significant contribution to the project include Dexter Duncan, Stefan Kirov, Zhiao Shi, and Jay Snoddy.  
  
**Funding credits:** NIH/NIAAA (U01 AA016662, U01 AA013512); NIH/NIDA (P01 DA015027); NIH/NIMH (P50 MH078028, P50 MH096972); NIH/NCI (U24 CA159988); NIH/NIGMS (R01 GM088822).
